# A new generic system for the pantropical Caesalpinia group (Leguminosae)

**DOI:** 10.3897/phytokeys.71.9203

**Published:** 2016-10-12

**Authors:** Edeline Gagnon, Anne Bruneau, Colin E. Hughes, Luciano Paganucci de Queiroz, Gwilym P. Lewis

**Affiliations:** 1 Institut de recherche en biologie végétale and Département de sciences biologiques, Université de Montréal, H1X 2B2, Montréal, Québec, Canada; 2 Department of Systematic and Evolutionary Botany, University of Zürich, 8008, Zürich, Switzerland; 3 Universidade Estadual de Feira de Santana, BR 116, Km 03, Campus Universitário, Feira de Santana 44031-460, Bahia, Brasil; 4 Comparative Plant and Fungal Biology Department, Royal Botanic Gardens, Kew, Richmond, Surrey, TW9 3AB, United Kingdom

**Keywords:** Mimosoideae-Caesalpinieae-Cassieae clade, Caesalpinioideae, Leguminosae, Fabaceae, generic delimitation, phylogeny, taxonomy

## Abstract

The Caesalpinia group is a large pantropical clade of ca. 205 species in subfamily Caesalpinioideae (Leguminosae) in which generic delimitation has been in a state of considerable flux. Here we present new phylogenetic analyses based on five plastid and one nuclear ribosomal marker, with dense taxon sampling including 172 (84%) of the species and representatives of all previously described genera in the Caesalpinia group. These analyses show that the current classification of the Caesalpinia group into 21 genera needs to be revised. Several genera (*Poincianella*, *Erythrostemon*, *Cenostigma* and *Caesalpinia* sensu Lewis, 2005) are non-monophyletic and several previously unclassified Asian species segregate into clades that merit recognition at generic rank. In addition, the near-completeness of our taxon sampling identifies three species that do not belong in any of the main clades and these are recognised as new monospecific genera. A new generic classification of the Caesalpinia group is presented including a key for the identification of genera, full generic descriptions, illustrations (drawings and photo plates of all genera), and (for most genera) the nomenclatural transfer of species to their correct genus. We recognise 26 genera, with reinstatement of two previously described genera (*Biancaea* Tod., *Denisophytum* R. Vig.), re-delimitation and expansion of several others (*Moullava*, *Cenostigma*, *Libidibia* and *Erythrostemon*), contraction of *Caesalpinia* s.s. and description of four new ones (*Gelrebia*, *Paubrasilia*, *Hererolandia* and *Hultholia*), and make 75 new nomenclatural combinations in this new generic system.

## Introduction

Resolving generic limits, reconciling genera with monophyletic groups and establishing stable generic classifications remain some of the most active and at times contentious issues in systematics (Humphreys and Linder 2009, Vences et al. 2013, Garnock-Jones 2014). This is very much the case in the large plant family Leguminosae, where delimitation of genera has been in a state of considerable flux, in large part because of the lack of robust and well-sampled species-level phylogenies (LPWG 2013, and LPWG submitted). In the past three decades, phylogenetic analyses of legume groups with adequate and representative species-level sampling have revealed the non-monophyly of numerous genera previously delimited using morphology alone (e.g. *Acacia* Mill. [e.g., Murphy 2008, Bouchenak-Khelladi et al. 2010; Miller and Seigler 2012], *Piptadenia* Benth. [Jobson and Luckow 2007], *Monopetalanthus* Harms [Wieringa 1999], *Hymenostegia* Harms [Mackinder et al. 2013; Mackinder and Wieringa 2013; Wieringa et al. 2013], *Vigna* Savi [Delgado-Salinas et al. 2011], *Lonchocarpus* Kunth [Da Silva et al. 2012], *Poecilanthe* Benth. [Meireles et al. 2014], *Derris* Lour. [Sirichamorn et al. 2014], *Otholobium* C.H. Stirt. [Egan and Crandall 2008; Dludlu et al. 2013], *Dioclea* Kunth, and *Galactia* P. Browne [De Queiroz et al. 2015]). In many other legume groups extensive non-monophyly of genera has been reported, but phylogenies with increased molecular and taxonomic sampling are necessary to provide the robust evidence needed to establish new generic systems (e.g. *Bauhinia* L., *Cynometra* L., *Maniltoa* Scheff., *Millettia* Wight & Arn., *Albizia* Durazz., *Archidendron* F. Muell., *Leucochloron* Barneby & J. W. Grimes, *Entada* Adans. (see LPWG 2013 and references therein).

**Figure 1.**
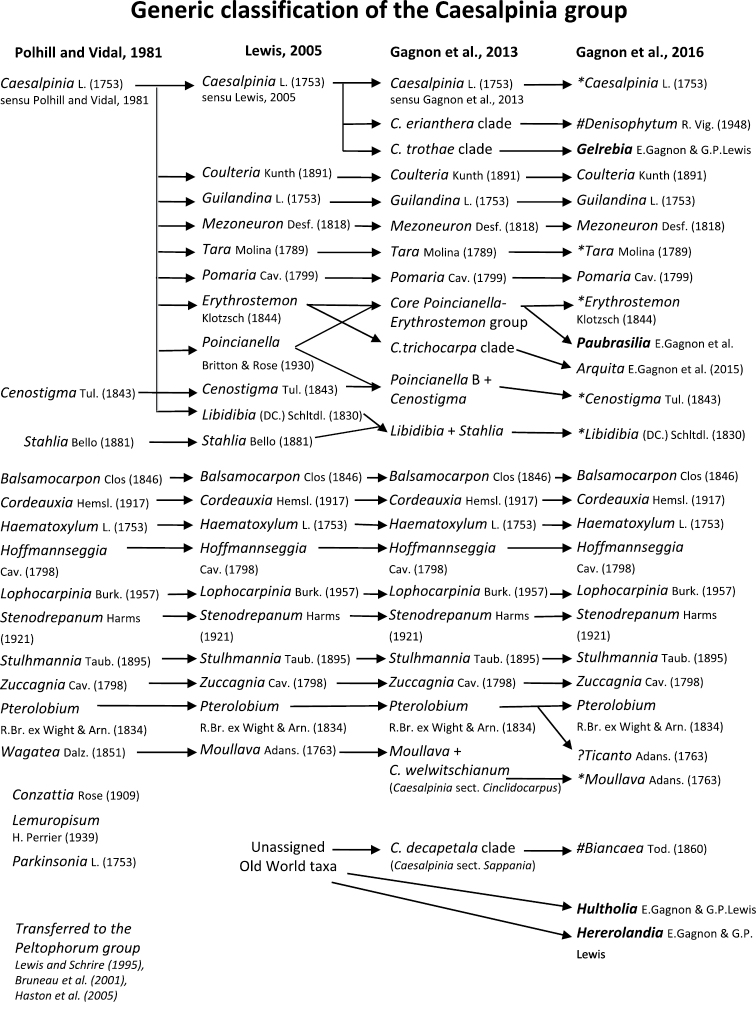
Comparison of generic classifications for the Caesalpinia group proposed by Polhill and Vidal (1981), Lewis (2005), Gagnon et al. (2013), and this study; names in bold represent new genera described here; prefix * indicates that the description of the genus is emended; prefix # indicates that the genus is being re-instated; prefix ? indicates that the status of the genus is uncertain.

The Caesalpinia group epitomises this generic flux, with persistent doubts about the delimitation of genera over the last 35 years (Gagnon et al. 2013; Fig. 1). This has been due to the difficulties of identifying diagnostic morphological synapomorphies and obtaining adequate sampling of taxa and genes in phylogenetic studies for this large pantropically distributed clade. The group is placed in the newly re-circumscribed subfamily Caesalpinioideae (LPWG submitted; equivalent to the Mimosoideae-Cassieae-Caesalpinieae, MCC clade *sensu* Doyle (2012); see also LPWG 2013), forming one of the informal groups in tribe Caesalpinieae. The Caesalpinia group was defined by Polhill and Vidal (1981) to include the genera with species that have a large variety of glandular trichomes, prickles and spines as a defense mechanism, and possessing zygomorphic flowers with a somewhat modified lower sepal and stamens crowded around the pistil. It is currently classified into 21 genera (Lewis 2005), but recent studies, and notably Gagnon et al. (2013, 2015), have demonstrated the non-monophly of some of these and the need for a new generic classification (Fig. 1). The group comprises ca. 205 species of small trees, woody shrubs and herbaceous subshrubs, with extremely diverse pollination and seed dispersal syndromes (the diversity of plant forms, flowers and fruits is extensively illustrated for all genera in the taxonomic acount), occurring predominantly in seasonally dry tropical forests and shrublands, but extending in a subset of clades into tropical and warm temperate savannas, tropical wet forests and tropical coastal habitats.

The genus *Caesalpinia* L. itself has been particularly problematic having been variously circumscribed by different authors. In its broadest sense *Caesalpinia* comprises ca. 150 species but these have had a tumultuous taxonomic and nomenclatural history, having been placed in up to 30 different genera since the description of the genus in 1753. These changing generic concepts illustrate the difficulties in establishing a stable classification of the group. The proliferation of generic names associated with *Caesalpinia* s. l. is due in part to the often complex, confusing and highly homoplastic nature of many morphological characters across the group, as well as the occurrence of many narrowly restricted endemics in a group with a pantropical distribution spanning five continents.

Previous molecular and morphologically-based phylogenetic analyses (Lewis and Schrire 1995, Simpson and Miao 1997, Simpson et al. 2003, Nores et al. 2012, Gagnon et al. 2013), including data from floral ontogeny (Kantz and Tucker 1994, Kantz 1996), phytochemistry (Kite and Lewis 1994), wood anatomy (Gasson et al. 2009), and leaf anatomy and secretory structures (Rudall et al. 1994, Lersten and Curtis 1994, 1996, Herendeen et al. 2003), attempted to more clearly delimit monophyletic genera within the Caesalpinia group. However, none of these studies achieved the comprehensive taxon sampling needed to fully understand and synthesize morphological diversity across the group as a whole. Other studies have focused on particular genera or clades, such as *Hoffmannseggia* Cav. (Simpson et al. 2004, 2005), *Pomaria* Cav. (Simpson et al. 2006), *Mezoneuron* Desf. (Clark and Gagnon 2015), and *Arquita* E. Gagnon, G. P. Lewis & C. E. Hughes (Gagnon et al. 2015). The most recent phylogenetic study (Gagnon et al. 2013), based on a single plastid marker (*rps16*) and sampling 120 of ca. 205 species (i.e. 58% taxon sampling), suggested that at least 23 genera would need to be recognised due to the non-monophyly of several genera, but lacked sufficient resolution and support as well as critical taxa (notably *Lophocarpinia* Burkart, *Stahlia* Bello, *Stenodrepanum* Harms, *Caesalpinia pearsonii* L. Bolus and *Caesalpinia glandulosa* Bertero ex DC.), to confidently propose a comprehensive new generic classification. Here we present a new phylogenetic analysis that samples the full morphological diversity and nearly the entire geographical range of the Caesalpinia group. This analysis is based on five plastid loci and the nuclear ribosomal ITS region, providing improved resolution and support over Gagnon et al. (2013). We use this densely sampled phylogenetic analysis to propose a new generic classification of the Caesalpinia group, in which we recognise 26 genera (with one additional clade tentatively suggested as a 27^th^ genus to be recognised pending additional taxon sampling), provide new or emended generic descriptions, a key to genera and, for genera where no further ambiguity as to species placements exists, the new nomenclatural combinations for species as required.

## Material and methods

### Taxon sampling

DNA was extracted from herbarium specimens and field-collected silica-dried leaves from wild and, in a few cases, cultivated plants. When possible, multiple individuals per species from different localities were sampled. In addition, previously published sequences (Bruneau et al. 2001, 2008, Simpson et al. 2003, 2005, 2006, Haston et al. 2005, Marazzi et al. 2006, Marazzi and Sanderson 2010, Manzanilla and Bruneau 2012, Nores et al. 2012, Babineau et al. 2013, Gagnon et al. 2013, 2015) were downloaded from GenBank (Appendix 1). All 21 genera belonging to the informal Caesalpinia group (sensu Lewis 2005), including all their type species (except for *Mezoneuron* Desf.), were sampled.

A total of 429 accessions representing 172 of the ca. 205 species (83.9%) of the Caesalpinia group, and including 131 species previously ascribed to the genus *Caesalpinia* s. l., were sequenced (Appendix 1). This sampling represents the full geographical range and morphological diversity of the group, with the important exception of seven species from mainland China for which no material was available for study. Several key species, whose phylogenetic and taxonomic affinities were previously unclear, including *Caesalpinia digyna* Rottler, *Caesalpinia tortuosa* Roxb., *Caesalpinia pellucida* Vogel, *Caesalpinia glandulosa*, and *Caesalpinia pearsonii*, are analysed here for the first time. Nine outgroup taxa spanning the MCC clade were included: *Gymnocladus chinensis* Baill., *Tetrapterocarpon geayi* Humbert (Umtiza grade), *Colvillea racemosa* Bojer, *Conzattia multiflora* (B.L. Rob.) Standl. (Peltophorum group) and *Cassia javanica* L., *Pterogyne nitens* Tul., *Senna alata* (L.) Roxb., *Senna covesii* (A. Gray) H.S. Irwin & Barneby and *Senna spectabilis* (DC.) H.S. Irwin & Barneby (Cassieae clade).

### Molecular methods

Three protocols were used to extract DNA: (1) a modified CTAB protocol (Joly and Bruneau 2006); (2) QIAGEN DNeasy Plant Mini Kit (Mississauga, ON, Canada); or (3) a 4% MATAB protocol (Ky et al. 2000). Six genetic markers were amplified, including the 5.8S subunit and flanking internal transcribed spacers, ITS1 and ITS2, of nuclear ribosomal DNA, and five plastid loci: *rps16*, the *trnD-trnT* intergenic spacer, *ycf6-psbM*, the *matK* gene and flanking *3’-trnK* intron, and the *trnL-trnF* intron-spacer region. The first four markers were amplified using both standard and nested-PCR protocols, described in Gagnon et al. (2015). The *matK*-3’-*trnK* region was amplified using the primers trnK685F (Hu et al. 2000), trnK4La (Wojciechowski et al. 2004), trnK2R* and KC6 (Bruneau et al. 2008), following the protocols described in Bruneau et al. (2008). Because of initially poor amplifications, we designed a new primer, matK-C6-Caesalpinia (5’-GAA TGC TCG GAT AAT TGG TTT-3’), which improved the amplification of the 5’section of this locus. The *trnL-trnF* intron-spacer region was amplified using the primers trnL-C, -D, -E and -F (Taberlet et al. 1991), using the same protocols as for the *rps16* locus (Gagnon et al. 2013), with annealing temperatures varying between 50 and 53 °C. While we attempted to amplify the first four loci for all available material, for the *matK*-3’*trnK* and *trnL-trnF* regions we sequenced a targeted subset of taxa to complement existing data. For problematic samples, including those presenting sequencing problems due to mononucleotide repeats, we used a protocol with Phusion Hot Start II High-Fidelity DNA polymerase (Thermo Scientific, United States), as described by Gagnon et al. (2013), which yields more accurate and longer quality mononucleotide sequence reads (Fazekas et al. 2010).

PCR amplifications were sequenced by Genome Quebec (Montreal, Canada), with Big Dye Terminator 3.1 chemistry on an ABI 3730xl DNA Analyzer (Applied Biosystems, Carlsbad, CA, USA). Geneious (version 5.6-6.1.8, Biomatters, Auckland, New Zealand) was used to assemble chromatograms and inspect and edit contigs. All sequences were submitted to BLAST (Altschul et al. 1990) to verify for non-specific amplification, and eliminated if they did not match Leguminosae sequences in GenBank. GenBank numbers with corresponding locality details and herbarium vouchers are listed in Appendix 1.

### Phylogenetic analyses

Sequences were aligned, inspected and manually adjusted using Geneious, and the resulting matrices are available from Dryad Digital Repository (doi: 10.5061/dryad.f4h2h). Regions of ambiguous alignment corresponding mostly to variable mononucleotide and/or tandem repeats were excluded as follows: 42 nucleotides for *ITS*, 92 for *rps16*, 146 for *trnD-trnT*, 157 for *ycf6-psbM*, 86 for *trnL-trnF* and 16 for *matK-3’trnK*. Gaps were coded using simple indel coding (Simmons and Ochoterena 2000) in SeqState 1.4.1 (Müller 2005), retaining only non-autapomorphic indels.

Phylogenetic analyses were carried out on each of the six loci individually and on two concatenated matrices, one with the five plastid loci and a second matrix with all six loci (plastid + ITS). Matrices were concatenated using SequenceMatrix (Vaidya et al. 2011). We used a Maximum Likelihood (ML) approach using RaxML 8.0.0 (Stamatakis 2014) on the CIPRES gateway v.3.3 (Miller et al. 2010). The analyses were conducted using the GTRGAMMA model for the DNA sequences and the BINCAT model for the indel partitions. Bootstrap support was assessed through 1000 non-parametric bootstrap replicates.

Because topological conflicts amongst the six individual gene trees were minimal, and where differences were found these were always only weakly supported (< 60% BS), all subsequent analyses were done on the six-locus concatenated matrix. Initial analyses of this six-locus matrix keeping all accessions of species as separate terminals resulted in a matrix with significant missing data because not all accessions were sequenced for all loci (see Tables 1 and 2). To reduce missing data, multiple accessions of the same species were concatenated if they occurred in the same clade in the preliminary RaxML analyses to maximize the number of loci represented for a species. When more than one sequence per species was available for a given locus, the longest sequence was selected, because we never found any sequence variation in the overlapping sections. This resulted in concatenation of accessions for 16 species (see Appendix 1): *Caesalpinia cacalaco* Bonpl., *Caesalpinia caladenia* Standl., *Caesalpinia caudata* (A. Gray) Fisher, *Caesalpinia colimensis* F. J. Herm., *Caesalpinia epifanioi* J. L. Contr., *Caesalpinia exilifolia* Griseb., *Caesalpinia madagascariensis* (R. Vig.) Senesse, *Caesalpinia melanadenia* (Rose) Standl., *Caesalpinia mimosoides* Lam., *Caesalpinia pringlei* (Britton & Rose) Standl., *Caesalpinia sappan* L., *Caesalpinia sessilifolia* S. Watson, *Libidibia sclerocarpa* (Standl.) Britton & Rose, *Haematoxylum brasiletto* H. Karst., *Haematoxylum dinteri* Harms and *Tara spinosa* (Molina) Britton & Rose. In addition to concatenating sequences obtained from different accessions of a species, preliminary analyses showed lack of resolution for a few accessions for which only one or two loci were sequenced. To explore the impacts of different levels of missing data, a series of matrices that progressively excluded accessions with five, four, three, two and one missing loci were generated, resulting in six different concatenated matrices (Table 2). Because the matrix containing sequences with no missing data lacked representatives from a number of genera and critical clades or species, a seventh matrix was generated (with 39 taxa) that added an accession from each of these critical taxa to maximise taxonomic representation while minimizing missing data.

**Table 1.** Character statistics for the six loci analysed, with the number of accessions for each locus, aligned length (including ambiguous alignment regions), number of indels scored, numbers and % of parsimony informative characters (for both DNA and indel characters), and critical missing genera and taxa.

Locus	Number of accessions	Aligned length	Number of informative indels	Numbers and % parsimony informative characters	Critical missing genera and taxa
*ITS*	251	820	113	550/891 = 62%	*Caesalpinia mimosoidesLophocarpiniaStenodrepanumStahlia*
*rps16*	298	1081	45	311/1034 = 30%	*LophocarpiniaStenodrepanum*
*trnD-trnT*	235	1921	108	513/1883 = 27%	*LophocarpiniaStenodrepanum*
*ycf6-psbM*	193	1795	141	540/1779 = 30%	*LophocarpiniaStenodrepanum*
*trnL-trnF*	171	1347	65	307/1326 =23%	None
*matK-3’trnK*	89	1839	20	308/1843 =17%	*Caesalpinia mimosoides*

**Table 2.** Statistics for the seven combined matrices, with the number of accessions, number of ingroup and outgroup species, % missing data, and missing genera/critical taxa. The results of the parsimony analyses are indicated, with the number of trees retained, the length of the shortest trees (length), consistency index (CI), and retention index (RI).

	All sequences	2 loci +	3 loci +	4 loci +	5 loci +	All 6 loci +	No missing genera
**Accessions**	408	312	223	175	76	30	39
**Nb. of Caesalpinia group species**	171/~205	163/~205	128/~205	103/~205	55/~205	26/~205	35/~205
**Nb. *Caesalpinia* s.l. species**	130/~155	123/~155	106/~155	84/~155	44/~155	20/~155	23/~155
**Outgroup species**	9	9	9	9	8	4	4
% **missing data**	61%	53%	43%	38%	28%	23%	30%
**Missing genera/critical taxa**	None	None	*2*: *Lophocarpinia*, *Stenodrepanum*	*2*: *Lophocarpinia*, *Stenodrepanum*	*3*: *Lophocarpinia*, *Stenodrepanum*, *Caesalpinia mimosoides*	*8*: *Caesalpinia mimosoides*, *Cenostigma*, *Guilandina*, *Moullava*, *Lophocarpinia*, *Pterolobium*, *Stahlia*, *Stenodrepanum*	None
**Nb trees found**	50,000	50,000	50,000	50,000	7	2	2
**Length**	12,212	11,986	10,909	10,101	7,615	4,715	5405
**CI**	0.43	0.45	0.45	0.47	0.53	0.62	0.60
**RI**	0.81	0.81	0.79	0.78	0.66	0.49	0.48

For these seven concatenanted matrices, phylogenetic analyses were carried out using ML, maximum parsimony (MP) and Bayesian methods. For the ML analyses, we used RaxML (Stamatakis 2014) as described above. For MP analyses, PAUP* (Swofford 2003) was used with a two-step approach (Davis et al. 2004) as described in Gagnon et al. (2013), but saving a maximum of 50,000 trees with 5,000 bootstrap replicates, with two trees retained per replicate. Bayesian analyses were conducted in MrBayes 3.2 (Ronquist et al. 2012) using MrModeltest v.2.3 (Nylander 2004) to select the GTR + I + G model for all six loci and the F81-like model for the indel partition. Analyses were run on a high performance computer cluster (Calcul Québec, Université de Montréal, Canada) with two parallel runs of eight Markov Chain Monte Carlo (MCMC) chains, four swaps per swapping cycle, and trees sampled every 1000 generations. The stop criterion was set to an average standard deviation of split frequencies that dropped to below the critical value of 0.01. Tracer v.1.6 (Rambaut et al. 2014) was used to ensure effective sample sizes were above 200 and that chains mixed appropriately, with 510,000 and 27 million generations, depending on the size of the matrix. The “burn-in” fraction for all analyses was set to 10%.

## Results

Of the six loci, ITS had the highest proportion of parsimony-informative characters (61.7%), followed by *ycf6-psbM*, *rps16*, *trnD-trnT*, *trnL-trnC*, and *matK-3’trnK* (Table 1). The concatenated six-locus matrix (aligned length = 8803 bp) included 429 accessions, which was reduced to 408 when accessions were combined for 16 species (see above). Table 2 summarises the number of accessions and species per locus, the percentage of missing data, the number of trees, tree length, CI and RI obtained in the MP analyses for the series of seven concatenated matrices with successively lower numbers of taxa with missing loci.

With the exception of the least informative (*trnL-trnF*) gene tree, which is poorly resolved (data not shown), the Caesalpinia group is monophyletic in all analyses, generally with high bootstrap and PP support (see Suppl. material 1). The 23 major clades identified from the *rps16* phylogeny by Gagnon et al. (2013; Fig. 1) are also generally recovered in each of the individual ML gene trees (Suppl. material 1), as well in the analyses of the matrices combining all six loci, with two notable exceptions. First, in the MP and ML analyses, *Lophocarpinia* is nested within *Haematoxylum*, but in the Bayesian analyses *Lophocarpinia* is sister to *Haematoxylum*. Second, the genus *Pterolobium* is also sometimes recovered as non-monophyletic, with *Caesalpinia crista* nested within it in some of the MP, ML and Bayesian analyses, while in other analyses it is recovered as monophyletic, but with poor to moderate support in the ML and Bayesian analyses of all six loci, with a minimum of 2 to 3 loci per accession (Suppl. material 1).

**Figure 2.**
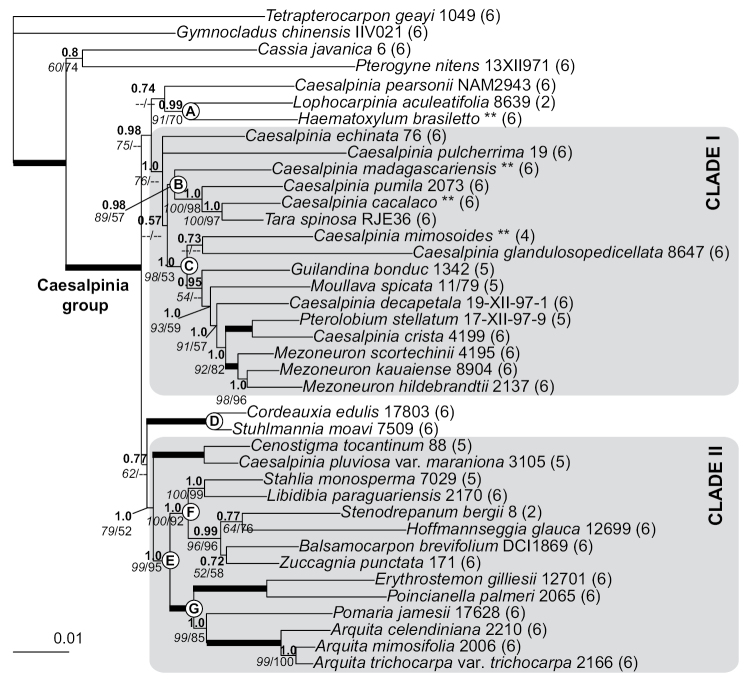
Phylogeny of the Caesalpinia group. Bayesian phylogram based on 39 accessions, minimizing missing data while maximizing the taxonomic representation of each of the 27 putative genera within the Caesalpinia group. Branch support values are indicated as follows: branches in bold indicate that maximum support has been attained in the parsimony, Maximum Likelihood and Bayesian analyses; otherwise, posterior probabilities are indicated above in bold, with bootstrap support from ML analyses (italicised) and parsimony analyses separated by a slash below the branches.

**Figure 3.**
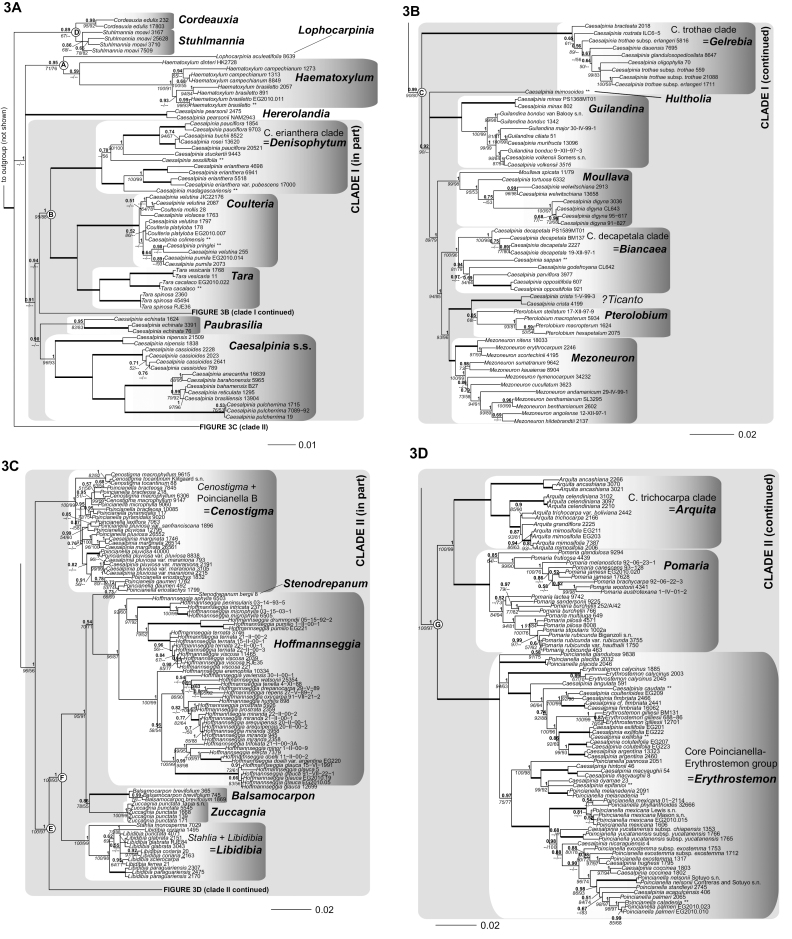
**A–D** Phylogeny of the Caesalpinia group. Bayesian phylogram based on 312 accessions, including only accessions with two or more loci. Branch support values are indicated as follows: branches in bold indicate that maximum support has been attained in the MP, ML and Bayesian phylogenetic analyses; otherwise, posterior probabilities are indicated above in bold, with bootstrap support from ML analyses (italicised) and parsimony analyses separated by a slash below the branches; for each terminal, the species name is followed by the collector number of the corresponding voucher (see Appendix 1 for full voucher details); the suffix ** indicates that several sequences from different accessions of the same species were concatenated for analysis (see Appendix 1 for details); for major clades and genera, the names used by Gagnon et al. (2013) are indicated, as well as the corresponding new genera.

In addition to these 23 clades (Fig. 1; see Gagnon et al. 2013), four other clades or monospecific lineages were consistently recovered in the MP, ML, and Bayesian analyses of the matrices with all six loci (Suppl. material 1): the three monospecific *Caesalpinia echinata*, *Caesalpinia mimosoides* and *Caesalpinia pearsonii* lineages, and the *Caesalpinia crista* clade, corresponding to Caesalpinia sect. Nugaria, represented by *Caesalpinia crista* and *Caesalpinia vernalis* in the *rps*16 gene tree of Gagnon et al. (2013), although it is important to note that *Caesalpinia vernalis* was excluded from later analyses of the concatenated matrices due to missing data and does not appear in Fig. 2 or Fig. 3. In total, this resulted in 27 possible genera in the Caesalpinia group, 26 of which are recognised here (see below). In addition, the MP, ML and Bayesian phylogenies based on the various concatenated datasets were generally congruent as to the relationships amongst these 27 lineages, regardless of the proportion of missing data, or number of missing genera/critical species. Minor differences observed between the topologies lacked support.

Given this congruence among the ML, MP and Bayesian analyses, only the Bayesian topology is presented (Figs 2 and 3A–D) and forms the basis for all subsequent discussion. The first diverging lineages of the Caesalpinia group comprise the species *Caesalpinia pearsonii*, the *Lophocarpinia* + *Haematoxylum* clade (Figs 2 and 3A, clade A), and the *Cordeauxia* + *Stuhlmannia* clade (Figs 2 and 3A, clade D). All other genera were placed in two large and robustly supported clades here designated **clades I** and **II** (Figs 2 and 3). **Clade I** (Figs 2 and 3A–B) includes *Caesalpinia echinata*, *Caesalpinia* s. s., a clade comprising *Tara* + *Coulteria* + the *Caesalpinia erianthera* clade (Figs 2 and 3A, clade B), as well as a group corresponding to the *Caesalpinia trothae* clade and all lineages consisting predominantly of Asian liana species (*Caesalpinia mimosoides* + *Guilandina* + *Moullava* + the *Caesalpinia decapetala* clade + the *Caesalpinia crista* clade + *Pterolobium* + *Mezoneuron*) (Figs 2 and 3B, clade C). **Clade II** (Figs 2 and 3C–D) includes the *Cenostigma*-Poincianella B clade as sister to a clade (Figs 2 and 3C, clade E) containing two main lineages: the first comprising *Stahlia* + *Libidibia*, *Balsamocarpon* + *Zuccagnia* + *Stenodrepanum* + *Hoffmannseggia* (Figs 2 and 3C, clade F), and the second made up of the core Poincianella-Erythrostemon group + *Pomaria* + *Arquita* (Figs 2 and 3D, clade G).

Although all 27 lineages and all 26 genera are robustly supported, the precise relationships amongst a few genera remain unresolved or are not supported. For example, the position of *Caesalpinia echinata* lacks support in both the MP and ML analyses (bootstrap support below 50%), while in the Bayesian analyses it is sometimes resolved as sister to *Caesalpinia* s. s. (PP between 64 and 97), emphasising that this species is phylogenetically isolated and justifying its recognition as a new genus (see below). Similarly, the relationships between *Caesalpinia mimosoides*, the *Caesalpinia trothae* clade, and *Guilandina* are sometimes resolved, but generally with low support, again pointing to the phylogenetic distinctiveness of *Caesalpinia mimosoides*. Within the core Poincianella-Erythrostemon clade, the relationships of *Caesalpinia placida* and *Caesalpinia glandulosa* are unstable, being placed either as sister to a Central American lineage or to a South American lineage. Finally, the position of *Stenodrepanum* as sister to *Hoffmannseggia* is consistent across all analyses, but always with low branch support (Fig. 3C).

## Discussion

In his generic classification of *Caesalpinia* s. l., Lewis (2005) suggested that molecular phylogenies with increased taxon sampling were needed to rigourously test the monophyly of the genera he was reinstating and to resolve the relationships of a group of 12 to15 Asian species that could not be placed in any of the proposed segregates. Whilst several recent studies based on single DNA sequence loci or morphology have partially addressed this problem (Simpson et al. 2003, Nores et al. 2012, Gagnon et al. 2013), the results presented here, based on combined analyses of six DNA sequence loci totaling 8.8 kb of DNA sequence data, and sampling 84% of species, provide the most comprehensively sampled and robust phylogeny of the group to date. As seen in many other species-level phylogenetic studies of legume taxa (e.g. Moura et al. 2016, Rando et al. 2016, Simon et al. 2016), the most informative DNA sequence locus is ITS, which has at least twice as many informative characters as the plastid loci included in this study. Near-complete sampling of species across the Caesalpinia group, provides a much more stringent and comprehensive assessment of the monophyly of the subclades, as well as of the homology and interpretation of morphological character evolution within the group. Furthermore, as found in both empirical and simulation studies of other taxa (Wiens 2003, 2006, Phillipe et al. 2004, Pyron et al. 2011, Johnson et al. 2012, Hinchliff and Roalson 2013), the concatenated supermatrix approach used here is shown to be robust to missing data. Of the 21 genera proposed by Lewis (2005; Fig. 1), it is clear that some of these groups, such as the Poincianella-Erythrostemon group (Lewis, 1998), *Caesalpinia* sensu Lewis (2005) and *Cenostigma* are non-monophyletic. Our analyses also reveal additional clades of Asian species that do not correspond to any of the genera in the Lewis (2005) classification system. In addition, three species (*Caesalpinia echinata*, *Caesalpinia mimosoides* and *Caesalpinia pearsonii*) are placed outside the clades corresponding to the genera proposed by Lewis (2005) or Gagnon et al. (2013) and comprise phylogenetically isolated monospecific lineages. Based on this new and much more comprehensively sampled phylogeny, thorough review of the literature and detailed survey of the morphological diversity of the group, we propose a new classification recognizing 26 genera corresponding to robustly supported clades found across analyses regardless of the amount of missing data. We also discuss the possibility of recognizing a 27^th^ genus, but more molecular and field sampling, especially of freshly collected field specimens, are needed before naming this clade at generic rank.

### Phylogenetic relationships and generic delimitation

In their description of the Caesalpinia group, Polhill and Vidal (1981) remarked that this was one of the most distinctive of the nine informal generic groups in tribe Caesalpinieae, based on several morphological characters, and notably the presence of a lower cucullate sepal on the calyx. Although they included the genera *Conzattia*, *Lemuropisum* and *Parkinsonia* in the Caesalpinia group, these were subsequently shown to belong to the Peltophorum group (Haston et al. 2005). The Caesalpinia group, as circumscribed by Lewis (2005), is here shown to form a robustly supported clade (Figs 2 and 3). All of the 13 genera outside *Caesalpinia* s. l. form robustly supported monophyletic groups, except *Moullava* and *Cenostigma*, which are both recircumscribed and expanded to include extra species that were previously placed in *Caesalpinia* s.l. Of the original eight genera re-instated by Lewis (2005), five (*Tara*, *Coulteria*, *Guilandina*, *Mezoneuron*, and *Libidibia*) also form robust clades in our analyses. These five genera are clearly defined by diagnostic morphological synapomorphies, as discussed in Gagnon et al. (2013).

*Libidibia* shares many similarities with the monotypic *Stahlia* from the Caribbean, the two together forming a robustly supported clade (Figs 2 and 3C), prompting re-evaluation of their status as distinct genera. *Stahlia* has been distinguished by its somewhat fleshy red fruits (Fig. 32A) and singly pinnate leaves. However, the pods of *Stahlia* are similar to those of some species of *Libidibia* (especially *Libidibia sclerocarpa* and some South American species) in terms of shape and lack of dehiscence (Fig. 32A–C and F). All other closely related genera have dehiscent pods. *Stahlia* has also been differentiated from *Libidibia* by the presence of pinnate rather than bipinnate leaves as in *Libidibia*, but the dark punctate gland dots on the undersurface of the leaflets, which are distinctively aligned parallel to the midvein, are also observed in certain species of *Libidibia*, including *Libidibia coriaria* and *Libidibia ferrea* (Simpson et al. 2003, Nores et al. 2012, Gagnon et al. 2013). Elsewhere in the Caesalpinia group, leaf pinnation and the occurrence of pinnate vs. bipinnate leaves can be extremely labile within genera (e.g. *Haematoxylum* and *Cenostigma*), within species (e.g. *Stuhlmannia moavi*), and even within individuals (e.g. *Haematoxylum sousanum* Cruz Durán & J. Jiménez Ramirez (Durán and Ramirez 2008)). Given these morphological similarities and the apparent lability of leaf division, we conclude that there is no justification for retaining *Stahlia* and *Libidibia* as separate genera.

As found previously by Gagnon et al. (2013, 2015), the other three genera recognised by Lewis (2005), *Poincianella*, *Erythrostemon* and *Caesalpinia* s. s., are not supported as monophyletic (Fig. 3A, D). Although Lewis (1998) considered that *Poincianella* and *Erythrostemon* together formed a clade, Gagnon et al. (2013, 2015) plus the more densely sampled phylogeny presented here (Fig. 3), show that their species fall into unrelated clades, providing the basis for recognition of three genera. First, a subset of *Poincianella* species corresponding to the Poincianella B group of Lewis and Schrire (1995) group with *Cenostigma* (Fig. 3C), as found in the morphological cladistic analysis of Lewis and Schrire (1995). These Poincianella B species differ from the remaining *Poincianella* and *Erythrostemon* species in wood anatomy (Gasson et al. 2009) and in their alternate to subopposite leaflets (De Queiroz 2009). While *Cenostigma* was originally considered as a distinct genus, in part based on its pinnate leaves, two species of the Poincianella B clade (*Caesalpinia marginata* and *Caesalpinia pinnata*) also have pinnate leaves. More importantly, several species of Poincianella B have internal secretory cavities in the leaflet lamina and inflorescences (Lersten and Curtis 1994; Rudall et al. 1994), as well as a stellate indumentum on the stems, leaves and/or inflorescences, both of which are considered as diagnostic characters of *Cenostigma*. These leaf traits are completely lacking in the core Poincianella-Erythrostemon group. In addition, Poincianella B and *Cenostigma* share robust pods with conspicuously thickened margins (Fig. 30B–E and G), which are absent in the other species of the Poincianella-Erythrostemon group and provide a diagnostic synapomorphy for an expanded *Cenostigma* including the Poincianella B species. It thus appears that in this group morphological homoplasy (pinnation of leaves, alternate to subopposite leaflets, the presence/absence of stipitate glands, stellate indumentum) has obscured relationships resulting in non-monophyletic genera. Here we expand *Cenostigma* to include the subset of Poincianella-Erythrostemon group species formerly assigned to Poincianella B by Lewis and Schrire (1995; Fig. 3C).

The remaining species of the former *Poincianella* and *Erythrostemon* are placed either in an Andean clade of five species, which is sister to *Pomaria*, or are part of another lineage containing the type species of both *Poincianella* and *Erythrostemon* (Fig. 3D). The Andean clade has recently been recognised as the new genus *Arquita*, based on a combination of morphological, ecological and geographical characters (Gagnon et al. 2015, Fig. 39I–O). In the other lineage, two robustly supported subclades are resolved, one including the type species of *Erythrostemon* (*Erythrostemon gilliesii*), and the other the type of *Poincianella* (*Poincianella mexicana*; Fig. 3D). While these two subclades could potentially be retained as distinct genera, the unresolved relationships of *Caesalpinia glandulosa* and *Caesalpinia placida* at the base of this Poincianella-Erythrostemon lineage in the current phylogeny (Fig. 3D) would entail recognizing two additional monospecific genera to account for these species. We prefer to treat this large Poincianella-Erythrostemon clade as a single genus which comprises a morphologically and ecologically coherent group of shrubs and small treelets in Neotropical seasonally dry tropical forests with a bicentric amphitropical distribution (Lewis 1998, Gagnon et al. 2013). Although there are currently more species under the name *Poincianella* Britton & Rose (1930), the older name *Erythrostemon* Klotzsch (1844) takes precedence. As such, *Erythrostemon* is here re-circumscribed to include *Poincianella* but excludes the subsets of *Poincianella* species now transferred to either *Cenostigma* or *Arquita*.

*Caesalpinia* s.s., as delimited by Lewis (2005), is also non-monophyletic and comprises three independent lineages. The most distinctive of these – the *Caesalpinia trothae* clade – clearly is not closely related to the remaining *Caesalpinia* s. s. species (Fig. 3B). This clade consists of African species found in dry forests and thickets from the Horn of Africa, across Tanzania, Botswana, Mozambique, and South Africa to Namibia. Species in this clade share a number of diagnostic morphological synapomorphies: they are all spiny, multi-stemmed shrubs with racemes of reddish-pink to whitish-pink flowers (Fig. 11J); have distinct pyriform pods, with large, rounded, oblique bases and an acute apex; bracts with an aristate tip; and leaflets with translucent dots on the lower surface. However, species delimitation needs to be re-examined. For example, Brenan (1963, 1967) remarked that the rostrate appendage on the calyx, which distinguishes *Caesalpinia rostrata*, is also found on some specimens of *Caesalpinia rubra*, bringing into question the distinction of these two species. Despite uncertainty about the number of species, this clade is phylogenetically, morphologically and geographically distinct, clearly meriting recognition as a new genus, here named *Gelrebia* after the Somali vernacular name for *Caesalpinia trothae*, which means camel trap and evidently alludes to the highly thorny and impenetrable habit of these plants.

The other two clades containing members of the former *Caesalpinia* s. s. lack obvious diagnostic morphological synapomorphies. Both clades include species of shrubs or small treelets that are eglandular and generally spiny (except for one species in each clade), and have explosively dehiscent pods with twisting valves. The type species of *Caesalpinia* s. s., *Caesalpinia brasiliensis*, is placed within a clade that includes a set of Caribbean species, most probably pollinated by bats (Koch et al. 2004), the Central American / Mexican *Caesalpinia pulcherrima*, pollinated by butterflies (Fig. 11F), the northern Andean *Caesalpinia cassioides* with red, laterally-compressed, tubular corollas, likely pollinated by birds (Fig. 11C), and *Caesalpinia nipensis*, endemic to the Sierra de Nipe in Cuba, which has a flower morphology and a yellow corolla suggestive of bee pollination (Fig. 11B). As recircumscribed here, a reduced *Caesalpinia* s. s. is now restricted to the Neotropics with no species now ascribed to this genus in Africa or Asia. The other group, the *Caesalpinia erianthera* clade (Fig. 3A), contains only yellow-flowered species, but these occur across a strikingly disjunct geographic range in Madasgascar (*Caesalpinia madagascariensis*, Fig. 11I), Ethiopia, Somalia and the Arabian Peninsula (*Caesalpinia erianthera*), South America (*Caesalpinia stuckertii*), Mexico (*Caesalpinia sessilifolia*), and the Caribbean (*Caesalpinia buchii*, *Caesalpinia pauciflora* (Fig. 11G, H) and *Caesalpinia rosei*). The *Caesalpinia erianthera* clade is morphologically distinct from its sister clade, the combined *Tara* + *Coulteria* clade. This latter clade includes species that are characterised by flowers having a distinctive lower sepal with a cucullate-pectinate margin (although the pectinate margin is absent in *Caesalpinia vesicaria*, and in *Caesalpinia cacalaco* the margin is only obscurely pectinate), and pods which are thick and indehiscent (*Tara*), or thin, chartaceous and indehiscent to tardily and passively dehiscent (*Coulteria*). Species from the *Caesalpinia erianthera* clade lack the cucullate-pectinate lower sepal margin and have pods that are explosively dehiscent, with twisting valves. Given the distant phylogenetic placement of the *Caesalpinia erianthera* clade from both *Gelrebia* and the recircumscribed *Caesalpinia* s. s., and its morphological distinctiveness from its sister group, it is clear that the *Caesalpinia erianthera* clade should also be recognised as a distinct genus. Within this clade, *Caesalpinia madagascariensis*, endemic to Madagascar, was formerly placed in the monospecific genus *Denisophytum*, here reinstated with an emended circumscription that includes all species of the *Caesalpinia erianthera* clade.

The majority of the rest of the currently unclassified Old World species fall into two main clades, the *Caesalpinia decapetala* clade and a clade that groups the monospecific genus *Moullava*, *Caesalpinia welwitschiana* and two species of Caesalpinia section Cinclidocarpus, which Gagnon et al. (2013) suggested to be closely related to *Moullava*. The species in these two Old World clades consist of lianas and scrambling shrubs, but are distinguished from the other liana taxa in the Caesalpinia group (which are concentrated in clade C, see Figs 2 and 3B) by their distinctive pods. In the *Caesalpinia decapetala* clade, the pods are oblong and somewhat laterally compressed, dehiscent along the dorsal suture, and slightly enlarged and truncate towards the apex. In the second clade, all four species have similar rounded, sub-torulose indehiscent pods, with thickened margins, and an exocarp and endocarp that are strongly adnate when dried. It is apparent that both clades merit recognition at the generic level. Based on the preliminary results of Gagnon et al. (2013), Molinari et al. (2016) reinstated the genus *Biancaea* Todaro (1860) for the *Caesalpinia decapetala* clade and provided new combinations for three species within the genus. Here we transfer an additional species of *Caesalpinia* to *Biancaea* and emend the description of the genus, which was not included in the treatment of Molinari et al. (2016). We also emend the description of *Moullava* to include three additional species in that genus (Fig. 3B) (see Taxonomic treatment for details).

### Monospecific genera

With near-complete taxon sampling and robust support across the phylogeny, it is now clear that the three species, *Caesalpinia mimosoides*, *Caesalpinia pearsonii* and *Caesalpinia echinata*, do not nest within any of the well resolved clades of the Caesalpinia group even though all six loci were sequenced for these species (except for ITS in *Caesalpinia mimosoides*). The taxonomic placements of these taxa have been problematic in the past, and each species is morphologically unique within the Caesalpinia group, especially with respect to pod morphology. To incorporate these unusual taxa in our generic classification, we propose three new monospecific genera, *Hultholia*, *Hererolandia* and *Paubrasilia*, respectively.

*Caesalpinia mimosoides* (Figs 17, 18) is a liana found in India, Bangladesh, Thailand, Vietnam, Laos, Myanmar and South-West China. It is morphologically distinct from all other liana species in the Caesalpinia group, because the stem, calyx and fruits are covered in glandular dots, and the pods are falcate, chartaceous and inflated. The robust, needle-like trichomes in *Caesalpinia mimosoides*, which are present on the stem, inflorescence rachis and pedicels, are also distinctive, and quite different from the more robust and strongly recurved prickles found on stems (and sometimes sparsely at the base of the inflorescences) of other Asian species of the Caesalpinia group. We propose the new generic name *Hultholia*, to honour the Cambodian taxonomist Dr. Salvamony Hul Thol (see Taxonomic treatment).

The second unplaced taxon, *Caesalpinia pearsonii*, differs from the rest of *Caesalpinia* s. l. primarily by its unusual flattened, circular or semi-circular one-seeded pods, covered in patent red trichomes up to 6 mm long (Fig. 5D). The precise relationships of this rarely collected species, endemic to Namibia, remain uncertain and weakly supported. Our analyses provide only weak support for a sister group relationship to the *Lophocarpinia* + *Haematoxylum* clade (Fig. 2), and in most analyses *Caesalpinia pearsonii* remains unresolved (Fig. 3A). *Caesalpinia pearsonii* differs from *Lophocarpinia* and *Haematoxylum* in having pinnate leaves arranged in fascicles on short brachyblasts, as opposed to the alternate pinnate or bipinnate leaves typical of these latter two genera. In addition, the secondary leaflet venation in *Caesalpinia pearsonii* is not visible, whereas in *Haematoxylum* the secondary veins are ascending, and form a sharp angle with the primary vein. Furthermore, armature among these genera differs, with curved and deflexed prickles on the stems and inflorescence rachis in *Caesalpinia pearsonii*, straight spinescent shoots in *Haematoxylum*, and straight, conical spines scattered along the branches in *Lophocarpinia*, which also has distinctively modified lateral, short, spine-tipped branchlets (Fig. 5H). Given the apparently isolated phylogenetic position of this taxon and its morphological distinctiveness, we recognise this species as a new genus, *Hererolandia*, a name referring to the type locality of *Hererolandia pearsonii*, which Bolus originally described as coming from “Hereroland” in Namibia, and also chosen to honour the Herero people of that country.

The third unplaced taxon, *Caesalpinia echinata*, also has several unusual morphological features. The pods of *Caesalpinia echinata* combine characteristics of *Guilandina* and *Caesalpinia* s. s. The patent, sub-woody bristles on the pod valves (Fig. 9B) are reminiscent of *Guilandina* pods (Fig. 20D and E), but the fruit is laterally compressed with lunate-falcate valves that twist after dehiscence and the seeds are flattened, as in many species of *Caesalpinia* s. s. In contrast to *Caesalpinia* s. s. and *Guilandina*, *Caesalpinia echinata* has reddish heartwood (Fig. 9F) which is a source of red dye (also found in *Caesalpinia sappan* in the *Caesalpinia decapetala* clade and in *Haematoxylum*). *Caesalpinia echinata* forms a medium-sized to large tree (Fig. 9E) with unusual upcurved prickles arising from woody protuberances on the trunk and branches (Fig. 9C). In our analyses, multiple accessions of *Caesalpinia echinata* form a clade in the ITS and *ycf6-psbM* gene trees and in the combined analysis (Fig. 3A), but in the other plastid gene trees there is no resolution amongst these accessions, suggesting lack of time for coalescence sensu Pennington and Lavin (2016) (Suppl. material 1). *Caesalpinia echinata* populations along the Atlantic coast of Brazil have been shown to be strongly differentiated genetically (Cardoso et al. 1998, 2005, Lira et al. 2003) and morphologically variable (Lewis 1998, De Lima et al. 2002). Denser sampling and detailed phylogeographical analyses are needed to assess whether these morphotypes represent a continuum or a set of discrete entities worthy of taxonomic recognition. Regardless, we consider that *Caesalpinia echinata* should be recognised as a distinct genus based on the available morphological and phylogenetic evidence. We propose the genus name *Paubrasilia*, based on the common name pau-brasil and in reference to the fact that *Paubrasilia* is the national tree of Brazil with a long and important association with the country.

### Unresolved generic relationships

Three areas of the phylogeny remain unclear and warrant greater sampling before making further adjustments to the generic classification. We hypothesise, based on morphology and preliminary phylogenetic results, that nine species from mainland Asia will form a well-supported clade with *Caesalpinia crista* (previously referred to as the *Caesalpinia nuga* clade; Gagnon et al. 2013), which is sister to *Pterolobium* and which also remains sparsely sampled (Fig. 3B). However, only two of these nine species, *Caesalpinia crista* and *Caesalpinia vernalis* (the latter not included in the combined analysis due to missing data, but placed in this clade in the *rps*16 gene tree in Gagnon et al. (2013)), have been sampled so far. If this putative *Caesalpinia crista* clade is indeed supported as monophyletic with greater taxon sampling, the oldest available generic name for the clade would be *Ticanto* Adans. It is notable that two of the species from mainland China (*Caesalpinia caesia* and *Caesalpinia sinense*) sometimes have a small wing on the fruit suggesting a fruit intermediate between the typical samara of *Pterolobium* and the wingless pods of species of the *Ticanto* clade. This morphological variation highlights the need for thorough sampling and detailed study to arrive at a better understanding of generic delimitation of this group (for more details see Clark 2016).

The other questionable taxa are the monospecific genera *Lophocarpinia* and *Stenodrepanum*, both of which could potentially be sunk into other genera. However, because only *trnL-trnF* and *matK-3’trnK*, the two least informative markers in our study, were sequenced for these two genera, their phylogenetic placements remain weakly or moderately supported. As found by Nores et al. (2012), *Lophocarpinia* is moderately supported as sister to *Haematoxylum* (Figs 2 and 3A, clade A). Burkart (1944, 1952) proposed that *Lophocarpinia* could be synonymised under *Haematoxylum* due to the strikingly similar vegetative morphology of the two genera, and despite the very distinctive lomentaceous and coarsely serrate-margined winged fruits of *Lophocarpinia* (Figs 5I and 6). Similarly, *Stenodrepanum* and *Hoffmannseggia* are weakly supported as sister taxa, and are distinguished morphologically only by their fruits which are cylindrical and torulose in *Stenodrepanum* and flattened in *Hoffmannseggia* (Fig. 34 F, H and K). Although these two generic pairs are differentiated on fruit characters alone, we refrain from proposing any taxonomic changes until additional sequence data can be obtained.

### Morphological variation in the Caesalpinia group

The Caesalpinia group has long been considered a morphologically heterogeneous group, in which morphological homoplasy and convergence have plagued previous attempts to provide a satisfactory generic system (see Lewis and Schrire 1995, Lewis 1998, Gagnon et al. 2013). As circumscribed here, the Caesalpinia group includes 27 robustly supported major lineages (26 of which are formally recognised here as genera). Although there are no unique diagnostic morphological synapomorphies for the clade as a whole, the Caesalpinia group can be recognised by a combination of features, including the presence of glandular trichomes, prickles and spines, bilaterally symmetrical flowers with a somewhat modified lower sepal, and free stamens crowded around the pistil; flowers vary greatly and can be strongly modified depending on pollination system, and fruits across the clade are extremely diverse reflecting a striking variation in seed dispersal strategies. Our new molecular phylogenies (Figs 2, 3) suggest that a number of leaf, armature and fruit characteristics can be used to distinguish genera and delimit the major clades, being exclusive, with minor exceptions, to particular clades. For example, bipinnate leaves with a terminal pinna occur almost exclusively in species of clade II, whereas almost all the species having bipinnate leaves without a terminal pinna are members of clade I. Similarly, clade II contains only species that lack thorns, spines or prickles, and almost all species that lack idioblasts in their leaflets (the latter are also absent in *Caesalpinia mimosoides* in clade I (Lersten and Curtis 1996) and in *Haematoxylum*), and almost all species in clade II are characterised by the presence of multi-cellular glandular structures on the stems, leaves and inflorescences (although *Haematoxylum dinteri*, *Caesalpinia mimosoides*, and members of *Coulteria* in clade I also have glandular structures on the margin of the pectinate lower cucullate sepal). In contrast, clade I contains all the species that are armed with spines and prickles along the branches (although *Coulteria*, *Caesalpinia madagascariensis* and *Caesalpinia nipensis* lack thorns, spines or prickles), and which have idioblasts in the lamina of their leaflets. The nearly mutually exclusive distribution of external glands vs. spines+idioblasts gives some support to the idea that these structures constitute alternative plant defense strategies against herbivory (Lersten and Curtis 1994, 1996), even though the role and function of idioblasts and secretory glands in the Caesalpinia group have never been studied in detail.

At the generic level, fruits are highly variable and taxonomically more useful than flowers. Several of the genera we recognise here can be differentiated based on fruit characteristics. For example, the pods of *Balsamocarpon*, *Cenostigma*, *Guilandina*, *Haematoxylum*, *Hererolandia*, *Hultholia*, *Libidibia*, *Lophocarpinia*, *Moullava*, *Mezoneuron*, *Paubrasilia*, *Pterolobium* and *Zuccagnia* are all distinctive and provide useful diagnostic synapomorphies for these genera (Figs 5, 9, 14, 18, 20, 24, 30, 34). In contrast, only a few floral synapomorphies are diagnostic at the generic level: *Guilandina* species have sepals that are valvate in bud; in the *Balsamocarpon*, *Zuccagnia*, and *Hoffmannseggia* clade, sepals are persistent until fruiting (Fig. 34), except in *Stenodrepanum* (Fig. 34); and in *Pomaria* species, the androecium and gynoecium are cupped in the lower cucullate sepal (Fig. 39A–C, F). In general, however, floral morphology within clades is highly variable reflecting differences in pollination syndromes, including examples of melittophily, chiropterophily, psychophily, phalaenophily and ornithophily, sometimes occurring among closely related congeneric species (e.g. *Caesalpinia* s. s., as emended here, and *Erythrostemon* – see above and Figs 11 and 42). These repeated floral morphologies across disparate members of the Caesalpinia group suggest convergent evolution of similar pollination modes in multiple clades across the group.

## Taxonomy

Here we present a comprehensive phylogenetically-based and significantly revised generic classification of the Caesalpinia group recognizing 26 genera, including re-instatement of two previously described genera, re-circumscription of eight genera and description of four new genera. A 27^th^ genus (*Ticanto*) is provisionally indicated, but not formally reinstated. A key to the identification of genera, full generic descriptions, and illustrations of all genera are presented. In addition, we provide new combinations where necessary and where we are confident about species affinities and taxonomy (*Biancaea*, *Cenostigma*, *Erythrostemon*, *Hererolandia*, *Hultholia*, *Libidibia*, *Moullava*, *Paubrasilia*) and/or lists of accepted species names (in bold) associated with each genus, as well as references to recently published species-level taxonomic accounts. For the genera *Guilandina*, *Coulteria* and *Ticanto*, only a preliminary list of species names (not bold) is indicated, with no nomenclatural combinations provided. These genera remain poorly understood taxonomically and work is currently ongoing in *Coulteria* to clarify and delimit species (Sotuyo et al., submitted).

### Key to the genera of the Caesalpinia group

Genus 27 *Ticanto* is provisionally indicated, pending further studies to establish the status of the genus

**Table:** 

1	Leaves pinnate	**2**
–	Leaves bipinnate	**10**
2	Armed shrubs or trees, with prickles scattered along the branches, or in pairs below the stipules, or plant with short branches modified into persistent thorns	**3**
–	Unarmed shrubs or trees	**6**
3	Sepals persistent in fruit; fruit a cylindrical pod covered with resinous hairs; pairs of needle-like prickles inserted below the stipules and leaf petiole; endemic to northern Chile, from the Coquibo and La Serena valleys	**20. *Balsamocarpon***
–	Sepals caducous; fruit a flattened and non-resinous pod; widely distributed across Central America, Mexico, the Caribbean, South America and Namibia	**4**
4	Fruit a lomentum, with 4 coarsely serrate wings, breaking up into one-seeded units (articles	**2. *Lophocarpinia***
–	Fruit unsegmented, without wings	**5**
5	Fruit sub-circular to sickle-shaped, tardily dehiscent along the sutures, finely pubescent and with robust patent trichomes	**1. *Hererolandia***
–	Fruit oblong to fusiform, dehiscent along the middle of the fruit valves or close to the fruit margin, but never along the sutures, lacking patent trichomes	**3. *Haematoxylum***
6	Sepals persistent; fruit a gall-like pod, covered with long bristles	**21. *Zuccagnia***
–	Sepals caducous; fruits ovoid to elliptic pods, not gall-like, glabrous or covered in a different type of indumentum	**7**
7	Fruit an elastically dehiscent pod, with valves twisting upon dehiscence, laterally-compressed and subligneous to woody, oblanceolate to oblong-elliptic	**8**
–	Fruit an indehiscent pod, thickened and fleshy, ovoid or elliptic	**9**
8	Fruit subligneous, lacking a crest; sepals valvate; restricted to Africa and Madagascar; stellate indumentum lacking	**17. *Stuhlmannia***
–	Fruit woody, with conspicuously thickened sutures, sometimes with a crest proximally on the adaxial side; sepals imbricate; restricted to the Neotopics; stellate indumentum often present	**18. *Cenostigma***
9	Fruit elliptic, somewhat thick and fleshy, bright red at maturity, rounded at apex and base, 1–2-seeded; leaflets with black, sessile glands on the under-surface; seeds compressed-turgid; sepals imbricate; endemic to Hispaniola and Puerto Rico	**19. *Libidibia monosperma***
–	Fruit ovoid, apex beaked; 1–4-seeded; leaflets with red glands on the lower surface; seeds ovoid; sepals valvate; endemic to NE Africa	**16. *Cordeauxia***
10	Leaves terminating in a pair of pinnae plus a single terminal pinna	**11**
–	Leaves terminating in a pair of pinnae	**18**
11	Plant armed; fruits oblong to fusiform, glabrous, dehiscing along the middle of the valves, or parallel to the margin	**3. *Haematoxylum***
–	Plant unarmed; fruits not dehiscing along the middle of the valves	**12**
12	Sepals persistent in fruit	**23. *Hoffmannseggia***
–	Sepals caducous in fruit	**13**
13	Pods cylindrical-torulose; central and western Argentina, in subtropical wooded grassland and scrub, especially on salt pans	**22. *Stenodrepanum***
–	Pods never cylindrical torulose	**14**
14	Stipules linear, persistent; androecium and gynoecium cupped in the lower cucullate sepal, lower lateral sepals forming a platform at right angles to the abaxial cucullate sepal; pods with simple trichomes, glandular-punctate trichomes, and plumose, dendritic and/or stellate trichomes	**25. *Pomaria***
–	Stipules caducous; androecium and gynoecium not cupped in the lower sepal, deflexed; lateral sepals not forming a platform; fruits glabrous or with simple and/or gland-tipped trichomes, the latter sometimes also dendritic or plumose	**15**
15	Fruits indehiscent; inflorescence a raceme or panicle, often corymbose; leaflets glabrescent and eglandular, or with glandular dots parallel to the midvein	**19. *Libidibia***
–	Fruits dehiscent, often with twisting valves; inflorescence a raceme or panicle, sometimes pyramidal in shape; leaflets glabrescent to densely pubescent, or with a stellate indumentum; leaflets eglandular, or with dark subepidermal glands, and/or with glandular dots sunken in the margins of the leaflets or parallel to the margin on the abaxial side	**16**
16	Leaflets alternate, or occasionally nearly opposite (rarely opposite), with dark subepidermal glands (best seen with a x10 hand lens); stellate indumentum sometimes present on foliage and inflorescence rachis; fruit subligneous to woody, with thickened sutures	**18. *Cenostigma***
–	Leaflets always opposite, without dark subepidermal glands; stellate indumentum never present on foliage or rachis; fruit coriaceous to subligneous, sutures not thickened	**17**
17	Shrubs or small to medium-sized trees varying from (0.5–) 1–12 (–20) meters tall, occasionally functionally herbaceous subshrubs, woody at the base; widespread across low-elevation seasonally dry tropical forests in Mexico, Central America, the Caribbean, and in Caatinga vegetation in Brazil, and in patches of dry forest, deserts, yungas-puna transition zones, and chaco-transition forests in Argentina, Bolivia, Chile and Paraguay; flowers yellow, red, pink or orange, sometimes laterally compressed; ovary eglandular or covered in gland-tipped trichomes, the hairs never dendritic	**26. *Erythrostemon***
–	Small to medium-sized, often decumbent, shrubs, 0.3–2.5 m tall; occurring at mid elevations in dry inter-Andean valleys, in Ecuador, Peru, Bolivia and Argentina; flowers yellow, sometimes all five petals streaked with red markings, never laterally compressed; ovary covered in gland-tipped trichomes, which are sometimes dendritic	**24. *Arquita***
18	Plants unarmed	**19**
–	Plants armed	**22**
19	Fruit thin, flat, oblong-elliptic to elliptic, membranaceous to papyraceous, indehiscent; margin of the lower cucullate sepal pectinate-glandular; flowers unisexual; leaflets eglandular	**8. *Coulteria***
–	Fruit an oblong-elliptic pod, elastically dehiscent with twisting valves; margin of the lower cucullate sepal entire; flowers bisexual; leaflets eglandular or with red glands	**20**
20	Flowers nearly actinomorphic; trees, up to 25 m tall; leaflets eglandular or with red glands; E Africa (Kenya and Tanzania), and N and NW Madagascar	**17. *Stuhlmannia***
–	Flowers clearly zygomorphic; shrubs or small trees, up to 5m tall; leaflets eglandular; Cuba or northern Madagascar (close to Antsiranana)	**21**
21	Fruits laterally compressed; anthers glabrous; endemic to Cuba (near Moa, in the Sierra de Nipe)	**5. *Caesalpinia nipensis***
–	Fruits inflated and hollow; anthers pubescent; endemic to the northern tip of Madagascar (Orangea peninsula, near Antsiranana)	**6. *Denisophytum madagascariense***
22	Trees or erect shrubs	**23**
–	Lianas or climbing or trailing shrubs	**27**
23	Fruits indehiscent, somewhat fleshy, turgid and coriaceous; lower cucullate sepal with a pectinate/fimbriate or entire margin	**7. *Tara***
–	Fruits dehiscent, with valves twisting upon dehiscence, laterally-compressed and subligneous to woody; lower cucullate sepal with an entire margin	**24**
24	Fruits armed with woody spines, stems with upturned thorns arising from woody protuberances; flowers yellow, the median petal with a conspicuous red blotch on the inner face	**4. *Paubrasilia***
–	Fruits unarmed, stems with straight to deflexed prickles; flowers yellow, white, pink, red or orange	**25**
25	Flowers pink-purple to whitish pink; bracts broadly ovate to suborbicular with an aristate apex; pyriform pods with rounded, oblique bases; sometimes translucent dots on leaflet lower surface	**9. *Gelrebia***
–	Flowers yellow, red, orange , green or white (horticultural variety sometimes pink); bracts lanceolate to linear with an acute to acuminate apex; pods oblong-elliptic, short-stipitate, with a cuneate base; leaflets eglandular	**26**
26	Flowers orange, red, green, white, rarely yellow or pink; Central America, Mexico, the Caribbean and the northern Andes (Peru to Colombia)	**5. *Caesalpinia***
–	Flowers yellow, sometimes with red markings on the standard (median petal); Somalia, Ethiopia, Argentina, Paraguay, Mexico, Florida and the Caribbean	**6. *Denisophytum***
27	Fruits with a wing, although this sometimes very narrow	**28**
–	Fruits without a wing	**31**
28	Fruit a samara (with a basal 1-seeded chamber and a prolonged upper suture that is broadly winged)	**14. *Pterolobium***
–	Fruit 1 or more seeded, with a longitudinal (often narrow) wing along the upper suture	**29**
29	Fruit with a wing 2 mm or more wide, chartaceous, coriaceous or ligneous; Africa, Madagascar and SE Asia across the Malay Peninsula and Archipelago to New Guinea, New Caledonia and Australia, one species endemic to Hawaii	**15. *Mezoneuron***
–	Fruit with a wing 2 mm wide or less; coriaceous or ligneous; southern (principally mainland) China, Myanmar (Burma), N Laos and N Vietnam	**30**
30	Fruit oblong-elliptic, terminating in a sharp beak; 4–9-seeded	**13. *Biancaea decapetala***
–	Fruit rhomboid-circular to sub-elliptic; 1 (rarely 2)–seeded	**27. ? *Ticanto* (*Caesalpinia caesia*)**
31	Glands on stems, leaf rachis, inflorescence, and fruits; needle-like trichomes on inflorescence rachis and pedicels	**10. *Hultholia***
–	Plants eglandular; stems with recurved prickles; pedicels and inflorescence peduncle with a few prickles near their bases	**32**
32	Fruit oblong to oblong-elliptic	**33**
–	Fruit broadly elliptic to circular	**34**
33	Fruit oblong, indehiscent, somewhat fleshy, sub-torulose, with thickened sutures, terminating in an acute apex, exocarp and endocarp strongly adnate; seeds sub-globular	**12. *Moullava***
–	Fruit oblong to oblong-elliptic, laterally compressed, dehiscent, coriaceous to subligneous, with a smooth, regular outer surface, base often much narrower than the truncate apex which terminates in a sharp beak, exocarp and endocarp separate easily; seeds flattened to ellipsoidal	**13. *Biancaea***
34	Flowers unisexual, segregated into female and male racemes; fruits usually covered in spinescent bristles; seeds globose, with parallel fracture lines concentric with the small apical hilum	**11. *Guilandina***
–	Flowers bisexual, in racemes; fruits always glabrous; seeds laterally compressed, smooth, without fracture lines	**27. ? *Ticanto***

### Taxonomic treatment of the genera of the Caesalpinia group

#### List of accepted genera

1. *Hererolandia* E. Gagnon & G. P. Lewis, **gen. nov.**

2. *Lophocarpinia* Burkart

3. *Haematoxylum* L.

4. *Paubrasilia* E. Gagnon, H. C. Lima & G. P. Lewis, **gen. nov.**

5. *Caesalpinia* L., descr. emended E. Gagnon & G. P. Lewis

6. *Denisophytum* R. Vig., descr. emended E. Gagnon & G. P. Lewis

7. *Tara* Molina, descr. emended E. Gagnon & G. P. Lewis

8. *Coulteria* Kunth, descr. emended E. Gagnon, Sotuyo, & G. P. Lewis

9. *Gelrebia* E. Gagnon & G. P. Lewis, **gen. nov.**

10. *Hultholia* E. Gagnon & G. P. Lewis, **gen. nov.**

11. *Guilandina* L.

12. *Moullava* Adans., descr. emended E. Gagnon & G. P. Lewis

13. *Biancaea* Tod., descr. emended E. Gagnon & G. P. Lewis

14. *Pterolobium* R. Br. ex Wight & Arn.

15. *Mezoneuron* Desf.

16. *Cordeauxia* Hemsl.

17. *Stuhlmannia* Taub.

18. *Cenostigma* Tul., descr. emended E. Gagnon & G. P. Lewis

19. *Libidibia* (DC.) Schltdl., descr. emended E. Gagnon & G. P. Lewis

20. *Balsamocarpon* Clos

21. *Zuccagnia* Cav.

22. *Stenodrepanum* Harms

23. *Hoffmannseggia* Cav.

24. *Arquita* E. Gagnon, G. P. Lewis & C. E. Hughes

25. *Pomaria* Cav.

26. *Erythrostemon* Klotzsch, descr. emended E. Gagnon & G. P. Lewis

?27. *Ticanto* Adans.

#### 1. Hererolandia

Taxon classificationPlantaeFabalesLeguminosae

E. Gagnon & G. P. Lewis gen. nov.

urn:lsid:ipni.org:names:77158009-1

Figs 4 , 5A–D

##### Diagnosis.

*Hererolandia* most closely resembles *Lophocarpinia*, but differs in having scattered curved, deflexed prickles on shoots (vs. scattered straight, conical spines, as well as modified, short, lateral, spinescent branchlets), pinnate leaves with (4–) 5–7 (–9) pairs of leaflets, arranged in fascicles (vs. alternate, pinnate leaves with 2–3 pairs of leaflets), and leaflets elliptic to oblong-elliptic (vs. leaflets obovate or elliptic-orbicular). The most distinctive feature of *Hererolandia* is the thinly woody, laterally compressed, almost circular to strongly sickle-shaped, usually 1-seeded fruit, covered in robust trichomes up to 6 mm long (vs. a segmented, falcate, lomentaceous fruit, with 4 coarsely serrate wings, breaking up into 1-seeded units).

##### Type.

*Hererolandia pearsonii* (L. Bolus) E. Gagnon & G. P. Lewis ≡ *Caesalpinia pearsonii* L. Bolus

##### Description.

A multi-stemmed shrub to 2 m, but usually less than 1 m tall, armed with curved, deflexed, 7 mm long prickles scattered along the branches; bark white or brown; stems terete and slightly sinuous, with a fine silvery indumentum on the young twigs, older stems glabrescent. Stipules not seen. Leaves pinnate, 7–17 mm long, subsessile, borne in fascicles on short woody brachyblasts that are usually subtended by a pair of tiny (sometimes obscure) prickles; leaflets opposite, (4–) 5–7 (–9) pairs per pinna, eglandular, covered in a fine silvery pubescence, 5–6.5 × 2.5–3 mm, elliptic to oblong-elliptic, apex obtuse, with an acuminate tip, main vein prominent, secondary venation not visible. Inflorescence a short raceme of bisexual flowers, about 5 cm long, usually borne on brachyblasts, covered in a fine silvery pubescence, with prickles along the inflorescence rachis; bracts about 2–3 × 1.5 mm, ovate, apex acute, caducous. Flowers zygomorphic; calyx with a short hypanthium, and 5 free sepals, c. 3–5 mm long, finely white pubescent, with the lower sepal cucullate and covering the other 4 sepals in bud, all sepals caducous, but hypanthium persistent as a ring around the stipe of the fruit; petals 5, yellow, free, c. 6–9 mm long, obovate; stamens 10, free, up to 10 mm long, eglandular, pubescent on the lower half; ovary pubescent, stigma a fringed and slightly indented chamber. Fruit a thinly woody, laterally compressed, almost circular to strongly sickle-shaped pod, c. 2–2.3 × 1–1.5 cm, dehiscing along the sutures, finely pubescent and covered in robust trichomes up to 6 mm long, usually 1-seeded. Seeds laterally compressed, about 6–8 mm long.

##### Geographic distribution.

A monospecific genus endemic to Namibia, on the Great Escarpment.

##### Habitat.

Semi-desert and desert areas, on stony, sandy soils.

##### Etymology.

Semiarid Hereroland, a region of eastern Namibia, is the type locality of *Hererolandia pearsonii*. The Herero people who inhabit this region are nomadic cattle herders and it is they and their region that are honoured in the name proposed for this monospecific genus, endemic to this restricted area of Namibia.

##### References.

Bolus (1920); Roux (2003); Curtis and Mannheimer (2005: 227).

**Figure 4.**
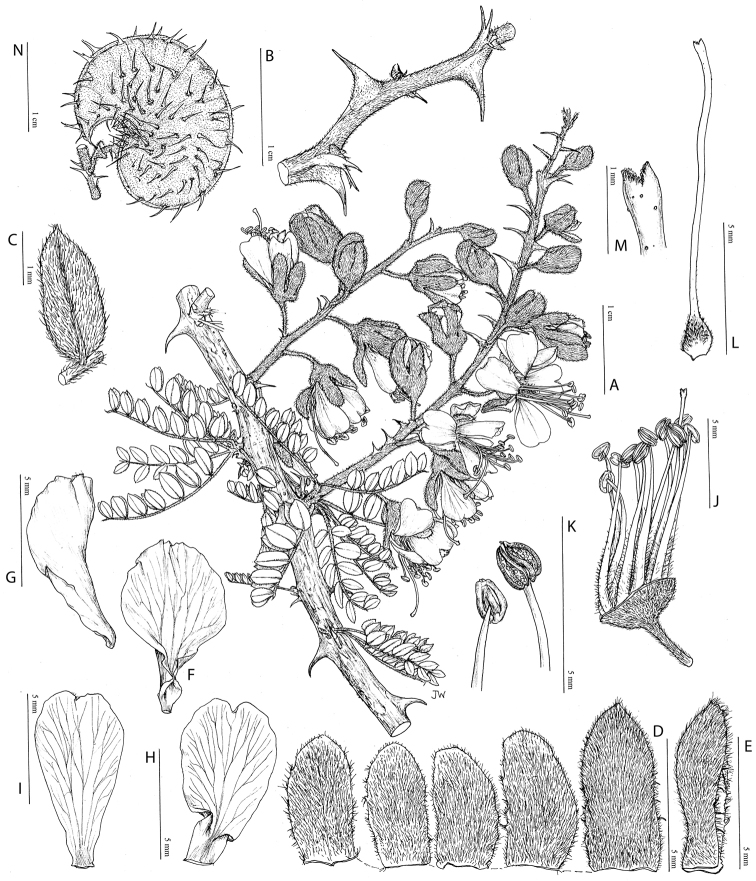
*Hererolandia pearsonii* (L. Bolus) E. Gagnon & G. P. Lewis. **A** foliage and inflorescences **B** stem armature detail **C** leaflet lower surface **D** calyx lobes outer surface **E** lower cucullate calyx lobe side view **F** median petal inner surface **G** median petal side view **H** upper lateral petal inner surface **I** lower lateral petal inner surface **J** stamens and part of gynoecium, with calyx lobes removed **K** anthers dorsal and ventral views **L** gynoecium **M** stigma detail, **N** fruit. **A, C–M** from *Müller* 1006, **B, N** from *Geiss et al.* 5156. Drawn by Juliet Williamson.

**Figure 5.**
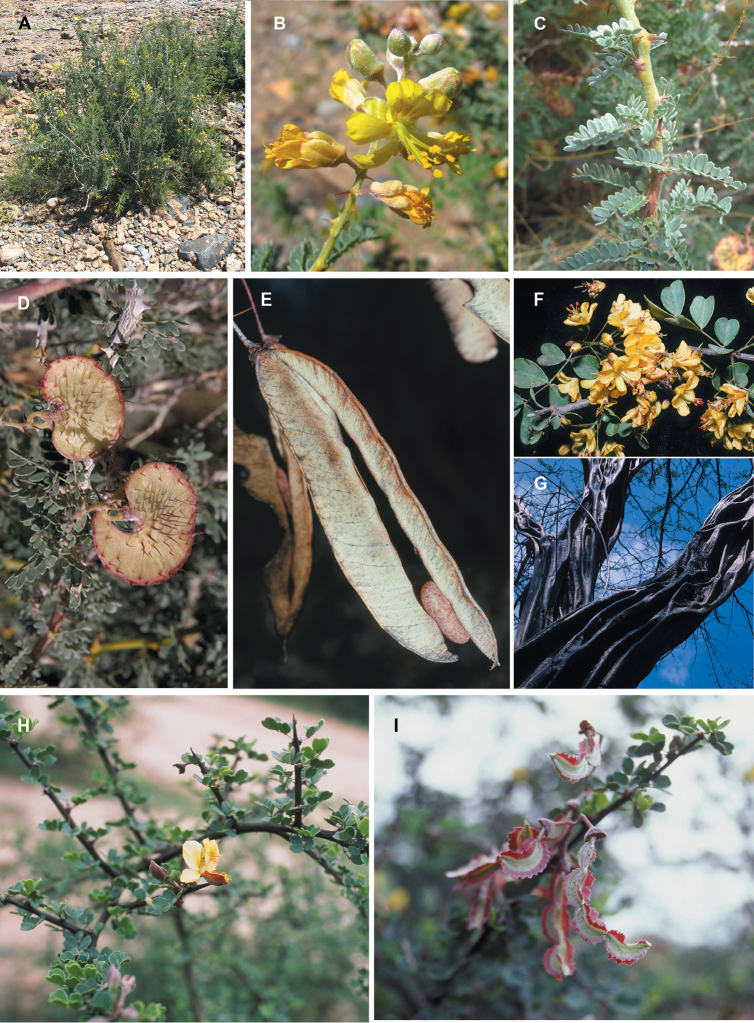
*Hererolandia pearsonii* (L. Bolus) E. Gagnon & G. P. Lewis. **A** shrubby habit **B** inflorescence **C** branch showing prickles and leaves **D** fruits (A. A. Dreyer, Sesriem Canyon, Namibia, *unvouchered*). *Haematoxylum brasiletto* H. Karst. **E** mature fruit dehiscing along the mid-valve (C. E. Hughes, Mexico, *unvouchered*) **F** inflorescences and leaves (G. P. Lewis, Mexico, *Lewis 2057* (K)) **G** distinctively fluted trunks (C. E. Hughes, Oaxaca, Mexico, *Hughes 1947* (FHO)) *Lophocarpinia aculeatifolia* (Burkart) Burkart **H** shrub with flowers, armed with straight conical spines **I** fruits (R. H. Fortunato, Paraguay, *Fortunato 8650* (BAB)).

#### 1.1 Hererolandia pearsonii

Taxon classificationPlantaeFabalesLeguminosae

(L. Bolus) E. Gagnon & G. P. Lewis comb. nov.

urn:lsid:ipni.org:names:77158011-1

##### Basionym.

*Caesalpinia pearsonii* L. Bolus, Annals of the Bolus Herbarium 3: 4. 1920.

##### Type.

NAMIBIA, Ababes, breccia banks of Tsondab River below farm, 29 Dec 1915, *Pearson 9162* (holotype: BOL; isotypes: K!, GRA, NBG, PRE).

#### 2. Lophocarpinia

Taxon classificationPlantaeFabalesLeguminosae

Burkart, Darwiniana 11: 256. 1957

Figs 5H–I , 6

##### Type.

*Lophocarpinia aculeatifolia* (Burkart) Burkart ≡ *Cenostigma aculeatifolium* Burkart.

##### Description.

Shrub 0.5 (– 3) m tall, armed with scattered straight, conical, 2–5 mm long spines on shoots; leaves and inflorescences crowded on brachyblasts; shoots glabrous, reddish, the lateral ones sometimes, spinescent. Stipules acuminate, caducous. Leaves alternate, paripinnate, 5–10 mm long; leaflets in 2 (– 3) pairs, obovate or elliptic-orbicular, 4–7 × 2–2.4 mm, finely pubescent, eglandular, with a pair of small prickles at the insertions of the leaflets. Inflorescences short, corymbiform, pubescent racemes, each with 3–6 bisexual flowers; bracts small, caducous. Flowers zygomorphic, 1–1.5 cm long; calyx with a turbinate, fleshy hypanthium, and 5 oblong, pubescent, caducous sepals, lower sepal cucullate and covering the other 4 sepals in bud, embracing the androecium and gynoecium at anthesis; petals 5, yellow to yellow-orange, free, the median petal differentiated from the rest by a fleshy claw and wavy blade margins, pubescent; stamens 10, free, filaments pubescent; ovary glabrous; stigma apical, concave. Fruit a lomentum, with 1–5 segments, falcate, with 4 coarsely serrate wings. Seeds ellipsoid to reniform, smooth.

##### Geographic distribution.

A monospecific genus restricted to Argentina and Paraguay (possibly also occurring in Mato Grosso do Sul, Brazil, pers. comm. H. C. de Lima).

##### Habitat.

Chaco woodland and seasonally dry tropical to subtropical forest.

##### Etymology.

From *lopho*- (Greek: combed or crested) and *carpos* (Greek: fruit), the fruit has 4 crested wings, the ending -inia signifies a close relationship with *Caesalpinia*.

##### References.

Burkart (1957); Ulibarri (2008); Nores et al. (2012).

**Figure 6.**
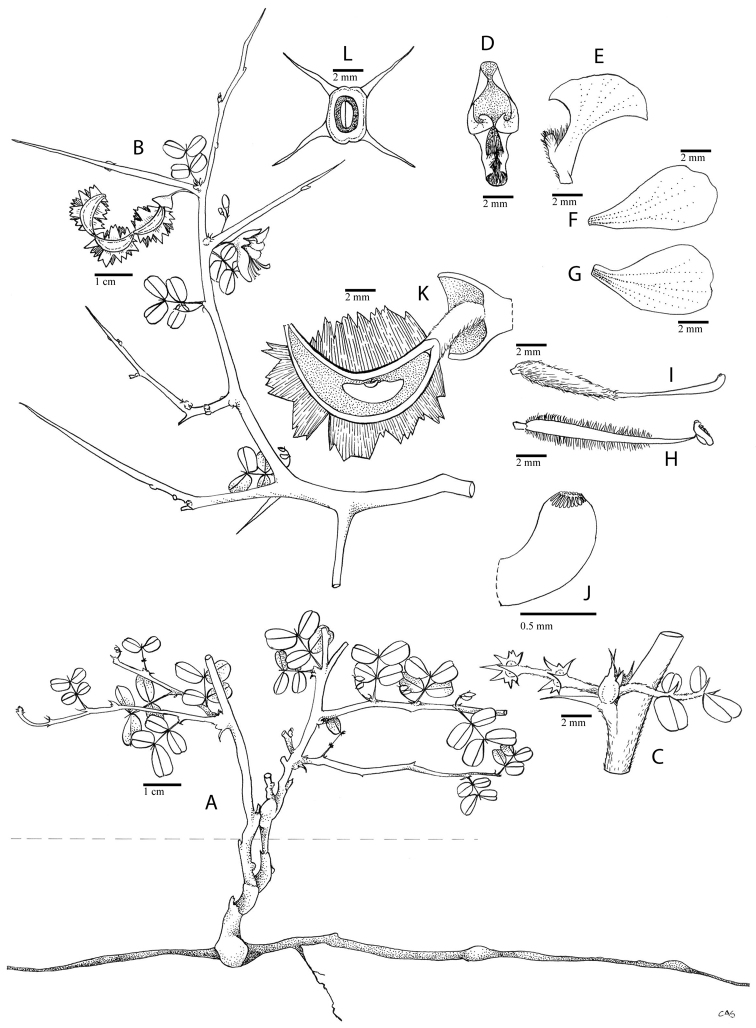
*Lophocarpinia aculeatifolia* (Burkart) Burkart. **A** habit **B** flowering and fruiting branch **C** detail of leaf attachment **D, E** median petal front and side views **F** upper lateral petal **G** lower lateral petal **H** stamen **I** gynoecium **J** stigma **K** fruit longitudinal section **L** fruit cross section. **A, B** from *Burkart* 20216 **C, K, L** after illustration by *Burkart* **D–J** from *Burkart* 20218. Drawn by Christi A. Sobel.

#### 2.1 Lophocarpinia aculeatifolia

Taxon classificationPlantaeFabalesLeguminosae

(Burkart) Burkart

#### 3. Haematoxylum

Taxon classificationPlantaeFabalesLeguminosae

L., Sp. Pl. 1: 384. 1753

Figs 5E–G , 7

Haematoxylon L., 1764, orthographic variant.
Cymbosepalum Baker, 1895.

##### Type.

*Haematoxylum campechianum* L.

##### Description.

Multi-stemmed shrubs to 3 m, to medium-sized trees, 3–15 m in height, armed with scattered straight conical spines, 0.5–1.5 cm long on shoots, and the short, lateral shoots spinescent; mature trees with conspicuously fluted trunks, shrubs often with ribbed branches; young stems reddish brown to grey, glabrous to pubescent, eglandular (or with stalked glands in *Haematoxylum dinteri*). Leaves alternate, pinnate or bipinnate (both can be present on the same individual in some species), glabrous to pubescent, eglandular, 1–10 cm long; pinnate leaves with 2–6 pairs of leaflets, 2.5–35 × 3–30 mm, glabrous to slightly pubescent, eglandular; bipinnate leaves with 1–3 pairs of pinnae plus a terminal pinna, each pinna with 2–5 (–6) pairs of leaflets, 5–11 × 2–4.5 mm; leaflets in opposite pairs, obcordate to obovate, apex emarginate to obtuse, base cuneate to attenuate (occasionally obtuse), short-petiolulate; primary vein centric, secondary veins ascending, and forming a sharp angle with the primary vein. Inflorescences terminal or axillary racemes or panicles of pedicellate flowers; rachis and pedicels unarmed, glabrous to pubescent, eglandular or glandular. Flowers bisexual, actinomorphic to zygomorphic; calyx comprising a hypanthium and 5 free sepals that are c. 6–7 mm long, glabrous to pubescent, eglandular or glandular, the lower sepal cucullate and slightly covering the other 4 in bud, sepals caducous, hypanthium persisting in fruit, forming a calyx ring; petals 5, yellow to pale yellow or white, free, imbricate, obovate to oblanceolate, 4–10 mm long; stamens 10, free, filaments pubescent, particularly on the lower half; ovary glabrous to pubescent. Fruit flattened, membranaceous to chartaceous, oblong to fusiform (occasionally falcate), apex rounded to obtuse, base acute, dehiscing along the middle of the valves, or near the margin of the fruit, but never along the sutures, 10–50 × 4–15 mm, 1–3-seeded. Seeds oblong to reniform, flattened, 6–12 × 3.8–5 mm.

##### Geographic distribution.

*Haematoxylum* comprises five species: two in Central America (Salvador to Costa Rica), Mexico, South America (Colombia and Venezuela) and the Caribbean (perhaps introduced), two endemic to Mexico and one in Southern Africa (Namibia).

##### Habitat.

Deserts, seasonally dry tropical semi-deciduous scrub and thorn scrub, sandy river beds and dry rocky hillsides. One species (*Haematoxylum campechianum*) is known to grow in frequently inundated marshy areas by rivers.

##### Etymology.

From *haemato*- (Greek: bloody) and *xylon* (Greek: wood), alluding to the blood-red heartwood of *Haematoxylum campechianum* L. which produces a brilliant red dye.

##### Notes.

There is a key to species by Durán and Sousa, in Novon 23(1): 31–36 (2014).

##### References.

Standley and Steyermark (1946); Ross (1977: 122–114); Roux (2003); Curtis and Mannheimer (2005: 215); Durán and Ramírez (2008); Barreto Valdés (2013); Durán and Sousa (2014).

**Figure 7.**
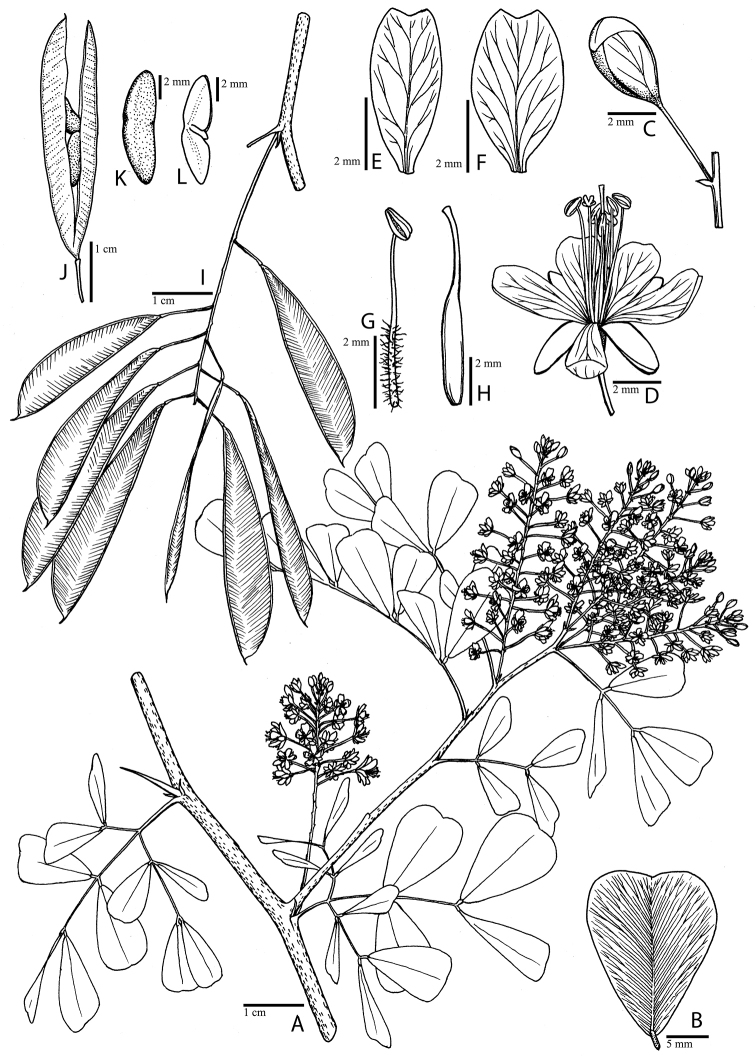
*Haematoxylum campechianum* L. **A** flowering branch **B** leaflet **C** flower bud **D** flower **E** median petal **F** lateral petal **G** stamen **H** gynoecium **I** infructescence **J** dehiscing fruit **K** seed **L** embryo. **A, B, D–H** from *Lorence* 2746 **C** from *Balfour* s.n. **I–L** from *Johnston* s.n. Drawn by Eleanor Catherine, originally published in *Flore des Mascareignes 80. Légumineuses*, page 6, plate 1.

#### 3.1 Haematoxylum brasiletto

Taxon classificationPlantaeFabalesLeguminosae

H. Karst.

#### 3.2 Haematoxylum calakmulense

Taxon classificationPlantaeFabalesLeguminosae

Cruz Durán & M. Sousa

#### 3.3 Haematoxylum campechianum

Taxon classificationPlantaeFabalesLeguminosae

L.

#### 3.4 Haematoxylum dinteri

Taxon classificationPlantaeFabalesLeguminosae

Harms

#### 3.5 Haematoxylum sousanum

Taxon classificationPlantaeFabalesLeguminosae

Cruz Durán & J. Jiménez Ram.

#### 4. Paubrasilia

Taxon classificationPlantaeFabalesLeguminosae

E. Gagnon, H. C. Lima & G. P. Lewis gen. nov.

urn:lsid:ipni.org:names:77158010-1

Figs 8 , 9

##### Diagnosis.

*Paubrasilia* is closely related to *Caesalpinia*, but differs in habit, forming medium-sized to large trees, 5–15+ m tall, armed with small to large upturned prickles, these usually arising from woody protuberances (vs. shrubs or small to medium sized trees, usually 1–6 m tall, unarmed or armed with curved deflexed prickles, either occurring in pairs at the base of leaves, or scattered on shoots, or both, and sometimes present at the base of trunk). *Paubrasilia* also differs from *Caesalpinia* by having alternate pinnae with consistently alternate leaflets (vs. opposite pinnae with opposite to alternate leaflets), the median petal with a blood red central blotch (vs. the median petal lacking a red central blotch) and a spiny, woody, finely pubescent, sub-lunate, 1–2-seeded pod (vs. an unarmed, glabrous, oblong-elliptic, generally 3–7-seeded pod, with a marcescent style forming an acute apex).

##### Type.

*Paubrasilia echinata* (Lam.) E. Gagnon, H.C. Lima & G. P. Lewis ≡ *Caesalpinia echinata* Lam.

##### Description.

Medium sized to large trees, 5–15+ m tall, armed with small to large upturned prickles, these usually arising from woody protuberances, 1–20 mm long (the prickles often sparse or lacking on more mature specimens and larger, older branches); bark chestnut brown to almost black with greyish pustular lenticels, flaking in large woody plates; heartwood red, with the trunk exuding a red sap when injured. Stipules lanceloate, acute to acuminate, caducous. Leaves bipinnate, ending with a pair of pinnae; petiole and rachis finely tomentose; pinnae alternate, the terminal pair opposite to subopposite, with (2–) 3–20 pairs of pinnae per leaf; leaflets alternate, with (2–) 3–19 (–21) leaflets per pinna (generally the number of leaflets is inversely proportional to their size), 0.9–5 × 0.5–3.6 cm (although some specimens have leaflets up to 12 cm long), leaflet blades coriaceous, broadly oblong to subrhombic, apex rounded, obtuse or emarginate, base asymmetric, eglandular, glabrous, midvein excentric, secondary veins brochidodromous. Inflorescence a terminal, or occasionally axillary, finely tomentose raceme or panicle, with c. 15–40 flowers; bracts broadly ovate-triangular, apex acute to acuminate, less than 1 mm long, pubescent, caducous. Flowers bisexual, zygomorphic; calyx a tomentose hypanthium with 5 sepals, that are c. 5–9 mm long, the lowest sepal cucullate, covering the other 4 in bud, all sepals caducous but the hypanthium persisting as a free ring around the pedicel as the pod matures; petals 5, free, bright yellow, the median petal with a blood-red blotch on the inner face, c. 11–15 × 4–10 mm, all petals eglandular, broadly-obovate to slightly spathulate, the petal claws pubescent; stamens 10, free, 7–9 mm long, eglandular, densely pubescent on lower half; ovary pubescent with small spines intermixed, stigma a subterminal fringed-chamber. Fruit a spiny, finely pubescent, sub-lunate, woody, 5.5–7.3 × 1.9–2.6 cm, elastically dehiscent pod with twisting valves, 1–2-seeded. Seeds laterally compressed, ovate-obovate.

##### Geographic distribution.

A monospecific genus endemic to Eastern Brazil, in the states of Pernambuco, Bahia, Espirito Santo and Rio de Janeiro. Widely cultivated in Brazil as an ornamental street or park tree, and sometimes in plantations.

##### Habitat.

Dry coastal cactus scrub often on rocky outcrops, inland in Mata Atlântica, and in tall restinga on well-drained sandy soil.

##### Etymology.

“Pau-brasil” is the national tree of Brazil, and has long been associated with the country. Its red sap was once used for dying cotton and cloth and its wood is much prized for the manufacture of high quality violin bows. Originally described as *Caesalpinia echinata* by Lamarck in 1785, it is appropriate that this phylogenetically isolated taxon should be placed in its own monospecific genus and a Latinization of its well-known and much used common name recognises the importance of the species to Brazil. For a detailed account of this iconic species refer to Pau-brasil by E. Bueno [et al.], São Paulo, Axis Mundi (2002).

##### References.

Lewis (1998: 152–158); Bueno (2002); Cardoso et al. (2005).

**Figure 8.**
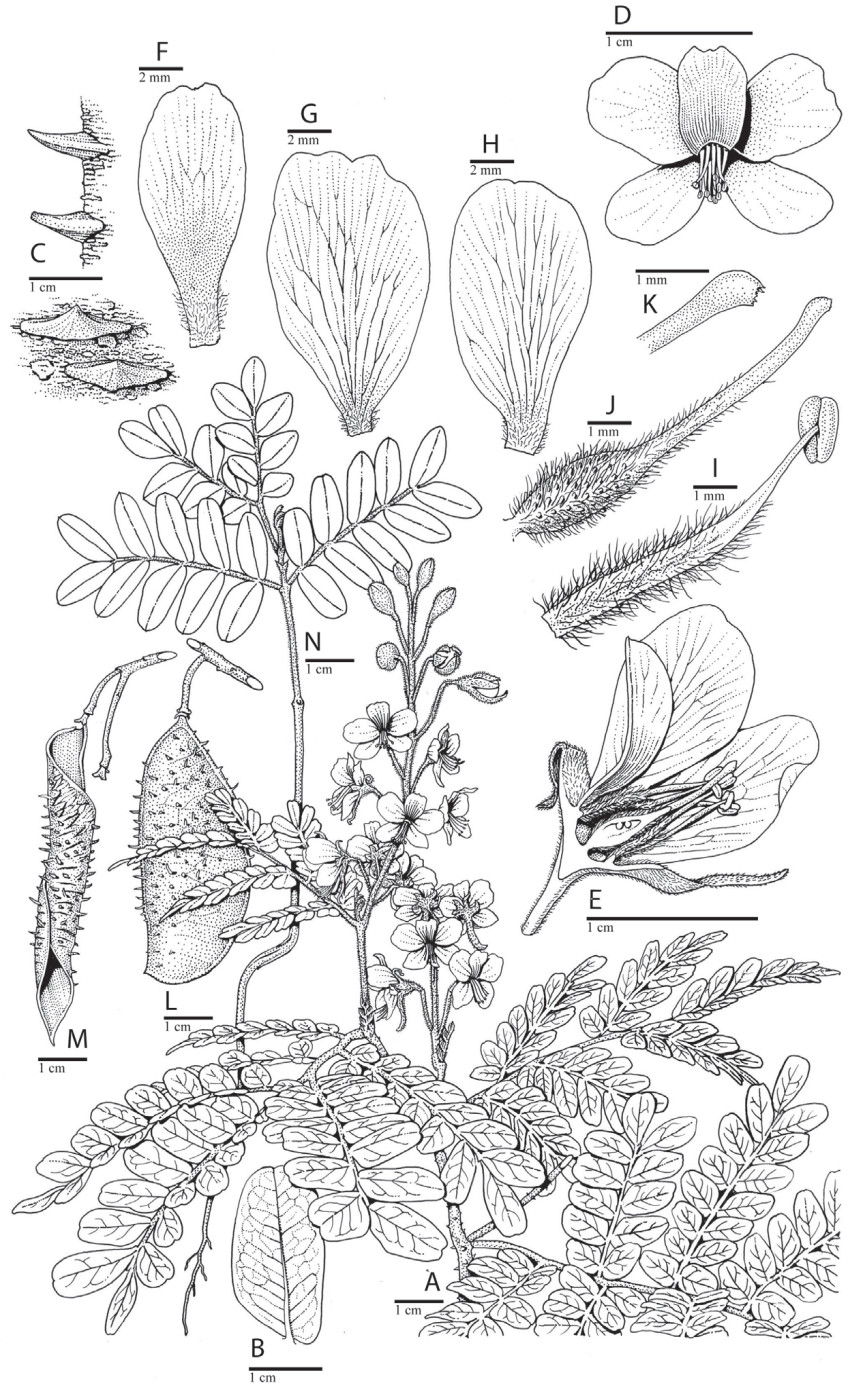
*Paubrasilia echinata* (Lam.) E. Gagnon, H. C. Lima & G. P. Lewis. **A** inflorescences and foliage **B** leaflet undersurface **C** bark armature (front and side views) **D** flower **E** flower l.s. **F** median petal **G** upper lateral petal **H** lower lateral petal **I** stamen **J** gynoecium **K** stigma **L** fruit **M** single valve of dehisced fruit **N** seedling. **A** from *Glaziou* 6839 **B, K** from *Angeli* 201 **C, M** from *Lewis et al.* 1634 **D** from *Lima et al.* 2705 **E–J** from *Ducke* 20623 **L** from *Mell* s.n., **N** from *Lewis et al.* 1624. Drawn by Tim Galloway.

**Figure 9.**
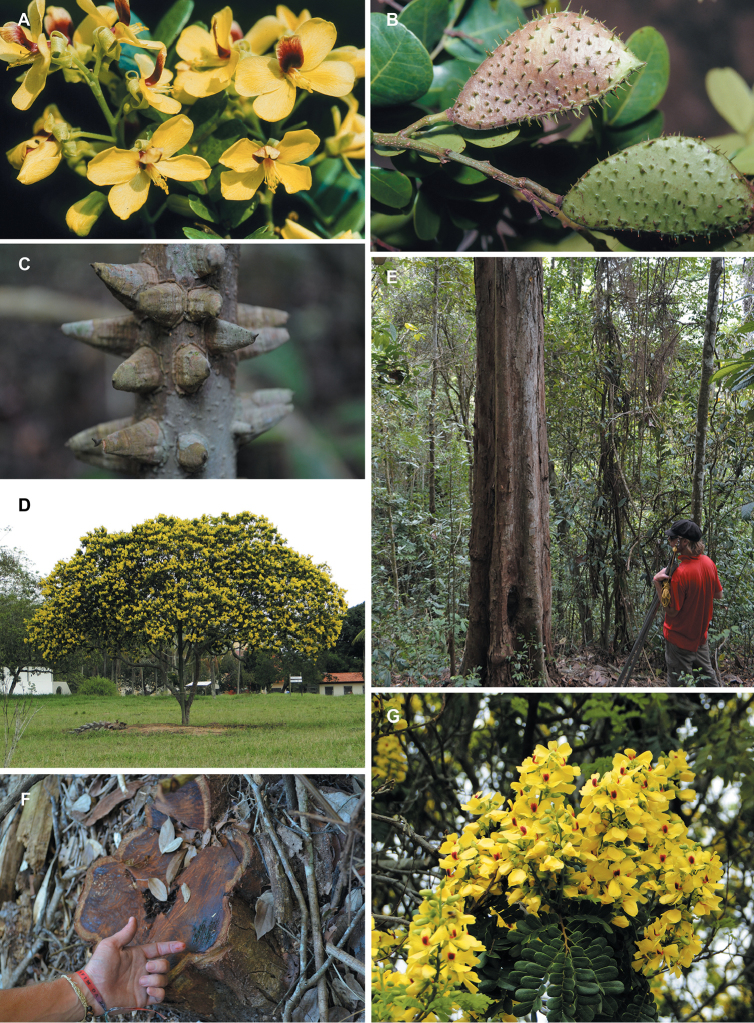
*Paubrasilia echinata* (Lam.) E. Gagnon, H. C. Lima & G. P. Lewis. **A** flowers (H.C. Lima, Brazil, *Lima et al. 2705* (RB)) **B** fruits (G. P. Lewis, Brazil, *unvouchered*) **C** prickles on woody protuberances on a young trunk (E. Gagnon, Bahia, Brazil, *Lima et al. 7909* (RB)) **D** habit (L. P. de Queiroz, Bahia, Brazil, *unvouchered*) **E** fluted trunk of a mature individual (E. Gagnon, Bahia, Brazil, *Lima et al. 7894* (RB) **F** cross section of the trunk, showing dark red heartwood (E. Gagnon, Espirito Santo, Brazil, *unvouchered*), **G** inflorescences (L. P. de Queiroz, Bahia, Brazil, *unvouchered*).

#### 4.1 Paubrasilia echinata

Taxon classificationPlantaeFabalesLeguminosae

(Lam.) E. Gagnon, H. C. Lima & G. P. Lewis comb. nov.

urn:lsid:ipni.org:names:77158012-1

Figs 8 , 9

##### Basionym.

*Caesalpinia echinata* Lam., Encycl. 1: 461. 1785. *Guilandina echinata* (Lam.) Spreng., Syst. Veg. 2: 327. 1825.

##### Type.

[BRAZIL] “In locis mari vicinis non apparet, sed tantum in mediterraneis silvis, unde magno labore ad littoralia vehitur” (Lectotype: [icon] “Ibirapitanga, sive Lignvm Rvbrvm” in Piso, De Indiae utriusque re naturali et medica: 164. 1658, designated here).

##### Epitype.

An epitype is to be selected in a subsequent paper focussing on the morphotypes of *Paubrasilia echinata* (De Lima et al., in prep.).

*Caesalpinia vesicaria* Vell., Fl. Flumin.: 172. 1829, Fl. Flumin. Icon. 4. t. 89. 1831. (“*vessicaria*”), non L. 1753. .

Type. [BRAZIL], “Habitat silvis maritimis usque ad Molendinum Sacchariferum dictum Itacurussá” (Lectotype: [icon] “*Cæsalpinia vessicaria*” in Velloso, Fl. Flumin. Icon. 4: t. 89. 1831).

*Caesalpinia obliqua* Vogel in Linnaea 11: 407. 1837.

Type: BRAZIL, *Sellow s.n.* (holotype ? B †; isotype P02142646!).

#### 5. Caesalpinia

Taxon classificationPlantaeFabalesLeguminosae

L. Sp. Pl. 1: 380 1753, descr. emended E. Gagnon & G. P. Lewis

Figs 10 , 11A–F

Poinciana L., in part (1753).
Brasilettia (DC.) Kuntze (1891), non sensu Britton & Rose (1930).

##### Diagnosis.

*Caesalpinia* resembles *Guilandina*, but differs in habit, comprising armed shrubs and small trees (vs. armed lianas and scrambling/trailing shrubs). It also differs in having racemes of bisexual flowers (vs. racemes of unisexual flowers), sepals imbricate in bud, with a pronounced lower cucullate sepal (vs. sepals valvate in bud), zygomorphic corollas variable in colour (yellow, white, red, orange, pink or green), with petals extending well beyond the sepals (vs. zygomorphic to sub-actinomorphic, yellow corollas, with petals barely extending beyond the sepals), coriaceous, oblong-elliptic to linear, laterally compressed, glabrous pods (vs. oblong-elliptic inflated pods, usually armed with 5–10 mm long spinescent bristles), and obovoid, laterally compressed seeds (vs. obovoid globular seeds).

##### Type.

*Caesalpinia brasiliensis* L.

##### Emended description.

Shrubs or small trees, usually 1–6 m tall, armed with curved deflexed prickles (except *Caesalpinia nipensis* which is unarmed), these either in pairs at the base of leaves, or scattered along the shoots (or both), or sometimes on woody protuberances at the base of trunks and stems; young shoots terete, glabrous and eglandular. Stipules not seen. Leaves alternate, bipinnate, c. 4–30 cm long, ending with a pair of pinnae, unarmed, or sometimes with a pair of prickles at the insertion of the pinnae on the leaf rachis, sometimes also at the insertions of the leaflets on the pinna rachis; pinnae opposite, in (1–) 2–6 pairs per leaf; leaflets alternate to opposite, in 3–13 pairs per pinna, short-petiolulate, blades suborbicular, obovate or elliptic, apex mucronate, rounded or emarginate, base cuneiform, rounded or oblique; main vein centric, secondary veins reticulate. Inflorescence a terminal or axillary raceme or panicle of pedicellate, bisexual flowers, c. 5–37 cm long, unarmed; bracts lanceolate or ovate, apex acute to acuminate, caducous. Flowers zygomorphic, c. 13–25 mm long; calyx comprising a hypanthium with 5 sepals, that are each c. 7–17 mm long, glabrous to occasionally finely puberulous, always eglandular, the lower sepal strongly cucullate and covering the other 4 sepals in bud, all sepals caducous, but hypanthium persistent as a free ring around the pedicel as the fruit matures; petals 5, variable in colour (yellow, white, red, orange, or green; certain horticultural varieties are also pink), the corolla also variable in shape (related to different pollination systems: bees, butterflies, birds and bats); stamens 10, free, c. 10–65 mm long, the filaments pubescent, eglandular; ovary glabrous and eglandular. Fruit a wingless, unarmed, coriaceous, glabrous, eglandular, oblong-elliptic, or linear pod, with a marcescent style forming an acute apex, c. 34–120 × 7–26 mm, explosively dehiscent, with twisting valves, 3–7-seeded. Seeds laterally compressed, obovate, up to 10 mm in diameter.

##### Geographic distribution.

*Caesalpinia*, as re-circumscribed here, is reduced to around nine species (a detailed taxonomic revision is needed to properly delimit species), and is now restricted to the Neotropics (apart from the pantropically cultivated *Caesalpinia pulcherrima*). All the Old World species previously included in *Caesalpinia* s.s. sensu Lewis (2005) are here transferred to other genera. One species (*Caesalpinia cassioides*) occurs in the northern Andes from Peru to Colombia, one (*Caesalpinia pulcherrima*) is likely native in Guatemala and the state of Sonora in Mexico), two occur in the Caribbean (one, *Caesalpinia nipensis*, is endemic to Cuba, the other widely distributed and possibly divisible into six separate species, all of which are listed below). *Caesalpinia pulcherrima* is a widely cultivated ornamental throughout the tropics. It includes red, orange, pink, and pure yellow-flowered forms and cultivated specimens are usually unarmed and lack bristles (unlike wild specimens which are armed and bristly).

##### Habitat.

Seasonally dry tropical forests, coastal thicket, bushland and thorn scrub, dry plains and riparian woodland, on soils derived from limestone or sandstone.

##### Etymology.

Named by Linnaeus for Andrea Cesalpino (1519–1603), Italian naturalist, botanical collector, systematist and philosopher, physician to Pope Clement VIII, professor of medicine and botany in Pisa and Rome.

##### References.

Britton and Rose (1930); Macbride (1943: 191, 194–195); Ulibarri (1996); Barreto Valdés (2013).

**Figure 10.**
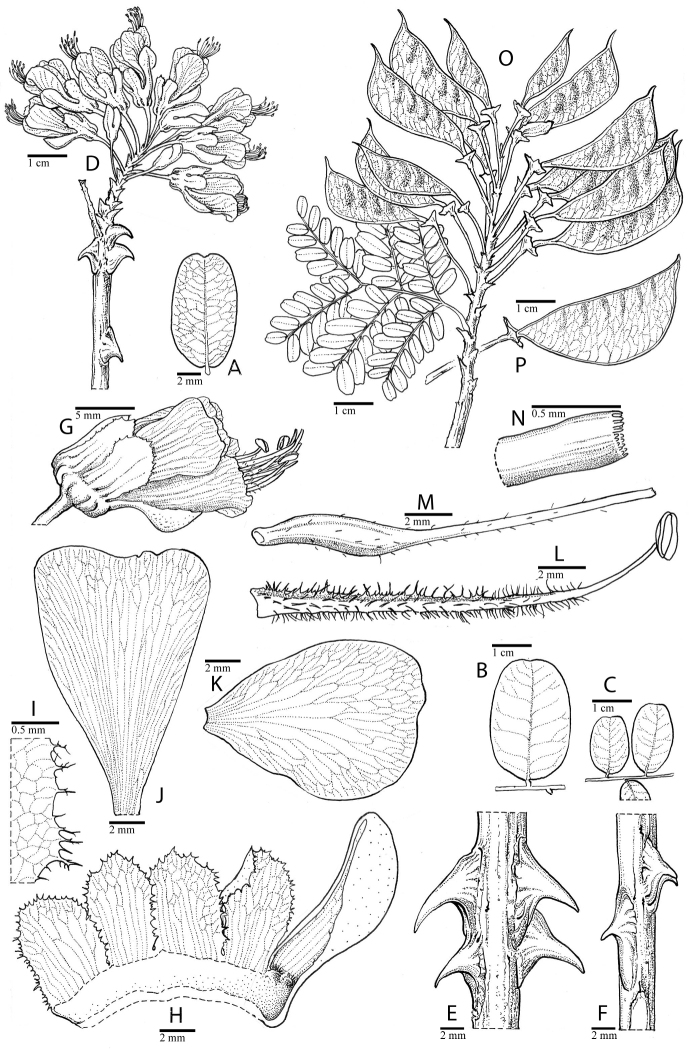
*Caesalpinia cassioides* Willd. **A** median leaflet **B, C** median leaflets (to show variation) **D** inflorescence **E, F** stem armature **G** flower **H** calyx opened out **I** calyx margin **J** median petal **K** upper lateral petal, **L** stamen **M** gynoecium **N** stigma **O** leaf and immature fruits **P** single immature fruit. **A, D, E, Q** from *Mayolo* 325 **B, C, R** from *Silverstone-Sopkin* 2004 **F** from *Sandeman* 4613 **G–P** from *Silverstone-Sopkin* 5139. Drawn by Sue Wickison.

**Figure 11.**
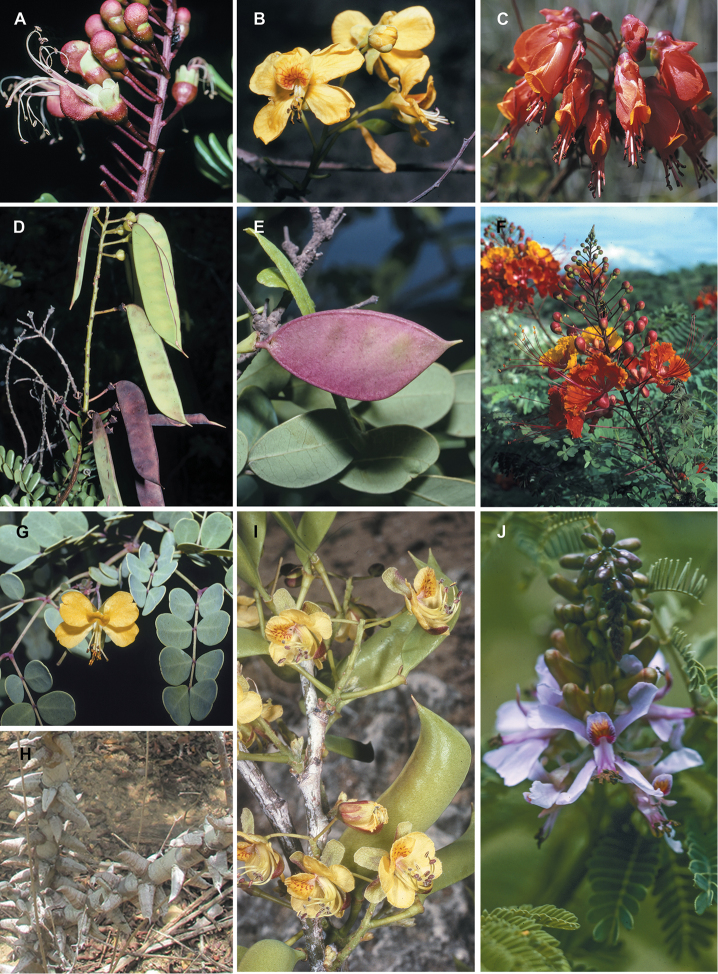
*Caesalpinia bahamensis* Lam. **A** inflorescence **D** fruits (G. P. Lewis, Cuba, *Lewis 1853* (K)). *Caesalpinia nipensis* Urb. **B** flowers **E** fruits (G. P. Lewis, Cuba, *Lewis 1838* (K)). *Caesalpinia cassioides* Willd. **C** inflorescence (C. E. Hughes, Ancash, Peru, *Hughes et al. 2228* (K)). *Caesalpinia pulcherrima* L. (Sw.) **F** inflorescence (C. E. Hughes, Sonora, Mexico, *unvouchered*); *Denisophytum pauciflorum* (Griseb.) E. Gagnon & G. P. Lewis **G** flower and leaves (G. P. Lewis, Cuba, *Lewis 1854* (K)) **H** branch with spine-tipped woody protuberances (B. Torke, Cuba, *Torke et al. 1424* (NY)). *Denisophytum madagascariense* R. Vig. **I** flowers and fruits (G. P. Lewis, Madagascar, *Lewis et al. 2158* (K)). *Gelrebia trothae* (Harms) E. Gagnon & G. P. Lewis **J** inflorescence (P.J. Cribb, Tanzania, *unvouchered*).

#### 5.1 Caesalpinia anacantha

Taxon classificationPlantaeFabalesLeguminosae

Urb.

#### 5.2 Caesalpinia bahamensis

Taxon classificationPlantaeFabalesLeguminosae

Lam.

#### 5.3 Caesalpinia barahonensis

Taxon classificationPlantaeFabalesLeguminosae

Urb.

#### 5.4 Caesalpinia brasiliensis

Taxon classificationPlantaeFabalesLeguminosae

L.

#### 5.5 Caesalpinia cassioides

Taxon classificationPlantaeFabalesLeguminosae

Willd.

#### 5.6 Caesalpinia monensis

Taxon classificationPlantaeFabalesLeguminosae

Britton

#### 5.7 Caesalpinia nipensis

Taxon classificationPlantaeFabalesLeguminosae

Urb.

#### 5.8 Caesalpinia pulcherrima

Taxon classificationPlantaeFabalesLeguminosae

(L.) Sw.

#### 5.9 Caesalpinia secundiflora

Taxon classificationPlantaeFabalesLeguminosae

Urb.

#### 6. Denisophytum

Taxon classificationPlantaeFabalesLeguminosae

R. Vig., Notul. Syst. (Paris) 13(4): 349. 1948, descr. emended E. Gagnon & G. P. Lewis

Figs 11G–I , 12

##### Diagnosis.

*Denisophytum* is closely related to *Tara* (Fig. 3), but differs in having flowers with a lower cucullate sepal with an entire margin (vs. a lower cucullate sepal with a pectinate margin), and dehiscent, coriaceous, laterally compressed pods (except for *Denisophytum madagascariense* which has inflated fruits) (vs. indehiscent, somewhat fleshy, coriaceous pods that are slightly turgid). Morphologically, species of *Denisophytum* are most likely to be confused with those of *Caesalpinia* s.s., but no reliable diagnostic characters have been found to differentiate these two genera. The corolla of *Denisophytum* species is consistently yellow and the flowers are bee pollinated, whereas *Caesalpinia* s.s. species display a wide range of flower colour (yellow, orange, red, green and white) and pollination syndromes (chiropterophily, ornitophily, psychophily and mellitophily).

##### Type.

*Denisophytum madagascariense* R. Vig.

##### Emended description.

Shrubs to small trees, 0.5–2 (–5) m tall, armed with straight or curved, deflexed prickles, scattered along shoots and also in pairs at the petiole base (except *Denisophytum madagascariense* which is unarmed); young twigs glabrous to pubescent, eglandular. Stipules either minute or foliaceous and conspicuous, caducous (persistent in *Denisophytum stuckertii*). Leaves alternate, bipinnate, ending with a pair of pinnae; petiole and rachis glabrous and eglandular, with membranous or spinulose stipels at the insertions of pinnae on the leaf rachis, occasionally also at the insertion of the leaflets on the pinnae; pinnae opposite, in 1–6 pairs per leaf; leaflets opposite, in 2–10 (–11) pairs per pinna, elliptic, obovate to orbicular, with a rounded, acuminate or emarginate apex, c. 2–25 × 3–12 mm, leaflet blades glabrous to pubescent, eglandular. Inflorescence a terminal or axillary raceme; bracts caducous (acuminate and filiform in *Denisophytum stuckertii*). Flowers bisexual, zygomorphic; calyx a short hypanthium with 5 sepals, c. 4–10 mm long, eglandular, glabrous to finely pubescent, lower sepal cucullate and covering the other 4 sepals in bud, all sepals caducous, leaving a persistent free hypanthium ring on the pedicel as the fruit develops; petals 5, free, yellow, the median petal sometimes with red markings on the inner face of the blade, c. 5–10 mm long, obovate, petal claw almost absent (present in *Denisophytum madagascariense*); stamens 10, free, filaments pubescent and eglandular (8–11 mm long in *Denisophytum madagascariense*), anthers dorsifixed, glabrous to pubescent; ovary glabrous. Fruits coriaceous, oblong-elliptic, laterally compressed (but inflated in *Denisophytum madagascariense*), glabrous, eglandular pods with a tapering, sharp beak, 18–49 × 5–15 mm, elastically dehiscent, with twisting valves. Seeds ovoid, laterally compressed.

##### Geographic distribution.

*Denisophytum* comprises nine taxa in eight species, found across North America, South America and Africa, including Madagascar, a classical highly disjunct trans-continental distribution typical of lineages occupying the succulent biome sensu Schrire et al. (2005). Three species are distributed in Mexico, Florida, and the Caribbean, one species is endemic to Paraguay and Argentina, one is endemic to northern Madagascar, and the other three occur in northern Kenya, Somalia and Arabia. An evaluation of species limits is needed in this group.

##### Habitat.

Low deciduous seasonally dry tropical woodland or scrubland, also in open pineland or coastal plains and foothills. Species in Madagascar and Africa grow in limestone soils.

##### Etymology.

There is no indication of the etymology of *Denisophytum* in the posthumous publication of the generic name. Nevertheless, it is quite likely that the author, René Viguier, had intended to honour his friend and collaborator, Marcel Denis, a botanist with expertise in the genus *Euphorbia* in Madagascar. Sadly, M. Denis passed away prematurely at the age of 33 in 1929 (Allorge and Allorge 1930).

##### References.

Britton and Rose (1930); Burkart (1936: 84–86); Viguier (1949); Roti-Michelozzi (1957); Brenan (1967); Capuron (1967); Thulin (1983: 16–18; 1993: 344–347); Ulibarri (1996); Du Puy and Rabevohitra (2002); Barreto Valdés (2013).

**Figure 12.**
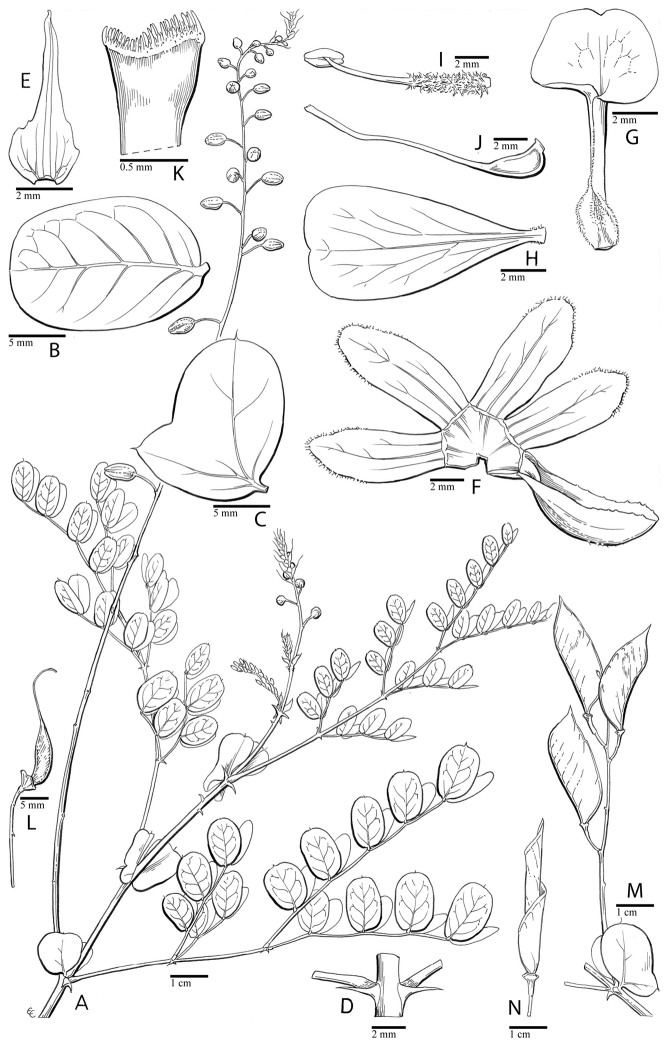
*Denisophytum stuckertii* (Hassl.) E. Gagnon & G. P. Lewis. **A** foliage and inflorescences **B** median leaflet undersurface **C** stipule **D** leaf rachis spines **E** bract **F** calyx opened out **G** median petal **H** lateral petal **I** stamen **J** gynoecium **K** stigma **L** developing ovary **M** infructescence, **N** single fruit valve after dehiscence. **A, B, D–K** from *Renvoize et al.* 3538 **C, M** from *Venturi* 7697 **L** from *Ruiz et al.* 10488c **N** from *Aguilar* 241. Drawn by Eleanor Catherine.

#### 6.1 Denisophytum bessac

Taxon classificationPlantaeFabalesLeguminosae

(Chiov.) E. Gagnon & G. P. Lewis comb. nov.

urn:lsid:ipni.org:names:77158013-1

##### Basionym.

*Caesalpinia bessac* Chiov., Flora Somala 1: 156. 1929.

##### Type.

SOMALIA, Uebi, Aug 1891, *Robecchi-Bricchetti 622* (FI).

*Denisophytum bessac* is based on depauperate material and is of dubious status (Thulin, 1993).

#### 6.2 Denisophytum buchii

Taxon classificationPlantaeFabalesLeguminosae

(Urb.) E. Gagnon & G. P. Lewis comb. nov.

urn:lsid:ipni.org:names:77158016-1

##### Basionym.

*Caesalpinia buchii* Urb., Symb. Antill. 7(4): 510. 1913.

##### Type.

HAITI, “inter Gonaïves et Grosmorne ad Perou”, *Buch 322* (holotype presumed at B†).

#### 6.3 Denisophytum eriantherum

Taxon classificationPlantaeFabalesLeguminosae

(Chiov.) E. Gagnon & G. P. Lewis comb. nov.

urn:lsid:ipni.org:names:77158014-1

##### Basionym.

*Caesalpinia erianthera* Chiov., Fl. Somala 1: 155. 1929.

##### Type.

SOMALIA, from Obbia to Wuarandi, Aug 1891, *Robecchi-Bricchetti 534* (syntype FI, fragments K!); and Boscaglia between Attod and Doldobscio, Apr 1924, *Puccioni & Stefanini 450* (syntype FI).

#### 6.3.1 Denisophytum eriantherum var. eriantherum

Taxon classificationPlantaeFabalesLeguminosae

#### 6.3.2 Denisophytum eriantherum var. pubescens

Taxon classificationPlantaeFabalesLeguminosae

(Brenan) E. Gagnon & G. P. Lewis comb. nov.

urn:lsid:ipni.org:names:77158021-1

##### Basionym.

Caesalpinia erianthera var. pubescens Brenan, Kew Bull. 17(2): 203. 1963.

##### Type.

KENYA, Northern Frontier Province, Banessa-Ramu, 23 May 1952, *Gillett 13274* (holotype K!; isotype EA).

#### 6.4 Denisophytum madagascariense

Taxon classificationPlantaeFabalesLeguminosae

R. Vig, Notul. Syst. (Paris) 13(4): 349. 1949

Caesalpinia madagascariensis (R. Vig.) Senesse, Bull. Mus. Nat. Hist. Nat., B, Adansonia. 10(1): 79. 1988.

##### Type.

MADAGASCAR, Loky R. basin, *Perrier de la Bâthie 4147* (holotype P).

*Caesalpinia antsiranensis* Capuron, Adansonia, sér. 2, 7: 203. 1967.

Type. MADAGASCAR, NE of Diego Suarez [Antsiranana], Orangea, *Capuron 22990-SF* (holotype P).

#### 6.5 Denisophytum pauciflorum

Taxon classificationPlantaeFabalesLeguminosae

(Griseb.) E. Gagnon & G. P. Lewis comb. nov.

urn:lsid:ipni.org:names:77158017-1

##### Basionym.

*Libidibia pauciflora* Griseb., Cat. Pl. Cub.: 78. 1866, (as “Lebidibia”).

*Poinciana pauciflora* (Griseb.) Small, Fl. SE United States: 59. 1903.

*Caesalpinia pauciflora* (Griseb.) C. Wright ex Sauvalle, Anal. Acad. Cienc. Med. Habana 5: 404. 1868 [1869].

**Type.** CUBA or. et occ., *Wright 2361* (holotype ?GOET, n.v., isotype K!).

#### 6.6 Denisophytum rosei

Taxon classificationPlantaeFabalesLeguminosae

(Urb.) E. Gagnon & G. P. Lewis comb. nov.

urn:lsid:ipni.org:names:77158018-1

##### Basionym.

*Caesalpinia rosei* Urb., Repert. Sp. Nov. Regni Veg. 15: 314. 1918.

##### Type.

DOMINICAN REPUBLIC (Santo Domingo) prope Azua, *Rose, Fitch & Russell 3861* (holotype US, photo K!).

#### 6.7 Denisophytum sessilifolium

Taxon classificationPlantaeFabalesLeguminosae

(S. Watson) E. Gagnon & G. P. Lewis comb. nov.

urn:lsid:ipni.org:names:77158015-1

##### Basionym.

*Caesalpinia sessilifolia* S. Watson, Proc. Amer. Acad. Arts and Sci. 21: 450 (1886).

*Poinciana sessilifolia* (S. Watson) Rose, in Contrib. U. S. Nat. Herb. 13(9): 303 (1911).

##### Type.

MEXICO, Bolson de Mapimi, 10 May 1847, *Gregg* s.n. (syntype NY); Mexico, Coahuila, on hills and mesas about Jumulco, May 1885, *Pringle* 202 (syntypes BR, CAS, CORD!, E, F, GH, GOET, JE, K!, MO, PH, SI!, US).

#### 6.8 Denisophytum stuckertii

Taxon classificationPlantaeFabalesLeguminosae

(Hassl.) E. Gagnon & G. P. Lewis comb. nov.

urn:lsid:ipni.org:names:77158019-1

##### Basionym.

*Caesalpinia stuckertii* Hassl., in Repert. Sp. Nov. Reg. Veg. 12: 201 (1913).

##### Type.

ARGENTINA, Prov. Tucuman, Dept. Bunyacu: prope Cañada Alegre, 5 Jan 1900, *Stuckert* 21276 (? holotype SI).

*Caesalpinia herzogii* Harms, in Meded. Rijks-Herb. 27: 38 (1915).

Type. ARGENTINA, Gran Chaco: near Camoteras, Nov 1910, *Herzog* 1077 (? holotype L).

Caesalpinia stuckertii var. robusta Hassl., in Repert. Sp. Nov. Reg. Veg.12: 202. 1913.

Type. ARGENTINA, Prov. Tucuman, Depto. Bunyacu: Cañada Alegre, 31 Dec 1908, *Stuckert 19726* (? holotype SI).

#### 7. Tara

Taxon classificationPlantaeFabalesLeguminosae

Molina, Saggio Chili 283. 1789, descr. emended E. Gagnon & G. P. Lewis

Figs 13 , 14C–I

Coulteria Kunth. 1824, in large part (excluding Coulteria mollis Kunth).
Nicarago Britton & Rose. 1930.
Russellodendron Britton & Rose. 1930.

##### Diagnosis.

*Tara* differs from the closely related *Coulteria* in having racemose or paniculate inflorescences of bisexual flowers (vs. racemose inflorescences of unisexual flowers), indehiscent, laterally compressed, oblong, straight, slightly turgid and somewhat fleshy, coriaceous, sessile pods (vs. chartaceous to papyraceous, laterally-compressed, oblong to elliptic, occasionally suborbicular, pods, with a stipe ca. 4–13 mm long), and ellipsoid (vs. ovate-orbicular to sub-quadrate, compressed) seeds.

##### Type.

*Tara tinctoria* Molina ≡ *Tara spinosa* (Molina) Britton & Rose

##### Emended description.

Shrubs or trees, 3–5 (– 8) m tall, armed with deflexed prickles on the shoots; twigs glabrous to puberulent. Stipules not seen. Leaves alternate, bipinnate, ending with a pair of pinnae, sometimes armed with prickles at the base of the pinnae and leaflets; pinnae in 2–5 opposite pairs; leaflets opposite, in 1–8 pairs per pinna, obovate, broadly elliptic to oblong-elliptic, apex rounded, obtuse, to slightly emarginate, base equal or asymmetrical, rounded to cuneate, 10–46 × 7–35 mm, eglandular, glabrous or pubescent on lower surface; primary vein centric, secondary venation reticulate. Inflorescences in terminal or axillary racemes or panicles, rachis c. 5–30 cm long, glabrous or puberulous, eglandular, unarmed; bracts minute, usually under 3 mm long, with a long acuminate tip, caducous. Flowers bisexual, zygomorphic; calyx a hypanthium with five sepals that are 6–9 mm long, eglandular, glabrous to puberulous, lower sepal cucullate covering the other 4 sepals in bud, with a pectinate, fimbriate or entire margin, sepals caducous, but the hypanthium persisting as a calyx ring around the pedicel as the pod matures; petals 5, free, yellow, the median petal with red markings, c. 10 mm long; stamens 10, free, the filaments pubescent, eglandular. Fruit an indehiscent, straight, oblong, laterally compressed, slightly turgid and somewhat fleshy, coriaceous pod, 4–15 × 1.2–4 cm, eglandular, often puberulent when young, glabrescent. Seeds ellipsoid, c. 8–10 mm diameter, brown, shiny.

##### Geographic distribution.

A genus of three species, one in South America (*Tara spinosa* thought to be native to Peru and Ecuador), one in Mexico (*Tara cacalaco*) and one in Mexico, Guatemala, Nicaragua and extending into the Caribbean (*Tara vesicaria*). *Tara spinosa* is also widely cultivated across the tropics and subtropics (including in the Canary Islands) as a source of tannins and occasionally as an ornamental.

##### Habitat.

Seasonally dry tropical forest to semi-arid thorn scrub.

##### Etymology.

Derived from the vernacular name ‘tara’ in Peru, Bolivia and Chile.

##### Notes.

Based on Gagnon et al. (2013), Molinari-Novoa and Sánchez Ocharan (2016) transfered *Caesalpinia cacalaco* and *Caesalpinia vesicaria* to the genus *Tara*, but did not emend the description of the genus, which we provide above.

##### References.

Britton and Rose (1930); Sprague (1931); Macbride (1943, as *Caesalpinia spinosa*, 195-196); Ulibarri (1996); Barreto Valdés (2013); Molinari-Novoa and Sánchez Ocharan (2016).

**Figure 13.**
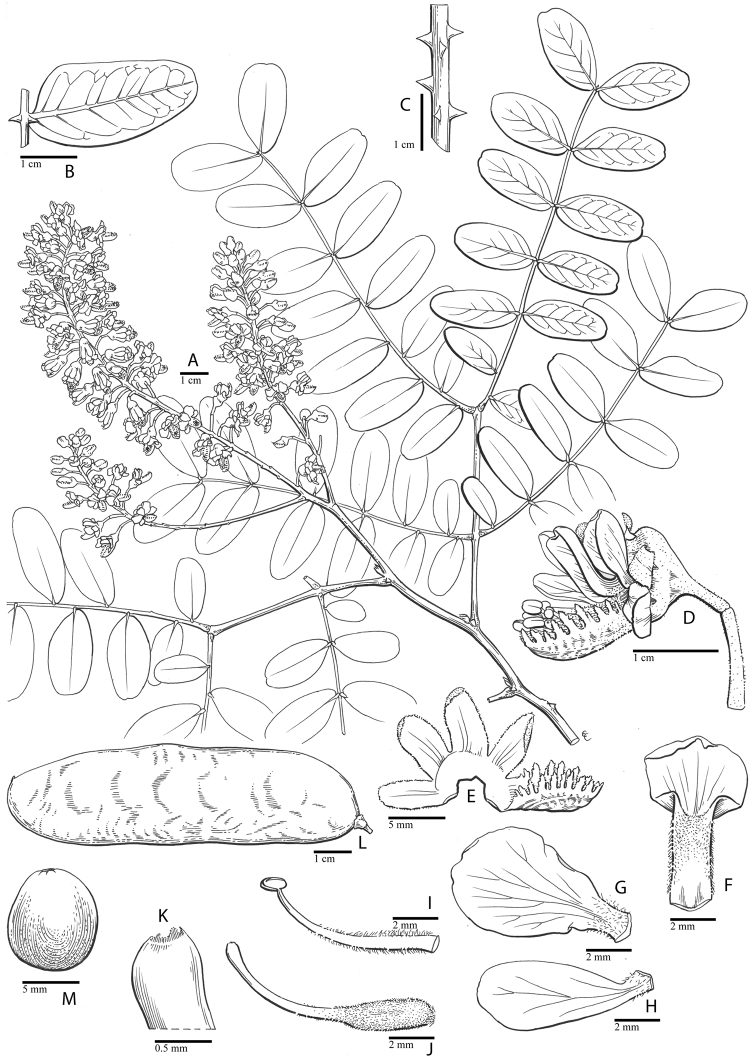
*Tara spinosa* (Molina) Britton & Rose. **A** habit **B** leaflet undersurface, **C** section of young stem **D** flower **E** calyx opened out **F** median petal **G** upper lateral petal **H** lower lateral petal **I** stamen **J** gynoecium **K** stigma **L** fruit **M** seed. **A–K** from *Lewis* 1416 **L, M** from *Filskov et al.* 37341. Drawn by Eleanor Catherine.

**Figure 14.**
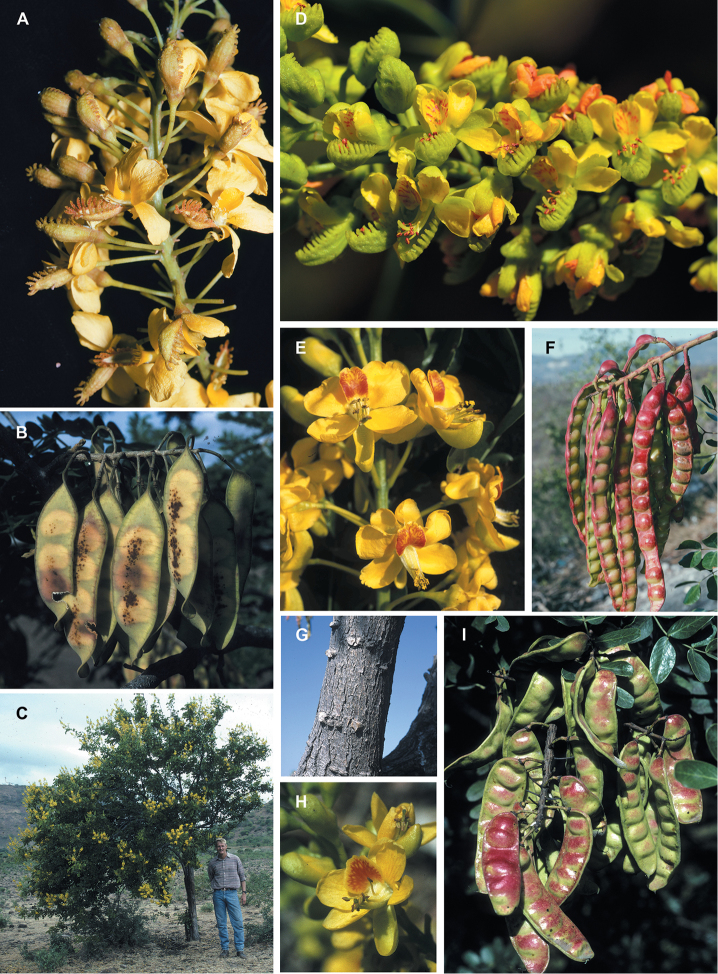
Caesalpinia (Coulteria) velutina Britton & Rose. **A** inflorescence (G. P. Lewis, Guatemala, *Lewis et al. 1713* (K)) **B** fruits (C. E. Hughes, Guatemala, *Lewis et al. 1714* (K)). *Tara vesicaria* (L.) Molinari, Sánchez Och. & Mayta **C** habit (C. E. Hughes, Tecolostote, Nicaragua, *Hughes 1376* (FHO)) **H** flower (C. E. Hughes, Rivas, Nicaragua, *J. A. Hawkins 11* (FHO). *Tara spinosa* (Molina) Britton & Rose **D** inflorescence (E. Gagnon, Ancash, Peru, *Hughes et al. 3043* (MT)) **I** unripe fruits (C. E. Hughes, Cajamarca, Peru, *Hughes 1996* (FHO)). *Tara cacalaco* (Humb. & Bonpl.) Molinari & Sánchez Och. **E** flowers (C. E. Hughes, Puebla, Mexico, *Hughes et al. 2169 (FHO)*) **F** unripe fruits (G. P. Lewis, Mexico, *MacQueen 488 (K)*) **G** bark (C. E. Hughes, Puebla, Mexico, *Hughes et al. 2073* (FHO)).

#### 7.1 Tara cacalaco

Taxon classificationPlantaeFabalesLeguminosae

(Humb. & Bonpl.) Molinari & Sánchez Och.

#### 7.2 Tara spinosa

Taxon classificationPlantaeFabalesLeguminosae

(Feuillé ex Molina) Britton & Rose

#### 7.3 Tara vesicaria

Taxon classificationPlantaeFabalesLeguminosae

(L.) Molinari, Sánchez Och. & Mayta

#### 8. Coulteria

Taxon classificationPlantaeFabalesLeguminosae

Kunth, Nov. Gen. Sp. 6 ed. fol. 258 (1824), 6 ed. qu. 328. 1824 (excluding t. 568 et 569 which ≡ Tara spinosa (Molina) Britton & Rose. 1824), descr. emended E. Gagnon, Sotuyo & G. P. Lewis

Figs 14A–B , 15

Brasilettia sensu Britton & Rose (1930), non (DC.) Kuntze (1891).
Guaymasia Britton & Rose (1930).

##### Diagnosis.

*Coulteria* differs from *Tara* by its racemose inflorescences of unisexual flowers (vs. inflorescences of racemes and panicles with bisexual flowers), chartaceous to papyraceous, laterally-compressed, oblong to elliptic (occasionally suborbicular) stipitate pods, subtended by a 4–13 mm long stipe (vs. indehiscent, laterally compressed but slightly turgid and somewhat fleshy, coriaceous, straight, oblong, sessile pods), and compressed, ovate-orbicular to sub-quadrate, compressed (vs. ellipsoid) seeds.

##### Type.

No type designated in the original publication, nor since. Type designated here: *Coulteria mollis* Kunth.

##### Emended description.

Trees or shrubs, 3–20 m tall, unarmed; young twigs with a dense velvety-bronze pubescence, glabrescent. Stipules not seen. Leaves alternate, bipinnate, ending in a pair of pinnae; petiole and rachis glabrous or densely velutinous; pinnae in 2–6 pairs; leaflets in (2–) 4–12 (– 14) pairs per pinna, 0.6–8 cm long, elliptic, oblong to ovate, apex obtuse to acute, base narrow, rounded or obtuse, eglandular, glabrous to velvety pubescent; main vein centric, secondary veins brochidodromous. Inflorescence racemose, axillary or terminal, 5–16 (– 25) cm long; bracts minute, with an acute tip, pubescent, caducous. Flowers unisexual, male and female flowers on separate trees, zygomorphic; calyx comprising a hypanthium with 5 sepals, 8–10 mm long, velvety-pubescent, lower sepal cucullate, glandular-pectinate, covering the other 4 sepals in bud; petals 5, yellow, free; male flowers with 10 free stamens, filaments pubescent, eglandular. Fruit chartaceous to papyraceous, laterally-compressed, oblong to elliptic (occasionally suborbicular), indehiscent (or sometimes opening along one suture), wingless, 3–15 × 2–4 cm, with a 4–13 mm long stipe, pendulous, often persisting to next flowering season, eglandular, glabrous to densely velutinous, 1–6-seeded. Seeds ovate orbicular or sub-quadrate, compressed.

##### Geographic distribution.

A genus of approximately seven species in Mexico and Central America, one species extending to Cuba, Jamaica and Curaçao, one to Venezuela (including Isla Margarita) and Colombia.

##### Habitat.

Seasonally dry tropical forest, deciduous woodland and dry thorn scrub, some species occurring on limestone.

##### Etymology.

Named by Kunth for the Irish botanist Thomas Coulter (1793–1846) who collected in central Mexico (1825–1834) and was curator of the herbarium at Trinity College, Dublin, Ireland.

##### Notes.

A revision of the genus has been submitted by S. Sotuyo, J. L. Contreras, E. Gagnon, and G. P. Lewis. The list of species names presented here simply includes all names associated with the genus *Coulteria* and will be reduced in the forthcoming taxonomic account.

##### References.

Britton and Rose (1930: 320–322); Ulibarri (1996); Zamora Villalobos (2010); Sotuyo et al. (submitted)

**Figure 15.**
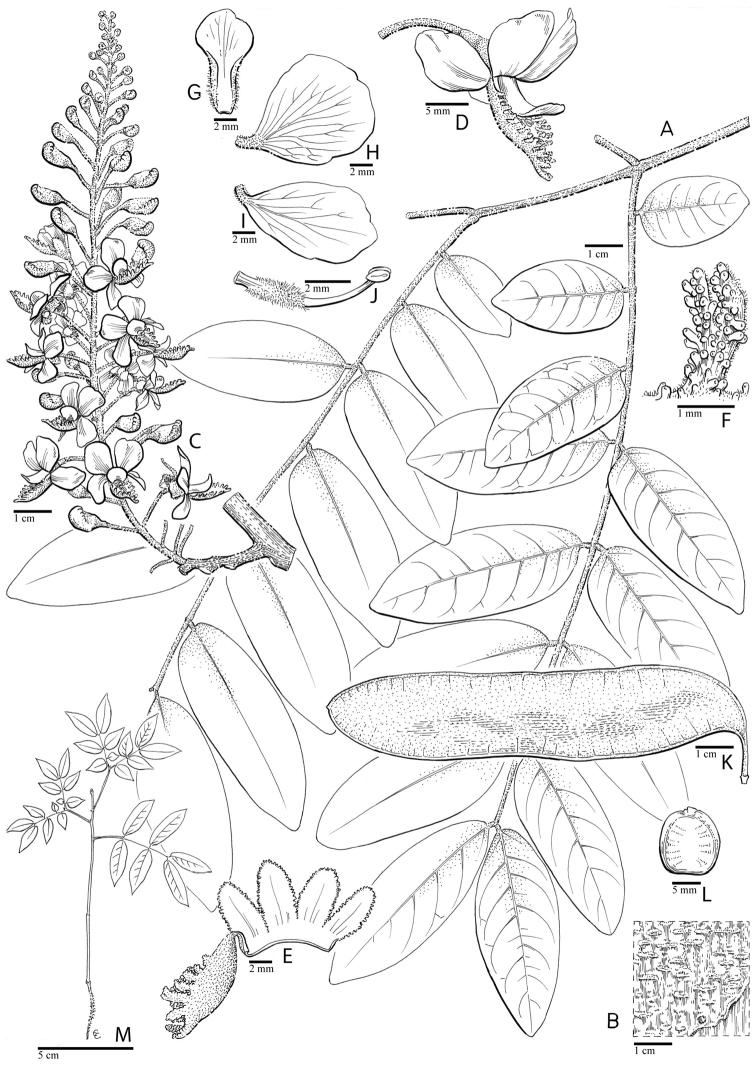
Caesalpinia (Coulteria) velutina Britton & Rose. **A** portion of leaf **B** detail of bark **C** inflorescence **D** flower **E** calyx opened out **F** detail of calyx lobe **G** median petal **H** upper lateral petal **I** lower lateral petal **J** stamen **K** fruit **L** seed **M** seedling. **A, K** from *Lewis and Hughes* 1714 **B–J, M** from *Lewis and Hughes* 1713. Drawn by Eleanor Catherine.

#### 8.1 Brasilettia glabra

Taxon classificationPlantaeFabalesLeguminosae

Britton & Rose

#### 8.2 Brasilettia pilosa

Taxon classificationPlantaeFabalesLeguminosae

Britton

#### 8.3 Brasilettia pubescens

Taxon classificationPlantaeFabalesLeguminosae

Britton

#### 8.4 Brasilettia pringlei

Taxon classificationPlantaeFabalesLeguminosae

Britton & Rose

#### 8.5 Brasilettia velutina

Taxon classificationPlantaeFabalesLeguminosae

Britton & Rose

#### 8.6 Caesalpinia acutifolia

Taxon classificationPlantaeFabalesLeguminosae

J. R. Johnst.

#### 8.7 Caesalpinia blasiana

Taxon classificationPlantaeFabalesLeguminosae

M. E. Jones

#### 8.8 Caesalpinia colimensis

Taxon classificationPlantaeFabalesLeguminosae

J. F. Herm.

#### 8.9 Caesalpinia cubensis

Taxon classificationPlantaeFabalesLeguminosae

Greenm. ex Combs

#### 8.10 Caesalpinia violacea

Taxon classificationPlantaeFabalesLeguminosae

(Mill.) Standl.

#### 8.11 Coulteria mollis

Taxon classificationPlantaeFabalesLeguminosae

Kunth

#### 8.12 Coulteria platyloba

Taxon classificationPlantaeFabalesLeguminosae

(S. Watson) N. Zamora

#### 8.13 Guaymasia pumila

Taxon classificationPlantaeFabalesLeguminosae

Britton & Rose

#### 8.14 Peltophorum linnaei

Taxon classificationPlantaeFabalesLeguminosae

Benth.

#### 8.15 Caesalpinia gracilis

Taxon classificationPlantaeFabalesLeguminosae

Benth. ex Hemsl.

#### 9. Gelrebia

Taxon classificationPlantaeFabalesLeguminosae

E. Gagnon & G. P. Lewis gen. nov.

urn:lsid:ipni.org:names:60473338-2

Figs 11J , 16

##### Diagnosis.

*Gelrebia* is morphologically similar to *Caesalpinia* s. s. but the two genera differ somewhat in habit, with *Gelrebia* species being erect to scrambling shrubs (vs. erect shrubs or small trees), in having dark pinkish mauve to light pinkish-white flowers (vs. flowers that are variable in colour, from yellow, white, red and orange to green), and coriaceous, broadly oblong-ovoid to obliquely pyriform pods, with a large, oblique, rounded base (vs. coriaceous, oblong-elliptic to linear pods, with an oblique cuneate base).

##### Type.

*Gelrebia rubra* (Engl.) E. Gagnon & G. P. Lewis ≡ *Hoffmannseggia rubra* Engl.: *Caesalpinia rubra* (Engl.) Brenan

##### Description.

Erect to scambling shrubs, 0.3–5 m tall, armed with scattered, straight or curved, deflexed prickles (these 7–20 mm long); stems puberulous to pubescent when young, glabrescent. Stipules not seen. Leaves alternate, bipinnate, ending in a pair of pinnae; pinnae opposite, in 1–17 pairs; leaflets opposite (except in *Gelrebia glandulosopedicellata*), in 1–33 pairs per pinna, narrowly oblong or oblong-elliptic, 3–11 × 2–5 mm, apex rounded to emarginate, sometimes mucronate, glabrous or sparsely pubescent, lower surface of the blades with numerous subepidermal glands or translucent dots (best seen with a × 10 hand lens or microscope). Inflorescence a terminal or axillary raceme, c. (1–) 2–19 (– 25) cm long, unarmed; bracts broadly ovate to suborbicular, apex aristate, 3–10 mm long, caducous. Flowers bisexual, zygomorphic; calyx comprising a short hypanthium with 5 sepals, c. 5–13 mm long, eglandular, glabrous to finely pubescent, lower sepal strongly cucullate (occasionally with a beaked apex), covering the other 4 sepals in bud before anthesis, all sepals caducous, but hypanthium persisting as a free ring around the pedicel as the pod matures; petals 5, free, dark pinkish mauve to light pinkish-white, c. 7–24 × 5–15 mm, eglandular; stamens 10, free, filaments 8–20 mm long, pubescent and eglandular; ovary glabrous. Fruit a coriaceous, broadly oblong-ovoid to obliquely pyriform pod, apex acute, with a large, oblique, rounded base, c. 15–40 × 12–23 mm, dehiscent along both sutures, glabrous to minutely pubescent, eglandular. Seeds obovoid, laterally compressed.

##### Geographic distribution.

A genus of nine taxa in eight species, restricted to Africa, in Namibia, Botswana, South Africa, Northern Kenya, Ethiopia, and Somalia. One species also found in the Democratic Republic of the Congo (Zaire, Katanga).

##### Habitat.

Deciduous bushland, dry woodlands, on rocky ridges, often along dry river beds, or on sandy valley floors. One species also found in degraded savanna, close to termite mounds.

##### Etymology.

Gelreb or gelrib is the Somali name for Gelrebia trothae subsp. erlangeri (field labels of Dale K724 (“gelrib”) and of Gillett 13223 (“gelreb”) from Kenya), meaning ‘camel trap’ and clearly alluding to the robust deflexed prickles characteristic of the species, and indeed the genus as a whole, which can hinder the passage of camels.

##### References.

Wilczek (1951); Roti-Michelozzi (1957); Brenan (1963, 1967); Ross (1977: 122–130); Thulin (1980, 1983: 16–18; 1993: 344–347); Germishuizen (1991); Roux (2003); Curtis and Mannheimer (2005: 226–228); Brummitt et al. (2007).

**Figure 16.**
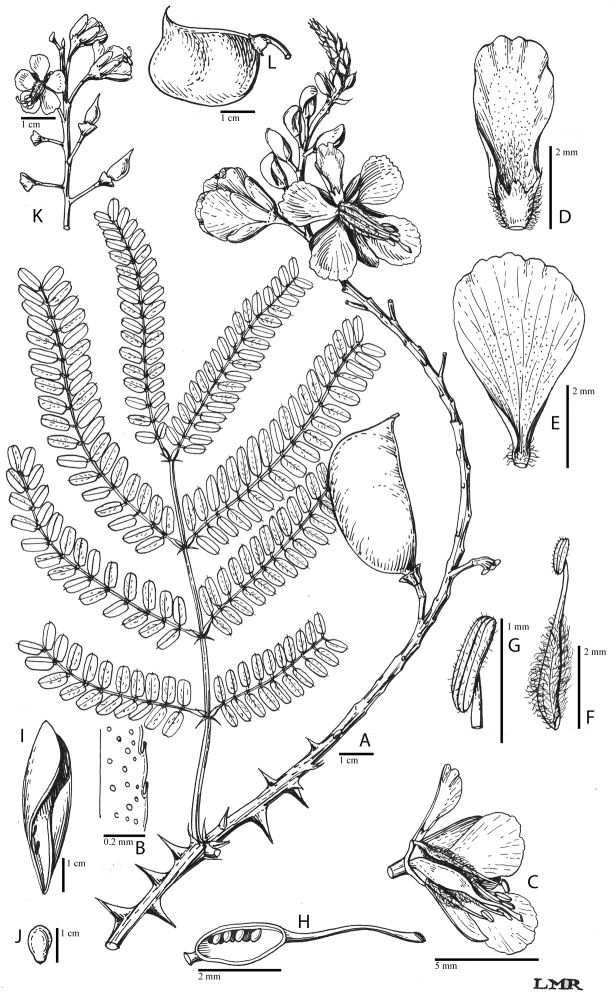
Gelrebia trothae E. Gagnon & G. P. Lewis subsp. trothae. **A** part of branch showing inflorescence with flowers and fruits **B** portion of leaflet margin, lower surface **C** longitudinal section of flower **D** median petal inner surface **E** lateral petal inner surface **F** stamen **G** anther **H** ovary with part of wall removed to expose ovules **I** fruit valve after dehiscence **J** seed. Gelrebia trothae subsp. erlangeri (Harms) E. Gagnon & G. P. Lewis **K** part of inflorescence **L** fruit. **A–H** from *Milne-Redhead & Taylor* 11177 **I, J** from *Ward* U27 **K** from *Gillett* 13223 **L** from *Hemming* 478. Drawn by L. M. Ripley, originally published in F.T.E.A., Leguminosae subfamily Caesalpinioideae, page 34, fig. 5 (1967).

#### 9.1 Gelrebia bracteata

Taxon classificationPlantaeFabalesLeguminosae

(Germish.) E. Gagnon & G. P. Lewis comb. nov.

urn:lsid:ipni.org:names:60473339-2

##### Basionym.

*Caesalpinia bracteata* Germish., Bothalia 21 (2): 153. 1991.

##### Type.

[South Africa, Cape Province]: “2819 (Ariamsvlei): Kenhardt District, on farm Skroef, near hot spring (Warmbad Noord) on Orange River (-DA)”, 29 Sep 1987, *Van Hoepen 1941* (holotype PRE).

#### 9.2 Gelrebia dauensis

Taxon classificationPlantaeFabalesLeguminosae

(Thulin) E. Gagnon & G. P. Lewis comb. nov.

urn:lsid:ipni.org:names:60473340-2

##### Basionym.

*Caesalpinia dauensis* Thulin, Kew Bull. 34(4): 819. 1980.

##### Type.

KENYA, 30 km on the Ramu-Malka road, c. 4°04'N, 40°59'E, 8 May 1978, *Gilbert & Thulin 1583* (holotype UPS; isotypes BR, EA, K!).

#### 9.3 Gelrebia glandulosopedicellata

Taxon classificationPlantaeFabalesLeguminosae

(R. Wilczek) E. Gagnon & G. P. Lewis comb. nov.

urn:lsid:ipni.org:names:60473341-2

##### Basionym.

*Caesalpinia glandulosopedicellata* R. Wilczek, Bull. Jard. Bot. Brux. 21: 83. 1951.

##### Type.

“Congo Belge”, district du Haut-Katanga: environs de Niemba, *Schmitz 1595*.

#### 9.4 Gelrebia merxmuellerana

Taxon classificationPlantaeFabalesLeguminosae

(A. Schreib.) E. Gagnon & G. P. Lewis comb. nov.

urn:lsid:ipni.org:names:60473342-2

##### Basionym.

*Caesalpinia merxmuellerana* A. Schreib., Mitt. Bot. St. Munchen 16, Beih., Die Gattung *Caesalpinia* in Südwestafrica, 64. 1980.

##### Type.

SOUTH WEST AFRICA, Dist. Lüderitz-Süd, Farm Uitsig, *Wendt* in herb. *W. Giess 14713* (holotype M; isotypes K!, PRE, WIND).

#### 9.5 Gelrebia oligophylla

Taxon classificationPlantaeFabalesLeguminosae

(Harms) E. Gagnon & G. P. Lewis comb. nov.

urn:lsid:ipni.org:names:60473343-2

##### Basionym.

*Caesalpinia oligophylla* Harms, Engl., Bot. Jahrb. Syst. 33: 160. 1902.

##### Type.

ETHIOPIA, “Arussi Galla”, Apr 1901, *Ellenbeck 2038* (holotype B †); SOMALIA, rive dello Scebelia Bulo Burti, 25 Feb 1924, *Puccioni & Stefanini 134* (neotype FI, designated by G. Roti-Michelozzi in Webbia 13: 207. 1957).

#### 9.6 Gelrebia rostrata

Taxon classificationPlantaeFabalesLeguminosae

(N.E.Br.) E. Gagnon & G. P. Lewis comb. nov.

urn:lsid:ipni.org:names:60473344-2

##### Basionym.

*Caesalpinia rostrata* N. E. Br., Hooker's Icon. Pl., 28: t. 2702. 1901.

##### Type.

SOUTH AFRICA, from cultivation in Durban Botanic Garden, raised from seed obtained from “Delagoa Bay”, Maputo (Lourenço Marques), *Wood 7943* (holotype K!; isotypes BOL, NH, PRE).

#### 9.7 Gelrebia rubra

Taxon classificationPlantaeFabalesLeguminosae

(Engl.) E. Gagnon & G. P. Lewis comb. nov.

urn:lsid:ipni.org:names:77158022-1

##### Basionym.

*Hoffmannseggia rubra* Engl., Bot. Jahrb. Syst. 10: 25. 1889. *Caesalpinia rubra* (Engl.) Brenan, Kew Bull. 17(2): 202. 1963.

##### Type.

NAMIBIA, Karibib Dist., Usakos, *Marloth 1432* (holotype ?B; isotypes BOL, PRE).

#### 9.8 Gelrebia trothae

Taxon classificationPlantaeFabalesLeguminosae

(Harms) E. Gagnon & G. P. Lewis comb. nov.

urn:lsid:ipni.org:names:60473345-2

##### Basionym.

*Caesalpinia trothae* Harms, Engl., Bot. Jahrb. Syst., 26: 277. 1899, as “trothaei”.

##### Type.

TANZANIA, ?Dodoma District, Ugogo, Chumo Pass, Jan. 1897, *von Trotha 186* (holotype B †).

#### 9.8.1 Gelrebia trothae subsp. trothae

Taxon classificationPlantaeFabalesLeguminosae

#### 9.8.2 Gelrebia trothae subsp. erlangeri

Taxon classificationPlantaeFabalesLeguminosae

(Harms) E. Gagnon & G. P. Lewis comb. nov.

urn:lsid:ipni.org:names:60473346-2

##### Basionym.

*Caesalpinia erlangeri* Harms, Engl., Bot. Jahrb. Syst. 33: 160. 1902.

Caesalpinia trothae subsp. erlangeri (Harms) Brenan, Kew Bull. 17(2): 20. 1963.

##### Type.

ETHIOPIA, Galla Sidama, Borana, Tarro Gumbi, *Ellenbeck 2071* (holotype B †). Somalia, Dolo, sul Daua, 6 May 1893, *Riva 1104* (neotype FI, designated by G. Roti-Michelozzi in Webbia 13: 209, 1957).

#### 10. Hultholia

Taxon classificationPlantaeFabalesLeguminosae

E. Gagnon & G. P. Lewis gen. nov.

urn:lsid:ipni.org:names:77158067-1

Figs 17 , 18

##### Diagnosis.

*Hultholia* is closely related and morphologically similar to *Guilandina*. While both genera form armed lianas, *Hultholia* differs in having stems with dome-shaped glands intermixed with dense slender, patent, needle-like prickles (vs. stems eglandular and with strongly recurved, robust prickles in *Guilandina*); both genera have sharp recurved prickles on the leaf and pinnae rachises. *Hultholia* has bisexual flowers (vs. unisexual flowers on separate female and male racemes in *Guilandina*), a zygomorphic corolla, with petals extending beyond the sepals, and the median (standard) petal smaller than the other four (vs. a sub-actinomorphic to zygomorphic corolla, with petals only slightly extending beyond the sepals in *Guilandina*), unarmed, obovoid, falcate, pubescent, vesicular pods (vs. oblong-elliptic, coriaceous, eglandular, inflated pods, usually armed with 5–10 mm long, slender spinescent bristles), and sub-globose, oblong, grey, ca. 10 × 7 mm, smooth seeds (vs. obovoid to globular c. 20 mm in diameter, grey, pale to dark brown or orange seeds, with parallel fracture lines concentric with the small apical hilum).

##### Type.

*Hultholia mimosoides* (Lam.) E. Gagnon & G. P. Lewis ≡ *Caesalpinia mimosoides* Lam.

##### Description.

Climbing woody shrub; branches densely armed with short, robust, needle-like trichomes; young stems pubescent, with rust-coloured, hyaline hairs and dome-shaped glands, topped with a few hairs. Stipules subulate, 7–15 mm long, pubescent, caducous. Leaves alternate, bipinnate, without a single terminal pinna, 22–40 cm long; pinnae opposite, in 10–30 pairs per leaf, about 3–5 cm long, pubescent, with a pair of deflexed prickles at the insertion of the pinnae on the leaf rachis, and at the insertion of leaflets on the pinnae rachises; leaflets opposite, in 7–20 pairs per pinna, oblong, asymmetric at base, c. 9 × 4 mm, glabrous, eglandular. Inflorescences terminal or leaf-opposed, lax racemes, with 50 or more flowers, 20–40 cm long; rachis and pedicels armed with needle-like, robust trichomes, pubescent and covered with domed, hair-tipped glands. Flowers bisexual, zygomorphic; calyx comprising a hypanthium with 5 sepals 13–16 × 6 mm; hypanthium and sepals pubescent and glandular, the sepal margins sometimes with small stipitate glands, < 1 mm long; petals 5, free, bright yellow, dark glands present on the blade, median (standard) petal c. 8 mm wide and smaller than the 4 lateral petals, that are c. 1.7 × 1.3 cm; stamens 10, free, filaments 1.8 cm long, pubescent at least on the lower ½; ovary densely pubescent, and with glandular dots (often obscured by the dense pubescence). Fruit an obovoid, falcate, vesicular, unarmed, dehiscent pod, sparsely pubescent, particularly along the margin, and with a few obscure stellate hairs, and covered in gland dots, 5–6 × 2.5–3 cm, 1–3-seeded. Seeds sub-globose, oblong, 10 × 7 mm, grey.

##### Geographic distribution.

The single species is distributed across Asia, in China (Yunnan), Bangladesh, India, Laos, Myanmar (Burma), Thailand and Vietnam.

##### Habitat.

In secondary thickets and clearings, often on roadsides, up to 1500 m elevation. More information on the ecology of this genus is needed.

##### Etymology.

The name *Hultholia* honours the Cambodian botanist Dr. Sovanmoly Hul Thol (born 1946), whose doctoral thesis, “Contribution à la révision de quelques genres de Caesalpiniaceae, representés en Asie” (1976), is an important revision of the Asian species and genera of the Caesalpinia group, and particularly the genus *Pterolobium*. Dr. Hul Thol retired from the Museum National d’Histoire Naturelle, Paris in 2014, but continues as an honorary researcher. She is a specialist on the flora of Cambodia and South East Asia, directed the publication of multiple volumes of the Flora of Cambodia, Laos and Vietnam from 1995, and is one of the co-founders of the National Herbarium of Cambodia, Royal University of Phnom Penh.

##### Notes.

Although *Hultholia mimosoides* is not known to be cultivated, the young, pungent, flowering shoots are sold as a vegetable in markets in Vientiane (Laos) (Vidal and Hul Thol 1976).

##### References.

Vidal and Hul Thol (1976); Chen et al. (2010a: 42–43).

**Figure 17.**
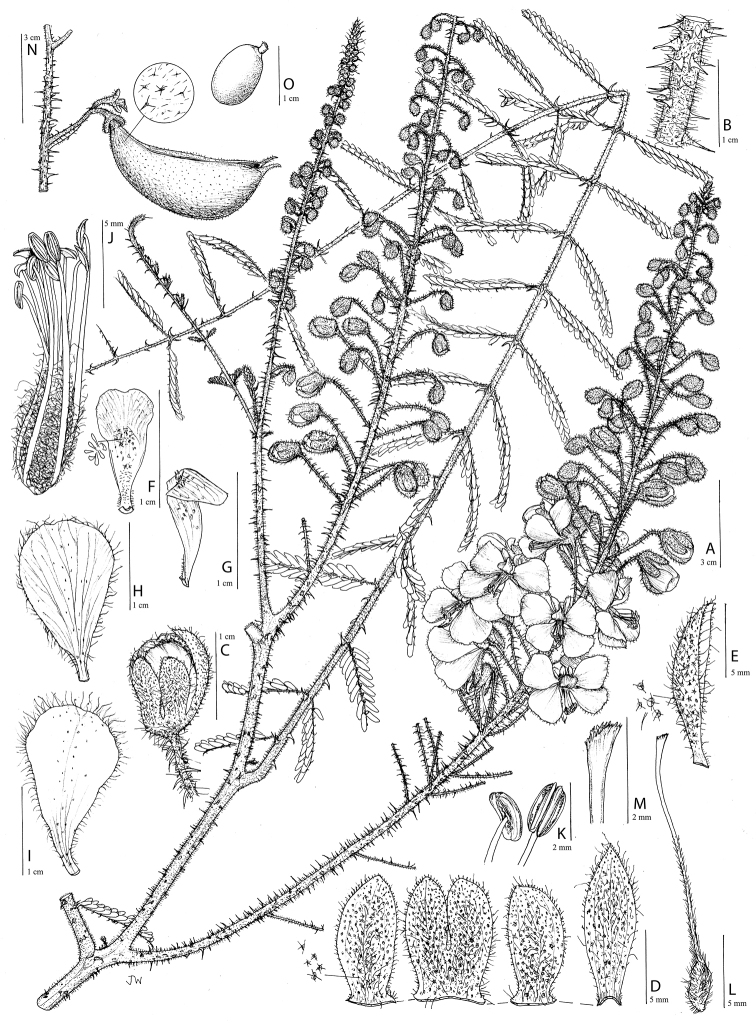
*Hultholia mimosoides* (Lam.) E. Gagnon & G. P. Lewis. **A** habit, including foliage and inflorescences **B** stem armature detail **C** bud showing cucullate lower lobe of calyx **D** calyx lobes outer surface **E** calyx cucullate lower lobe side view, **F** median petal inner surface **G** median petal side view **H** upper lateral petal inner surface **I** lower lateral petal inner surface **J** stamens **K** anthers dorsal and ventral views **L** gynoecium **M** stigma detail **N** fruit **O** seed. **A–K** from *Clark* 237 **L**, **M** from *Beusekom & Geesink* 4706 **N**, **O** from *Bunchuai* 1342. Drawn by Juliet Williamson.

**Figure 18.**
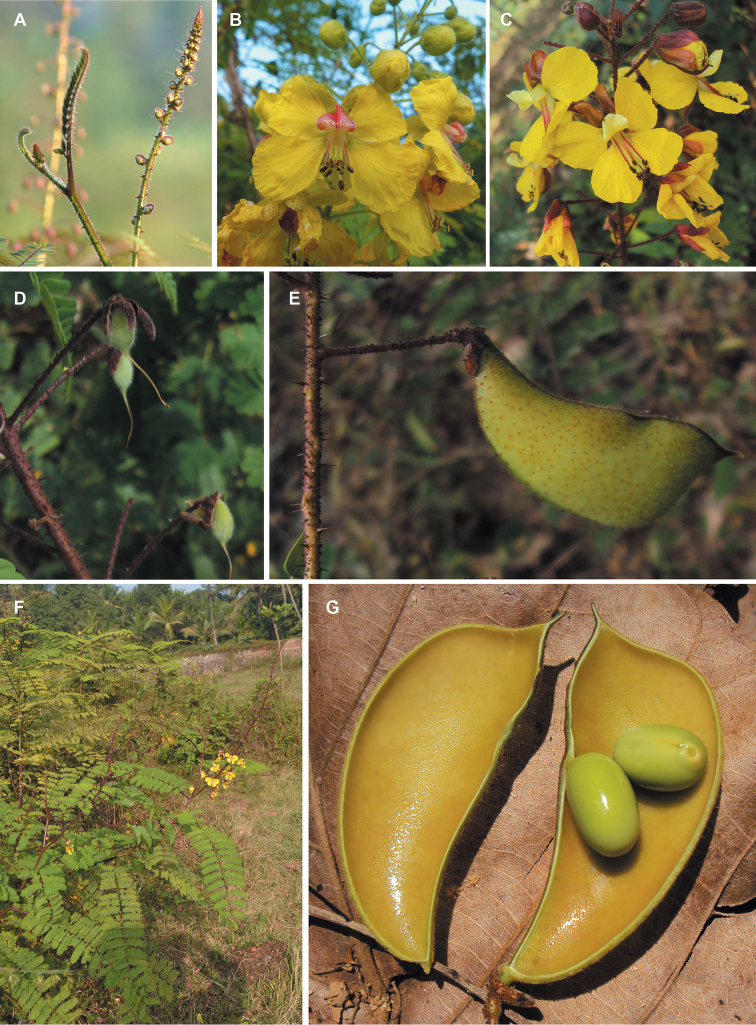
*Hultholia mimosoides* (Lam.) E. Gagnon & G. P. Lewis. **A** young leaves and inflorescence in bud (J. Jose, Wikicommons (https://commons.wikimedia.org/wiki/File:Caesalpinia_mimosoides_2_at_Kudayathoor.jpg), Kerala, India, *unvouchered*) **B** flower (R. Clark, Thailand, *Clark et al. 237* (K)) **C** flowers **D** immature fruits **E** mature fruit **F** habit **G** open fruit with seeds (V. R. Vinayaraj, Wikicommons (https://commons.wikimedia.org/wiki/Category:Caesalpinia_mimosoides, the basionym of *Hultholia mimosoides*), India, *unvouchered*).

#### 10.1 Hultholia mimosoides

Taxon classificationPlantaeFabalesLeguminosae

(Lam.) E. Gagnon & G. P. Lewis comb. nov.

urn:lsid:ipni.org:names:77158068-1

##### Basionym.

*Caesalpinia mimosoides* Lam., Encycl. Méth., Bot. 1(2): 462 (1785).

*Biancaea mimosoides* (Lam.) Tod., Hort. Bot. Panorm. 1(1): 3 (1875).

##### Type.

Specimen originally from Malabar, sent to Lamarck by Sonnerat (P: Herb. Lamarck, fide Vidal and Hul Thol. 1976).

#### 11. Guilandina

Taxon classificationPlantaeFabalesLeguminosae

L., Sp. Pl.: 381. 1753

Figs 19 , 20D–F

Bonduc Mill. (1754).
Caesalpinia subgenus Guilandina (L.) Gillis & Proctor (1974).

##### Type.

*Guilandina bonduc* L.

##### Description.

Lianas, woody climbers, scrambling or trailing shrubs, often forming dense tangled clumps, densely armed with recurved prickles on branches and shoots, as well as in pairs at leaf bases (except *Caesalpinia murifructa* and closely related species in the Caribbean which are unarmed). Stipules foliaceous to subulate, sub-persistent or caducous. Leaves bipinnate, ending with a pair of pinnae, prickles present in pairs at the insertion of pinnae and scattered on the leaf rachis, and at the insertion of leaflets on the pinnae rachises; leaflets oblong, apex obtuse and mucronulate to acuminate, base rounded. Inflorescences supra-axillary or terminal racemes, 30–60 cm long; bracts narrow, lanceolate, aristulate, 1 mm long, to conspicuous and exceeding floral buds, caducous. Flowers unisexual, segregated on separate male and female racemes, the female flowers cryptically bisexual with 10 fully formed stamens, but these produce no pollen; male flowers with a highly reduced, non-functional pistil, zygomorphic to sub-actinomorphic; calyx with a hypanthium and 5 almost equal sepals, these valvate in bud, the lower sepal slightly cucullate, the hypanthium and sepals caducous, leaving no persistent calyx ring, eglandular, without spines (except Madagascan *Caesalpinia delphinensis* in which the calyx is armed with slender prickles); petals 5, free, yellow, barely exceeding the sepals; stamens 10, free, pubescent near the filament base; ovary usually covered in bristly trichomes, except in a few species, including *Caesalpinia solomonensis* and *Caesalpinia murifructa*. Fruits oblong-elliptic, inflated pods, usually armed with 5–10 mm long spinescent bristles, apex terminating in a beak, base acute, 1–4-seeded. Seeds obovoid to globular, c. 2 cm in diameter, smooth, grey, pale to dark brown, or orange, with parallel fracture lines concentric with the small apical hilum.

##### Geographic distribution.

This pantropical genus lacks a recent global taxonomic account and there are doubts about the number of species, with previous estimates ranging from seven to as many as 19. Species occur from as far north as Japan, south to South Africa, with three species in the Caribbean, one in China, India, Myanmar (Burma), Indo China, Hong Kong and Taiwan, one endemic to Madagascar, one in Australia, and two widespread across the Old and New World tropics.

##### Habitat.

Coastal thickets on sand, in secondary forest, and lowland rain forest, occasionally on limestone.

##### Etymology.

Named by Linnaeus for Melchior Wieland (1515–1589), Prussian naturalist, traveller and scholar from Königsberg, who settled in Italy and italianised his name to ‘Guilandini’, or Guilandinus in Latin; he was sent to the Levant, Asia and Africa (1559–1560), was captured by pirates and finally ransomed by Gabriele Falloppio.

##### Notes.

Pending a complete taxonomic revision, the list of 19 names presented below provides a guide to potential species content in *Guilandina*, but includes no synonymy and no information on types, nor any new nomenclatural combinations for the five species of *Caesalpinia* that as yet have no published name in *Guilandina*.

##### References.

Britton and Rose (1930: 336–341); Wilczek (1951); Brenan (1967); Gillis and Proctor (1974); Hattink (1974); Vidal and Hul Thol (1976); Du Puy and Rabevohitra (2002: 46–48); Chen et al. (2010a).

**Figure 19.**
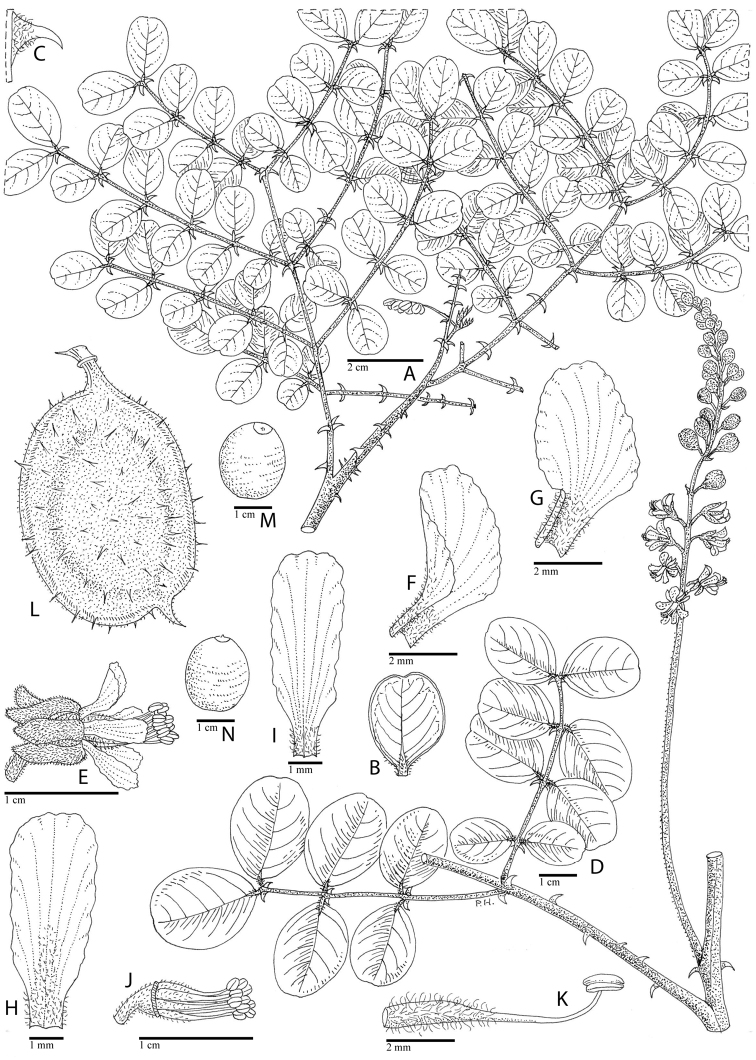
*Guilandina ciliata* Bergius ex Wikstrom. **A** foliage **B** leaflet undersurface **C** prickle enlarged to show indumentum **D** inflorescence and portion of leaf; **E** flower **F, G** median petal **H** upper lateral petal (outer surface) **I** lower lateral petal (inner surface) **J** stamens **K** stamen **L** fruit **M, N** seeds. **A–C** from *Ekman* 5413 **D–K** from *Curtiss* 143 **L–N** from *Pannell* 179. Drawn by Pat Halliday.

**Figure 20.**
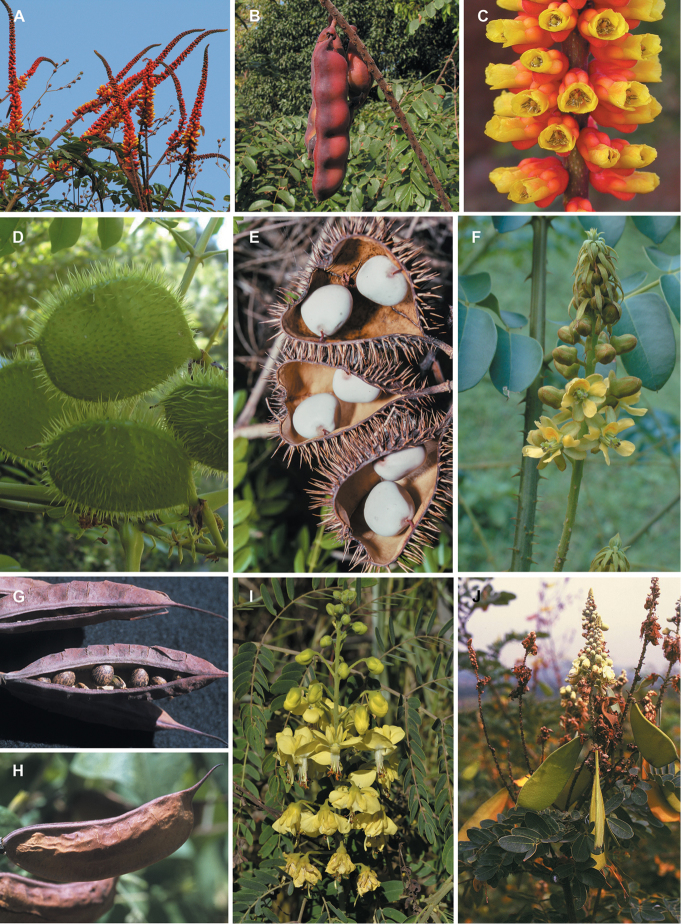
*Moullava spicata* (Dalzell) Nicolson. **A** inflorescences **B** fruit (P. Awale, Flowers of India (http://www.flowersofindia.net/), Maharashtra, India, *unvouchered*) **C** flowers (M. Sanjappa, India, *unvouchered*). *Guilandina bonduc* L. **D** young fruits (F. Starr and K. Starr, Starr Environmental (http://www.starrenvironmental.com/images/species/?q=Caesalpinia+bonduc), Florida, USA, unvouchered) **E** fruits with mature seeds (G. P. Lewis, Madagascar, *Du Puy et al. M665* (K)) **F** inflorescence (M. Sanjappa, India, *unvouchered*). *Biancaea decapetala* (Roth) O. Deg. **G** fruits with seeds **H** fruit with thickened suture (C. E. Hughes, Ancash, Peru, *Hughes et al. 2227* (FHO)) **I** inflorescence (E. Gagnon, Ancash, Peru, *Hughes et al. 3055* (MT)). *Biancaea godefroyana* (Kuntze) Molinari, Mayta & Sánchez Och. **J** inflorescences and fruits (F. Xaver, Wikicommons (https://commons.wikimedia.org/wiki/File:Caesalpinia_godefroyana_1.jpg), Cambodia, *unvouchered*).

#### 11.1 Guilandina barkeriana

Taxon classificationPlantaeFabalesLeguminosae

(Urb. & Ekman) Britton

#### 11.2 Guilandina bonduc

Taxon classificationPlantaeFabalesLeguminosae

L.

#### 11.3 Guilandina caymanensis

Taxon classificationPlantaeFabalesLeguminosae

(Millsp.) Britton & Rose

#### 11.4 Guilandina ciliata

Taxon classificationPlantaeFabalesLeguminosae

Bergius ex Wikstrom

#### 11.5 Guilandina culebrae

Taxon classificationPlantaeFabalesLeguminosae

Britton & Wilson ex Britton & Rose

#### 11.6 Caesalpinia delphinensis

Taxon classificationPlantaeFabalesLeguminosae

Du Puy & Rabev.

#### 11.7 Guilandina glaucophylla

Taxon classificationPlantaeFabalesLeguminosae

(Urb.) Britton & Rose

#### 11.8 Caesalpinia homblei

Taxon classificationPlantaeFabalesLeguminosae

R. Wilczek

#### 11.9 Guilandina intermedia

Taxon classificationPlantaeFabalesLeguminosae

(Urb.) Britton & Rose

#### 11.10 Guilandina major

Taxon classificationPlantaeFabalesLeguminosae

(DC.) Small

#### 11.11 Caesalpinia minax

Taxon classificationPlantaeFabalesLeguminosae

Hance

#### 11.12 Caesalpinia murifructa

Taxon classificationPlantaeFabalesLeguminosae

Gillis & Proctor

#### 11.13 Guilandina portoricensis

Taxon classificationPlantaeFabalesLeguminosae

Britton & Wilson

#### 11.14 Guilandina socorroensis

Taxon classificationPlantaeFabalesLeguminosae

Britton & Rose

#### 11.15 Caesalpinia solomonensis

Taxon classificationPlantaeFabalesLeguminosae

Hattink

#### 11.16 Guilandina sphaerosperma

Taxon classificationPlantaeFabalesLeguminosae

(Urb. & Ekman) Britton

#### 11.17 Guilandina urophylla

Taxon classificationPlantaeFabalesLeguminosae

(Donn. Sm.) Britton & Rose

#### 11.18 Caesalpinia volkensii

Taxon classificationPlantaeFabalesLeguminosae

Harms

#### 11.19 Guilandina wrightiana

Taxon classificationPlantaeFabalesLeguminosae

(Urb.) Britton & Rose

#### 12. Moullava

Taxon classificationPlantaeFabalesLeguminosae

Adans., Fam. Pl. 2: 318. 1763, descr. emended E. Gagnon & G. P. Lewis

Figs 20A–C , 21

Wagatea Dalzell (1851).
Cinclidocarpus Zoll. & Moritzi (1846).
Caesalpinia sect. Cinclidocarpus (Zoll. & Moritzi) Benth. & Hook. (1865).

##### Diagnosis.

*Moullava* is related to *Mezoneuron*, but differs by its fleshy, oblong-elliptic, indehiscent, sub-torulose, wingless pods, with thickened sutures (vs. laterally compressed, chartaceous, coriaceous or ligneous, indehiscent pods, with a longitudinal wing along the upper suture), and by its subglobular (vs. compressed) seeds.

##### Type.

“H.M. 6 t. 6” (= Rheede`s Hortus Malabaricus 6, plate 6, 1686) = *Moullava spicata*.

##### Emended description.

Lianas and scrambling shrubs, armed with deflexed prickles on shoots. Stipules not seen. Leaves alternate, bipinnate, ending with a pair of pinnae, 12–40 cm long, glabrous to pubescent-tomentose, with a pair of prickles at the insertion of each pinna; pinnae opposite, in 7–20 pairs; leaflets in 5–40 opposite pairs per pinna, sessile, narrowly oblong to ovate-oblong, apex rounded to emarginate, sometimes mucronate, base asymmetrical to rounded, blades eglandular, glabrous to pubescent, 4–20 × 2–6 mm. Inflorescence an elongated terminal or axillary raceme, the flowers subsessile, pedicels, when present, 10–25 mm long, the racemes sometimes aggregated into panicles, 8–60 cm long, unarmed or with a few prickles at the base. Flowers bisexual, sub-actinormophic or zygomorphic; calyx comprising a hypanthium with 5 sepals, 6–12 × 2–4 mm, the lower sepal strongly cucullate, covering the other 4 sepals in bud, all sepals eglandular and glabrous; petals 5, free, yellow, the median and lateral petals sometimes streaked red, eglandular; stamens 10, free, barely exserted beyond the corolla, densely pubescent on lower half of filaments, 8–15 mm long; ovary glabrous or pubescent. Fruit fleshy, oblong-elliptic, unarmed, indehiscent, sub-torulose, with thickened sutures, the apex apiculate, 35–50 (–80) × 15–30 mm, drying black (immature fruits of *Moullava spicata* red-tomentose), exocarp and endocarp strongly adnate, glabrous, 1–4-seeded. Seeds sub-globular, 12–20 mm in diameter, olive-brown to black.

##### Geographic distribution.

A genus of four species, three in south Asia: India, Nepal, Myanmar (Burma), Thailand, Laos, Cambodia, Sri Lanka, southern China (Yunnan and Hainan), and the Malay Peninsula and Archipelago, and one in Africa: Cameroun, Gabon, the Democratic Republic of Congo, Angola, Zambia (Kabompo Dist.), Uganda and Tanzania (Kigoma Dist.).

##### Habitat.

The Asian species are found in seasonally dry tropical semi-evergreen forest margins, secondary thickets, and on mountain slopes, up to 1200 m elevation. The African species occurs mostly in riverine habitats in lowland rainforests.

##### Etymology.

Derived from the vernacular name of *Moullava spicata*, “mulu” (Malayalam: spiny), a spiny climber.

##### References.

Brenan (1963, 1967); Hattink (1974); Vidal and Hul Thol (1976); Nicolson (1980); Ansari (1990); Sanjappa (1992: 33); Brummitt et al. (2007, see both *Moullava* and *Mezoneuron welwitschianum*); Chen et al. (2010a).

**Figure 21.**
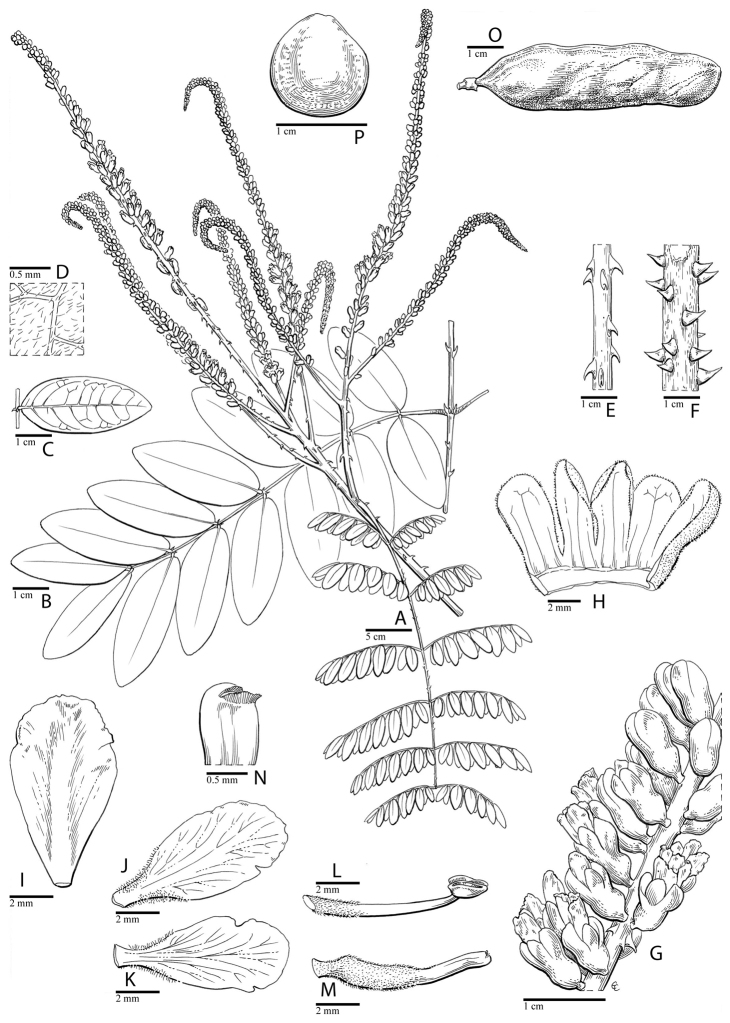
*Moullava spicata* (Dalzell) Nicolson. **A** flowering branch **B** single pinna of bipinnate leaf **C** leaflet undersurface **D** leaflet undersurface detail **E** young stem **F** older stem **G** part inflorescence **H** calyx opened out **I** median petal **J** upper lateral petal **K** lower lateral petal **L** stamen **M** gynoecium **N** stigma **O** fruit **P** seed. **A**, **G** from photo by P. S. Green **B**–**D, H**–**N** from Cult. Foster Bot. Gard. F1901, specimen *Hutchinson* 2784 **E** from *Critchett* 11/79 **F** from *Nana* 5620 **O**, **P** from *Meebold* 8605. Drawn by Eleanor Catherine.

#### 12.1 Moullava digyna

Taxon classificationPlantaeFabalesLeguminosae

(Rottl.) E. Gagnon & G. P. Lewis comb. nov.

urn:lsid:ipni.org:namess:77158131-1

##### Basionym.

*Caesalpinia digyna* Rottl., Ges. Naturf. Freude Berlin Neue Schriften 4:198–200, pl. 3. 1803.

##### Type.

[S. INDIA] Marmelon (near Madras), 9 Oct 1799, *Rottler s.n.* (? B: Herb. Willdenow, K!).

*Caesalpinia gracilis* Miq., Fl. Ned. Ind. 1:110. 1855.

Type. INDIA, *Roxburgh* (n.v.).

*Caesalpinia oleosperma* Roxb., Hort. Bengal. 32. 1814.

Type. JAVA, *Horsfield 138* (holotype K!; isotype BM).

*Caesalpinia flavicans* Grah., Cat.: 5825. 1832, nom. nud.

#### 12.2 Moullava spicata

Taxon classificationPlantaeFabalesLeguminosae

(Dalzell) Nicolson, Bot. Hist. Hort. Malabaricus [K.S.Manilal]: 184. 1980

##### Basionym.

*Caesalpinia spicata* Dalzell, in Hooker’s J. Bot. Kew Gard. Misc. 3: 89 (1851).

*Wagatea spicata* Dalzell, in Hooker’s J. Bot. Kew Gard. Misc. 3: 89 (1851).

##### Type.

WESTERN INDIA, Bombay presidency.

*Caesalpinia ferox* Hohen., Pl. Ind. Or. Exs. No. 414, non Hassk.

Type. Not traced.

*Caesalpinia digyna* Graham, Cat. 60. 1839, non Rottl. 1803, nom. illeg.

*Caesalpinia mimosoides* Heyne & Wall, Numer. List n. 5837. 1831, nom. illeg., non Lam.1785.

#### 12.3 Moullava tortuosa

Taxon classificationPlantaeFabalesLeguminosae

(Roxb.) E. Gagnon & G. P. Lewis comb. nov.

urn:lsid:ipni.org:names:77158069-1

##### Basionym.

*Caesalpinia tortuosa* Roxb., Fl. Ind. (ed. 1832) 2: 365. 1832.

##### Type.

Specimen originating from SUMATRA, cultivated in the Botanic Garden of Calcutta, “Hort. Calc. E. Sumatra”, *Roxburgh s.n.* (holotype: K!).

*Caesalpinia acanthobotrya* Miq., Fl. Ned. Ind. 1(Suppl.): 108 (1860) & 293 (1861).

Type. W. SUMATRA, prov. Priaman, 1855–60, *Diepenhorst HB2240* (holotype U; isotype BO).

*Caesalpinia microphylla* Buch.-Ham ex Prain, in J. Asiat. Soc. Bengal, Pt. 2, Nat. Hist. 66: 471. 1897, non Mart. ex G. Don, 1832.

Type. INDIA, Goyalpara, 6 Aug 1908, *Wallich 5826* (K!).

Caesalpinia tortuosa var. grandifolia Craib, Fedde Repert. Spec. Nov. Reg. Veg. 12: 392. 1913.

Type. MYANMAR [Burma], Kowpok, Jan 1912, *Meebold 17208* (K!).

*Caesalpinia cinclidocarpa* Miq., in Fl. Ned. Ind 1: 110 (1855).

Type. JAVA, as for *Cinclidocarpus nitidus*, non *Caesalpinia nitida* Hassk. (1844).

*Cinclidocarpus nitidus* Zoll. & Moritzi, in Naturr-Geneesk. Arch. Ned.-Indie 3: 82 (1846).

Type. JAVA, *Zollinger 3462* (holotype L; isotypes A, BM, P).

*Caesalpinia tortuosa* Wall., Numer. List n. 5827 D. 1831, nom. nud.

#### 12.4 Moullava welwitschiana

Taxon classificationPlantaeFabalesLeguminosae

(Oliv.) E. Gagnon & G. P. Lewis comb. nov.

urn:lsid:ipni.org:names:77158070-1

##### Basionym.

*Mezoneuron welwitschianum* Oliv., Fl. Trop. Afr. 2: 261. 1871.

*Caesalpinia welwitschiana* (Oliv.) Brenan, Kew Bull. 17(2): 203. 1963.

##### Type.

ANGOLA, Cuanza Norte, Golungo Alto, *Welwitsch 608* (holotype LISU; isotypes BM, K!).

#### 13. Biancaea

Taxon classificationPlantaeFabalesLeguminosae

Tod., Nuovi Gen. Sp. Orto Palermo: 21. 1860, descr. emended E. Gagnon & G. P. Lewis

Figs 20G–J , 22

Campecia Adans. 1763; no type species designated, and no species names ever published in this genus. It is thus not possible to apply this name which is rejected against Biancaea.
Caesalpinia sect. Sappania DC. 1825.

##### Diagnosis.

*Biancaea* is closely related to *Mezoneuron*, differing principally in its fruit, a coriaceous, laterally compressed, wingless, dehiscent pod (except *Biancaea decapetala*, which has somewhat inflated, boat-shaped pods, often with a narrow wing or ridge along the upper suture). In contrast, *Mezoneuron* has chartaceous, coriaceous or ligneous pods, which are also laterally compressed, but indehiscent, and with a wing along the upper suture. In addition, the ovary of *Biancaea* species always has a velvety indumentum (vs. glabrous to pubescent in *Mezoneuron*).

##### Type.

*Biancaea scandens* Tod. ≡ *Biancaea decapetala* (Roth) Deg.

##### Emended description.

Lianas, climbing or trailing shrubs (1–3 m), or small trees (2.5–10 m), armed with short, slightly recurved prickles, scattered along the branches; young shoots pubescent or glabrescent. Stipules lanceolate-oblong to broadly-ovate, sometimes amplexicaul at base, 3–4 mm to 4.5 cm long, caducous or sub-persistent to persistent. Leaves alternate (except in *Biancaea oppositifolia*), bipinnate, ending with a pair of pinnae, rachis pubescent (glabrous in *Biancaea oppositifolia*), armed with pairs of prickles at the base of each pinna, sometimes also scattered on the rachis; pinnae in 4–19 opposite to alternate pairs; leaflets opposite to alternate, in 5–20 pairs per pinna, blade membranous, eglandular, glabrous to pubescent, 10–35 × 4–15 mm (4–10 × 1.5–4.5 cm in *Biancaea oppositifolia*), oblong-elliptic, apex acute, obtuse, rounded to emarginate, base asymmetric. Inflorescences erect, showy, terminal or axillary racemes or panicles; rachis eglandular, pubescent, unarmed or with a few scattered prickles, mainly near the base; bracts ovate-lanceolate, acuminate, 2–8 mm long, caducous. Flowers bisexual, zygomorphic; calyx with a short hypanthium and 5 sepals, the lower sepal cucullate and covering the other 4 in bud, sepals pubescent (except in *Biancaea sappan*), caducous, but the hypanthium persisting as a calyx ring around the pedicel as fruits mature; petals 5, free, yellow to white, eglandular, the claws pubescent; the median petal smaller than the other 4, and inrolled towards the centre, lateral petals oblong, obovate to spathulate, 4–10 × 2–8 mm; stamens 10, filaments densely pubescent (most evident at the base), eglandular, 10–15 mm long; ovary densely velutinous. Fruit a coriaceous, glabrous, eglandular, oblong-elliptic to obovate, dehiscent, wingless, laterally compressed (but somewhat inflated and often with a narrow wing along the upper suture in *Biancaea decaptala*), 4.5–10 × 2–4 cm, 2–8-seeded pod, usually much broader at the rounded to truncate apex, which terminates in a sharp beak. Seeds flat, elliptic, ovoid to orbicular, c. 2 cm in diameter, black or brown.

##### Geographic distribution.

A genus of six species widespread across southern Asia, from India, to Myanmar (Burma), Thailand, Cambodia, Vietnam, south China, Japan, the Philippines, and the Malay Peninsula and Archipelago, one species endemic to Sabah (near Sandakan). *Biancaea decapetala*, native to Asia, has been widely introduced across the tropics as a hedge plant or ornamental and is considered to be invasive in South Africa and Hawaii.

##### Habitat.

Primary forest and forest margins, grasslands, scrub vegetation, riverine habitats, secondary thickets and clearings. From the coast to mountain slopes.

##### Etymology.

Unknown.

##### Notes.

Based on the study of Gagnon et al. (2013), Molinari-Novoa et al. (2016) provided some, but not all, of the required nomenclatural transfers to the genus *Biancaea*. Furthermore, they did not emend the description of the genus, as provided here.

##### References.

Hattink (1974); Vidal and Hul Thol (1976); Jansen (2005); Brummitt et al. (2007); Chen et al. (2010a); Molinari-Novoa et al. (2016).

**Figure 22.**
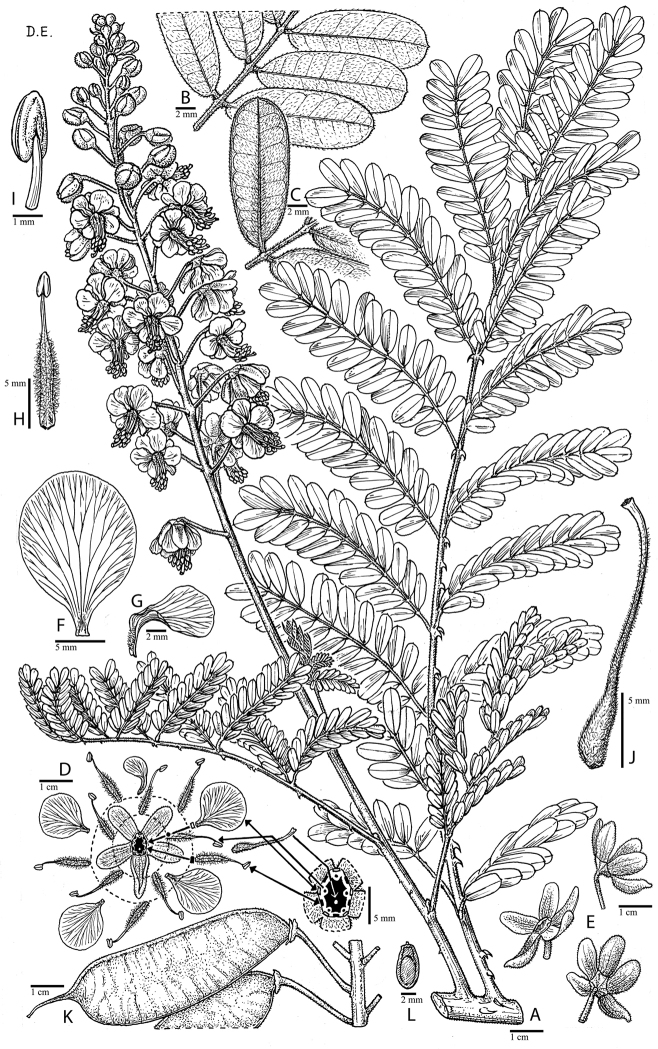
*Biancaea decapetala* (Roth) O. Deg. **A** flowering branchlet and foliage **B, C** leaflets viewed from above and below, respectively **D** flower with parts separated, and centre of flower enlarged **E** calyx three views **F** lateral petal **G** median petal **H** stamen **I** anther **J** gynoecium **K** fruits **L** seed. **A** from *Rutherford-Smith* 11062 **B, C** from *White* 2478 **D–J** from *Chase* 4564 **K**, **L** from *Myre* 2528. Drawn by D. Erasmus, originally published in *Flora Zambesiaca*, vol. 3 part 2, page 182, figure 3.2.39 (2007).

#### 13.1 Biancaea decapetala

Taxon classificationPlantaeFabalesLeguminosae

(Roth) O. Deg., Fl. Hawaiiensis K7. 1936

##### Basionym.

*Reichardia decapetala* Roth, Nov. Pl. Sp. 212. 1821.

*Caesalpinia decapetala* (Roth) Alston, Handb. Fl. Ceylon 6: 89. 1931.

**Type.** INDIA, (fl.), *Heyne s.n.* (isotype K!).

*Biancaea scandens* Tod., in Nuov. Gen. Sp. Pl.: 22. 1860.

Type. “Cortivasi da lungo tempo nel Real Orto Botanico [di Palermo] in piena terra, col nome di *Caesalpinia sepiaria*”.

*Caesalpinia benguetensis* Elmer, in Leafl. Philipp. Bot. 1: 226 (1907).

*Mezoneuron benguetense* (Elmer) Elmer, in Leafl. Philipp Bot 1: 362 (1908).

Type. PHILIPPINES, Luzon, Benguet prov. Baguio, (fl. fr.), Mar 1907, *Elmer 8720* (BO, K!, L, PHN).

*Caesalpinia japonica* Sieb. & Zucc., in Abh. Math.-Phys. Cl. Königl. Bayer Akad. Wiss. 4(2): 117. 1845.

Caesalpinia sepiaria var. japonica (Siebold & Zucc.) Gagnep., in Fl. Indo-Chine 2: 180. 1913.

Caesalpinia sepiaria var. japonica (Siebold & Zucc.) Makino, Ill. Fl. Nippon: 431. 1940.

Caesalpinia decapetala var. japonica (Siebold & Zucc.) H. Ohashi, Fl. E. Himalaya 3: 58. 1975.

Caesalpinia decapetala var. japonica (Siebold & Zucc.) Isely, Mem. New York Bot. Gard. 24(2): 193. 1975.

Type. JAPAN, *Siebold & Zuccanini*.

*Caesalpinia ferox* Hassk., Ind. Sem. Hort. Amst. 1841.

*Biancaea ferox* (Hassk.) Tod., Hort. Bot. Panorm. 1(1): 3. 1875.

Type. probably a living plant in Hort. Bog., fide Hattink (1974).

*Caesalpinia sepiaria* Roxb., Fl. Ind. 2: 360. 1832. *Biancaea sepiaria* (Roxb.) Tod., Hort. Bot. Panorm. 1(1): 3. 1875.

Type. INDIA, *Roxburgh* without number (isotypes: BM, K!, in *Hb. Wallich* 5834A).

Caesalpinia sepiaria Roxb. var. pubescens T. Tang. & F.T. Wang, Illust. Treat. Prin. Pl. China (Leguminosae): 96. 1955, without Latin description.

Caesalpinia sepiaria Roxb. var. pubescens T. Tang & F. T. Wang ex C. W. Chang, Flora Tsinlingensis 1(3): 444. 1981.

Caesalpinia decapetala (Roth) Alston var. pubescens P. C. Huang, Sylva Sinica 2: 1187. 1985, nom. illeg., without Latin description or type.

Caesalpinia decapetala var. pubescens (T. Tang & F. T. Wang ex C. W. Chang) X. Y. Zhu, in Legumes of China: 5. 2007.

Type. CHINA.

#### 13.2 Biancaea godefroyana

Taxon classificationPlantaeFabalesLeguminosae

(Kuntze) Molinari, Mayta & Sánchez Och., Weberbauerella 1(11): 3. 2016

##### Basionym.

*Caesalpinia godefroyana* Kuntze, Rev. Gen. Pl. 1: 166. 1891.

##### Type.

VIETNAM (South), Cap St-Jacques (Vung Tau), 18 Mar 1875, *Godefroy s.n.* (lectotype K!, designated by Vidal and Hul Thol, 1976).

*Caesalpinia thorelii* Gagnep., Notul. Syst. (Paris). 2: 207. 1912.

Types. VIETNAM, 1^er^ pont de l’avalanche près Saïgon, 14 Jan 1865, *Lefèvre, Thorel et Godefroy no.* 145 (syntype P02940578!); Cochinchine, Bien-hoa, Nov 1866, *Thorel 848* (syntype P02940348!); ad Bienhoa, *Pierre 130* (syntype P02940353); Cochinchine, Baria, *Baudoin and Talmy 104* (syntype);

#### 13.3 Biancaea millettii

Taxon classificationPlantaeFabalesLeguminosae

(Hook. & Arn.) E. Gagnon & G. P. Lewis comb. nov.

urn:lsid:ipni.org:names:77158071-1

##### Basionym.

*Caesalpinia millettii* Hook. & Arn., Bot. Beechey Voy. 182 (1841[1833]).

##### Type.

CHINA, *Millett s.n.* (K!).

*Pterolobium subvestitum* Hance, J. Bot. 22(12): 365. 1884.

*Cantuffa subvestita* (Hance) Kuntze, Rev. Gen. Pl. 1: 168. 1891.

Type. CHINA, Kwangtung, Lo Fau Sahn, *Faber in herb. Hance 22291* (BM).

#### 13.4 Biancaea oppositifolia

Taxon classificationPlantaeFabalesLeguminosae

(Hattink) Molinari & Mayta, Weberbauerella 1(11): 3. 2016

##### Basionym.

*Caesalpinia oppositifolia* Hattink, Reinwardtia 9(1): 43. 1974.

##### Type.

MALESIA, Sabah [North Borneo], Ranau Distr. Hot Spring track, 15 Feb 1961, *J. Singh 24026* (holotype SAN; isotypes K!, L).

#### 13.5 Biancaea parviflora

Taxon classificationPlantaeFabalesLeguminosae

(Prain ex King) Mayta & Molinari, Weberbauerella 1(11): 3. 2016

##### Basionym.

*Caesalpinia parviflora* Prain ex King, J. Asiat. Soc. Bengal, Pt. 2, Nat. Hist. 66: 230. 1897.

##### Type.

MALAY PENINSULA, Perak, Relau Tugor, May 1888, *Wray 1909* (lectotype CAL, designated by Hattink 1974; isolectotypes K!, SING).

Caesalpinia parviflora var. stipularis Prain, in J. Asiat. Soc. Bengal, Pt. 2, Nat. Hist. 66: 230. 1897.

Types. MALAY PENINSULA, Perak, Larut, *Wray 3983, 3991, 4261* (syntypes).

*Caesalpinia stipularis* Ridl., in Fl. Malay Penin. 1: 651 (1922), nom. illeg., non *Caesalpinia stipularis* (Vogel) Benth. (1870) (= *Pomaria stipularis* (Vogel) B.B. Simpson & G. P. Lewis).

Caesalpinia parviflora var. typica (Prain ex King) Prain, J. Asiat. Soc. Bengal, Pt. 2, Nat. Hist. 60: 230. 1897, nom. illeg.

*Caesalpinia borneensis* Merr., Univ. Calif. Publ. Bot. 15: 104. 1929.

Type. BORNEO, Tawao, Elphinstone Prov., Oct 1922– Mar 1923, *Elmer 21449* (holotype MO; isotypes A, BM, BO, K!, L, NY, P, SING, U, UC).

*Caesalpinia macra* Craib, Bull. Misc. Inform. Kew 2: 386. 1927.

Type. THAILAND, Saraburi, Muak Lek, 10 Nov 1924, *Marcan 1866* (syntype K), Pak Chong, 30 Dec 1923, *Marcan 1532* (syntype K).

*Caesalpinia minutiflora* Elmer, Leafl. Philipp. Bot. 5: 1803. 1913.

Type. PHILIPPINES, Palawan, Puerto Princesa, Mt. Pulgar, Apr 1911, *Elmer 12969* (BM, K!, L, P, PNH, U).

#### 13.6 Biancaea sappan

Taxon classificationPlantaeFabalesLeguminosae

(L.) Tod., Hort. Bot. Panorm. 1(1): 3. 1875

##### Basionym.

*Caesalpinia sappan* L., Sp. Pl. 1: 381. 1753.

##### Type.

SRI LANKA (CEYLON), *Hb. Hermann*, vol. 4, fol. 31 (holotype BM).

*Caesalpinia angustifolia* Salisb., Prod.: 326. 1796, nom. illeg.

#### 14. Pterolobium

Taxon classificationPlantaeFabalesLeguminosae

R. Br. ex Wight & Arn., Prodr: 283. 1834

Figs 23 , 24A–C

Cantuffa J.F. Gmel. (1791).
Reichardia Roth (1821), nom. illeg., non Roth (1787), nec Roth (1800).

##### Type.

*Pterolobium lacerans* R. Br. ex Wight & Arn., nom. illeg. (*Cantuffa exosa* J.F. Gmel. = *Pterolobium exosum* (J.F. Gmel.) E.G. Baker; this now considered a synonym of *Pterolobium stellatum* (Forssk.) Brenan).

##### Description.

Lianas or scrambling / trailing shrubs, armed with prickles on shoots, as well as in pairs at the base of leaves. Stipules small, inconspicuous, subulate or triangular-subulate, caducous. Leaves alternate, bipinnate, ending in a pair of pinnae, 6–30 cm long; petiole and rachis pubescent to sparsely pubescent or glabrous; pinnae opposite, in 5–20 pairs; leaflets opposite, in 6–25 pairs per pinna, linear-oblong to elliptic-oblong, apex rounded to emarginate, sometimes mucronate, eglandular or punctate-glandular, 6–15 × 1.5–10 mm. Inflorescences terminal or axillary racemes, often aggregated into panicles, pubescent to glabrous, 4–25 cm long; bracts small, caducous. Flowers bisexual, sub-actinomorphic to zygormophic; calyx comprising a short hypanthium and 5 sepals, glabrous to pubescent, the lower sepal cucullate, covering the other 4 sepals in bud; petals 5, free, yellow to white, equal to slightly differentiated, claws pubescent, the median petal sometimes inrolled; stamens 10, free, filaments pubescent (occasionally glabrous); ovary pubescent, stigma chambered. Fruit a red to brown samara, the basal seed-containing portion 12–20 × 8–15 mm, reticulate or smooth, glabrous to pubescent, the upper suture much prolonged and broadly winged, the wing 20–45 mm long and usually wider distally, 1 (–2)-seeded.

##### Geographic distribution.

A genus of 10 species; one in southern tropical Africa, East Africa and Arabia, nine in SE Asia (one endemic to India, two in China, four in Indo-China [one endemic to Thailand, two extending to Malesia], three restricted to the Malay Peninsula and Archipelago [one endemic to the Philippines]).

##### Habitat.

Seasonally dry tropical upland evergreen forest, riverine and humid forest, woodland and wooded grassland.

##### Etymology.

From *ptero*- (Greek: wing) and *lobion* (Greek: pod, fruit), in reference to the fruit which is a samara.

##### Notes.

Vidal and Hul Thol (1974) published a revision of *Pterolobium*, with a key to species. We provide below a list of species currently accepted in the genus, taking into account the treatment of *Pterolobium sinense* as a synonym of *Pterolobium macropterum* (Chen et al. 2010b).

##### References.

Roti-Michelozzi (1957); Brenan (1967: 40–42); Vidal and Hul Thol (1974, 1976); Hul Thol and Hideux (1977); Hou et al. (1996: 654–700); Chen et al. (2010b).

**Figure 23.**
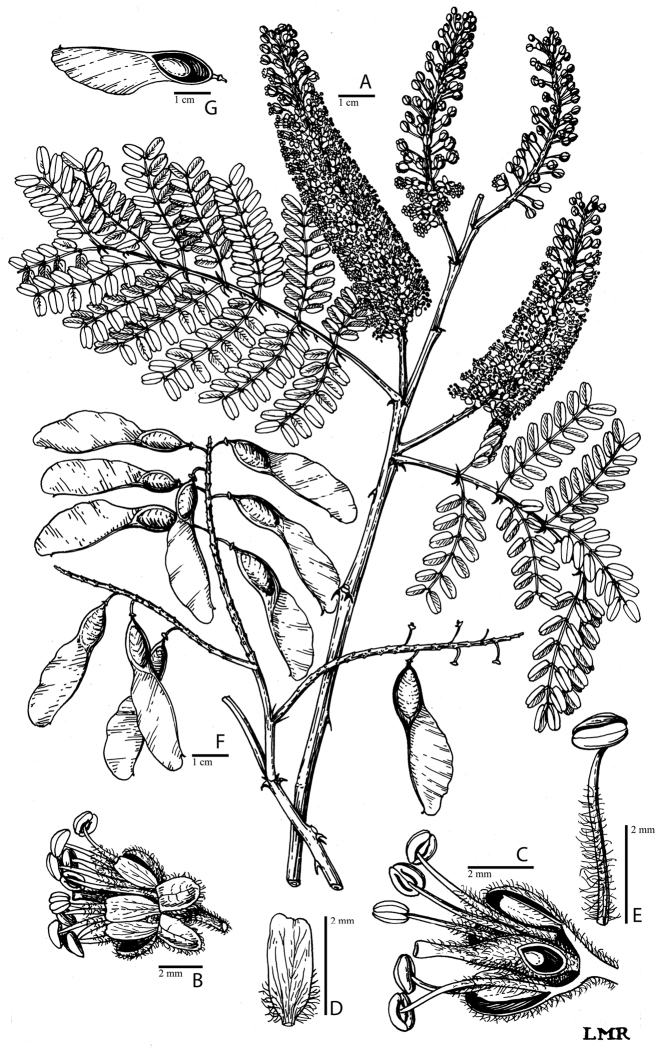
*Pterolobium stellatum* (Forssk.) Brenan. **A** part of flowering branch **B** flower **C** longitudinal section of flower **D** petal **E** stamen **F** infructescence with mature fruits **G** samara with part cut away to reveal seed. **A–E** from *Richards* 11275 **F** from *Eggeling* 3400 **G** from *Sandwith* 25. Drawn by L. M. Ripley, originally published in *Flora of Tropical East Africa, Leguminosae subfamily Caesalpinioideae*, page 41, fig. 7 (1967).

**Figure 24.**
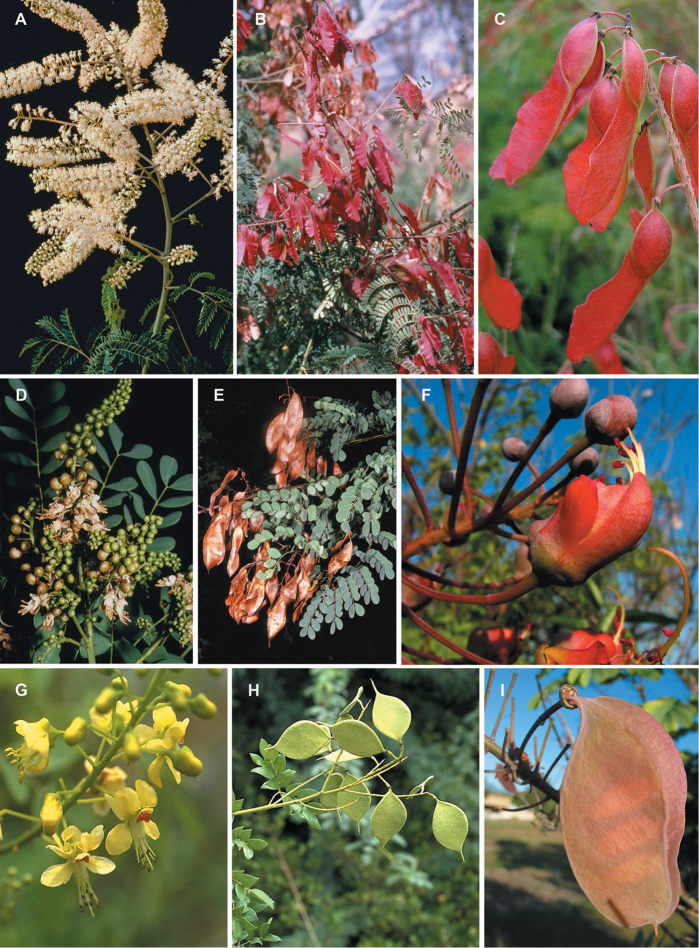
*Pterolobium stellatum* (Forssk.) Brenan. **A** inflorescences (P. van Wyk, Africa, *unvouchered*) **B** fruits (J. Anton-Smith, Africa, *unvouchered*) **C** close up of fruits (B. T. Wursten, Flora of Zimbabwe (http://www.zimbabweflora.co.zw/speciesdata/image-display.php?species_id=127190&image_id=1), Zimbabwe, *unvouchered*). *Mezoneuron hildebrandtii* Vatke **D** inflorescences (D. Du Puy, Majunga, Madagascar, *Du Puy M286* (P)) **E** fruits (D. Du Puy, Antsiranana, Madagascar, *Du Puy M273* (P)). *Mezoneuron kauaiense* (H. Mann) Hillebr. **F** flower and buds **I** fruit (D. Eickhoff, Wikicommons (https://commons.wikimedia.org/wiki/Category:Mezonevron_kavaiense) cultivated, Hawaii, U.S.A., *unvouchered*). *Caesalpinia crista* L. emend. Dandy & Exell (?*Ticanto*) **G** flowers **H** young fruits (P. Grard: Institut Français de Pondichéry, Andhra Pradesh, India, *unvouchered*).

#### 14.1 Pterolobium borneense

Taxon classificationPlantaeFabalesLeguminosae

Merrill

#### 14.2 Pterolobium densiflorum

Taxon classificationPlantaeFabalesLeguminosae

Prain

#### 14.3 Pterolobium hexapetalum

Taxon classificationPlantaeFabalesLeguminosae

(Roth) Santapau & Wagh

#### 14.4 Pterolobium integrum

Taxon classificationPlantaeFabalesLeguminosae

Craib

#### 14.5 Pterolobium macropterum

Taxon classificationPlantaeFabalesLeguminosae

Kurz

#### 14.6 Pterolobium membranulaceum

Taxon classificationPlantaeFabalesLeguminosae

(Blanco) Merrill

#### 14.7 Pterolobium micranthum

Taxon classificationPlantaeFabalesLeguminosae

Gagnep., emend. Craib

#### 14.8 Pterolobium microphyllum

Taxon classificationPlantaeFabalesLeguminosae

Miq.

#### 14.9 Pterolobium punctatum

Taxon classificationPlantaeFabalesLeguminosae

Hemsl.

#### 14.10 Pterolobium stellatum

Taxon classificationPlantaeFabalesLeguminosae

(Forssk.) Brenan

#### 15. Mezoneuron

Taxon classificationPlantaeFabalesLeguminosae

Desf., Mém. Mus. Hist. Nat. 4: 245. 1818

Figs 24D–F, I , 25

Mezonevron Desf. and Mezoneurum DC. (1825), (orth. vars.).
Caesalpinia subg. Mezoneuron (Desf.) Vidal ex Herend. & Zarucchi (1990).

##### Type.

*Mezoneuron glabrum* Desf. ≡ *Mezoneuron pubescens* Desf.

##### Description.

Scrambling shrubs or lianas, occasionally medium -sized trees (*Mezoneuron kauaiense*) to 12 m, usually armed with recurved prickles on stem and leaves, rarely unarmed. Stipules very small, often caducous. Leaves alternate or occasionally opposite, bipinnate, ending in a pair of pinnae; pinnae opposite to sub-opposite, in (1–)2–18 pairs; leaflets opposite to alternate, in 1–15 pairs per pinna, elliptic, oblong, suborbicular to occasionally subrhombic, the base oblique, the apex obtuse to acute. Inflorescences terminal or axillary racemes (often aggregated into panicles); bracteoles small. Flowers bisexual, zygomorphic; calyx comprising a hypanthium and 5 imbricate sepals, the lower sepal cucullate, and overlapping the other 4 in bud; petals 5, free, usually yellow with red markings on the median petal, or occasionally red, pink or cream, the median petal somewhat modified (either with a fleshy ligule or a patch of hairs on the inner surface between the blade and claw, or the petal bilobed); stamens 10, free, filaments alternately longer and shorter, usually all 10 pubescent or villous on lower half, or one or all glabrous; ovary glabrous to hairy, 1-many ovuled, stigma cupular, funnel-shaped, terminal or laterally placed, glabrous, or the rim fimbriate with papillate hairs, not peltate. Fruit laterally compressed, indehiscent, chartaceous, coriaceous or woody, venose, longitudinally and often broadly winged along the upper suture, the wing 1–18 mm wide. Seeds 1–13 per pod, ± transversely arranged in seed chamber, compressed, endosperm lacking.

##### Geographic distribution.

A genus of 24 extant species, mainly in Asia, extending to Australia, Polynesia, Madagascar and Africa; two species on mainland Africa (one widespread in West Africa, the other in both West, East and Southeast Africa); one endemic to Madagascar; five endemic to New Caledonia; one endemic in Hawaii; one in Vietnam; four endemic to Australia (Queensland and New South Wales); one endemic in the Philippines; one in Australia and Papua New Guinea; nine species more widespread across Asia.

##### Habitat.

Tropical and subtropical riverine forest, lowland rain forest, swamp forest, seasonally dry forest, thicket, vine forest and wooded grassland, especially along forest and river margins.

##### Etymology.

From *meso*- (Greek: middle) or *meizon* (Greek: greater) and *neuron* (Greek: nerve), the upper suture of the fruit is bordered by a usually broad longitudinal wing so that the suture appears as a prominent sub-central nerve or vein.

##### Notes.

The genus has recently been revised by Clark (2016), who provides full synonymy, a key to species, and a list of fossil taxa associated with this genus.

##### References.

Brenan (1967: 38–40); Hattink (1974); Vidal and Hul Thol (1976); Verdcourt (1979: 18–20); Lock (1989: 25); Herendeen and Zarucchi (1990); Pedley (1997); George (1998: 59–67); Wagner et al. (1999); Du Puy and Rabevohitra (2002: 48–49); Brummitt et al. (2007); Clark and Gagnon (2015); Clark (2016).

**Figure 25.**
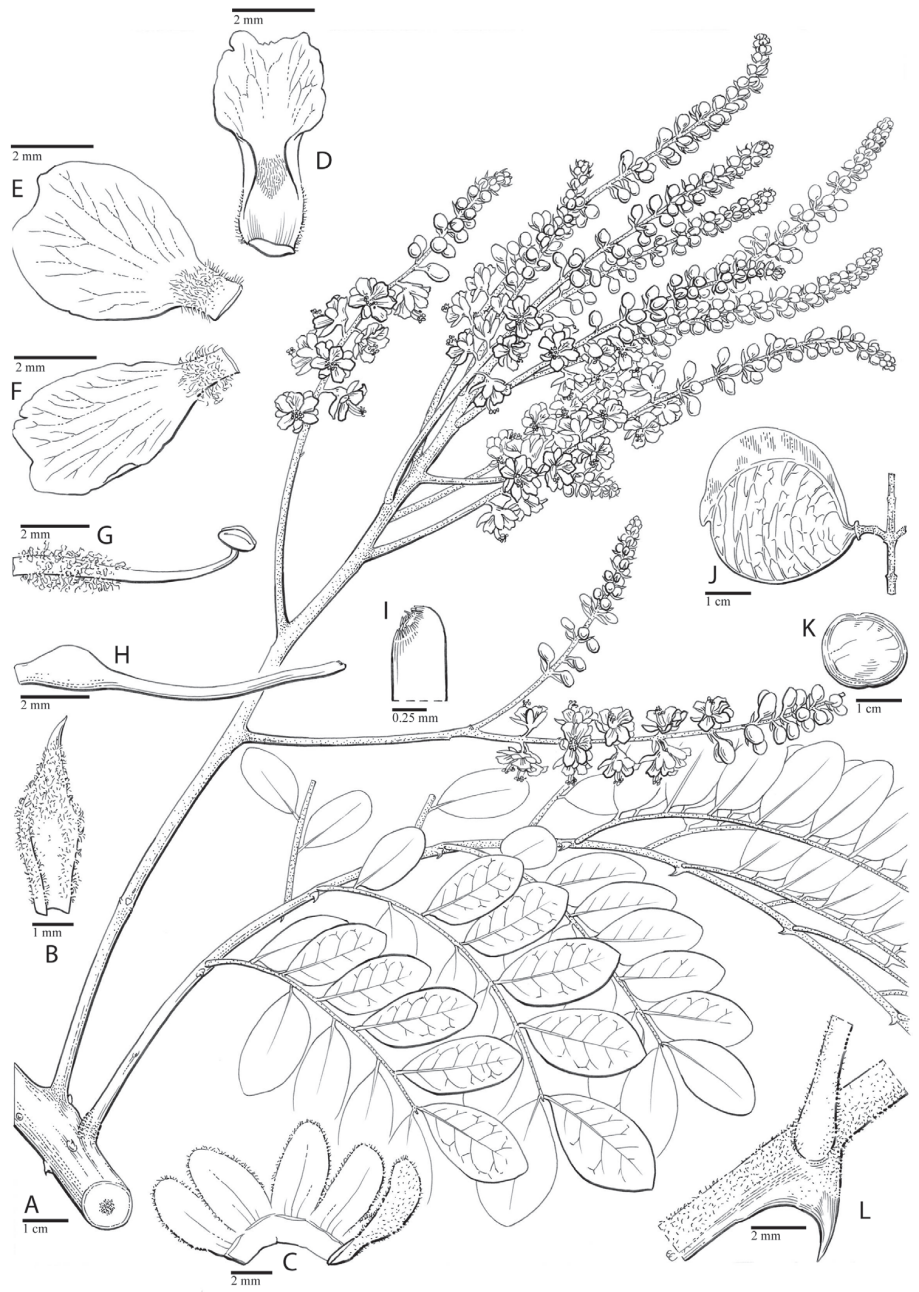
*Mezoneuron scortechinii* F. Muell. **A** flowering branch **B** bract **C** calyx opened out **D** median petal **E** upper lateral petal **F** lower lateral petal **G** stamen **H** gynoecium **I** stigma **J** fruit **K** seed **L** detail of prickle from leaf. **A–I, L** from *Hoogland* 11665 **J** from *Thurtill & Coveny* 3880 **K** from *White* s.n. 6/1926. Drawn by Eleanor Catherine.

#### 15.1 Mezoneuron andamanicum

Taxon classificationPlantaeFabalesLeguminosae

Prain

#### 15.2 Mezoneuron angolense

Taxon classificationPlantaeFabalesLeguminosae

Welw. ex Oliv.

#### 15.3 Mezoneuron baudouinii

Taxon classificationPlantaeFabalesLeguminosae

Guillaumin

#### 15.4 Mezoneuron benthamianum

Taxon classificationPlantaeFabalesLeguminosae

Baill.

#### 15.5 Mezoneuron brachycarpum

Taxon classificationPlantaeFabalesLeguminosae

Benth.

#### 15.6 Mezoneuron cucullatum

Taxon classificationPlantaeFabalesLeguminosae

(Roxb.) Wight & Arn.

#### 15.7 Mezoneuron enneaphyllum

Taxon classificationPlantaeFabalesLeguminosae

(Roxb.) Wight & Arn. ex Voigt

#### 15.8 Mezoneuron erythrocarpum

Taxon classificationPlantaeFabalesLeguminosae

(Pedley) R. Clark & E. Gagnon

#### 15.9 Mezoneuron furfuraceum

Taxon classificationPlantaeFabalesLeguminosae

Prain

#### 15.10 Mezoneuron hildebrandtii

Taxon classificationPlantaeFabalesLeguminosae

Vatke

#### 15.11 Mezoneuron hymenocarpum

Taxon classificationPlantaeFabalesLeguminosae

Wight & Arn. ex Prain

#### 15.12 Mezoneuron kauaiense

Taxon classificationPlantaeFabalesLeguminosae

(H. Mann) Hillebr.

#### 15.13 Mezoneuron latisiliquum

Taxon classificationPlantaeFabalesLeguminosae

(Cav.) Merr.

#### 15.14 Mezoneuron mindorense

Taxon classificationPlantaeFabalesLeguminosae

Merr.

#### 15.15 Mezoneuron montrouzieri

Taxon classificationPlantaeFabalesLeguminosae

Guillaumin

#### 15.16 Mezoneuron nhatrangense

Taxon classificationPlantaeFabalesLeguminosae

Gagnep.

#### 15.17 Mezoneuron nitens

Taxon classificationPlantaeFabalesLeguminosae

(F. Muell. ex Benth.) R. Clark & E. Gagnon

#### 15.18 Mezoneuron ouenensis

Taxon classificationPlantaeFabalesLeguminosae

(Guillaumin) R. Clark

#### 15.19 Mezoneuron pubescens

Taxon classificationPlantaeFabalesLeguminosae

Desf.

#### 15.20 Mezoneuron rubiginosum

Taxon classificationPlantaeFabalesLeguminosae

(Guillaumin) R. Clark

#### 15.21 Mezoneuron sinense

Taxon classificationPlantaeFabalesLeguminosae

Hemsl.

#### 15.22 Mezoneuron schlechteri

Taxon classificationPlantaeFabalesLeguminosae

(Harms) R. Clark

#### 15.23 Mezoneuron scortechinii

Taxon classificationPlantaeFabalesLeguminosae

F. Muell.

#### 15.24 Mezoneuron sumatranum

Taxon classificationPlantaeFabalesLeguminosae

(Roxb.) Wight & Arn.

#### Fossil taxa

##### 15.25 Mezoneuron claibornensis

Taxon classificationPlantaeFabalesLeguminosae

(Herendeen & Dilcher) R. Clark & E. Gagnon

##### 15.26 Mezoneruon flumen-viridensis

Taxon classificationPlantaeFabalesLeguminosae

(Herendeen & Dilcher) R. Clark & E. Gagnon

##### 15.27 Mezoneuron spokanensis

Taxon classificationPlantaeFabalesLeguminosae

(Knowlton) R. Clark & E. Gagnon

#### 16. Cordeauxia

Taxon classificationPlantaeFabalesLeguminosae

Hemsl., Bull. Misc. Inform. Kew 1907: 361. 1907

Figs 26 , 27A–E

##### Type.

*Cordeauxia edulis* Hemsl.

##### Description.

Evergreen shrubs, multi-stemmed, to 4 m tall, unarmed, red gland dots on stems. Leaves alternate, pinnate; leaflets in (1–) 2–4 (– 6) pairs per leaf, ovate-oblong, coriaceous, with conspicuous red glands on the lower surface, elliptic-oblong, up to 3 (– 5) × 1.5 (– 2.5) cm. Inflorescence a terminal, few-flowered raceme. Flowers bisexual, sub-actinomorphic; sepals c. 1 cm long, with red gland dots; petals 5, free, yellow, c. 1.5 cm long, clawed; stamens 10, free, filaments pubescent; ovary with red gland dots. Fruit a compressed-ovoid, ligneous, dehiscent pod, 4–6 × 2 cm, with very hard, thick valves, and a cornute beak, 1–4-seeded. Seeds ovoid, 20–45 mm long.

##### Geographic distribution.

A monospecific genus from NE Africa (Somalia and Ethiopia). Introduced in Israel, Kenya, Sudan, Tanzania, and Yemen (Orwa et al. 2009).

##### Habitat.

Seasonally dry tropical (semi-desert) bushland and thicket on sand.

##### Etymology.

Named by Hemsley for Captain H. E. S. Cordeaux (1870–1943), one time H. M. Commissioner in Somalia.

##### References.

Roti-Michelozzi (1957); Thulin (1983: 20–21; 1993: 348); Brink (2006).

**Figure 26.**
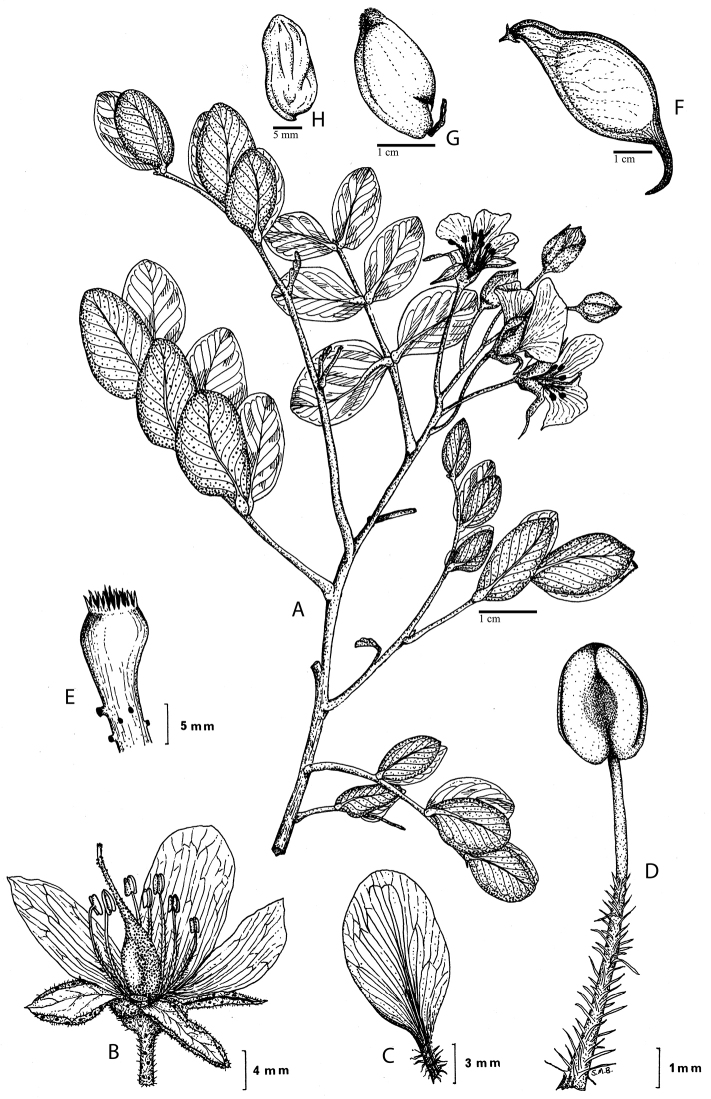
*Cordeauxia edulis* Hemsl. **A** branch with foliage and flowers **B** flower **C** petal **D** stamen **E** stigma **F** fruit **G** seed **H** seed with testa removed. **A, C–E** from *Thulin & Warfa* 4610 **B** from *Hemming* 375 **F** from *Wood* 2184 **G, H** from *Cordeaux* s.n. (type). Drawn by unknown artist.

**Figure 27.**
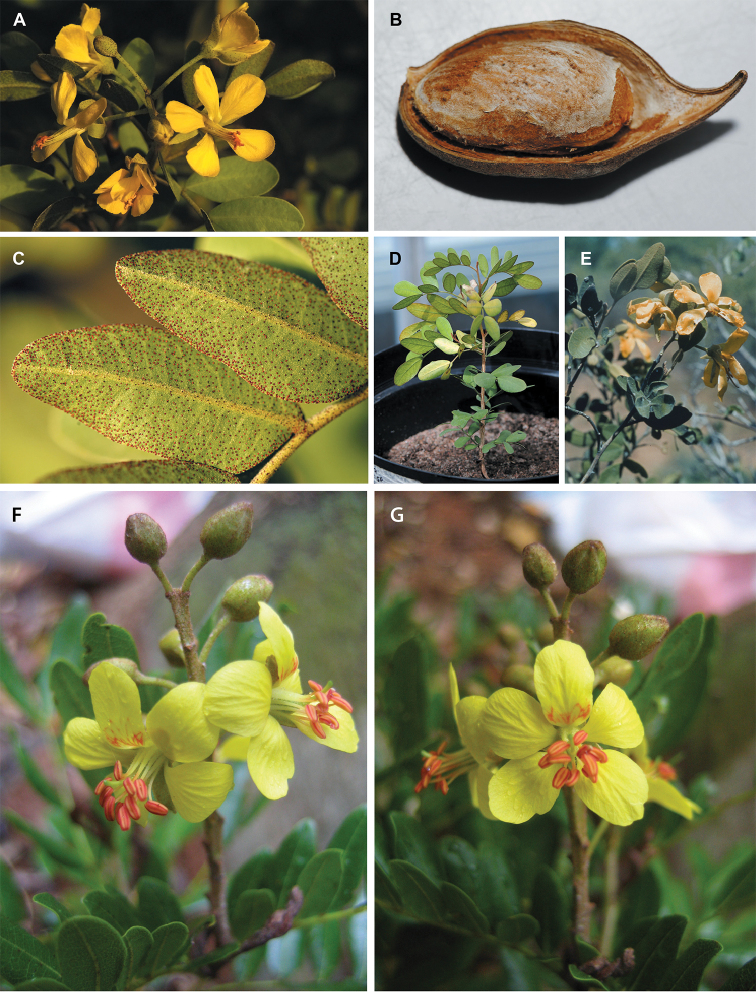
*Cordeauxia edulis* Hemsl. **A** inflorescence **B** open fruit with seed **C** undersurface of leaflets showing glands **D** young seedling (Jarmo Holopainen, cultivated plants in Sweden and Finland, *unvouchered*) **E** branch with flowers (M. Thulin, Somalia, *unvouchered*). *Stuhlmannia moavi* Taub. **F, G** inflorescence (R. Randrianaivo, Madagascar, *Radrianaivo 1486* (MO, TAN)).

#### 16.1 Cordeauxia edulis

Taxon classificationPlantaeFabalesLeguminosae

Hemsl.

#### 17. Stuhlmannia

Taxon classificationPlantaeFabalesLeguminosae

Taub., Engler, Pflanzenw. Ost.-Afr. C: 201. 1895

Figs 27F–G , 28

##### Type.

*Stuhlmannia moavi* Taub.

##### Description.

Unarmed trees, to 25 m tall; bark brown, fissured and fibrous; young shoots eglandular or with small red glands. Stipules not seen. Leaves alternate, pinnate or bipinnate and then ending in a pair of pinnae, (1.5–) 5–11 (– 20 cm) long, pinnae in (1–) 2–10 pairs per leaf, with reddish glands; leaflets in 3–12 pairs per pinna, opposite to sub-opposite, elliptic, 7–75 (– 120) × 3–30 (– 60) mm, obtuse at the base and apex, glabrous, eglandular or with red glands on the lower surface. Inflorescence a 2–11 cm long, terminal or axillary raceme; pedicels 3–13 mm long. Flowers bisexual, sub-actinomorphic; calyx comprising a hypanthium and 5 sepals, these 5–6.5 mm long, valvate in bud, caducous; petals 5, free, yellow, the median petal with red markings, obovate, 9–12 × 3–6 mm, apex rounded, median petal slightly smaller than the others; stamens 10, free, 5.5–8 mm long, filaments pubescent; ovary stipitate, with red sessile glands, glabrous to pubescent. Fruit a flattened, oblong, woody, elliptic pod with an acuminate apex, 4.5–6 × 1.5–2 cm, dehiscing along both sutures, valves twisting, glabrous to thinly puberulous. Seeds flattened, sub-circular to ovate, c. 10–13 × 8–9 mm, brown.

##### Geographic distribution.

A monospecific genus in E Africa (Kenya and Tanzania) and N Madagascar.

##### Habitat.

Seasonally dry tropical forest, woodland on limestone and in riverine forest.

##### Etymology.

Named by Taubert for the German naturalist Franz Ludwig Stuhlmann (1863–1928).

##### References.

Brenan (1967: 45–47); Capuron (1967, under *Caesalpinia insolita*); Lewis (1996); Du Puy and Rabevohitra (2002: 48, 50, under *Caesalpinia insolita*); Lemmens (2010).

**Figure 28.**
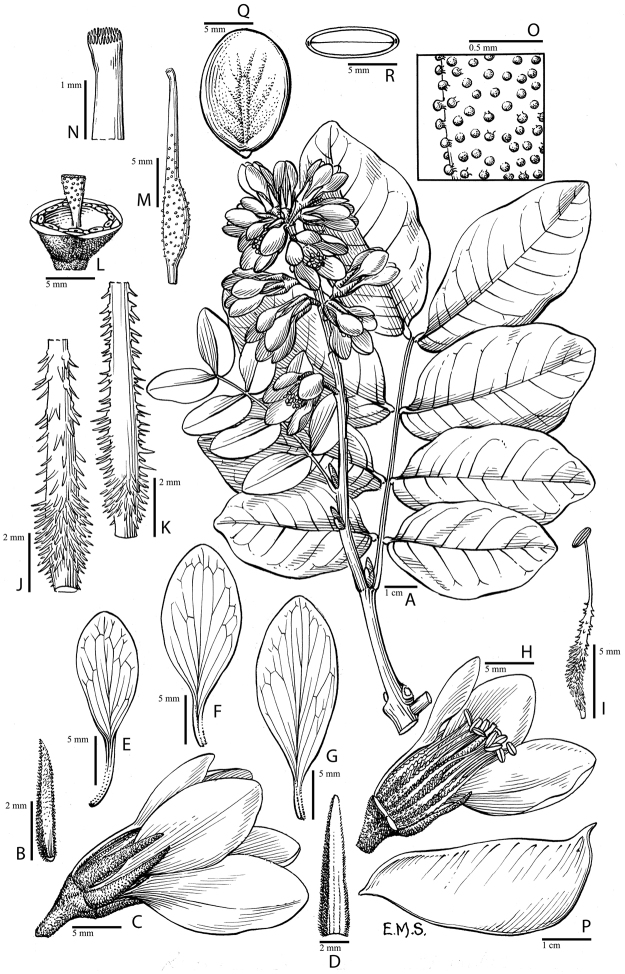
*Stuhlmannia moavi* Taub. **A** inflorescence and pinnate leaf **B** flower bract **C** flower **D** sepal **E** median petal **F** upper lateral petal **G** lower lateral petal **H** flower with sepals and petals removed from one side to show arrangement of stamens **I** stamen **J** lower portion of stamen filament, seen from inside the flower **K** lower portion of stamen filament seen from outside the flower **L** hypathium after fall of sepals, petals and stamens **M** gynoecium, **N** stigma and apical portion of style **O** detail of outer surface of ovary showing sessile glands **P** fruit **Q** seed **R** transverse section of seed. **A** from *Tanner* 3167 **B, P–R** from *Tanner* 3724 **C–O** from *Tanner* 2467. Drawn by E. M. Stones, originally published in Hooker`s Icones Plantarum, Tab. 3626 (1967).

#### 17.1 Stuhlmannia moavi

Taxon classificationPlantaeFabalesLeguminosae

Taub.

Caesalpinia insolita (Harms) Brenan & Gillett
Caesalpinia dalei Brenan & Gillett

#### 18. Cenostigma

Taxon classificationPlantaeFabalesLeguminosae

Tul., Ann. Sci. Nat., Bot., sér. 2. 20: 140. 1843, descr. emended E. Gagnon & G. P. Lewis

Figs 29 , 30

Poincianella Britton & Rose. 1930, pro parte, excluding the type.

##### Diagnosis.

*Cenostigma* is morphologically most similar to the genus *Erythrostemon*. It differs from the latter by its leaves with alternate to subopposite (occasionally opposite) leaflets (vs. leaflets consistently opposite in *Erythrostemon*). A number of other characters can help to distinguish between the two genera, but these are not constant across species of *Cenostigma*. For example, a stellate indumentum on the leaflets, inflorescences, and/or sepals is found on some, but not all *Cenostigma* species, but is always lacking in *Erythrostemon*. Black subepidermal glands (visible with a × 20 lens) can be found scattered in the undersurface of leaflets and/or on sepals in *Cenostigma* (vs. these always lacking in *Erythrostemon*). *Cenostigma* pods are generally woody with thickened margins or an adaxial, proximal woody ridge or crest (vs. less robust pods lacking any woody ridge or crest in *Erythrostemon*).

##### Type.

*Cenostigma macrophyllum* Tul.

##### Emended description.

Unarmed multi-stemmed shrubs, small compact trees, (0.3–) 0.5–6 m, or large trees to 35 m tall, the larger trees with fluted trunks at maturity (*Cenostigma bracteosum*, *Cenostigma pluviosum*, *Cenostigma eriostachys*, *Cenostigma tocantinum* and *Cenostigma macrophyllum*); bark smooth, or occasionally rough and flaking (some infraspecific taxa of *Cenostigma pluviosum*), brown, grey, or mottled silver or grey; young shoots terete, glabrous to pubescent, glandular to eglandular. Stipules red, with ciliate margins, broadly ovate with a rounded apex, and caducous in *Cenostigma pyramidale*, not seen in other species. Leaves alternate, pinnate or bipinnate and then ending in a pair of pinnae plus a single terminal pinna, glabrous to densely pubescent, sometimes with stellate hairs or various types of sessile or stalked glands; petioles (0.1–) 0.6–4.8 (–6) cm, rachis 0.5–17 (– 26.5) cm; species with pinnate leaves (*Cenostigma tocantinum*, *Cenostigma marginatum*, *Cenostigma pinnatum*, and *Cenostigma macrophyllum*) either with three leaflets or 2–9 pairs of opposite leaflets; species with bipinnate leaves with 1–11 pairs of opposite to alternate pinnae, plus a terminal pinna, each pinna with 3–29 alternate to subopposite (occasionally opposite) individual leaflets; leaflets vary greatly in size, 0.5–15 × 0.1–7 cm, glossy on the upper surface, usually more or less coriaceous (chartaceous in *Cenostigma tocantinum*), ovate-elliptic, lanceolate with an acute to acuminate apex (some specimens of *Cenostigma tocantinum*), obovate, oblong-elliptic or suborbicular, apex rounded or emarginate, mucronate, base cuneate, cordate or truncate, the blade often inequilateral at the base, eglandular, or with black subepidermal glands (visible with a × 20 lens) scattered on the undersurface, and/or with conspicuous, sessile or punctate glands on the undersurface or along the margins, in addition to stipitate glands; veins usually prominent, main vein often excentric, secondary venation brochidodromous. Inflorescences either axillary or terminal racemes, these sometimes pyramidal in shape, sometimes aggregated into large showy panicles, inflorescence rachis and pedicels densely tomentose to glabrescent, sometimes covered in stellate hairs, these occasionally intermixed with stipitate glands; pedicels 5–22 mm long, articulated; bracts 2.5–6 mm long, caducous. Flowers bisexual, zygomorphic; calyx a short hypanthium with 5 sepals, 4.5–9 (– 11) mm long, the lower cucullate sepal generally slightly longer than the other four, apices entire or with a fimbriate-glandular margin, puberulous or tomentose, sometimes with a dense stellate indumentum (*Cenostigma eriostachys*, *Cenostigma tocantinum* and *Cenostigma macrophyllum*), the sepal lobes eglandular or with scattered dark, subepidermal glands, caducous, but the hypanthium persisting as a calyx ring in fruit; all 5 petals free and clawed, bright yellow, the median petal (7.5–) 9–15 (– 19) × 5–13 (– 17) mm, with red or orange markings on the inner surface of the blade, suborbicular to elliptic or spathulate, with a thickened, pubescent claw, the outer surface of which has short-stalked glands, these sometimes also on the dorsal surface of the blade, lateral petals 0.9–2.7 × 0.4–2 cm, broadly elliptic, sub-rectangular, obovate or suborbicular, petal claws pubescent and with stalked-glands, these sometimes also on the dorsal surface of the blade; stamens 10, free, filaments (7–) 8–14 (–21) mm long, pubescent on lower ⅔ to ½, with short-stipitate glands along entire length (except in *Cenostigma macrophyllum*); ovary pubescent with glands intermixed, these sometimes obscured by the indumentum, stigma a terminal fringed-chamber. Fruits laterally compressed, coriaceous to woody pods, (3.8–) 5–14 (– 16) × 1.2–3.3 (– 3.7) cm, with conspicuously thickened margins (an adaxial, proximal woody ridge or crest in *Cenostigma macrophyllum*), elastically dehiscent (sometimes tardily), the valves twisting at maturity, either glabrous or pubescent, smooth or prominently reticulately veined (on herbarium specimens), usually eglandular or with a few scattered stipitate or sessile glands (densely glandular in *Cenostigma microphyllum*). Seeds 2–6 (– 8) per pod, ovate-elliptic to ovate-orbicular, 9–19 × (6–) 8–12 × 1–3 mm, ochre, brown, or mottled, shiny.

##### Geographic distribution.

We recognise 20 taxa in 14 species, all of them neotropical; only two of these taxa do not require new names, while the rest are species of *Caesalpinia* here transferred to *Cenostigma*. The majority of species are found in central and NE Brazil, including parts of the Amazon. Two species extend around the circum-Amazonian arc of dry forests and adjacent cerrado, including in Paraguay, Argentina and Bolivia, and one taxon is also found in the seasonally dry inter-Andean valleys of Peru. Species are also found throughout Central America, from Panama northwards and in Mexico, extending to the Caribbean, with endemics in Cuba and Hispaniola.

##### Habitat.

Seasonally dry tropical forest, bushland and thicket (restinga, caatinga, semi-arid thorn scrub), wooded grassland (cerrado and cerradão) and terra firme forest.

##### Etymology.

From *ceno*- (Greek: empty) and *stigma*, presumably alluding to the chambered stigma (a character of many species of the Caesalpinia Group, and not restricted to *Cenostigma*).

##### References.

Lewis (1987: 34–35, 1998); Freire (1994); Ulibarri (1996); De Queiroz (2009: 129–130, see also under *Poincianella*, 121–128); Warwick and Lewis (2009); Lewis et al. (2010).

**Figure 29.**
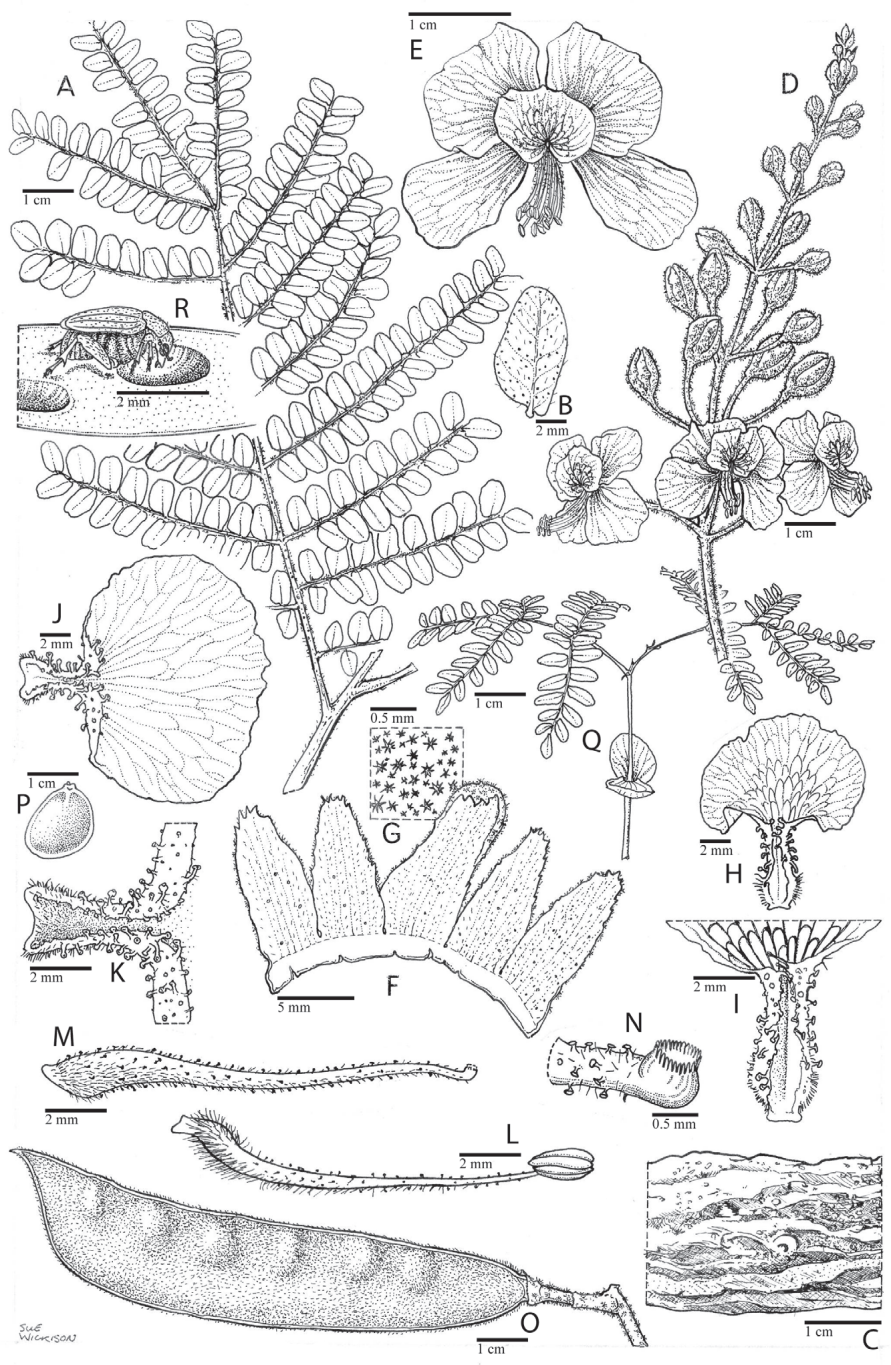
*Cenostigma eriostachys* (Benth.) E. Gagnon & G. P. Lewis. **A** part of bipinnate leaf **B** median leaflet undersurface, **C** section of branchlet bark **D** inflorescence **E** flower **F** calyx opened out **G** detail of stellate hairs on calyx **H** median petal **I** median petal claw **J** upper lateral petal **K** detail of lateral petal claw **L** stamen **M** gynoecium **N** stigma **O** fruit **P** seed **Q** seedling **R** bruchid emerged from seed. **A, B, P–R** from *Lewis & Hughes* 1799 **C** from *Lewis et al.* 1719 **D–N** from *Lewis et al.* 1718 **O** from *Lewis & Hughes* 1775. Drawn by Sue Wickison.

**Figure 30.**
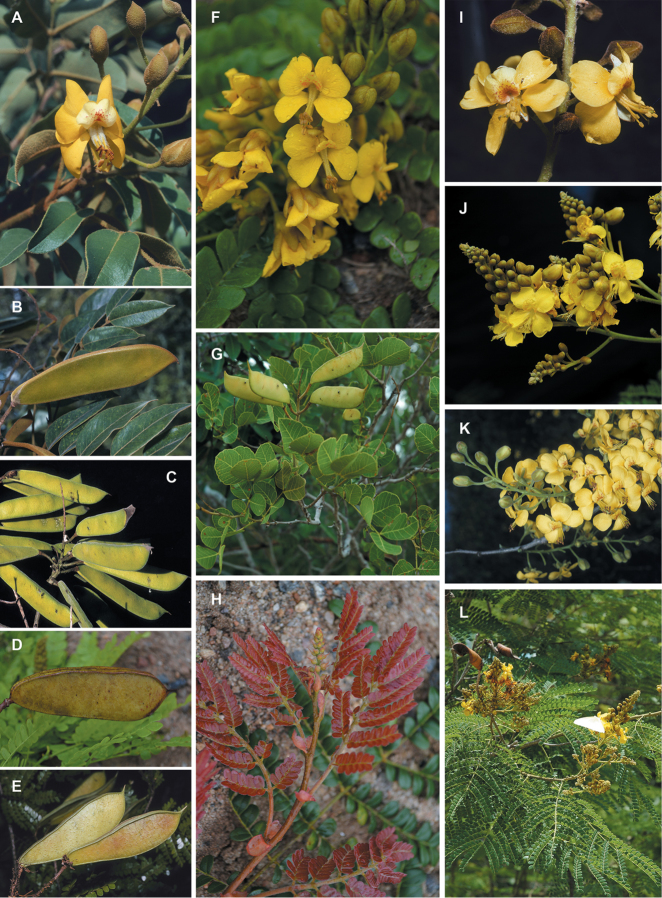
*Cenostigma macrophyllum* Tul. **A** flower (G. P. Lewis, Piauí, Brazil, *Lewis 1342* (K)) **B** fruit (G. P. Lewis, Brazil, *unvouchered*). *Cenostigma eriostachys* (Benth.) E. Gagnon & G. P. Lewis **C** fruits (C. E. Hughes, Oaxaca, Mexico, *Hughes 1935* (FHO)) **I** flowers (G. P. Lewis, Mexico, *MacQueen et al. 408* (K)). *Cenostigma pluviosum* (DC.) E. Gagnon & G. P. Lewis cf. var. intermedium (G.P. Lewis) E. Gagnon & G. P. Lewis **D** fruit **F** inflorescence **H** a new flush of leaves (E. Gagnon, Bahia, Brazil, *H.C. Lima et al. 7901* (RB)). Cenostigma pluviosum var. cabralianum (G. P. Lewis) E. Gagnon & G. P. Lewis **E** fruits (G. P. Lewis, Brazil, *Lewis et al. 2019* (K)). *Cenostigma marginatum* (Tul.) E. Gagnon & G. P. Lewis **G** leaves and fruits (C. E. Hughes, Bolivia, *Wood et al. 26514* (K)). Cenostigma pluviosum (DC.) E. Gagnon & G. P. Lewis var. pluviosum **J** inflorescences **L** inflorescences, foliage and dehisced fruits (C. E. Hughes, Santa Cruz, Bolivia, *Wood et al. 26552* (K)). *Cenostigma gaumeri* (Greenm.) E. Gagnon & G. P. Lewis **K** inflorescence (C. E. Hughes, Quintana Roo, Mexico, *Lewis & Hughes 1762* (K)).

#### 18.1 Cenostigma bracteosum

Taxon classificationPlantaeFabalesLeguminosae

(Tul.) E. Gagnon & G. P. Lewis comb. nov.

urn:lsid:ipni.org:names:77158073-1

##### Basionym.

*Caesalpinia bracteosa* Tul., Arch. Mus. Hist. Nat., Paris 4: 141. 1844. *Poincianella bracteosa* (Tul.) L. P. Queiroz, Leguminosas da Caatinga: 122. 2009.

##### Type.

BRAZIL, Piauí, *Gardner* 2144 (holotype P!; isotypes BM!, K!).

#### 18.2 Cenostigma eriostachys

Taxon classificationPlantaeFabalesLeguminosae

(Benth.) E. Gagnon & G. P. Lewis comb. nov.

urn:lsid:ipni.org:names:77158074-1

##### Basionym.

*Caesalpinia eriostachys* Benth., Bot. Voy. Sulphur: 88. 1844.

*Poincianella eriostachys* (Benth.) Britton & Rose, N. Amer. Fl. 23(5): 332. 1930.

##### Type.

COSTA RICA, Cocos Island, *Barclay s.n.* (lectotype K!, designated by Lewis, 1998).

*Schizolobium covilleanum* Pittier, Contr. U.S. Natl. Herb. 18: 231. 1917, pro parte (flowering material only).

Type. PANAMA, Prov. Coclé, between Aguadulce and Chico River, *Pittier 5105*.

#### 18.3 Cenostigma gaumeri

Taxon classificationPlantaeFabalesLeguminosae

(Greenm.) E. Gagnon & G. P. Lewis comb. nov.

urn:lsid:ipni.org:names:77158075-1

##### Basionym.

*Caesalpinia gaumeri* Greenm., Publ. Field Mus. Nat. Hist., Bot. Ser. 2: 330. 1912.

*Poincianella gaumeri* (Greenm.) Britton & Rose, N. Amer. Fl. 23(5): 333. 1930.

##### Type.

MEXICO, Yucatán, Progresso, 5 Mar 1899, *Millspaugh 1675* (holotype F).

*Poincianella guanensis* Britton, N. Amer. Fl. 23(5): 333. 1930.

*Caesalpinia guanensis* (Britton) León, Contr. Ocas. Mus. Hist. Nat. Colegio “De La Salle” 9: 12. 1950.

Type. CUBA, Remates de Guane, Pinar del Rio, Apr 1926, *Fors 3965* (holotype NY!).

#### 18.4 Cenostigma laxiflorum

Taxon classificationPlantaeFabalesLeguminosae

(Tul.) E. Gagnon & G. P. Lewis comb. nov.

urn:lsid:ipni.org:names:77158076-1

##### Basionym.

*Caesalpinia laxiflora* Tul., Arch. Mus. Hist. Nat., Paris 4: 143. 1844.

*Poincianella laxiflora* (Tul.) L. P. Queiroz, Leguminosas da Caatinga: 123. 2009.

##### Type.

BRAZIL, Bahia, near Villa da Barra, *Blanchet 3146* (isotypes BM!, BR!, F!, GH!, K!, MG!, P! [P02142655, P02142656, P02142657]).

Caesalpinia laxiflora Tul. var. pubescens Benth., Mart., Fl. Bras. 15(2): 70. 1870.

Type. BRAZIL, Bahia, near Maracás, *Martius s.n.* (holotype M!; isotypes M!).

#### 18.5 Cenostigma macrophyllum

Taxon classificationPlantaeFabalesLeguminosae

Tul., Ann. Sc. Nat. 2 Sér. 20: 141, pl. 3. 1843

##### Type.

BRAZIL, Mato Grosso, 1883, *C. Gaudichaud, Herb. Imp. Bras. No. 213* (P03014131!).

*Cenostigma gardnerianum* Tul., Ann. Sc. Nat. 2 Sér. 20: 141, pl. 3. 1843.

Type. BRAZIL, Piauí, *Gardner 2523* (isotype K!).

*Cenostigma angustifolium* Tul. , Ann. Sc. Nat. 2 Sér. 20: 141, pl. 3. 1843.

Types. BRAZIL, Bahia, Gentio do Ouro: Serra do Açuruá, *Blanchet 2798* (syntypes K!, MO!, P 03104099!); Marais de St-Antoine, *Blanchet 3144* (syntype P03104095!)

#### 18.6 Cenostigma marginatum

Taxon classificationPlantaeFabalesLeguminosae

(Tul.) E. Gagnon & G. P. Lewis comb. nov.

urn:lsid:ipni.org:names:77158077-1

##### Basionym.

*Caesalpinia marginata* Tul., Arch. Mus. Hist. Nat., Paris 4: 147. 1844.

##### Type.

BOLIVIA, Chiquitos, near San-Juan (Bois de la Tapira), without date, *d’Orbingy 831* (holotype P0242658!).

*Cenostigma sclerophyllum* Malme, Bih. Kongl. Svenska Vetensk.-Akad. Handl. 25 (11): 24. 1900.

Type. PARAGUAY, Colonia Risso, near Rio Apa, 20 Oct 1893, *Malme 1084* (lectotype S!, designated by Lewis (1998); isolectotype S!).

#### 18.7 Cenostigma microphyllum

Taxon classificationPlantaeFabalesLeguminosae

(Mart. ex G. Don) E. Gagnon & G. P. Lewis comb. nov.

urn:lsid:ipni.org:names:77158078-1

##### Basionym.

*Caesalpinia microphylla* Mart. ex G. Don, Gen. Syst. 2: 431. 1832.

*Poincianella microphylla* (Mart. ex. G. Don) L. P. Queiroz, Leguminosas da Caatinga: 124. 2009.

##### Type.

BRAZIL, Bahia, in sylvis catingas, *Martius Obsv. 2274* (lectotype M!, designated by Lewis (1998); isolectotypes K!, M!).

#### 18.8 Cenostigma myabense

Taxon classificationPlantaeFabalesLeguminosae

(Britton) E. Gagnon & G. P. Lewis comb. nov.

urn:lsid:ipni.org:names:77158079-1

##### Basionym.

*Caesalpinia myabensis* Britton, Mem. Torrey Bot. Club 16: 66. 1920.

*Poincianella myabensis* (Britton) Britton & Rose, N. Amer. Fl. 23(5): 334. 1930.

##### Type.

CUBA, Oriente, between Holguin and Myabe, Apr 1909, *Shafer 1403* (holotype NY!; isotype A!).

Libidibia pauciflora Griseb. var. ? puberula Griseb., Cat. Pl. Cub.: 79. 1866.

Type. CUBA, *Wright 2362* (incorrectly given as “1362”).

*Caesalpinia hornei* Britton, Mem. Torrey Bot. Club 16: 67. 1920.

*Poincianella hornei* (Britton) Britton & Rose, N. Amer. Fl. 23(5): 333 (1930).

Caesalpinia myabensis var. hornei (Britton) Barreto, Acta Bot. Cub. 89: 5 1992.

Type. CUBA, Ciego de Avila, Camaguey, 3 Sep 1905, *Horne 95* (holotype NY!).

*Caesalpinia subglauca* Britton in Mem. Torrey Bot. Club 16: 66 (1920).

*Poincianella subglauca* (Britton) Britton & Rose, N. Amer. Fl. 23(5): 333 (1930).

Caesalpinia myabensis var. subglauca (Britton) Barreto, Acta Bot. Cub. 89: 6 (1992).

Type. CUBA, Oriente, near Santiago, *Britton et al. 12596* (holotype NY!).

*Poincianella clementis* Britton, N. Amer. Fl. 23(5): 333. 1930.

*Caesalpinia clementis* (Britton) León, Contr. Ocas. Mus. Hist. Nat. Colegio “De La Salle” 9: 12. 1950.

Caesalpinia myabensis var. clementis (Britton) Barreto, Acta Bot. Cub. 89: 6. 1992.

Type. CUBA, Oriente, Renté, Santiago, Jul 1919, *Clement 135* (holotype NY!; isotype HAC!).

*Caesalpinia hermeliae* León, Contr. Ocas. Mus. Hist. Nat. Colegio “De La Salle” 9: 12. 1950.

Caesalpinia myabensis var. hermeliae (León) Barreto, Acta Bot. Cub. 89: 5. 1992.

Type. CUBA, Oriente, SW of Holguin, orillas del monte de Caguairanal, 18 Mar 1932, *León & Garcia 15501* (holotype LS (transferred to HAC)!; isotypes HAC!, NY!).

#### 18.9 Cenostigma nordestinum

Taxon classificationPlantaeFabalesLeguminosae

E. Gagnon & G. P. Lewis nom. nov.

urn:lsid:ipni.org:names:77158101-1

Caesalpinia gardneriana Benth., in Mart., Fl. Bras. 15 (2): 68. 1870.
Poincianella gardneriana (Benth.) L. P. Queiroz, Leguminosas da Caatinga: 123. 2009, non Cenostigma gardnerianum Tul. (1843), a synonym of Cenostigma macrophyllum Tul. (1843).

##### Type.

BRAZIL, Piauí, between Praya Grande and Boa Esperança, Feb 1839, *Gardner 2148* (holotype K!; isotype BM!).

#### 18.10 Cenostigma pellucidum

Taxon classificationPlantaeFabalesLeguminosae

(Vogel) E. Gagnon & G. P. Lewis comb. nov.

urn:lsid:ipni.org:names:77158080-1

##### Basionym.

*Caesalpinia pellucida* Vogel, Linnaea 10: 601. 1836.

*Poincianella pellucida* (Vogel) Britton & Rose, N. Amer. Flora 23(5): 334. 1930.

##### Type.

DOMINICAN REPUBLIC, *Ehrenberg s.n.* (isotype NY!).

#### 18.11 Cenostigma pinnatum

Taxon classificationPlantaeFabalesLeguminosae

(Griseb.) E. Gagnon & G. P. Lewis comb. nov.

urn:lsid:ipni.org:names:77158094-1

##### Basionym.

*Libidibia pinnata* Griseb. Cat. Pl. Cub.: 79. 1866 (As “Lebidibia pinnata”).

*Caesalpinia pinnata* (Griseb.) C. Wright, in Suav., Anales Acad. Ci. Med. Habana 5: 404. 1869.

*Poincianella pinnata* (Griseb.) Britton & Rose, N. Amer. Fl. 23(5): 335. 1930.

##### Type.

CUBA, *Wright 2360* (holotype GOET!; isotypes GH!, K!, NY!).

*Caesalpinia oblongifolia* Urban, Symb. Ant. 2: 281 (1900).

*Poincianella oblongifolia* (Urban) Britton & Rose, N. Amer. Fl. 23(5): 335 (1930).

Type. As for *Caesalpinia pinnata*.

*Poincianella savannarum* Britton & Wilson, N. Amer. Fl. 23(5): 335 1930.

*Caesalpinia savannarum* (Britton & Wilson) León, Contr. Ocas. Mus. Hist. Nat. Colegio “De La Salle” 10 (Fl. Cub. 2): 283. 1951.

Caesalpinia oblongifolia var. savannarum (Britton & Wilson) A. Borhidi & O. Muniz, Bot. Közlem. 62 (1): 25. 1975.

Type. CUBA, Sancti Spiritus, 20 Jul 1915, *León & Roca 7835* (holotype NY!).

#### 18.12 Cenostigma pluviosum

Taxon classificationPlantaeFabalesLeguminosae

(DC.) E. Gagnon & G. P. Lewis comb. nov.

urn:lsid:ipni.org:names:77158081-1

##### Basionym.

*Caesalpinia pluviosa* DC., Prodr. 2: 483. 1825.

*Poincianella pluviosa* (DC.) L. P. Queiroz, Leguminosas da Caatinga: 126. 2009.

##### Type.

BRAZIL, 1819, *Leandro di Sacramento 5* (P02142667!).

#### 18.12.1 Cenostigma pluviosum var. pluviosum

Taxon classificationPlantaeFabalesLeguminosae

Caesalpinia floribunda Tul., Arch. Mus. Hist. Nat., Paris 4: 140. 1844. Type. BOLIVIA, Prov. de Chiquitos, camino de San Rafel a Santa Ana, [without date], *Orbigny 1039* (holotype P02142650!; isotypes G, P02142651!).
Caesalpinia taubertiana S. Moore, Trans. Linn. Soc. London, Bot. 4: 345. 1895. Type. BRAZIL, near Corumbá, Jan 1891–1892, *Moore 1037* (holotype BM!; isotype BM!).

#### 18.12.2 Cenostigma pluviosum var. cabralianum

Taxon classificationPlantaeFabalesLeguminosae

(G. P. Lewis) E. Gagnon & G. P. Lewis comb. nov.

urn:lsid:ipni.org:names:77158096-1

##### Basionym.

Caesalpinia pluviosa var. cabraliana G. P. Lewis, Caesalpinia: Revis. Poincianella-Erythrostmeon group: 148. 1998.

Poincianella pluviosa var. cabraliana (G. P. Lewis) L. P. Queiroz, Neodiversity 5(1): 11. 2010.

##### Type.

BRAZIL, Bahia, Mun. Santa Cruz de Cabrália, c. 12 km NW of Porto Seguro, 27 Nov 1979, *Mori et al. 13029* (holotype CEPEC!; isotypes K!, NY).

#### 18.12.3 Cenostigma pluviosum var. intermedium

Taxon classificationPlantaeFabalesLeguminosae

(G. P. Lewis) E. Gagnon & G. P. Lewis comb. nov.

urn:lsid:ipni.org:names:77158097-1

##### Basionym.

*Caesalpinia pluviosa var. intermedia* G. P. Lewis, Caesalpinia: Revis. Poincianella-Erythrostemon group: 141. 1998.

Poincianella pluviosa var. intermedia (G. P. Lewis) L. P. Queiroz, Leguminosas da Caatinga: 127. 2009.

##### Type.

BRAZIL, Bahia, Abaíra, road to Jussiape, 15 Feb 1987, *Harley et al. 24326* (holotype SPF; isotype K!).

#### 18.12.4 Cenostigma pluviosum var. maraniona

Taxon classificationPlantaeFabalesLeguminosae

(G. P. Lewis & C. E. Hughes) E. Gagnon & G. P. Lewis comb. nov.

urn:lsid:ipni.org:names:77158087-1

##### Basionym.

Caesalpinia pluviosa var. maraniona G. P. Lewis & C. E. Hughes, Kew Bull. 65(2): 213-217. 2010.

##### Type.

 PERU, Cajamarca, Celendín, Marañón Valley, km 50 rd from Celendín to Leimebamba, 23 Apr 2002, fl. & fr., *Hughes*, *Daza & Forrest 2215* (holotype FHO!; isotypes K!, MOL!).

#### 18.12.5 Cenostigma pluviosum var. paraense

Taxon classificationPlantaeFabalesLeguminosae

(Ducke) E. Gagnon & G. P. Lewis comb. nov.

urn:lsid:ipni.org:names:77158088-1

##### Basionym.

*Caesalpinia paraensis* Ducke, Archiv. Jard. Bot. Rio de Janeiro 4: 59. 1925.

Caesalpinia pluviosa var. paraensis (Ducke) G. P. Lewis, Caesalpinia: Revis. Poincianella-Erythrostemon group: 150. 1998.

Poincianella pluviosa var. paraensis (Ducke) L. P. Queiroz, Neodiversity 5(1): 11. 2010.

##### Type.

BRAZIL, Pará, near Monte Alegre, *Ducke s.n.* (BM!, K!, MG, RB).

#### 18.12.6 Cenostigma pluviosum var. peltophoroides

Taxon classificationPlantaeFabalesLeguminosae

(Benth.) E. Gagnon & G. P. Lewis comb. nov.

urn:lsid:ipni.org:names:77158098-1

##### Basionym.

*Caesalpinia peltophoroides* Benth., Mart., Fl. Bras. 15(2): 72. 1870.

*Caesalpinia pluviosa var. peltophoroides* (Benth.) G. P. Lewis, Caesalpinia: Revis. Poincianella-Erythrostemon group: 146. 1998.

Poincianella pluviosa var. peltophoroides (Benth.) L. P. Queiroz, in Neodiversity 5(1): 11. 2010.

##### Type.

BRAZIL, Rio de Janeiro, *Glaziou* 1032 (syntypes BM!, BR!, F!, P02142662!); *Glaziou* 6 (syntype BR!).

#### 18.12.7 Cenostigma pluviosum var. sanfranciscanum

Taxon classificationPlantaeFabalesLeguminosae

(G. P. Lewis) E. Gagnon & G. P. Lewis comb. nov.

urn:lsid:ipni.org:names:77158089-1

##### Basionym.

Caesalpinia pluviosa var. sanfranciscana G. P. Lewis, Caesalpinia: Revis. Poincianella-Erythrostemon group: 151. 1998.

Poincianella pluviosa var. sanfranciscana (G. P. Lewis) L. P. Queiroz, Leguminosas da Caatinga: 127. 2009.

##### Type.

BRAZIL, Bahia, 35 km S of Livramento do Brumado, 1 Apr 1991, *Lewis & Andrade 1932* (holotype CEPEC!; isotype K!).

#### 18.13 Cenostigma pyramidale

Taxon classificationPlantaeFabalesLeguminosae

(Tul.) E. Gagnon & G. P. Lewis comb. nov.

urn:lsid:ipni.org:names:77158099-1

##### Basionym.

*Caesalpinia pyramidalis* Tul., Arch. Mus. Hist. Nat., Paris 4: 139. 1844.

*Poincianella pyramidalis* (Tul.) L. P. Queiroz, Leguminosas da Caatinga: 128. 2009.

##### Type.

BRAZIL, Serra Jacobina, 1841, *J. S. Blanchet 3425* (holotype P003790235!; isotypes BM!, BR!, F!, MG!).

#### 18.13.1 Cenostigma pyramidale var. pyramidale

Taxon classificationPlantaeFabalesLeguminosae

Caesalpinia pyramidalis var. alagoensis Tul., Arch. Mus. Hist. Nat., Paris 4: 140. 1844. Type. BRAZIL, Alagoas, banks of the Rio St. Francisco at Propiá, Feb 1838, *Gardner 1278* (holotpye BM!; isotypes F!, GH!, K!, US!).

#### 18.13.2 Cenostigma pyramidale var. diversifolium

Taxon classificationPlantaeFabalesLeguminosae

(Benth.) E. Gagnon & G. P. Lewis comb. nov.

urn:lsid:ipni.org:names:77158100-1

##### Basionym.

Caesalpinia pyramidalis var. diversifolia Benth., Mart., Fl. Bras. 15(2): 69. 1870.

##### Type.

BRAZIL, Maranhão, Jun 1841, *Gardner 6006* (lectotype K!, designated by Lewis, 1998; isolectotype BM!).

#### 18.14 Cenostigma tocantinum

Taxon classificationPlantaeFabalesLeguminosae

Ducke, Arch. Jard. Bot. Rio de Janeiro 29, pl. 10 (1915)

##### Type.

BRAZIL, Pará, Alcobaça, Rio Tocantins, *Ducke s.n., H.A.M.P. no. 15643* (holotype MG).

#### 19. Libidibia

Taxon classificationPlantaeFabalesLeguminosae

(DC.) Schltdl., in Linnaea 5: 192. 1830, descr. emended E. Gagnon & G. P. Lewis

Figs 31 , 32

Caesalpinia section Libidibia DC. (1825).
Stahlia Bello (1881), **syn. nov.**

##### Diagnosis.

*Libidibia* is related to *Hoffmannseggia*, *Stenodrepanum*, *Balsamocarpon* and *Zuccagnia* but differs in being a genus of medium to tall trees, 6–20 m in height (versus woody based perennial herbs to shrubs, 10 cm to 5 m tall), most species have a distinctive, smooth patchwork bark in shades of white, grey and green (“snake skin bark”) a characteristic not found in the other four genera. *Libidibia* (except *Libidibia monosperma*) has bipinnate leaves (*Balsamocarpon* and *Zuccagnia* are pinnate) and coriaceous or woody, glabrous, eglandular, indehiscent fruits which dry black (red in *Libidibia monosperma*) versus thick, turgid, glandular, resinous, indehiscent fruits (*Balsamocarpon*), or laterally compressed, gall-like, ?indehiscent fruits covered in trichomes (*Zuccagnia*). *Stenodrepanum* and *Hoffmannseggia* are bipinnate but the fruits of most species of *Hoffmannseggia* are dehiscent with twisting pod valves and persistent sepals (in *Libidibia* sepals are caducous in fruit); the fruits of *Stenodrepanum* are narrow, cylindrical and torulose.

##### Type.

*Libidibia coriaria* (Jacq.) Schltdl. ≡ *Poinciana coriaria* Jacq.

##### Emended description.

Small to medium-sized or large unarmed trees, 6–20+ meters in height; bark hard, smooth, with a patchwork of shades of grey, white and pale green, often referred to as snake skin bark, (except in *Libidibia coriaria* and *Libidibia monosperma*, where it is rough and fissured). Stipules not seen. Leaves alternate, bipinnate and ending in a pair of pinnae plus a single terminal pinna, rarely pinnate (*Libidibia monosperma*); pinnae (in bipinnate species) in 2–10 opposite pairs, plus a single terminal pinna; leaflets opposite, in 3–31 pairs per pinna, ovate, elliptic to oblong, apex rounded, mucronate or acute, base often oblique, subcordate, rounded or obtuse, eglandular or with subsessile gland dots on the undersurface of the blades, on either side of the midvein, glabrous to occasionally puberulous; in bipinnate leaves the leaflets (3–) 4–31 × 2.5–14 mm; in pinnate leaves, leaflets are much larger, c. 40–90 × 15–35 mm. Inflorescences terminal or axillary racemes or panicles, sometimes corymbose, with pedicellate flowers. Flowers bisexual, zygomorphic; calyx comprising a hypanthium and 5 sepals, the lower sepal slightly longer and cucullate in bud, caducous, but hypanthium persisting as a calyx ring around the pedicel as pods mature; petals 5, free, yellow or white, the median petal sometimes flecked or blotched orange or red; stamens 10, free, pubescent on the lower half of the filaments, eglandular (except for *Libidibia ferrea*, which has stipitate glands); ovary eglandular, glabrous or pubescent. Fruit coriaceous to woody, oblong-elliptic to suborbicular, straight (contorted in *Libidibia coriaria*), indehiscent, eglandular, glabrous, black (red and somewhat fleshy in *Libidibia monosperma*), 15–80 × 10–30 mm. Seeds oblong to elliptic, somewhat laterally compressed, smooth.

##### Geographic distribution.

A genus of ten taxa in seven species in the Neotropics. One species in Mexico, one widespread in Brazil, one in Colombia, Venezuela and the Antilles, one in Colombia, Ecuador and Peru, one in Paraguay, Bolivia, Argentina and SW Brazil, one (*Libidibia monosperma*, previously in the monospecific genus *Stahlia*) endemic to Puerto Rico and the Dominican Republic, and *Libidibia coriaria* widespread throughout Mexico, Central America, the Caribbean and NW South America. Other species perhaps waiting to be discovered and described, both in the field and in herbaria; the genus needs revising.

##### Habitat.

Seasonally dry tropical forest and thorn scrub (including Brazilian caatinga) and savanna woodland. *Libidibia monosperma* occurs along the margins of mangrove swamps and in marshy deltas, in drier edaphic conditions.

##### Etymology.

The name *Libidibia* is derived from the vernacular name ‘libi-dibi’ or ‘divi-divi’ used for some species.

##### References.

Britton (1927); Britton and Rose (1930: 221, 318–319); Burkart (1936, *Caesalpinia melanocarpa*: 78–82); Macbride (1943, *Caesalpinia paipai*: 193–194); Little and Wadsworth (1964); U.S. Fish and Wildlife Service (1995); Ulibarri (1996); De Queiroz (2009: 130–133); Borges et al. (2012); Barreto Valdés (2013).

**Figure 31.**
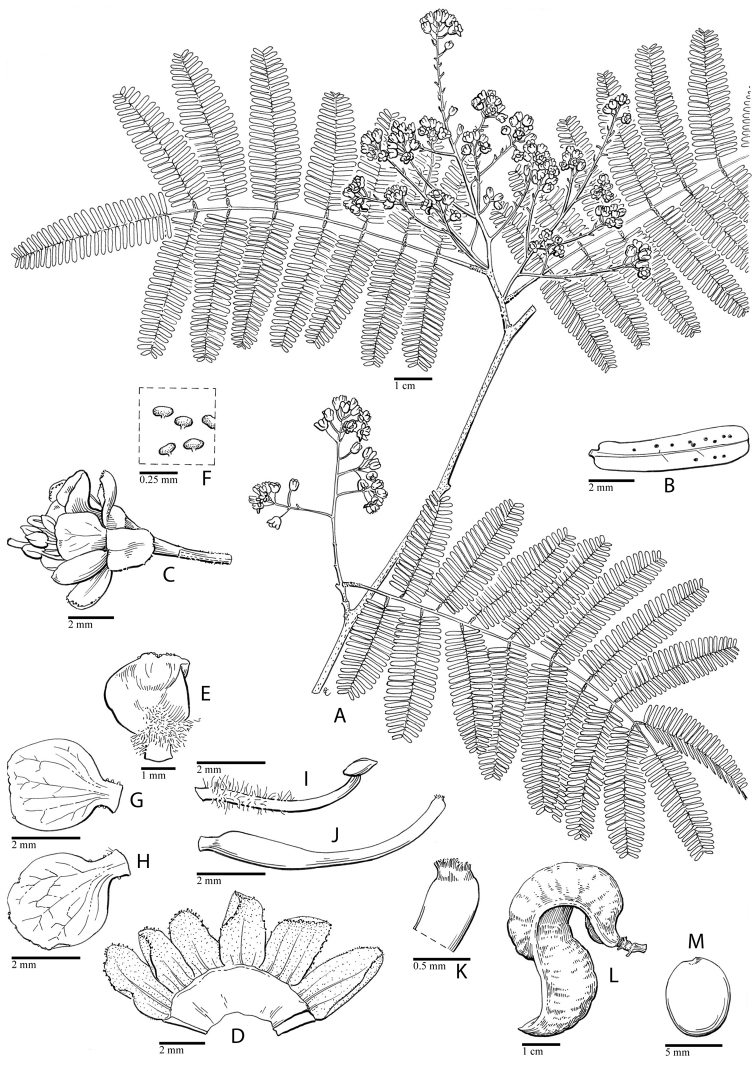
*Libidibia coriaria*. **A** inflorescences and foliage **B** leaflet undersurface showing glands **C** flower **D** calyx opened out **E** median petal **F** detail of glands on back of median petal **G** upper lateral petal **H** lower lateral petal **I** stamen **J** gynoecium **K** stigma **L** fruit **M** seed. **A–C** from *Hughes* 1495 **D–M** from *Macqueen* 8. Drawn by Eleanor Catherine.

**Figure 32.**
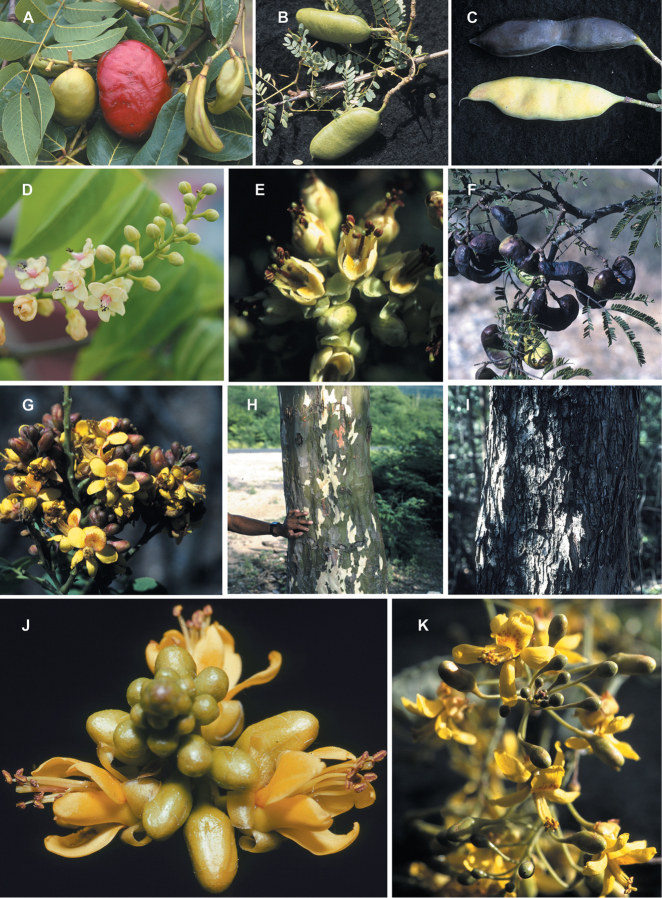
*Libidibia monosperma* (Tul.) E. Gagnon & G. P. Lewis. **A** fruits and foliage (M. F. Gardner, Dominican Republic, *Gardner & Knees 7027* (E)) **D** inflorescence (Carlos Pacheco, Wikicommons (https://commons.wikimedia.org/wiki/File:Stahlia_monosperma_flower_(5840542648).jpg), Puerto Rico, USA, *unvouchered*). *Libidibia paraguariensis* (D. Parodi) G. P. Lewis **B** unripe fruits (C. E. Hughes, Santa Cruz, Bolivia, *Hughes 2475* (FHO)). *Libidibia glabrata* (Kunth) C. Cast. & G. P. Lewis **C** fruits **K** inflorescence (C. E. Hughes, La Libertad, Peru, *Eastwood et al. RJE85* (FHO)). *Libidibia coriaria* (Jacq.) Schltdl. **E** flowers (C. E. Hughes, Estelí, Nicaragua, *MacQueen 8* (FHO)) **F** branch with fruits (C. E. Hughes, Metapan, El Salvador, *Lewis 1745* (K)) **I** bark (C. E. Hughes, Oaxaca, Mexico, *Hughes 1933* (FHO)). *Libidibia sclerocarpa* (Standl.) Britton & Rose, **G** inflorescence (C. E. Hughes, Oaxaca, Mexico, *Lewis 1800* (K)) **H** bark (C. E. Hughes, Oaxaca, Mexico, *Hughes et al. 1494* (FHO)). Libidibia ferrea var. parvifolia (Benth.) L. P. de Queiroz **J** inflorescence (G. P. Lewis, Bahia, Brazil, *unvouchered*).

#### 19.1 Libidibia coriaria

Taxon classificationPlantaeFabalesLeguminosae

(Jacq.) Schltdl., Linnaea 5: 193. 1830

##### Basionym.

*Poinciana coriaria* Jacq., Select. Stirp. Amer. Hist. 123, pl. 175, f. 36 (flower, fruit and seed). 1763.

*Caesalpinia coriaria* (Jacq.) Willd., Sp. Pl. 2: 532. 1799.

##### Type.

Curação, “Habitat in Curação & Carthagenae frequens; in limosis praesertim inudatisque maritimis; ad salinas”, [no date], *Jacquin s.n.* (holotype probably in W; photo Field Museum 1794 of probable isotype “Hb. Willdenow” (fl.); by micro. Reprod. of the same Hb. *Willdenow* 8023: SI).

*Caesalpinia thomaea* Spreng., Syst. Veg. 2: 343. 1825.

Type. “Ins. S. Thomae, Bertero”.

#### 19.2 Libidibia ferrea

Taxon classificationPlantaeFabalesLeguminosae

(Mart. ex Tul.) L. P. Queiroz, Leguminosas da Caatinga: 130. 2009

##### Basionym.

*Caesalpinia ferrea* Mart. ex Tul., Arch. Mus. Hist. Nat. Paris 4: 137. 1844.

##### Type.

BRAZIL, “Province of Alagoas, Tropical Brazil, *Gardner 1277* (holotype P02736428!; isotypes BM!, K!).

#### 19.2.1 Libidibia ferrea var. ferrea

Taxon classificationPlantaeFabalesLeguminosae

Caesalpinia ferrea var. petiolulata Tul., Arch. Mus. Hist. Nat. Paris 4: 138. 1844. Type. BRAZIL, Piaui (“Piauhy”), 1839, *Gardner 2147* (syntypes K!, P02736427!); Bahia, *Blanchet 3264* (syntype P02142648!).
Caesalpinia ferrea var. megaphylla Tul., in Arch. Mus. Hist. Nat. Paris 4: 139. 1844. Type. BRAZIL, Piaui (“Piauhy”), dry woods near Villa do Crato, Jan 1839, *Gardner 1934* (holotype P02736441!; isotype K!).

#### 19.2.2 Libidibia ferrea var. glabrescens

Taxon classificationPlantaeFabalesLeguminosae

(Benth.) L. P. Queiroz, Leguminosas da Caatinga: 131. 2009

##### Basionym.

Caesalpinia ferrea var. glabrescens Benth., Mart., Fl. Brasil 15(2): 70. 1870.

##### Type.

BRAZIL, Sergipe-Alagoas, “banks of the Rio St. Francisco”, Feb 1838, *Gardner 1276* (holotype K).

#### 19.2.3 Libidibia ferrea var. leiostachya

Taxon classificationPlantaeFabalesLeguminosae

(Benth.) L. P. Queiroz, Neodiversity 5(1): 11. 2010

##### Basionym.

Caesalpinia ferrea Mart. ex Tul. var. leiostachya Benth., Mart., Fl. Bras. 15(2): 70. 1870. *Caesalpinia leiostachya* (Benth.) Ducke, Mem. Inst. Oswaldo Cruz 51: 458. 1953.

##### Type.

BRAZIL “prope Rio de Janeiro juxta viam ad Jacarépaguá ducentem”, 13 Mar 1868, *Glaziou 2555* (P02736434!).

#### 19.2.4 Libidibia ferrea var. parvifolia

Taxon classificationPlantaeFabalesLeguminosae

(Benth.) L. P. Queiroz, Leguminosas da Caatinga: 133. 2009

##### Basionym.

Caesalpinia ferrea var. parvifolia Benth., Mart., Fl. Brasil 15(2): 70. 1870.

##### Type.

BRAZIL, “in sylvis catingas de interioribus prov. Bahia”, *Martius s.n.*

#### 19.3 Libidibia glabrata

Taxon classificationPlantaeFabalesLeguminosae

(Kunth) C. Castellanos & G. P. Lewis, Revista Acad. Colomb. Ci. Exact. 36(139): 183. 2012

##### Basionym.

*Caesalpinia glabrata* Kunth, Nov. Gen. Sp. 6: 326. 1823.

##### Type.

 PERU, “Crescit inter urbem Caxamarcae et pagum Madgalenae, Peruvia”, *M. A. Bonpland 3712* (holotype P00679209!; isotype P02142659!, photo K!, photo and fragment F 937253).

*Libidibia corymbosa* (Benth.) Britton & Killip, Ann. N. Y.Acad. Sci. 35(3): 189 (1936).

*Caesalpinia corymbosa* Benth., Pl. Hartw.: 117. 1832.

Type. ECUADOR, Guayaquil, [without date], *Hartweg 651* (holotype K!; isotypes K!, P! (two sheets: P02737048!, P02737051!), photo at F, no. 1774).

*Caesalpinia paipai* Ruíz & Pav., Fl. Peruv. 4, Ic. 375. 1830.

Type. PERU, “Limae & Chancay” (lectotype based on Ic. 375, fragment of the material probably used for the illustration “Hb. Ruíz & Pavon, Peru, Chacau” MA: F842538).

Caesalpinia paipai var. pubens J.F. Macbr., Field Mus. Nat. Hist. Bot. Ser. (Fl. Peru) 13, 3, 1: 193. 1943.

Type. PERU, Dpto. Piura: Salitral y Serrán, Mar 1912, *Weberbauer 5994* (holotype F).

#### 19.4 Libidibia monosperma

Taxon classificationPlantaeFabalesLeguminosae

(Tul.) E. Gagnon & G. P. Lewis comb. nov.

urn:lsid:ipni.org:names:77158082-1

##### Basionym.

*Caesalpinia monosperma* Tul., Arch. Mus. Hist. Nat. Paris 4: 148. 1844. *Stahlia monosperma* (Tul.) Urb., Symb. Antill. 2(2): 285. 1900.

##### Type.

PUERTO RICO, without exact locality or date, *A. Plée* 713 (lectotype P03090076, designated by Santiago-Valentín, Sánchez-Pinto & Francisco-Ortega, 2015).

Stahlia monosperma var. domingensis Standl, Trop. Woods 40: 16. 1934.

Type. DOMINICAN REPUBLIC, delta of Soco River, *J.C. Scarff s.n.* (“type” Hb. Field Mus. No. 7147180; Yale No. 27244).

*Stahlia maritima* Bello, Anales Soc. Esp. Hist. Nat. 10: 255. 1881.

Type. PUERTO RICO, Guánica, in sylvis inter Barina et la Boca, 2 Mar 1886, *P. E. E. Sintensis 3876* (neotype NY, designated by Santiago-Valentín, Sánchez-Pinto & Francisco- Ortega, 2015; isoneotypes BM, G, GH, NY, P, W).

#### 19.5 Libidibia paraguariensis

Taxon classificationPlantaeFabalesLeguminosae

(D. Parodi) G. P. Lewis, in Mabberley, Pl. Book (ed. 3): 1021. 2008

##### Basionym.

*Acacia paraguariensis* D. Parodi, Revista Farm. 3: 7. 1862.

*Caesalpinia paraguariensis* (D. Parodi) Burkart, Darwiniana 10(1): 26. 1952.

##### Type.

PARAGUAY, “Arbor sylvestris in ripa fluminis Paraguay” (holotype probably at BAF, not found).

*Caesalpinia melanocarpa* Griseb., Abh. Königl. Ges. Wis. Göttingen (Pl. Lorentz) 19: 80. 1874.

Type. ARGENTINA, Tucumán, infrecuens in sylvis subtropicis et in campis, pr. La Cruz, 20–24 Apr 1872, *Lorentz 196*. (holotype GOET; isotypes CORD, SI).

*Caesalpinia coriaria* Micheli, Mem. Soc. Phys. Genève 29(7): 42. 1883, non (Jacq.) Willd. (1799).

Type. PARAGUAY, Assomption in hortis culta, *Balansa 1397* and *1397a* (syntypes BAF, G, K!).

#### 19.6 Libidibia punctata

Taxon classificationPlantaeFabalesLeguminosae

(Willd.) Britton, Sci. Surv. Porto Rico & Virgin Islands 5: 378. 1924

##### Basionym.

*Caesalpinia punctata* Willd., Enum. Pl. 455. 1809.

##### Type.

Herb. Willd. 822, plant cult. Source erroneously attributed to Brazil.

*Caesalpinia granadillo* Pittier, Bol. Cien. Técn. Mus. Com. Venez. 1:56. 1926.

*Libidibia granadillo* (Pittier) Pittier, Man. Pl. Usual. Venez. (Suppl.): 37. 1939.

Type. VENEZUELA, Zulia: selva montañosa de San Martín, Río Palmar, 15 Oct 1922, *Pittier 10515* (holotype VEN, isotypes GH, P02736828!, US!).

*Caesalpinia ebano* H. Karst., Fl. Columb. 2: 57, pl. 129. 1862.

*Libidibia ebano* (H. Karst.) Britton & Killip, Ann. New York Acad. Sci. 35(4): 189. 1936.

Type. COLOMBIA, “regiones septentrionales calidus, siccas”.

#### 19.7 Libidibia sclerocarpa

Taxon classificationPlantaeFabalesLeguminosae

(Standl.) Britton & Rose, N. Amer. Fl. 23 (5): 319. 1930

##### Basionym.

*Caesalpinia sclerocarpa* Standl., Contrib. U. S. Nat. Herb. 20(6): 214–215. 1919.

##### Type.

MEXICO, Oaxaca, between San Geronimo and La Venta, alt. 50 m, 13 Jul 1895, *E. W. Nelson 2784* (holotype US 229315).

#### 20. Balsamocarpon

Taxon classificationPlantaeFabalesLeguminosae

Clos, Fl. Chile. 2(2): 226; Atlas Botanico t. 20. 1846

Figs 33 , 34A–C

##### Type.

*Balsamocarpon brevifolium* Clos

##### Description.

Shrub 1–2 m tall, with long terete branches with thin, straight, 3–5 mm long, often caducous spines. Stipules deltoid, hairy, glandular. Leaves in fascicles on short brachyblasts, pinnate, 3–8 mm long; leaflets in 3–4 pairs, elliptic-obovate to orbicular, 1.5–4.5 × 1–2 mm, glabrous, fleshy. Inflorescences composed of short racemes; pedicels and rachis hairy and glandular; bracts deltoid, hairy and glandular. Flowers bisexual, sub-zygomorphic; calyx comprising a hypanthium and 5 sepals, c. 5–6 × 4.2 mm, fimbriate, hairy and with glandular trichomes, sepals persistent in fruit; petals 5, free, yellow, obovate, subequal, short-clawed, 10 × 3–4.5 mm, with glandular trichomes on the dorsal surface; stamens 10, free, filaments pubescent, eglandular; ovary glandular, finely pubescent, stigma a fringed chamber. Fruit a thick, turgid, resinous, glandular, indehiscent pod, 2.5–4 × 1.5 cm, 3–4-seeded.

##### Geographic distribution.

A monospecific genus endemic to northern Chile, from the Coquibo and La Serena valleys.

##### Habitat.

Desert scrub, rocky hillsides.

##### Etymology.

From *balsamo*- (Gk.: balsam) and *carpos* (Gk.: fruit), the pods yield a sticky resin traditionally used for tanning.

##### References.

Burkart (1940: 162); Ulibarri (1996, 2008); Nores et al. (2012).

**Figure 33.**
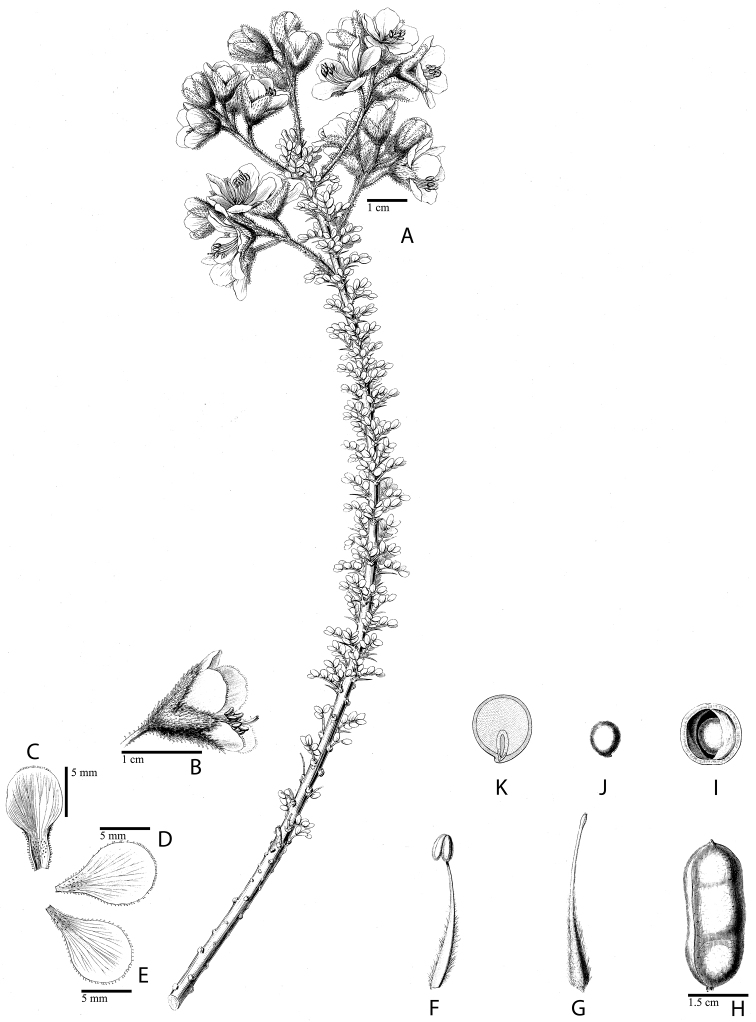
*Balsamocarpon brevifolium* Clos. **A** flowering stem **B** flower **C** median petal **D** upper lateral petal **E** lower lateral petal **F** stamen **G** gynoecium **H** fruit **I** dissected seed **J** longitudinal section of seed **K** embryo. Drawn by A. Riocreux, first published in Historia fysica y polica de Chile, Botanica, Atlas, col. 1: t. 20 (1854). Scale bars were estimated for this plate based on descriptions and comparison with herbarium specimens; we were unable to estimate these for **F**, **G**, **K**, **J**, **I**.

**Figure 34.**
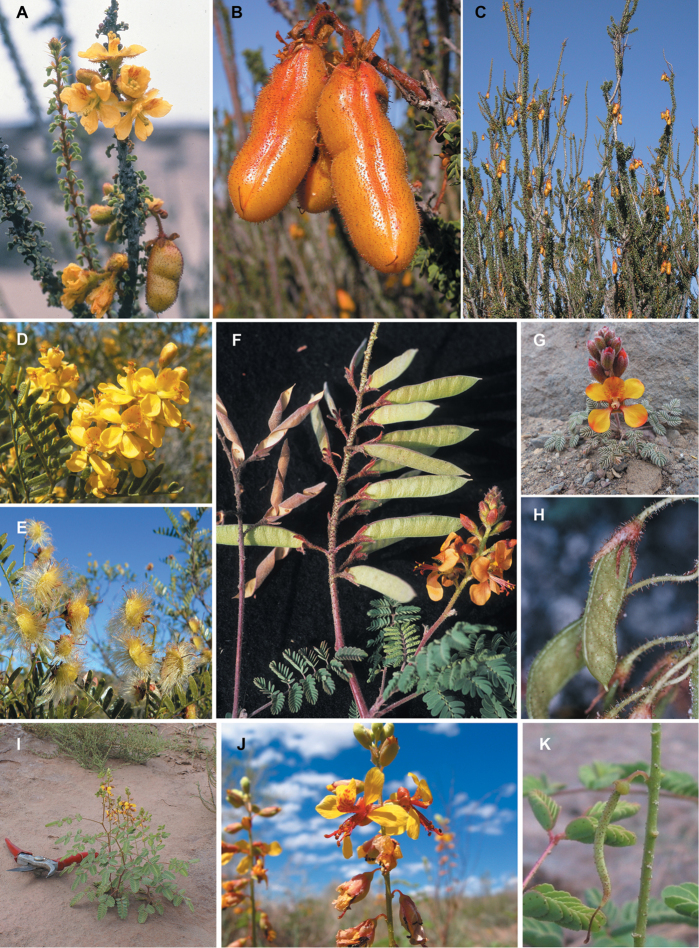
*Balsamocarpon brevifolium* Clos. **A** branch with inflorescence and fruit (M.F. Gardner, Chile, *Gardner & Knees 5825* (E)) **B** fruits with persistent calyx, **C** habit (P. Baxter, Chile, *Baxter et al. DCI 1859* (E)). *Zuccagnia punctata* Cav. **D** flowers **E** fruits (I. Specogna, Flora mendocina (http://www.floramendocina.com.ar/), Mendoza, Argentina, *unvouchered*). *Hoffmannseggia arequipensis* Ulibarri **F** fruits with persistent calyx, and inflorescence (C. E. Hughes, Arequipa, Peru, *Hughes et al. 2342* (FHO)). *Hoffmannseggia minor* (Phil.) Ulibarri, **G** habit and inflorescence (G. P. Lewis, Bolivia, *unvouchered*). *Hoffmannseggia humilis* (Mart. & Galeotti) Hemsl. **H** fruit with persistent sepals (J. Neff, Puebla, Mexico, *unvouchered*). *Stenodrepanum bergii* Harms **I** habit **J** inflorescence **K** fruit (R. H. Fortunato, Argentina, *Fortunato 9144* (BAB)).

#### 20.1 Balsamocarpon brevifolium

Taxon classificationPlantaeFabalesLeguminosae

Clos

#### 21. Zuccagnia

Taxon classificationPlantaeFabalesLeguminosae

Cav., Icon. 5: 2. 1799

Figs 34D–E , 35

##### Type.

*Zuccagnia punctata* Cav.

##### Description.

Shrubs, 1–5 m. Stipules caducous. Leaves alternate, pinnate, (2–) 3–5 (– 6) cm long; leaflets in 5–13 subopposite pairs, elliptic-linear, rarely obovate, 4–14 × 1–3 mm, with glandular dots on both surfaces of the leaflet blades. Inflorescences terminal, erect racemes; bracts deltoid, glabrous, glandular, caducous. Flowers bisexual, zygomorphic; calyx comprising a hypanthium and 5 glabrous sepals, persistent after fruit develops, the lower sepal cucullate and covering the other four in bud; petals 5, free, yellow, obovate to broadly obovate, short-clawed, glandular trichomes on the dorsal surface of the petal blades; stamens 10, free, pubescent; ovary pilose. Fruit an ovoid-acute, oblique, laterally compressed, indehiscent (?), gall-like pod, on a short stipe and covered with long reddish brown bristles, c. 1 × 0.6 cm, 1-seeded.

##### Geographic distribution.

A monospecific genus restricted to Chile, NW and central-W Argentina.

##### Habitat.

Dry temperate upland and montane bushland and thickets on sandy plains.

##### Etymology.

Named by Cavanilles for the Italian physician, traveller and plant collector, Attilio Zuccagni (1754–1807).

##### References.

Burkart (1952: 184–185); Kiesling et al. (1994: 286); Ulibarri (2005, 2008); Nores et al. (2012).

**Figure 35.**
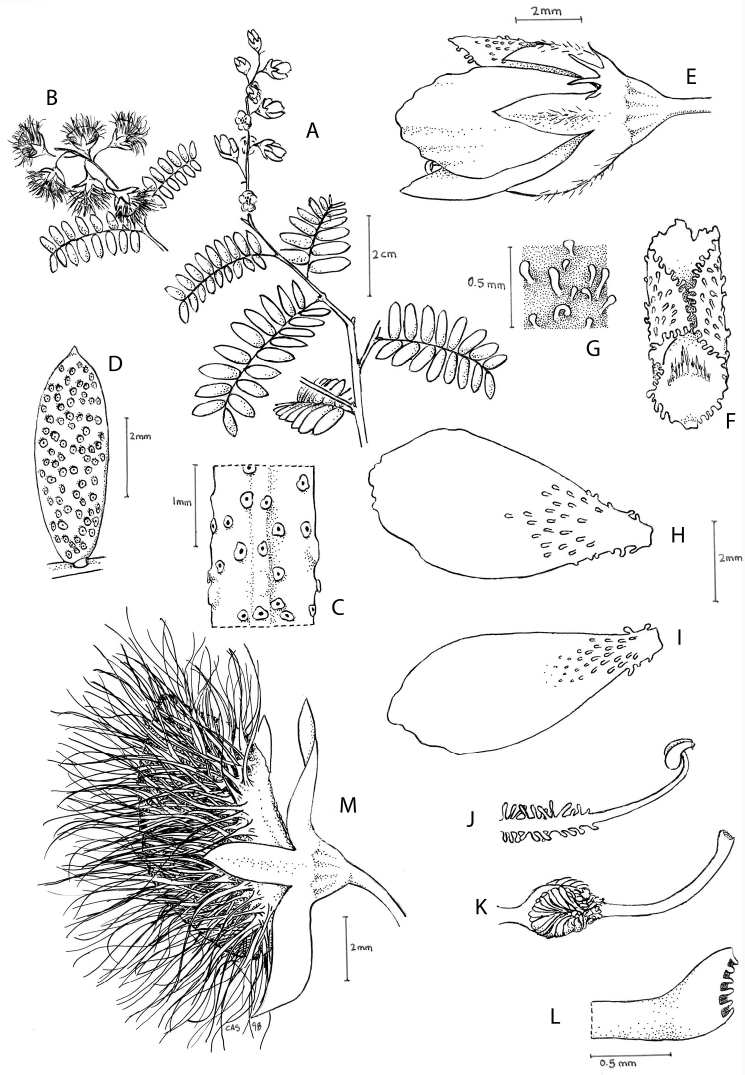
*Zuccagnia punctata* Cav.. **A** flowering branchlet **B** infructescence **C** stem section **D** leaflet **E** flower (unopened) **F** median petal **G** detail of petal glands **H** upper lateral petal **I** lower lateral petal **J** stamen **K** gynoecium **L** stigma **M** fruit. **A, D** from *Tinto* 2017 **B, M** from *Wingenroth et al.* 354 **C, E–L** from *Cabrera* 30149. Drawn by Christi A. Sobel.

#### 21.1 Zuccagnia punctata

Taxon classificationPlantaeFabalesLeguminosae

Cav.

#### 22. Stenodrepanum

Taxon classificationPlantaeFabalesLeguminosae

Harms, Notizbl. Bot. Gart. Berlin-Dahlem 7: 500. 1921

Figs 34I–K , 36

##### Type.

*Stenodrepanum bergii* Harms.

##### Description.

Suffrutescent shrub, (10–) 20–40 cm tall, with bud-bearing and occasionally tuber-forming roots; glabrous, with globose sessile glands scattered along the branches. Stipules ovate, membranous, 2.5–4 × 2–2.5 mm. Leaves alternate, bipinnate, pinnae in 1–3 pairs plus a single terminal pinna, 4–10 cm long; leaflets in 5–9 pairs per pinna, obtuse, 5–12 × 2–5.5 mm, with a crenulate, glandular margin, and embedded glands on the lower surface. Inflorescence a lax, terminal raceme, 4–14 cm long. Flowers bisexual, zygomorphic; calyx comprising a hypanthium and 5 sepals (these not persisting in fruit), glabrous, glandular, the lower cucullate sepal covering the other four in bud; petals 5, free, yellow, the median petal with red markings, obovate, with stipitate glands on the dorsal surface; stamens 10, free, filaments pubescent and glandular; ovary glandular. Fruit a linear to slightly falcate, cylindrical, torulose pod, 30–60 × 2–2.5 mm, 1–5-seeded. Seeds ovoid.

##### Geographic distribution.

A monospecific genus endemic to central and western Argentina.

##### Habitat.

Subtropical wooded grassland and scrub, especially close to salt pans.

##### Etymology.

From *steno*- (Greek: narrow) and *drepano*- (Greek: sickle), in allusion to the narrow sickle-shaped fruit.

##### References.

Ulibarri (1979, 2008); Kiesling et al. (1994: 285); Caponio et al. (2012); Nores et al. (2012).

**Figure 36.**
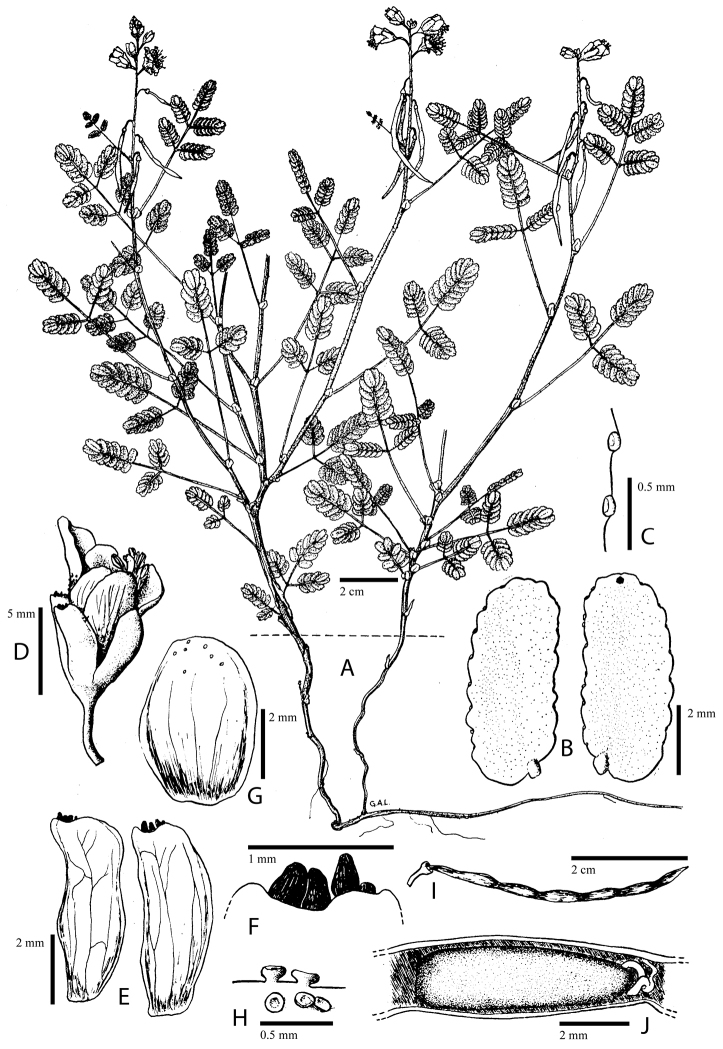
*Stenodrepanum bergii* Harms. **A** habit **B** leaflets **C** glands on the margin of the leaflets **D** flower **E** sepals with glands **F** apical glands of the sepals **G** lower cucullate sepal **H** glands on the petals **I** fruit **J** position of a seed in the fruit. **A**–**H** from *Piccini-Leguizamon* 1970 **I, J** from *Soriano* 787. **A** drawn by G. A. Larsen, **B–J** drawn by Emilio A. Ulibarri, originally published in *Darwiniana*, vol. 21 (nos. 2–4), page 402 (1978).

#### 22.1 Stenodrepanum bergii

Taxon classificationPlantaeFabalesLeguminosae

Harms

#### 23. Hoffmannseggia

Taxon classificationPlantaeFabalesLeguminosae

Cav., Icon. 4: 63. 1798

Figs 34F–H , 37

Larrea Ortega (1797), nom. rejec. against Larrea Cav. (1800) in the Zygophyllaceae.
Moparia Britton & Rose (1930).

##### Type.

*Hoffmannseggia falcaria* Cav., nom. illeg. = *Hoffmannseggia glauca* (Ortega) Eifert.

##### Description.

Perennial woody herbs, most species forming a basal rosette, or subshrubs to 3 m, unarmed, often arising from bud-bearing and tuberous roots, shoots pubescent and with gland-tipped trichomes. Stipules not seen. Leaves alternate, bipinnate, ending in a pair of pinnae plus a single terminal pinna (except for *Hoffmannseggia aphylla*); pinnae opposite, in 1-13 pairs; leaflets small and numerous, in 2–15 (– 18) pairs per pinna, glabrous to pubescent, and glandular. Inflorescences terminal or axillary racemes; bracts often caducous. Flowers bisexual, zygomorphic; calyx comprising a hypanthium and 5 sepals, these weakly imbricate, persistent as pods mature (except in *Hoffmannseggia microphylla* and *Hoffmannseggia peninsularis*, where they are not always persistent); petals 5, free, yellow to orange, the median petal often with red markings; stamens 10, free, filaments pubescent; ovary glabrous to pubescent, eglandular to glandular, stigma apical, concave. Fruit a laterally compressed, straight or sometimes falcate pod, the sutures almost parallel, papery to leathery, glabrous to pubescent, eglandular or with glandular trichomes, indehiscent or dehiscent, with twisting valves. Seeds compressed, ovoid.

##### Geographic distribution.

*Hoffmannseggia* comprises 25 taxa in 23 species and occupies a classical amphitropical distribution in the New World with 10 species restricted to North America (southern USA and Mexico), 12 in South America (Peru, Bolivia to south-central Argentina and Chile, mainly Andean), and one species (*Hoffmannseggia glauca* (Ortega) Eifert) widespread throughout the range of the genus.

##### Habitat.

Subtropical desert and semi-desert grassland, often in open areas and on disturbed sites, on sandy, rocky or calcareous soils.

##### Etymology.

Named by Cavanilles for the German botanist, entomologist and ornithologist, Johann Centurius Graf von Hoffmannsegg (1766–1849).

##### References.

Britton and Rose (1930, under *Larrea* and *Moparia*); Burkart (1936); Macbride (1943, under *Caesalpinia*); Ulibarri (1979, 1996); Simpson (1999); Simpson et al. (2004, 2005); Lewis (1998, see *Caesalpinia pumilio*: 171–173); Simpson and Ulibarri (2006); Lewis and Sotuyo (2010).

##### Notes.

A complete synopsis and key to species (except *Hoffmannseggia aphylla*) is available in Simpson and Ulibarri (2006). A list of accepted species is given below excluding types and synonymy, for which the reader should refer to Simpson and Ulibarri (2006).

**Figure 37.**
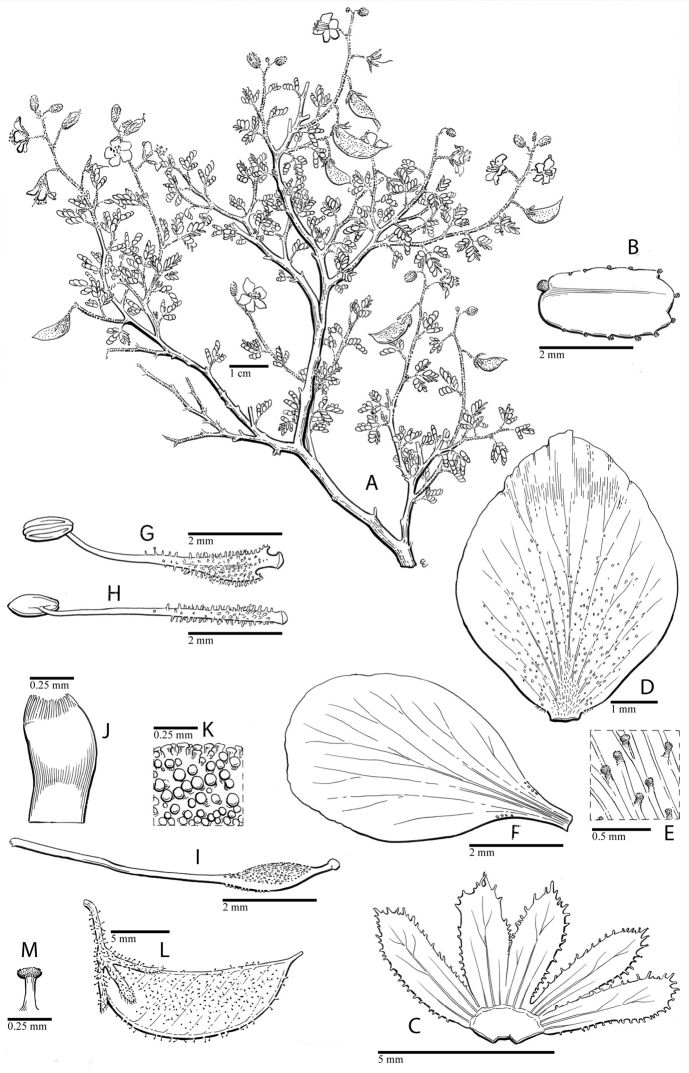
*Hoffmannseggia pumilio* (Griseb.) B. B. Simpson. **A** habit **B** median leaflet undersurface **C** calyx opened out **D** median petal **E** detail of glands on dorsal surface of median petal **F** lateral petal **G, H** stamens **I** gynoecium **J** stigma **K** detail of glands on ovary **L** fruit **M** gland on fruit. **A, L–M** from *Cabrera* 30150 **B–K** from *Venturi* 8309. Drawn by Eleanor Catherine.

#### 23.1 Hoffmannseggia aphylla

Taxon classificationPlantaeFabalesLeguminosae

(Phil.) G.P. Lewis & Sotuyo

#### 23.2 Hoffmannseggia arequipensis

Taxon classificationPlantaeFabalesLeguminosae

Ulibarri

#### 23.3 Hoffmannseggia doelli

Taxon classificationPlantaeFabalesLeguminosae

Phil.

#### 23.2.1 Hoffmannseggia doelli Phil. subsp. doellii

Taxon classificationPlantaeFabalesLeguminosae

#### 23.2.2 Hoffmannseggia doelli Phil. subsp. argentina

Taxon classificationPlantaeFabalesLeguminosae

Ulibarri

#### 23.4 Hoffmannseggia drepanocarpa

Taxon classificationPlantaeFabalesLeguminosae

A. Gray

#### 23.5 Hoffmannseggia drummondii

Taxon classificationPlantaeFabalesLeguminosae

Torr. & A. Gray

#### 23.6 Hoffmannseggia erecta

Taxon classificationPlantaeFabalesLeguminosae

Phil.

#### 23.7 Hoffmannseggia eremophila

Taxon classificationPlantaeFabalesLeguminosae

(Phil.) Burkart ex Ulibarri

#### 23.8 Hoffmannseggia glauca

Taxon classificationPlantaeFabalesLeguminosae

(Ortega) Eifert

#### 23.9 Hoffmannseggia humilis

Taxon classificationPlantaeFabalesLeguminosae

(Mart. & Galeotti) Hemsl.

#### 23.10 Hoffmannseggia intricata

Taxon classificationPlantaeFabalesLeguminosae

Brandegee

#### 23.11 Hoffmannseggia microphylla

Taxon classificationPlantaeFabalesLeguminosae

Torr.

#### 23.12 Hoffmannseggia minor

Taxon classificationPlantaeFabalesLeguminosae

(Phil.) Ulibarri

#### 23.13 Hoffmannseggia miranda

Taxon classificationPlantaeFabalesLeguminosae

Sandwith

#### 23.14 Hoffmannseggia oxycarpa

Taxon classificationPlantaeFabalesLeguminosae

Benth.

#### 23.14.1 Hoffmannseggia oxycarpa Benth. subsp. oxycarpa

Taxon classificationPlantaeFabalesLeguminosae

#### 23.14.2 Hoffmannseggia oxycarpa Benth. subsp. arida

Taxon classificationPlantaeFabalesLeguminosae

(Rose) B. B. Simpson

#### 23.15 Hoffmannseggia peninsularis

Taxon classificationPlantaeFabalesLeguminosae

(Britton) Wiggins

#### 23.16 Hoffmannseggia prostrata

Taxon classificationPlantaeFabalesLeguminosae

Lag. ex DC.

#### 23.17 Hoffmannseggia pumilio

Taxon classificationPlantaeFabalesLeguminosae

(Griseb.) B. B. Simpson

#### 23.18 Hoffmannseggia repens

Taxon classificationPlantaeFabalesLeguminosae

(Eastw.) Cockerell

#### 23.19 Hoffmannseggia tenella

Taxon classificationPlantaeFabalesLeguminosae

Tharp & L. P. Williams

#### 23.20 Hoffmannseggia trifoliata

Taxon classificationPlantaeFabalesLeguminosae

Cav.

#### 23.21 Hoffmannseggia viscosa

Taxon classificationPlantaeFabalesLeguminosae

(Ruiz & Pav.) Hook.

#### 23.22 Hoffmannseggia watsonii

Taxon classificationPlantaeFabalesLeguminosae

(Fisher) Rose

#### 23.23 Hoffmannseggia yaviensis

Taxon classificationPlantaeFabalesLeguminosae

Ulibarri

#### 24. Arquita

Taxon classificationPlantaeFabalesLeguminosae

E. Gagnon, G. P. Lewis & C. E. Hughes, Taxon 64(3): 479. 2015

Figs 38 , 39I–O

##### Type.

*Arquita mimosifolia* (Griseb.) E. Gagnon, G. P. Lewis & C. E. Hughes.

##### Description.

Small to medium-sized, often decumbent shrubs, 0.3–2.5 m in height, slender in stature, usually with glandular trichomes on various parts of the plant; young stems and inflorescence rachises red-orange to maroon. Stipules ovate-obovate to deltoid, chartaceous, 2.5–5.5 mm long, usually with a fimbriate-glandular margin and short-stalked glands (except in some specimens of *Arquita ancashiana*), caducous. Leaves bipinnate, with 1–5 pairs of pinnae, usually with a single terminal pinna; petiole (0.3–) 0.5–6 cm long; rachis 0.5–6 cm long (but sometimes absent); leaflets in 4–12 opposite pairs per pinna, oblong-obovate, 2.5–10 (– 14) × 1–3.5 (– 6) mm, often with maroon/black glands in depressions on crenulated leaflet margins, and sometimes with occasional sessile black glands on the undersurface of leaflet blades (in *Arquita ancashiana* the glands are submarginal on the lower half of the basal leaflets of the pinnae). Inflorescences leaf-opposed, determinate racemes (with only 1 to 2 flowers open at a given time), (5–) 7–21 (– 41.5) cm long; bracts lanceolate, acuminate, either eglandular or covered in gland-tipped trichomes, 2.75–7 mm long, caducous. Flowers bisexual, zygomorphic; calyx comprising a hypanthium, and 5 sepals, 6–11 mm long, caducous, the lower sepal cucullate, and sepals either have an entire or glandular-fimbriate margin; petals 5, free, yellow to orange, median petal, sometimes streaked red, 6–17 × 4–12 mm, claw pubescent at the base, either flat or inrolled, sometimes with stipitate-glandular trichomes on the dorsal surface of the whole petal, upper and lower lateral petals 6–17 × 3–12 mm; stamens 10, free, 5–13 mm long, anthers 0.75–2.3 mm long, the stamens deflexed and loosely grouped around the gynoecium; ovary usually covered with gland-tipped trichomes. Fruits laterally compressed, lunate-falcate pods with a marcescent style, covered sparsely to densely with gland-tipped trichomes, these sometimes dendritic, 2–4.7 × (0.7–) 0.9–1 cm. Seeds laterally compressed, ovate-orbicular, 4.5–6 × 3.5–4.5 × 1 mm, the testa shiny olive-grey, sometimes mottled or streaked black.

##### Geographic distribution.

The genus *Arquita* comprises six taxa in five species restricted to the Andes in South America, in disjunct inter-Andean valleys, in Ecuador, Peru, Bolivia and Argentina.

##### Habitat.

Seasonally dry, montane, rupestral habitats in inter-Andean valleys.

##### Etymology.

The name *Arquita* derives from the vernacular name of *Arquita trichocarpa* in Argentina (Ulibarri 1996).

##### Notes.

A revision of *Arquita* with a complete key to species is available in Gagnon et al. (Taxon 64(3): 468–490, 2015).

##### References.

Burkart (1936); Ulibarri (1996); Lewis (1998: 167–171, 174–179); Lewis et al. (2010); Gagnon et al. (2015: 468–490).

**Figure 38.**
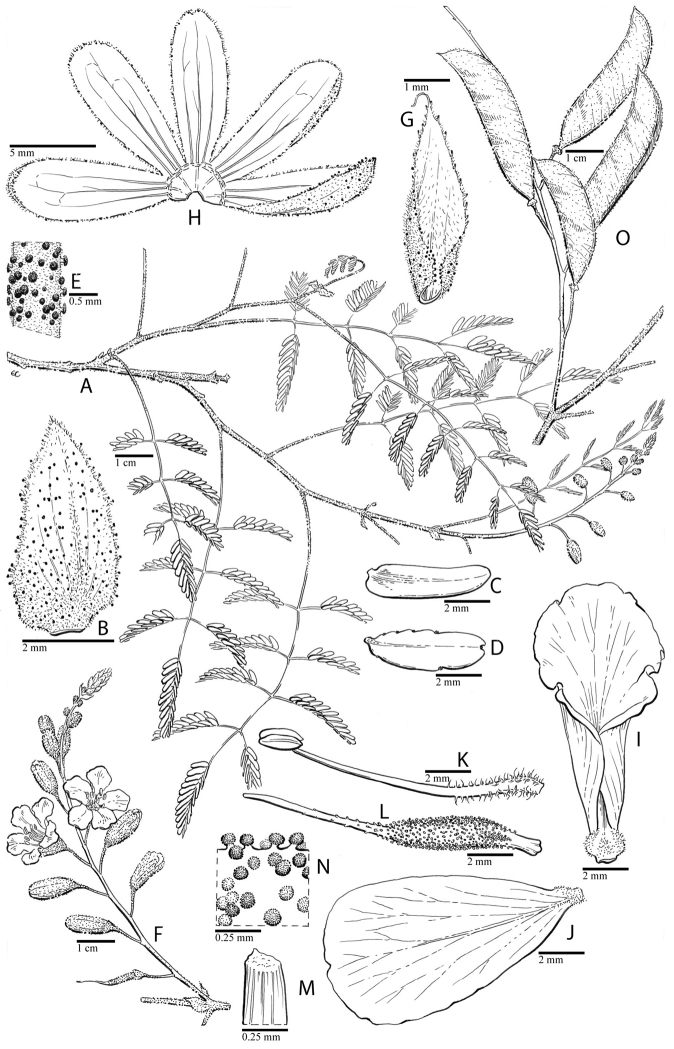
*Arquita mimosifolia* (Griseb.) E. Gagnon, G. P. Lewis & C. E. Hughes. **A** flowering branchlet **B** stipule **C** eglandular leaflet undersurface **D** glandular leaflet undersurface **E** detail of glands on stem **F** inflorescence **G** bract **H** calyx opened out **I** median petal **J** lateral petal **K** stamen **L** gynoecium **M** stigma **N** detail of glands on ovary **O** fruits. **A–E, G–N** from *Kiesling et al.* 4990 **F** from *Lorentz* s.n. **O** from *Schreiter* 68526. Drawn by Eleanor Catherine.

**Figure 39.**
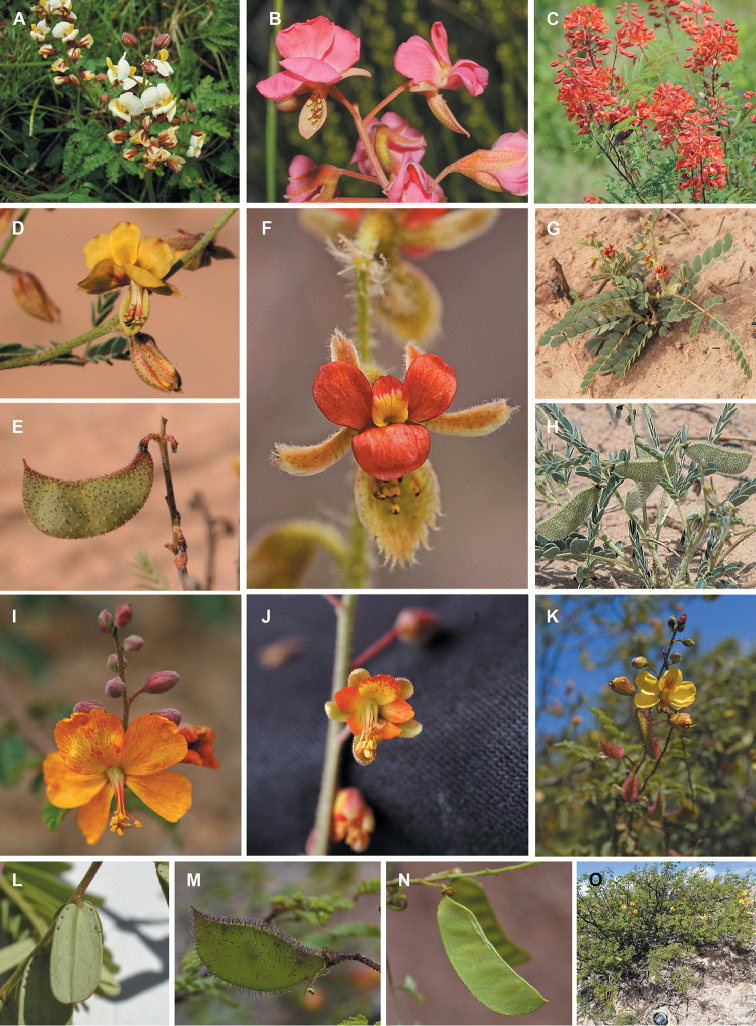
*Pomaria pilosa* (Vogel) B. B. Simpson & G. P. Lewis. **A** inflorescences (A. A. Schneider, Flora Digital (http://www.ufrgs.br/fitoecologia/florars/), Rio Grande do Sul, Brazil, *unvouchered*). *Pomaria rubicunda* (Vogel) B. B. Simpson & G. P. Lewis **B** flowers **C** inflorescences (S. Bordignon, Flora Digital (http://www.ufrgs.br/fitoecologia/florars/), Rio Grande do Sul, Brazil, *unvouchered*). *Pomaria jamesii* (Torr. & Gray) Walp. **D** flower **E** fruit (P. Alexander, SEINet Arizona Chapter (http://swbiodiversity.org/seinet/imagelib/), Arizona, USA, *unvouchered*); *Pomaria burchellii* (DC.) B. B. Simpson & G. P. Lewis subsp. burchellii (captions continued on next page) **F** flower **G** habit **H** fruits (O. Bourquin, Flora of Zimbabwe (http://www.zimbabweflora.co.zw/), Ghanzi district, Botswana, *unvouchered*). *Arquita grandiflora* E. Gagnon, G. P. Lewis & C. E. Hughes **I** flower and buds (C. E. Hughes, Ancash, Peru, *Särkinen et al. 2225* (FHO)). *Arquita celendiniana* (G. P. Lewis & C. E. Hughes) E. Gagnon, G. P. Lewis & C. E. Hughes **J** flower (E. Gagnon, Cajamarca, Peru, *Hughes & al. 3097* (MT)). *Arquita trichocarpa* (Griseb.) E. Gagnon, G. P. Lewis & C. E. Hughes **K** inflorescence **M** fruit (E. Gagnon, Salta, Argentina, *Gagnon & Atchison 218* (MT)) **O** habit (E. Gagnon, Jujuy, Argentina, *Gagnon et al. 204* (MT)). *Arquita ancashiana* (Ulibarri) E. Gagnon, G. P. Lewis & C. E. Hughes **L** undersurface of leaflet (E. Gagnon, Cajamarca, Peru, *Hughes et al. 3065* (MT)). *Arquita mimosifolia* (Griseb.) E. Gagnon, G. P. Lewis & C. E. Hughes **N** fruit (E. Gagnon, Salta, Argentina, *Gagnon et al. 203* (MT)).

#### 24.1 Arquita ancashiana

Taxon classificationPlantaeFabalesLeguminosae

(Ulibarri) E. Gagnon, G. P. Lewis & C. E. Hughes

#### 24.2 Arquita celendiniana

Taxon classificationPlantaeFabalesLeguminosae

(G. P. Lewis & C. E. Hughes) E. Gagnon, G. P. Lewis & C. E. Hughes

#### 24.3 Arquita grandiflora

Taxon classificationPlantaeFabalesLeguminosae

E. Gagnon, G. P. Lewis & C. E. Hughes

#### 24.4 Arquita mimosifolia

Taxon classificationPlantaeFabalesLeguminosae

(Griseb.) E. Gagnon, G. P. Lewis & C. E. Hughes

#### 24.5 Arquita trichocarpa

Taxon classificationPlantaeFabalesLeguminosae

(Griseb.) E. Gagnon, G. P. Lewis & C. E. Hughes

#### 24.5.1 Arquita trichocarpa (Griseb.) E. Gagnon, G. P. Lewis & C. E. Hughes var. trichocarpa

Taxon classificationPlantaeFabalesLeguminosae

#### 24.5.2 Arquita trichocarpa (Griseb.) E. Gagnon, G. P. Lewis & C. E. Hughes var. boliviana

Taxon classificationPlantaeFabalesLeguminosae

E. Gagnon, G. P. Lewis & C. E. Hughes

#### 25. Pomaria

Taxon classificationPlantaeFabalesLeguminosae

Cav., Icon. 5: 1. 1799

Figs 39A–H , 40

Melanosticta DC. (1825).
Cladotrichium Vogel (1837).

##### Type.

*Pomaria glandulosa* Cav.

##### Description.

Small shrubs, subshrubs or perennial herbs, with a moderate to dense indumentum of simple curled hairs, sometimes also scattered plumose trichomes, intermixed with sessile, oblate glands (drying black) on stems. Stipules laciniate, pubescent, glandular, persistent. Leaves alternate, bipinnate, pinnae in 1–8 (– 11) pairs plus a terminal pinna; leaflets small, in 2–16 (– 27) pairs per pinna, always with multiple sessile glands on their lower surface (these orange in the field, drying black). Inflorescence a terminal or axillary raceme; bracts caducous. Flowers bisexual, zygomorphic; calyx comprising a hypanthium and 5 lanceolate sepals, the lower sepal cucullate, covering the other 4 in bud, and closely embracing the androecium and gynoecium at anthesis, sepals not persistent in fruit; petals 5, free, yellow, white, red or pink; stamens 10, filaments pubescent; ovary sparsely to densely hairy and glandular, stigma lateral. Fruit a linear or sickle-shaped, laterally-compressed pod, apex acute, with a sparse to dense covering of plumose/dendritic or stellate trichomes (these sometimes obscure and restricted to the fruit margin) intermixed with sessile oblate glands (drying black), elastically dehiscent, with twisting valves. Seeds laterally compressed.

##### Geographic distribution.

A genus of 17 taxa in 16 species: nine in North America (south-eastern USA, central and northern Mexico), four in South America (south-eastern Brazil, Paraguay, and Argentina), and three in southern Africa (Namibia, Botswana and South Africa).

##### Habitat.

Mainly in subtropical dry grassland and in degraded sites, many on limestone.

##### Etymology.

Named by Cavanilles for Dominic Pomar, botanist from Valencia, and doctor to Philip III (1598–1621), King of Spain.

##### Notes.

Revisions of the species of *Pomaria* are available for North America (Simpson, 1998), South America and Africa (Simpson and Lewis 2003), and southern Africa (under the name *Hoffmannseggia*, Brummit and Ross 1974). A list of accepted species is given below, but excludes types and synonymy which are available in the aforementioned revisions.

##### References.

Burkart (1936: 86–90); Brummitt and Ross (1974, as *Hoffmannseggia*); Ulibarri (1996, 2008); Simpson (1998); Simpson and Lewis (2003); Simpson et al. (2006).

**Figure 40.**
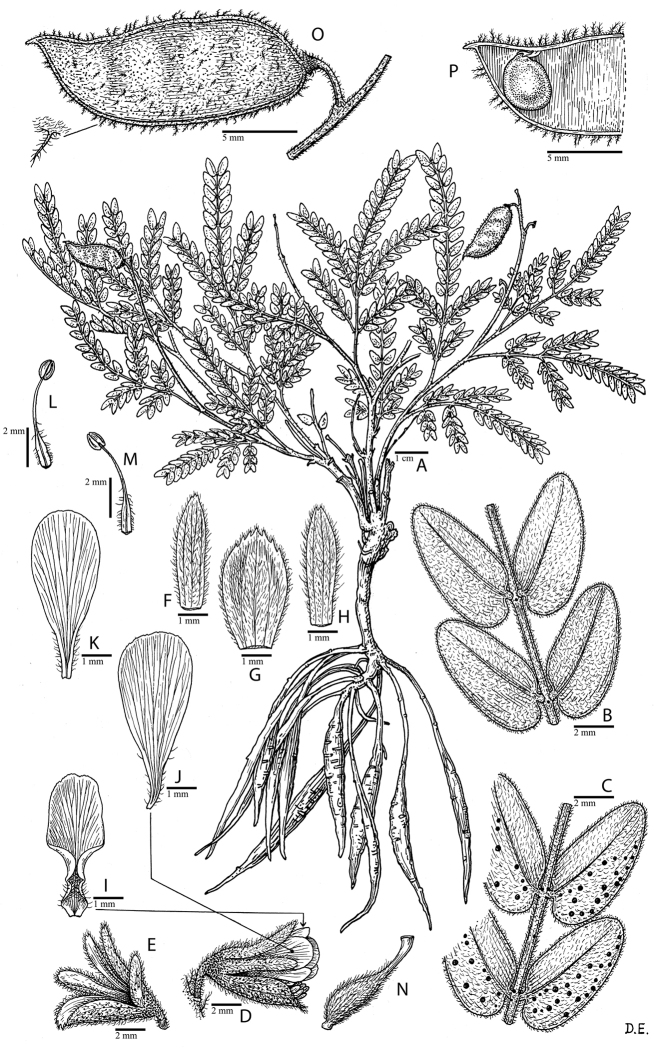
*Pomaria burchellii* (DC.) B. B. Simpson & G. P. Lewis subsp. burchellii. **A** habit **B, C** leaflets from above and beneath, respectively **D** flower **E** calyx **F–H** calyx lobes **I** median petal **J** upper lateral petal **K** lower lateral petal **L, M** stamens **N** gynoecium **O** fruit, with enlargement of single trichome **P** part of single fruit valve showing seed. **A–C, O, P** from *Wild & Drummond* 6913 **D–N** from *Galala* 72. Drawn by D. Erasmus, originally published in *Flora Zambesiaca*, vol. 3 part 2, page 185 (2007).

#### 25.1 Pomaria austrotexana

Taxon classificationPlantaeFabalesLeguminosae

B. B. Simpson

#### 25.2 Pomaria brachycarpa

Taxon classificationPlantaeFabalesLeguminosae

(A. Gray) B. B. Simpson

#### 25.3 Pomaria burchellii

Taxon classificationPlantaeFabalesLeguminosae

(DC.) B. B. Simpson & G. P. Lewis

#### 25.4 Pomaria canescens

Taxon classificationPlantaeFabalesLeguminosae

(Fisher) B. B. Simpson

#### 25.5 Pomaria fruticosa

Taxon classificationPlantaeFabalesLeguminosae

(S. Watson) B. B. Simpson

#### 25.6 Pomaria glandulosa

Taxon classificationPlantaeFabalesLeguminosae

Cav.

#### 25.7 Pomaria jamesii

Taxon classificationPlantaeFabalesLeguminosae

(Torr. & A. Gray) Walp.

#### 25.8 Pomaria lactea

Taxon classificationPlantaeFabalesLeguminosae

(Schinz) B. B. Simpson & G. P. Lewis

#### 25.9 Pomaria melanosticta

Taxon classificationPlantaeFabalesLeguminosae

S. Schauer

#### 25.10 Pomaria multijuga

Taxon classificationPlantaeFabalesLeguminosae

(S. Watson) B. B. Simpson

#### 25.11 Pomaria parviflora

Taxon classificationPlantaeFabalesLeguminosae

(Micheli) B. B. Simpson & G. P. Lewis

#### 25.12 Pomaria pilosa

Taxon classificationPlantaeFabalesLeguminosae

(Vogel) B. B. Simpson & G. P. Lewis

#### 25.13 Pomaria rubicunda

Taxon classificationPlantaeFabalesLeguminosae

(Vogel) B. B. Simpson & G. P. Lewis

#### 25.13.1 Pomaria rubicunda (Vogel) B. B. Simpson & G. P. Lewis var. rubicunda

Taxon classificationPlantaeFabalesLeguminosae

#### 25.13.2 Pomaria rubicunda (Vogel) B. B. Simpson & G. P. Lewis var. hauthalii

Taxon classificationPlantaeFabalesLeguminosae

(Harms) B. B. Simpson & G. P. Lewis

#### 25.14 Pomaria sandersonii

Taxon classificationPlantaeFabalesLeguminosae

(Harv.) B. B. Simpson & G. P. Lewis

#### 25.15 Pomaria stipularis

Taxon classificationPlantaeFabalesLeguminosae

(Vogel) B. B. Simpson & G. P. Lewis

#### 25.16 Pomaria wootonii

Taxon classificationPlantaeFabalesLeguminosae

(Britton) B. B. Simpson

#### 26 Erythrostemon

Taxon classificationPlantaeFabalesLeguminosae

Klotzsch, in Link, Klotzsch & Otto, Icon. Pl. Rar. Horti. Berol. 2: 97, t. 39. 1844, descr. emended E. Gagnon & G. P. Lewis

Figs 41 , 42

Poincianella Britton & Rose (1930), pro parte, including the type species Caesalpinia mexicana A. Gray = Poincianella mexicana (A. Gray) Britton & Rose.
Schrammia Britton & Rose (1930).

##### Diagnosis.

*Erythrostemon* is closely related to *Pomaria*, but differs in habit, consisting of large shrubs and small to medium sized trees, or occasionally suffrutices (vs. shrubs, suffrutices, or perennial herbs in *Pomaria*). It also differs by its ovate-lanceolate to orbicular sepals (vs. linear, laciniate sepals in *Pomaria*), leaflets that are either eglandular or with conspicuous black sessile glands along the margin, these sometimes sunken in the sinuses of the crenulated margin (vs. leaflets with multiple glandular dots on the lower leaflet surfaces, that are orange in the field, drying black), the androecium and gynoecium free from the calyx (vs. the androecium and gynoecium cupped in the lower cucullate sepal), deflexed petals (vs. the two lower petals forming a horizontal platform above the lower cucullate sepal), and oblong-elliptic pods, the valves chartaceous to slightly woody, glabrous to pubescent, eglandular or with stipitate glands (vs. linear to sickle-shaped pods, the valves glabrous or with plumose trichomes and stipitate glands).

##### Type.

*Erythrostemon gilliesii* (Hook.) Klotzsch.

##### Emended description.

Shrubs or small to medium-sized trees varying from (0.5–) 1–12 (– 20) meters tall, occasionally suffrutices (*Erythrostemon nelsonii* and *Erythrostemon caudatus*), unarmed (except *Erythrostemon glandulosus*); bark variable, smooth or rough, sometimes exfoliating, grey, greyish white, pale brown or reddish brown, often with white or black pustular lenticels; young stems terete (angular in *Erythrostemon angulatus*), glabrous to densely pubescent, eglandular to densely covered in stipitate-glands. Stipules ovate-lanceolate, ovate to orbicular, apex acute to acuminate, caducous (persistent in *Erythrostemon argentinus* and *Erythrostemon caudatus*). Leaves alternate, bipinnate, usually ending in a pair of pinnae plus a single terminal pinna; petioles (0.2–) 0.5–8 (– 10) cm long; rachis (0.5–) 1.2–14.5 (– 21.5) cm long, or lacking; petiole and rachis glabrous to densely pubescent, eglandular or covered in stipitate glands; pinnae in 1–6 (– 15) pairs, plus a terminal pinna (this occasionally lacking); leaflets in 2–13 (– 20) opposite pairs per pinna, size varying from a few mm in length and width (1.4–3 × 0.75–2 mm in *Erythrostemon exilifolius*), to 5.3 × 2.5 cm, elliptic, oblong-elliptic, obovate, ovate or sub-orbicular, leaflet blades eglandular or with conspicuous black sessile glands along the margin, these sometimes sunken in the sinuses of the crenulated margin. Inflorescence an axillary or terminal raceme. Flowers bisexual, zygomorphic; calyx a short hypanthium with 5 sepals, 4.5–25 mm long, glabrous to pubescent, eglandular or with stipitate-glands, lower sepal cucullate in bud, all sepals caducous, the hypanthium persistent and abscising to form a free ring around the pedicel as the fruit matures; petals 5, free, imbricate, bright golden yellow, to creamish yellow, salmon pink or pink-scarlet, the median petal often with red-orange markings, the corolla diverse in form, the median petal 6–32 × 3.2–20 mm, the lateral petals 6–32 × 3.5–18.5 mm, petal blades eglandular or the dorsal surface covered with stipitate glands, claw margins glabrous to pubescent, eglandular or with gland-tipped trichomes; stamens 10, free, 0.6–3.5 cm long (up to 10 cm in *Erythrostemon gilliesii*), filaments pubescent, eglandular or with stipitate glands; ovary pubescent, eglandular or with sessile or stipitate glands, stigma a terminal fringed chamber. Fruit a chartaceous to coriaceous or slightly woody, laterally compressed pod, with a marcescent style persisting as a small beak, elastically dehiscent with twisting valves, 2.4–12.5 × 1–2.8 cm, glabrous to pubescent, eglandular or with stipitate glands, (1–) 2–7 (– 8)-seeded. Seeds yellow to ochre-brown, or mottled with grey and black.

##### Geographic distribution.

The genus comprises 34 taxa in 31 species. Its circumscription is emended here to include many species previously placed in Central American and Mexican *Poincianella*. 22 species are found across the southern USA, Mexico and Central America, one occurs in the Caribbean (Cuba and Hispaniola), eight occur in South America, with one endemic in the caatinga vegetation of Brazil, and the other seven in Argentina, Bolivia, Chile, and Paraguay.

##### Habitat.

Low-elevation seasonally dry tropical forests across Mexico, Central America, the Caribbean and in caatinga vegetation in Brazil; also in patches of dry forest, deserts, yungas-puna transition zones, and chaco-transition forests in Argentina, Bolivia, Chile and Paraguay.

##### Etymology.

From *erythro*- (Greek: red) and *stemon* (Greek: stamen), the type species *Erythrostemon gilliesii* (Wall. ex Hook.) Klotzsch has long red exserted stamens, but this is unusual in the genus as circumscribed here.

##### Notes.

Species descriptions (under *Caesalpinia* binomials) are available in Lewis (1998). A key is also available in that revision, but it includes species now considered to belong in *Cenostigma*, *Arquita*, and *Hoffmannseggia*.

##### References.

Britton and Rose (1930); Burkart (1936: 82–84, 97–108); Ulibarri (1996); Lewis (1998); De Queiroz (2009: 120–121).

**Figure 41.**
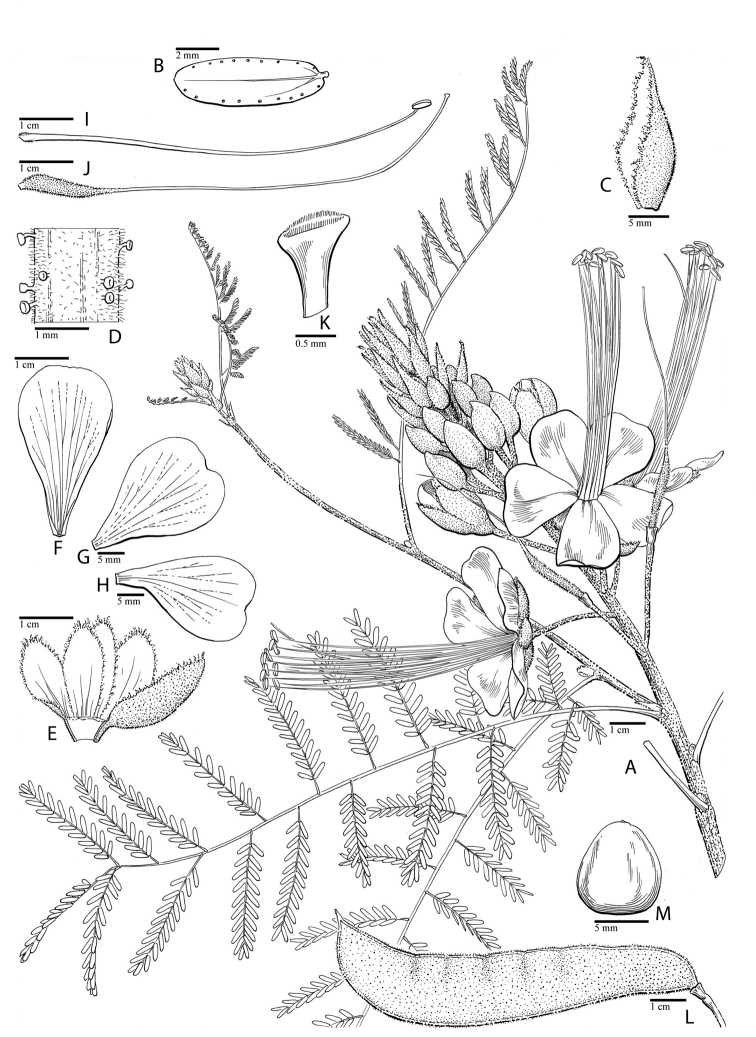
*Erythrostemon gilliesii* (Hook.) Klotzsch. **A** inflorescence and foliage **B** leaflet undersurface with submarginal glands **C** bract **D** detail of glandular pedicel **E** calyx opened out **F** median petal **G** upper lateral petal **H** lower lateral petal **I** stamen **J** gynoecium **K** stigma **L** fruit **M** seed. **A** from *Venturi* 5365 **B, L** from *Kiesling et al.* 4891 **C–K** from Cult. Kew 213-69 01878 **M** from *Lewis* 1417. Drawn by Eleanor Catherine.

**Figure 42.**
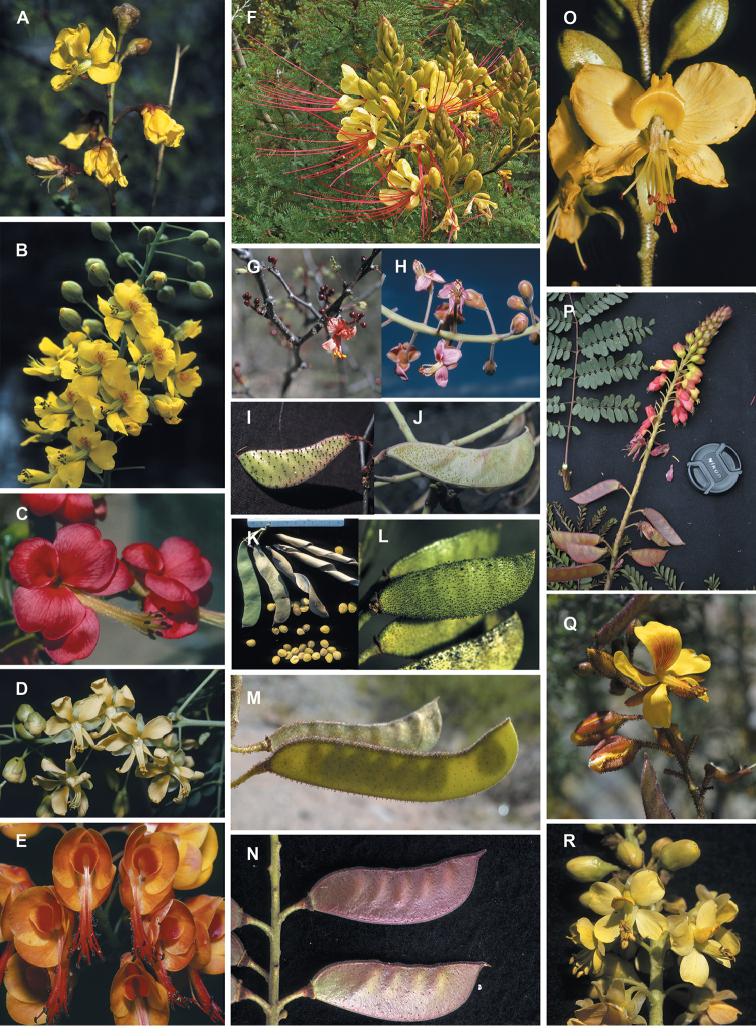
*Erythrostemon placidus* (Brandegee) E. Gagnon & G. P. Lewis. **A** flowers (C. E. Hughes, Baja California, Mexico, *Lewis 2031* (K)). *Erythrostemon mexicanus* (A. Gray) E. Gagnon & G. P. Lewis **B** inflorescence (C. E. Hughes, San Luís Potosí, Mexico, *Hughes et al. 1606* (FHO)). *Erythrostemon coccineus* (G. P. Lewis & J. L. Contr.) E. Gagnon & G. P. Lewis **C** flowers (C. E. Hughes, Oaxaca, Mexico, *Lewis et al. 1802* (K)). *Erythrostemon pannosus* (Brandegee) E. Gagnon & G. P. Lewis (captions continued on next page) **D** (G. P. Lewis, cultivated in University of Texas from seeds collected in Mexico, *B. L. Turner 88* (TEX)). *Erythrostemon exostemma* (DC.) E. Gagnon & G. P. Lewis **E** flowers (G. P. Lewis, Comayagua, Honduras, *Lewis & Hughes 1709* (K)). *Erythrostemon gilliesii* (Hook.) Klotzsch **F** Inflorescences (Stan Shebs, Wikicommons (https://commons.wikimedia.org/wiki/File:Caesalpinia_gilliesii_2.jpg), Nevada, U.S.A., *unvouchered*). *Erythrostemon melanadenius* (Rose) E. Gagnon & G. P. Lewis **G** inflorescence **I** fruit (C. E. Hughes, Oaxaca, Mexico, *Hughes et al. 2091* (FHO)). *Erythrostemon hintonii* (Sandwith) E. Gagnon & G. P. Lewis **H** inflorescence **J** fruit (G. P. Lewis, Mexico, *MacQueen et al. 428* (K)). *Erythrostemon hughesii* (G. P. Lewis) E. Gagnon & G. P. Lewis **K** unripe, ripe and dehisced fruits and seeds (C.E. Hughes, Oaxaca, Mexico, *Lewis et al. 1795* (K)). *Erythrostemon nicaraguensis* (G. P. Lewis) E. Gagnon & G. P. Lewis **L** fruits (C. E. Hughes, Esteli, Nicaragua, *Hawkins et al. 4* (FHO)). *Erythrostemon exilifolius* (Griseb.) E. Gagnon & G. P. Lewis **M** fruits (E. Gagnon, Argentina, *Gagnon et al. 203* (MT)) **Q** flower and buds (E. Gagnon, Catamarca, Argentina, *Gagnon & Atchison 222* (MT)). *Eythrostemon fimbriatus* (Tul.) E. Gagnon & G. P. Lewis **N** fruits (C. E. Hughes, La Paz Bolivia, *Hughes et al. 2441* (FHO)). Erythrostemon cf. fimbriatus (Tul.) E. Gagnon & G. P. Lewis **R** flowers (C. E. Hughes, Santa Cruz, Bolivia, *Hughes et al. 2466* (FHO)). *Erythrostemon calycinus* (Benth) L.P. Queiroz **O** flower (G. P. Lewis, Bahia, Brazil, *unvouchered)*. *Erythrostemon coulterioides* (Griseb. emend. Burkart) E. Gagnon & G. P. Lewis **P** leaves, inflorescence with flowers and developing fruits (E. Gagnon, Jujuy, Argentina, *Gagnon & Atchison 209* (MT).

#### 26.1 Erythrostemon acapulcensis

Taxon classificationPlantaeFabalesLeguminosae

(Standl.) E. Gagnon & G. P. Lewis comb. nov.

urn:lsid:ipni.org:names:77158083-1

##### Basionym.

*Caesalpinia acapulcensis* Standl., Contr. U.S. Natl. Herb. 20: 213. 1919.

*Poincianella acapulcensis* (Standl.) Britton & Rose, N. Amer. Fl. 23(5): 329. 1930.

##### Type.

MEXICO, Guerrero, vicinity of Acapulco, Oct 1894– Mar 1895, *Palmer 505* (holotype US!; isotypes F!, GH!, K!, MEXU!, NY!).

#### 26.2 Erythrostemon angulatus

Taxon classificationPlantaeFabalesLeguminosae

(Hook. & Arn.) E. Gagnon & G. P. Lewis comb. nov.

urn:lsid:ipni.org:names:77158084-1

##### Basionym.

Zuccagnia ? angulata Hook. & Arn., Bot. Beechy’s Voyage: 22. 1830.

*Caesalpinia angulata* (Hook. & Arn.) Baill., Adansonia 9: 227. 1870.

##### Type.

CHILE, Coquimbo (holotype ?E, n.v.).

*Caesalpinia angulicaulis* Clos, Fl. Chile: 223. 1846.

Type. CHILE, Coquimbo, Andacollo, near the Rio Hurtado, 1837, *C. Gay 525* (holotype ?TL, n.v.; isotype SGO).

#### 26.3 Erythrostemon argentinus

Taxon classificationPlantaeFabalesLeguminosae

(Burkart) E. Gagnon & G. P. Lewis comb. nov.

urn:lsid:ipni.org:names:77158085-1

##### Basionym.

*Caesalpinia argentina* Burkart, Revista Argent. Agron. 3: 105. 1936.

##### Type.

ARGENTINA, Jujuy, Santa Cornelia, Sierra de Santa Bárbara, Nov 1911, *Spegazzini 2159* (holotype LP, isotype SI).

*Caesalpinia coulterioides* Griseb. Symb. Fl. Argent.: 113. 1879, pro parte.

#### 26.4 Erythrostemon caladenia

Taxon classificationPlantaeFabalesLeguminosae

(Standl.) E. Gagnon & G. P. Lewis comb. nov.

urn:lsid:ipni.org:names:77158086-1

##### Basionym.

*Caesalpinia caladenia* Standl., Contr. U.S. Natl. Herb. 20: 214. 1919.

*Poincianella caladenia* (Standl.) Britton & Rose, N. Amer. Fl. 23(5): 329. 1930.

##### Type.

MEXICO, Sonora, c. 5 miles below Minas Nuevas, 12 Mar 1910, *Rose et al. 12660* (holotype US!; isotype NY!).

#### 26.5 Erythrostemon calycinus

Taxon classificationPlantaeFabalesLeguminosae

(Benth.) L. P. Queiroz, in Leguminosas da Caatinga: 121. 2009, as "calycina"

##### Basionym.

*Caesalpinia calycina* Benth., Mart., Fl. Brasil. 15(2): 71. 1870.

##### Type.

BRAZIL, Bahia, near Rio de Contas, Mar 1817, *Prinz zu Wied-Neuwied* (Princeps Maximilianus Neovidensis) *s.n.* (holotype BR!).

#### 26.6 Erythrostemon caudatus

Taxon classificationPlantaeFabalesLeguminosae

(A. Gray) E. Gagnon & G. P. Lewis comb. nov.

urn:lsid:ipni.org:names:77158090-1

##### Basionym.

*Hoffmannseggia caudata* A. Gray, Boston J. Nat. Hist. 6: 179. 1850.

*Caesalpinia caudata* (A. Gray) E. M. Fisher, Bot. Gaz. 18: 123. 1893.

*Schrammia caudata* (A. Gray) Britton & Rose, N. Amer. Flora 23(5): 317. 1930.

##### Type.

U. S. A., Texas, between the Nueces and the Rio Grande, *Wright 146* (holotype GH; isotype K!).

#### 26.7 Erythrostemon coccineus

Taxon classificationPlantaeFabalesLeguminosae

(G. P. Lewis & J. L. Contr.) E. Gagnon & G. P. Lewis comb. nov.

urn:lsid:ipni.org:names:77158120-1

##### Basionym.

*Caesalpinia coccinea* G. P. Lewis & J. L. Contr., Kew Bull. 49: 103. 1994.

##### Type.

MEXICO, Oaxaca State, 27 Mar 1989, *Lewis et al. 1802* (holotype MEXU!; isotypes FCME!, FHO!, K!, M!, NY!, SI!).

#### 26.8 Erythrostemon coluteifolius

Taxon classificationPlantaeFabalesLeguminosae

(Griseb.) E. Gagnon & G. P. Lewis comb. nov.

urn:lsid:ipni.org:names:77158091-1

##### Basionym.

*Caesalpinia coluteifolia* Griseb., Symb. Fl. Argent.: 111. 1879.

##### Type.

Argentina, Tucumán, near El Alduralde on the route to Salta, Feb 1873, *Lorentz & Hieronymus 1004* (holotype GOET!; isotype CORD).

#### 26.9 Erythrostemon coulterioides

Taxon classificationPlantaeFabalesLeguminosae

(Griseb. emend. Burkart) E. Gagnon & G. P. Lewis comb. nov.

urn:lsid:ipni.org:names:77158119-1

##### Basionym.

*Caesalpinia coulterioides* Griseb., Symb. Fl. Argent: 113. 1879, (as “*coulteriodes*”), pro parte quoad material from El Volcan.

##### Type.

ARGENTINA, Jujuy, Depto. Tumbaya, El Volcán, 12–13 May 1873, *Lorentz & Hieronymus 760* (holotype GOET; isotype CORD).

*Caesalpinia coulterioides* Griseb., emend. Burkart, Revista Argent. Agron. 3: 97. 1936.

#### 26.10 Erythrostemon epifanioi

Taxon classificationPlantaeFabalesLeguminosae

(J. L. Contr.) E. Gagnon & G. P. Lewis comb. nov.

urn:lsid:ipni.org:names:77158121-1

##### Basionym.

*Caesalpinia epifanioi* J. L. Contr., Anales Inst. Biol. Univ. Nac. Auton. Mexico, Bot. 58: 55. 1989.

##### Type.

MEXICO, Guerrero, Mpio. Mártires de Cuéllar, 18 Feb. 1986, *Contreras 1825* (holotype FCME; isotype MEXU).

#### 26.11 Erythrostemon exilifolius

Taxon classificationPlantaeFabalesLeguminosae

(Griseb.) E. Gagnon & G. P. Lewis comb. nov.

urn:lsid:ipni.org:names:77158092-1

##### Basionym.

*Caesalpinia exilifolia* Griseb., Plant. Lorentz: 80. 1874.

##### Type.

ARGENTINA, Catamarca, near San José, 4 Jan 1872, *Lorentz 352* (holotype GOET!).

#### 26.12 Erythrostemon exostemma

Taxon classificationPlantaeFabalesLeguminosae

(DC.) E. Gagnon & G. P. Lewis comb. nov.

urn:lsid:ipni.org:names:77158093-1

##### Basionym.

*Caesalpinia exostemma* DC., Prodr. 2: 483. 1825.

*Poincianella exostemma* (DC.) Britton & Rose, N. Amer. Fl. 23(5): 328. 1930.

##### Type.

MEXICO, a painting, one of the copies of Ic. Fl. Mex. 80, represented at G-DC by de Candolle plate 218.

#### 26.12.1 Erythrostemon exostemma subsp. exostemma

Taxon classificationPlantaeFabalesLeguminosae

? Poinciana compressa Sessé & Mociño ex. G. Don, Gen. Hist. 2: 433 (1832).
? Caesalpinia compressa (G. Don) D. Dietr. Syn. Pl. 2:1494. 1840. Type. MEXICO, *Sessé & Mociño*, formerly in herb. Lambert– not located in recent times, but a specimen in the Sessé & Mociño herbarium (MA), no. 1097, labelled Poinciana compressa, represents Caesalpinia exostemma according to P. Standley (fide McVaugh, 1987).
Caesalpinia affinis Hemsl., Diag. Pl. Nov. Mexic. 8. 1878.
Poincianella affinis (Hemsl.) Britton & Rose, N. Amer. Fl. 23(5): 328. 1930. Type. GUATEMALA, *Skinner s.n.* (holotype K!; isotype K!).
Poinciana conzattii Rose, Contr. U.S. Natl. Herb. 13: 303. 1911.
Poincianella conzattii (Rose) Britton & Rose, N. Amer. Fl. 23(5): 328. 1930.
Caesalpinia conzattii (Rose) Standl., Trop. Woods 37: 34. 1934. Type. MEXICO, Tehuantepec, 1909, *Hugo & Conzatti 2444* (holotype US!, national herbarium number 841055).

#### 26.12.2 Erythrostemon exostemma subsp. tampicoanus

Taxon classificationPlantaeFabalesLeguminosae

(Britton & Rose) E. Gagnon & G. P. Lewis comb. nov.

urn:lsid:ipni.org:names:77158117-1

##### Basionym.

*Poincianella tampicoana* Britton & Rose, N. Amer. Fl. 23(5): 330. 1930.

*Caesalpinia tampicoana* (Britton & Rose) Standl., Publ. Field Mus. Nat. Hist., Bot. Ser. 11(5): 159. 1936.

Caesalpinia exostemma subsp. tampicoana (Britton & Rose) G. P. Lewis, Caesalpinia: Revis. Poincianella-Erythrostemon group: 72. 1998.

##### Type.

MEXICO, Veracruz, vicinity of Pueblo Viejo, 2 km S of Tampico, 1 and 2 Jun 1910. *Palmer 556* (holotype US!).

#### 26.13 Erythrostemon fimbriatus

Taxon classificationPlantaeFabalesLeguminosae

(Tul.) E. Gagnon & G. P. Lewis comb. nov.

urn:lsid:ipni.org:names:60473354-2

##### Basionym.

*Caesalpinia fimbriata* Tul., Arch. Mus. Hist. Nat. Paris 4: 145. 1844.

##### Type.

BOLIVIA, “Chivesivi, Vallé S de La Paz, alt. 8500–12000 ped. angl.”, *Pentland 39* (holotype P!; isotype F!).

*Caesalpinia bangii* Rusby, Mem. Torrey Bot. Club 3(3): 22. 1893.

Type. BOLIVIA, 1891, *Bang 757* (holotype NY!; isotypes E!, F!, GH!, K!).

*Caesalpinia cromantha* Burkart, Revista Argent. Agron. 3(2): 100. 1936.

Type. ARGENTINA, Prov. Salta, Depto. Guachipas, Pampa Grande, Jan 1897, *Spegazzini 2198* (holotype SI!; isotype LP).

#### 26.14 Erythrostemon gilliesii

Taxon classificationPlantaeFabalesLeguminosae

(Hook.) Klotzsch, Ic. Pl. Rar. Horti. Berol. 2 (3): 97, t. 39. 1844

##### Basionym.

*Poinciana gilliesii* Wall. ex Hook., Bot. Misc. 1: 129. 1829 [1830].

*Caesalpinia gilliesii* (Hook.) D. Dietr., Synop. Pl. 2: 1495. 1840.

##### Type.

ARGENTINA, near Rio Quatro and Rio Quinto, and in La Punta de San Luis, *Gillies s.n.* (holotype K!).

#### 26.15 Erythrostemon glandulosus

Taxon classificationPlantaeFabalesLeguminosae

(Bertero ex DC.) E. Gagnon & G. P. Lewis comb. nov.

urn:lsid:ipni.org:names:77158102-1

##### Basionym.

*Caesalpinia glandulosa* Bertero ex DC., Prodr. 2: 482. 1825.

*Poincianella glandulosa* (Bertero ex DC.) Britton & Rose, N. Amer. Fl. 23(5): 336. 1930.

##### Type.

HISPANIOLA, *Bertero* 84 (holotype G-DC).

#### 26.16 Erythrostemon hintonii

Taxon classificationPlantaeFabalesLeguminosae

(Sandwith) E. Gagnon & G. P. Lewis comb. nov.

urn:lsid:ipni.org:names:77158103-1

##### Basionym.

*Caesalpinia hintonii* Sandwith. Kew Bull. 1937: 303. 1937.

##### Type.

MEXICO, Guerrero, District of Coyuca, Cuajilote, 9 May 1935, *Hinton 7746* (holotype K!; isotypes A!, F!, GH!, MEXU).

#### 26.17 Erythrostemon hughesii

Taxon classificationPlantaeFabalesLeguminosae

(G. P. Lewis) E. Gagnon & G. P. Lewis comb. nov.

urn:lsid:ipni.org:names:60473355-2

##### Basionym.

*Caesalpinia hughesii* G. P. Lewis, Caesalpinia: Revis. Poincianella-Erythrostemon group: 73. 1998.

##### Type.

MEXICO, Oaxaca, 5 km W of Rio Grande, 25 Mar 1989, *Lewis et al. 1795* (holotype K!; isotypes FCME!, FHO!, K!, MEXU!).

#### 26.18 Erythrostemon laxus

Taxon classificationPlantaeFabalesLeguminosae

(Benth.) E. Gagnon & G. P. Lewis comb. nov.

urn:lsid:ipni.org:names:77158104-1

##### Basionym.

*Caesalpinia laxa* Benth., Pl. Hartw.: 60. 1840.

*Poincianella laxa* (Benth.) Britton & Rose, N. Amer. Flora 23(5): 329. 1930.

##### Type.

MEXICO, Oaxaca, Teojomulco, *Hartweg 455* (holotype BM!; isotypes E!, K!, MEXU!, photos F!).

#### 26.19 Erythrostemon macvaughii

Taxon classificationPlantaeFabalesLeguminosae

(J. L. Contr. & G. P. Lewis) E. Gagnon & G. P. Lewis comb. nov.

urn:lsid:ipni.org:names:77158111-1

##### Basionym.

*Caesalpinia macvaughii* J. L. Contr. & G. P. Lewis, Kew Bull. 47: 309. 1992.

##### Type.

MEXICO, Guerrero, Mpio. Zirándaro de Chávez, 8 Mar 1988, *Contreras 2343* (holotype FCME; isotypes K!, MEXU).

*Caesalpinia laxa* sensu McVaugh, pro parte quoad *McVaugh 22517*, non Benth.

#### 26.20 Erythrostemon melanadenius

Taxon classificationPlantaeFabalesLeguminosae

(Rose) E. Gagnon & G. P. Lewis comb. nov.

urn:lsid:ipni.org:names:60473356-2

##### Basionym.

*Poinciana melanadenia* Rose, Contr. U.S. Natl. Herb. 13: 303. 1911.

*Caesalpinia melanadenia* (Rose) Standl., Contr. U.S. Natl. Herb. 23: 425. 1922.

*Poincianella melanadenia* (Rose) Britton & Rose, N. Amer. Flora 23(5): 334. 1930.

##### Type.

MEXICO, Puebla, near Tehuacán, 1 Sep 1906, *Rose & Rose 11249* (holotype US!).

#### 26.21 Erythrostemon mexicanus

Taxon classificationPlantaeFabalesLeguminosae

(A. Gray) E. Gagnon & G. P. Lewis comb. nov.

urn:lsid:ipni.org:names:60473357-2

##### Basionym.

*Caesalpinia mexicana* A. Gray, Proc. Amer. Acad. Arts 5: 157. 1861.

*Poinciana mexicana* (A. Gray) Rose, Contr. U.S. Natl. Herb. 13: 303. 1911.

*Poincianella mexicana* (A. Gray) Britton & Rose, N. Amer. Fl. 23(5): 330. 1930.

##### Type.

MEXICO, Nuevo León, near Monterrey, 11 Feb 1847, *Gregg s.n.* (lectotype GH!, *fide* McVaugh, 1987).

#### 26.22 Erythrostemon nelsonii

Taxon classificationPlantaeFabalesLeguminosae

(Britton & Rose) E. Gagnon & G. P. Lewis comb. nov.

urn:lsid:ipni.org:names:77158112-1

##### Basionym.

*Poincianella nelsonii* Britton & Rose in N. Amer. Fl. 23(5): 331. 1930.

*Caesalpinia nelsonii* (Britton & Rose) J. L. Contr., Thesis, UNAM, Mexico D.F.: 91. 1991.

##### Type.

MEXICO, Guerrero, between Copala and Juchitango [Juchitan], 9 Feb 1895, *Nelson 2303* (holotype US!; isotypes GH!, NY!, photo MEXU).

#### 26.23 Erythrostemon nicaraguensis

Taxon classificationPlantaeFabalesLeguminosae

(G. P. Lewis) E. Gagnon & G. P. Lewis comb. nov.

urn:lsid:ipni.org:names:60473358-2

##### Basionym.

*Caesalpinia nicaraguensis* G. P. Lewis, Caesalpinia: Revis. Poincianella-Erythrostemon group: 86. 1998.

##### Type.

NICARAGUA, Department of Esteli, *Hughes 1406* (holotype MEXU!; isotypes EAP, FHO, K!, NY!).

#### 26.24 Erythrostemon oyamae

Taxon classificationPlantaeFabalesLeguminosae

(Sotuyo & G. P. Lewis) E. Gagnon & G. P. Lewis comb. nov.

urn:lsid:ipni.org:names:60473359-2

##### Basionym.

*Caesalpinia oyamae* Sotuyo & G. P. Lewis, Brittonia 59: 34. 2007.

##### Type.

MEXICO, Puebla, Mpio. Acatlán de Osorio, 20 km to the W of Acatlán on the road from Oaxaca City to Izúcar de Matamoros (Hwy. 190), 18°17'N, 98°5'W, 19 Feb 1993, *J. A. Hawkins & C. E. Hughes 23* (holotype MEXU; isotypes FHO!, K!, MEXU).

#### 26.25 Erythrostemon palmeri

Taxon classificationPlantaeFabalesLeguminosae

(S. Watson) E. Gagnon & G. P. Lewis comb. nov.

urn:lsid:ipni.org:names:77158105-1

##### Basionym.

*Caesalpinia palmeri* S. Watson, Proc. Am. Acad. Arts 24: 47. 1889.

*Poinciana palmeri* (S. Wats.) Rose, Contr. U.S. Natl. Herb. 13: 303. 1911.

*Poincianella palmeri* (S. Watson) Britton & Rose, N. Amer. Flora 23(5): 332. 1930.

##### Type.

MEXICO, Sonora, Guaymas, Jun 1887, *Palmer 70* (holotype US!; isotypes GH!, K!, NY!).

*Poincianella arida* Britton & Rose, N. Amer. Flora 23 (5): 332. 1930.

*Caesalpinia arida* (Britton & Rose) Wiggins, Contr. Dudley Herb. 3(3): 69. 1940.

Type. MEXICO, Sonora, near Hermosillo, 7 Mar 1910, *Rose et al. 12508* (holotype NY!).

#### 26.26 Erythrostemon pannosus

Taxon classificationPlantaeFabalesLeguminosae

(Brandegee) E. Gagnon & G. P. Lewis comb. nov.

urn:lsid:ipni.org:names:77158113-1

##### Basionym.

*Caesalpinia pannosa* Brandegee, Proc. Calif. Acad. Sci., Ser. 2: 150. 1889. (See also Proc. Calif. Acad. Sci., Ser. 3: 130. 1891).

*Poinciana pannosa* (Brandegee) Rose, Contr. U.S. Natl. Herb. 13: 303. 1911.

*Poincianella pannosa* (Brandegee) Britton & Rose, N. Amer. Flora 23(5): 331. 1930.

##### Type.

MEXICO, Baja California, San Gregoria, 1 Feb 1889, *Brandegee s.n.* (lectotype UC!, designated by Lewis 1998).

Caesalpinia mexicana A. Gray var. californica A. Gray, Proc. Amer. Acad. Arts 5: 157. 1861.

*Poinciana californica* (A. Gray) Rose, Contr. U.S. Natl. Herb. 13: 303. 1911.

*Caesalpinia californica* (A. Gray) Standl., Contr. U.S. Natl. Herb. 23: 426. 1922.

*Poincianella californica* (A. Gray) Britton & Rose, N. Amer. Flora 23(5): 331. 1930.

Type. MEXICO, Baja California, Cape St. Lucas, Aug 1859– Jan 1860, *Xantus 29* (lectotype GH!, designated by Lewis 1998; isolectotype NY!).

*Caesalpinia arenosa* Wiggins, Contr. Dudley Herb. 3(3): 68. 1940.

Type. MEXICO, Baja California, 4 miles S of Guadalupe, 21 Mar 1935, *Whitehead 839* (holotype DS).

#### 26.27 Erythrostemon phyllanthoides

Taxon classificationPlantaeFabalesLeguminosae

(Standl.) E. Gagnon & G. P. Lewis comb. nov.

urn:lsid:ipni.org:names:77158106-1

##### Basionym.

*Caesalpinia phyllanthoides* Standl., Contr. U.S. Natl. Herb. 23: 425. 1922. *Poincianella phyllanthoides* (Standl.) Britton & Rose, N. Amer. Fl. 23(5): 332. 1930.

##### Type.

MEXICO, Tamaulipas, Hacienda Buena Vista, 18 Jun 1919, *Wooton s.n.* (holotype US!; isotype NY!).

#### 26.28 Erythrostemon placidus

Taxon classificationPlantaeFabalesLeguminosae

(Brandegee) E. Gagnon & G. P. Lewis comb. nov.

urn:lsid:ipni.org:names:77158114-1

##### Basionym.

*Caesalpinia placida* Brandegee, Proc. Calif. Acad. Sci., Ser. 2, 3: 131. 1891.

*Poinciana placida* (Brandegee) Rose, Contr. U.S. Natl. Herb. 13: 303. 1911.

*Poincianella placida* (Brandegee) Britton & Rose, N. Amer. Fl. 23(5): 331. 1930.

##### Type.

MEXICO, Baja California, La Paz, 4 Feb 1890, *Brandegee s.n.* (lectotype UC!, designated by Lewis 1998; isolectotype GH!).

#### 26.29 Erythrostemon robinsonianus

Taxon classificationPlantaeFabalesLeguminosae

(Britton & Rose) E. Gagnon & G. P. Lewis comb. nov.

urn:lsid:ipni.org:names:77158116-1

##### Basionym.

*Poincianella robinsoniana* Britton & Rose, N. Amer. Fl. 23(5): 330. 1930.

*Caesalpinia robinsoniana* (Britton & Rose) G. P. Lewis, Caesalpinia: Revis. Poincianella-Erythrostemon group: 42. 1998.

##### Type.

MEXICO, Jalisco, Zapotlán, 25 May 1893, *Pringle 5467* (holotype GH!; isotype MEXU!).

Caesalpinia mexicana A. Gray var. pubescens B.L. Rob. & Greenm., Proc. Amer. Acad. Arts 29: 386. 1894.

Type. As above.

#### 26.30 Erythrostemon standleyi

Taxon classificationPlantaeFabalesLeguminosae

(Britton & Rose) E. Gagnon & G. P. Lewis comb. nov.

urn:lsid:ipni.org:names:77158115-1

##### Basionym.

*Poincianella standleyi* Britton & Rose, N. Amer. Fl. 23(5): 330. 1930.

*Caesalpinia standleyi* (Britton & Rose) Standl., Publ. Field Mus. Nat. Hist., Bot. Ser. 11(5): 159. 1936.

##### Type.

MEXICO, Nayarit, Acaponeta, 9 Apr 1910, *Rose et al. 14190* (holotype NY!).

#### 26.31 Erythrostemon yucatanensis

Taxon classificationPlantaeFabalesLeguminosae

(Greenm.) E. Gagnon & G. P. Lewis comb. nov.

urn:lsid:ipni.org:names:77158107-1

##### Basionym.

*Caesalpinia yucatanensis* Greenm., Publ. Field Mus. Nat. Hist., Bot. Ser. 2: 252. 1907.

*Poincianella yucatanensis* (Greenm.) Britton & Rose, N. Amer. Fl. 23(5): 330. 1930.

##### Type.

MEXICO, Yucatán, near Izamal, 1895, *Gaumer 371* (holotype F!; isotypes F!, K!, NY!).

#### 26.31.1 Erythrostemon yucatanensis subsp. yucatanensis

Taxon classificationPlantaeFabalesLeguminosae

Caesalpinia recordii Britton & Rose, Trop. Woods 7: 6. 1926.
Poincianella recordii (Britton & Rose) Britton & Rose, N. Amer. Fl. 23(5): 329. 1930. Type. BELIZE, Feb 1926, *Record s.n.* (holotype US; isotypes F!, GH!, NY!).

#### 26.31.2 Erythrostemon yucatanensis subsp. chiapensis

Taxon classificationPlantaeFabalesLeguminosae

(G. P. Lewis) E. Gagnon & G. P. Lewis comb. nov.

urn:lsid:ipni.org:names:77158108-1

##### Basionym.

Caesalpinia yucatanensis subsp. chiapensis G. P. Lewis, Caesalpinia: Revis. Poincianella-Erythrostemon group: 85. 1998.

##### Type.

MEXICO, Chiapas, c. 4 km from Comalapa on road to La Trinitaria, 27 Feb 1992, *Hughes et al. 1684* (holotype K (sheet 2)!, isotypes E!, FHO!, K!, MEXU!, MO!, NY!).

#### 26.31.3 Erythrostemon yucatanensis subsp. hondurensis

Taxon classificationPlantaeFabalesLeguminosae

(G. P. Lewis) E. Gagnon & G. P. Lewis comb. nov.

urn:lsid:ipni.org:names:77158109-1

##### Basionym.

Caesalpinia yucatanensis subsp. hondurensis G. P. Lewis, in Caesalpinia: Revis. Poincianella-Erythrostemon group: 86. 1998.

##### Type.

HONDURAS, Dept. Yoro, lower Aguán Valley, c. 31 km W of Olanchito, 25 Mar 1991, *Hughes 1448* (holotype K!; isotype FHO!).

#### 27. ? Ticanto

Taxon classificationPlantaeFabalesLeguminosae

Adans., Fam. Pl. 2: 319. 1763.

Fig. 24G–H

?Ticanto Adans., Fam. Pl. 2: 319. 1763.
Caesalpinia sect. Nugaria DC. 1825.

##### Type.

“H.M. 6: t. 19” (= Rheede`s Hortus Malabaricus 6, plate 19, 1686).

##### Notes.

More work is needed to determine whether the species listed below form a clade and merit reinstatement as a distinct genus, or alternatively if the name *Ticanto* should be synonymised under another genus in the Caesalpinia group. The list of species presented below includes names that most probably belong in *Ticanto*, but revisionary and phylogenetic work are needed to accurately delimit species, and determine types and synonyms.

##### References.

Hattink (1974); Vidal and Hul Thol (1976); Chen et al. (2010a).

#### 27.1 Caesalpinia caesia

Taxon classificationPlantaeFabalesLeguminosae

Handel-Mazzetti

#### 27.2 Caesalpinia chinensis

Taxon classificationPlantaeFabalesLeguminosae

Roxb.

#### 27.3 Caesalpinia crista

Taxon classificationPlantaeFabalesLeguminosae

L. emend. Dandy & Exell

#### 27.4 Caesalpinia elliptifolia

Taxon classificationPlantaeFabalesLeguminosae

S. J. Li, Z. Y. Chen & D. X. Zhang

#### 27.5 Caesalpinia hypoglauca

Taxon classificationPlantaeFabalesLeguminosae

Chun & How

#### 27.6 Caesalpinia kwangtungensis

Taxon classificationPlantaeFabalesLeguminosae

Merr.

#### 27.7 Caesalpinia laevigata

Taxon classificationPlantaeFabalesLeguminosae

Perr.

#### 27.8 Caesalpinia magnifoliolata

Taxon classificationPlantaeFabalesLeguminosae

Metcalf

#### 27.9 Caesalpinia nuga

Taxon classificationPlantaeFabalesLeguminosae

(L.) Ait.

#### 27.10 Caesalpinia paniculata

Taxon classificationPlantaeFabalesLeguminosae

(Lam.) Roxb.

#### 27.11 Caesalpinia rhombifolia

Taxon classificationPlantaeFabalesLeguminosae

J. E. Vidal

#### 27.12 Caesalpinia scandens

Taxon classificationPlantaeFabalesLeguminosae

Heyne ex Roth

#### 27.13 Caesalpinia szechuanensis

Taxon classificationPlantaeFabalesLeguminosae

Craib

#### 27.14 Caesalpinia vernalis

Taxon classificationPlantaeFabalesLeguminosae

Champion

#### 27.15 Caesalpinia yunnanensis

Taxon classificationPlantaeFabalesLeguminosae

S. J. Li, D. X. Zhang & Z. Y. Chen

## Authors’ contributions

EG, AB, CEH and GPL were involved in study conception and design; EG, AB, CEH, GPL and LPdQ collected and provided herbarium and field samples for analysis; EG generated and assembled all the data, which she was also responsible for analysing and interpreting; EG drafted the manuscript, and critical revisions were provided by AB, CEH, GPL and LPdQ; EG also wrote the key, generic descriptions and provided the list of species belonging to each genus. These were all critically revised by GPL, who completed this work by verifying the nomenclature and identifying types for all species names and synonyms. GPL was also the main instigator behind the new generic names (*Paubrasilia*, *Hultholia*, *Hererolandia* and *Gelrebia*).

## Appendix 1

**Table:** Accessions included in this study. Species of the Caesalpinia group **are classified sensu Lewis (2005)**, and the number of species sampled over the total number of species recognized in the genus is given in parentheses. Type species for genera in the Caesalpinia group are preceded by an asterisk (*). Collector names, numbers (and herbarium acronym) of voucher specimens are listed for all material that was taken from herbarium specimens and for the voucher specimens of seed collections and silica-dried leaf samples, if known. Collection locality indicates the country where the specimen originated, and indicates which accessions were from cultivated specimens; N/A indicates that locality data was not available. Accession numbers are provided for published sequences downloaded directly from GenBank; with the exception of 22 sequences for the species *Caesalpinia crista*, *Caesalpinia decapetala*, *Caesalpinia sappan*, *Cenostigma gardnerianum*, *Coulteria platyloba*, *Guilandina bonduc*, *Libidibia coriaria*, *Poincianella exostemma*, *Poincianella bracteosa*, *Poincianella pyramidalis* and *Pterolobium stellatum*, the majority of the sequences come from the following published studies: Bruneau et al. (2001), Simpson et al. (2003), Haston et al. (2005), Simpson et al. (2005), Marazzi et al. (2006), Simpson et al. (2006), Marazzi and Sanderson (2010), Babineau et al. (2013), Gagnon et al. (2013), and Gagnon et al. (2015). Furthermore, certain accessions were combined together in phylogenetic analyses: these accessions are in bold. If there were redundant sequences between these combined accessions, the longest sequence for each available marker were selected (GenBank numbers in bold).

Genus (no. of species sampled/total no. species) Species	Voucher specimen (Herbarium)	Collection locality	*rps16*	*trnD-trnT*	*ycf6-psbM*	*ITS*	*trnL-trnF*	*matK-3’trnK*
**OUTGROUP**								
*Cassia javanica* L.	Fougère-Danezan 6 (MT)	Singapore, cultivated	KF522255	KX379272	KX372932	KX372778	EU361782	EU361910
***Colvillea racemosa* Bojer ex Hook.**	Haston V200303 (RNG)	Madagascar	**AY899794**	-	-	-	**AY899739**	-
***Colvillea racemosa* Bojer ex Hook.**	Bruneau 1397 (MT)	Madagascar	-	**KX379275**	-	**KX372928**	-	**KX176814**
***Conzattia multiflora* (B.L. Rob.) Standl.**	Du 600 (K), Haston V200303 (RNG)	Mexico	**AY899785**	-	-	-	-	-
***Conzattia multiflora* (B.L. Rob.) Standl.**	Hughes 1815 (NY)	Mexico	-	**KX379270**	-	**KX372927**	-	-
***Conzattia multiflora* (B.L. Rob.) Standl.**	Simpson 17-XI-97 (TEX)	Mexico	-	-	-	-	**AF430770**	-
***Conzattia multiflora* (B.L. Rob.) Standl.**	Werling 399 (ASU)	Mexico	-	-	-	-	-	**AY386918**
***Gymnocladus chinensis* Baill.**	Herendeen II-V-02-1 (US)	USA, cultivated	**KF522308**	**KX379269**	**KX372931**	**KX372777**	**AY232786**	-
***Gymnocladus chinensis* Baill.**	Herendeen 8-V-2003-1 (US)	USA, cultivated	-	-	-	-	-	**AY386928**
***Pterogyne nitens* Tul.**	Pennington 244 (FHO)	Brazil	**AY899747**	-	-	-	**AY899689**	-
***Pterogyne nitens* Tul.**	Herendeen 13-XII-97-1 (US)	Tanzania	-	**KX379276**	**KX372936**	**KX372782**	-	**EU362031**
***Senna covesii* (A. Gray) H.S. Irwin & Barneby**	Marazzi BM297 (ARIZ)	USA, cultivated	**HM236885**	-	-	-	-	-
***Senna covesii* (A. Gray) H.S. Irwin & Barneby**	Wojciechowski 876 (ASU)	USA	-	-	-	**KX372779**	**EU361835**	**AY386850**
***Senna spectabilis* (DC.) H.S. Irwin & Barneby**	Marazzi et al. BM029 (PY, CTES, Z)	Paraguay	**AM086983**	-	-	-	-	**AM086900**
***Senna spectabilis* (DC.) H.S. Irwin & Barneby**	Herendeen & Pooma 24-IV-99-6 (US)	Thailand	-	**KX379274**	**KX372934**	**KX372781**	-	-
*Senna alata* (L.) Roxb.	Bruneau 1076 (MT)	Cameroun, cultivated	-	KX379273	KX372933	KX372780	AF365091	EU362042
***Tetrapterocarpon geayi* Humbert**	Noyes 1049 (K)	Madagascar	**AY899742**	-	-	-	**AY899684**	-
***Tetrapterocarpon geayi* Humbert**	Bruneau & Ranaivojaona 1395 (WAG)	Madagascar	-	**KX379271**	**KX372935**	**KX372783**	-	**GU321972**
**CAESALPINIA GROUP**								
***Arquita*** Gagnon et al. (5/5 species)								
**Arquita mimosifolia* (Griseb.) E. Gagnon et al.	Gagnon et al. EG203 (MT)	Argentina	KF522160	KP003760	KP003707	KP003654	-	KX176810
**Arquita mimosifolia* (Griseb.) E. Gagnon et al.	Gagnon & Atchison EG211 (MT)	Argentina	KF522159	KP003759	KP003706	KP003653	-	KX176809
**Arquita mimosifolia* (Griseb.) E. Gagnon et al.	Särkinen et al. 2006 (FHO)	Argentina	KF522161	KP003761	KP003708	KP003655	KX373124	KX176837
**Arquita mimosifolia* (Griseb.) E. Gagnon et al.	Chumley 7387 (TEX)	Argentina	-	-	-	AY549893	AY535818-AY535805	-
*Arquita ancashiana* (Ulibarri) E. Gagnon et al.	Hughes et al. 3021 (MT, Z)	Peru	KF522164	KP003747	KP003696	KP003643	-	KX176804
*Arquita ancashiana* (Ulibarri) E. Gagnon et al.	Hughes et al. 3070 (MT, Z)	Peru	KF522167	KP003749	KP003698	KP003644	-	-
*Arquita ancashiana* (Ulibarri) E. Gagnon et al.	Lewis & Klitgaard 2266 (K)	Ecuador	KF522170	KP003753	KP003792	KP003647	KX373114	-
*Arquita celendiniana* (G.P. Lewis & C.E. Hughes) E. Gagnon et al.	Hughes et al. 2210 (FHO)	Peru	KF522148	KP003756	KP003703	KP003650	KX373092	KX176805
*Arquita celendiniana* (G. P. Lewis & C.E. Hughes) E. Gagnon et al.	Hughes et al. 3097 (MT, Z)	Peru	KF522149	KP003757	KP003704	KP003651	-	KX176823
*Arquita celendiniana* (G. P. Lewis & C.E. Hughes) E. Gagnon et al.	Hughes et al. 3102 (MT, Z)	Peru	KF522147	KO003758	KP003705	KP003652	-	KX176824
*Arquita celendiniana* (G.P. Lewis & C.E. Hughes)	Pennington 17567 (E)	Peru	-	-	-	KX372914	-	-
Arquita trichocarpa (Griseb.) E. Gagnon et al. var. trichocarpa	Lewis & Klitgaard 2166 (K)	Argentina	KF522163	KP003762	KP003709	KP003659	AF430740	KX176828
Arquita trichocarpa var. boliviana E. Gagnon et al.	Hughes et al. 2442 (FHO)	Bolivia	KF522162	KP003764	KP003711	KP003657	-	KX176833
*Arquita grandiflora* E. Gagnon et al.	Särkinen et al. 2225 (FHO)	Peru	KF522151	KP003763	KP003710	KP003656	-	KX176811
***Balsamocarpon*** Clos (1/1 species)								
**Balsamocarpon brevifolium* Clos	Baxter et al. DCI 1869 (E)	Chile	KF522135	KP003801	KP003743	KP003689	EU361739.	KX176815
**Balsamocarpon brevifolium* Clos	Taylor 745 (K)	Chile	KF522136	KX379415	KX373043	KX372915	-	-
**Balsamocarpon brevifolium* Clos	Coccuci & Sérsic 365 (CORD)	Chile	-	-	-	AY308548	JX219457	AF430761
***Caesalpinia*** L. sensu Lewis (2005; 21/~25 species)								
**Caesalpinia brasiliensis* L.	Léonard & Léonard 13904 (US, K)	Haiti	KF522092	KX379366	KX373030	KX372861	-	-
*Caesalpinia anacantha* Urb.	Liogier 16639 (P)	Dominican Republic	KX373127	KX379263	-	KX372859	-	-
*Caesalpinia bahamensis* Lam.	Baker B27 (K)	Bahamas	KF522091	KX379367	-	KX372862	-	-
*Caesalpinia bahamensis* Lam.	Michael 8975 (MEXU)	Bahamas	KF522093	-	-	-	-	-
*Caesalpinia barahonensis* Urb.	Ekman 5965 (K)	Haiti	KF522094	KX379365	-	KX372860	-	-
*Caesalpinia bracteata* Germish.	van Hoepen 2018 (K)	South Africa	KF522258	KX379345	KX372952	KX372784	-	-
*Caesalpinia buchii* Urb.	Acevedo-Rodriguez et al. 8522 (K, US)	Dominican Republic	KF522115	KX379341	KX373021	KX372870	-	-
*Caesalpinia buchii* Urb.	Ekman 8491 (K)	Haiti	-	KX379258	-	-	-	-
*Caesalpinia cassioides* Willd.	Hughes et al. 2023 (FHO)	Peru	KF522097	KX379358	KX373036	KX372855	-	-
*Caesalpinia cassioides* Willd.	Hughes et al. 2228 (FHO)	Peru	KF522098	KX379359	KX373035	KX372856	-	-
*Caesalpinia cassioides* Willd.	Hughes et al. 2641 (FHO)	Peru	KF522095	KX379360	KX373033	KX372857	-	-
*Caesalpinia cassioides* Willd.	Pennington et al. 789 (E)	Peru	KF522096	KX379361	KX373034	KX372858	KX373102	-
*Caesalpinia cassioides* Willd.	Lewis et al. 3281 (K)	Ecuador	-	-	-	-	AF430711	-
*Caesalpinia dauensis* Thulin	Gilbert et al. 7695 (K)	Ethiopia	KF522266	KX379346	KX372950	KX372788	-	-
*Caesalpinia erianthera* Chiov.	Friis et al. 4698 (K)	Somalia	KF522123	KX379333	KX373023	KX372878	-	-
*Caesalpinia erianthera* Chiov.	Radcliffe-Smith 5518 (K)	Oman	KF522122	KX379335	KX373026	KX372877	-	-
*Caesalpinia erianthera* Chiov.	Thulin & Mohamed 6941 (K)	Somalia	KF522125	KX379332	KX373024	KX372879	-	-
Caesalpinia erianthera Chiov. var. erianthera	Thulin 5557 (K)	Somalia	KF522118	-	-	-	-	-
Caesalpinia erianthera var. pubescens Brenan	Boulos et al. 17000 (K)	Yemen	KF522117	KX379334	KX373025	KX372876	-	-
*Caesalpinia glandulosopedicellata* R. Wilczek	Bamps & Malaisse 8647 (K)	Zaire	KF522261	KX379343	KX372953	KX372787	KX373118	KX176838
***Caesalpinia madagascariensis* (R.Vig) Senesse**	Bruneau 1348 (MT)	Madagascar	**KF522119**	**KX379330**	**KX373027**	**KX372874**	-	**KX176834**
***Caesalpinia madagascariensis* (R.Vig) Senesse**	Lewis et al. 2158 (K)	Madagascar	KF522120	KX379331	KX373028	KX372875	**KX373096**	-
*Caesalpinia nipensis* Urb.	Marie-Victorin et al. 21500 (MT)	Cuba	-	KX379414	-	-	-	-
*Caesalpinia nipensis* Urb.	Marie-Victorin et al. 21509 (MT)	Cuba	KX373129	KX379267	-	KX372865	-	-
*Caesalpinia nipensis* Urb.	Lewis 1838 (K)	Cuba	KX373128	KX379413	KX372980	KX372864	-	KX176835
*Caesalpinia oligophylla* Harms.	Hassan 70 (FHO, K)	Somalia	KF522262	KX379348	-	KX372786	-	-
*Caesalpinia pauciflora* (Griseb.) C. Wright	Ekman 9703 (K)	Cuba	KF522124	KX379338	KX373020	KX372872	-	-
*Caesalpinia pauciflora* (Griseb.) C. Wright	Liogier & Liogier 20521 (NY)	Hispaniola	KF522116	KX379340	KX373022	-	KX373097	-
*Caesalpinia pauciflora* (Griseb.) C. Wright	Lewis 1854 (K)	Cuba, cultivated	-	KX379339	-	KX372873	-	-
*Caesalpinia pulcherrima* (L.) Sw.	Cox 1, RBG Liv.Coll. 1975-3028 (K)	United Kingdom, cultivated	KF522174	-	-	-	-	-
*Caesalpinia pulcherrima* (L.) Sw.	Fougère-Danezan 19 (MT)	Singapore, cultivated	KF522172	KX379363	KX373031	KF379227	KX373109	KX176820
*Caesalpinia pulcherrima* (L.) Sw.	Lewis & Hughes 1715 (K)	Guatemala	KF522171	KX379362	-	KX372853	AF430733	-
*Caesalpinia pulcherrima* (L.) Sw.	Montreal Botanical Garden 7089-92 (MT)	Canada, cultivated	KF522173	KX379364	KX373032	KX372854	-	-
*Caesalpinia reticulata* Britton	Pollard et al. 1295 (K)	Turks & Caicos Islands	KX373130	KX379368	-	KX372863	-	-
*Caesalpinia rosei* Urb.	Ekman H13620 (K, TEX)	Dominican Republic	KX373131	KX379342	-	KX372871	AF430735	-
*Caesalpinia rostrata* N.E. Br.	ILC6-5 (PRE)	South Africa, cultivated	KX373132	KX379259	KX372951	KX372785	KX373116	-
*Caesalpinia rubra* (Engl.) Brenan	de Winter 3164 (K)	South Africa	KF522260	-	-	-	-	-
*Caesalpinia rubra* (Engl.) Brenan	Oshikoto 1917BD (K)	Namibia	KF522259	-	-	-	-	-
***Caesalpinia sessilifolia* S. Watson**	Palmer 533 (K, MO)	Mexico	**KX373133**	**KX379336**	**KX373018**	**KX372868**	-	-
***Caesalpinia sessilifolia* S. Watson**	Neff 8-24-91-4 (TEX)	Mexico	-	-	-	-	**AF430737**	-
*Caesalpinia sessilifolia* S. Watson	Hinton 24737 (MEXU)	Mexico	KF522121	-	-	-	-	-
*Caesalpinia stuckertii* Hassl.	Beck 9443 (NY)	Bolivia	KF522126	KX379337	KX373019	KX372869	KX373095	-
*Caesalpinia stuckertii* Hassl.	Krapovickas 4626 (K)	Argentina	KF522127	-	-	-	-	-
Caesalpinia trothae subsp. erlangeri (Harms) Brenan	Beckett & White 1711 (K)	Somalia	KF522263	KX379349	KX372948	KX372789	-	KX176812
Caesalpinia trothae subsp. erlangeri (Harms) Brenan	Thulin & Warfa 5816 (K)	Somalia	KF522267	KX379344	-	-	-	-
Caesalpinia trothae subsp. erlangeri (Harms) Brenan	Vollesen & Hassan 4873 (K)	Somalia	KF522264	-	-	-	-	-
Caesalpinia trothae Harms subsp. trothae	Bidgood et al. 559 (K)	Tanzania	KF522265	KX379350	KX372949	-	KX373117	KX176829
Caesalpinia trothae Harms subsp. trothae	Gillett 21088 (K)	Kenya	-	KX379347	-	KX372790	-	-
***Cenostigma*** Tul. (2/2 species)								
**Cenostigma macrophyllum* Tul.	Coradin et al. 6306 (K)	Brazil, Bahia	KF522053	KX379446	KX372981	-	-	-
**Cenostigma macrophyllum* Tul.	Thomas 9615 (K)	Brazil, Piaui	KF522069	-	KX372991	-	-	-
**Cenostigma macrophyllum* Tul.	de Queiroz 9147 (HUEFS)	Brazil, Bahia	KF522037	KX379447	KX372982	-	-	-
**Cenostigma macrophyllum* Tul.	Ferreira et al. 6371 (MBG)	From GenBank	-	-	-	-	JX073262	-
*Cenostigma tocantinum* Ducke	Klitgaard & de Lima 88 (K)	Brazil, cultivated	KF522071	KP003803	KP003740	KP003694	-	KX176806
*Cenostigma tocantinum* Ducke	Klitgaard s.n. (INPA)	Brazil	KF522070	KX379453	KX372992	KX372835	-	-
*Cenostigma gardnerianum* Tul. (synonym of *Cenostigma macrophyllum*)	(retrieved from GenBank)	Brazil	-	-	-	DQ787400	-	-
***Cordeauxia*** Hemsl. (1/1 species)								
**Cordeauxia edulis* Hemsl.	Gillett & Beckett 23305 (K)	Somalia	KF522083	-	-	-	-	-
**Cordeauxia edulis* Hemsl.	Hassan 232 (FHO, K)	Somalia	AY899748	-	-	-	AY899690	-
**Cordeauxia edulis* Hemsl.	Kuchar 17803 (K)	Somalia	KF522084	KX379430	KX372998	KX372826	EU361787	EU361920
**Cordeauxia edulis* Hemsl.	Annable & Collins 3541 (NY)	Hawaii, cultivated	-	-	-	-	AF430771	-
***Coulteria*** Kunth (7/9-10 species)								
**Coulteria mollis* Kunth	Way NMLW 28 (K)	Venezuela	KF522187	KX379403	KX373051	KX372887	-	-
*Coulteria platyloba* (S. Watson) N. Zamora	Gagnon & Marazzi, EG2010.007 (MT)	USA, cultivated	KF522175	KX379407	KX373057	KX372894	-	-
*Coulteria platyloba* (S. Watson) N. Zamora	Espinoza, BioBot01994 BOLD rec.: MHPAF1646-11	Costa Rica	-	-	-	-	-	JQ587526
*Coulteria platyloba* (S. Watson) N. Zamora	Lorea Lozada 685 (MEXU)	Mexico	KF522183	-	-	-	-	-
*Coulteria platyloba* (S. Watson) N. Zamora	MacQueen 178 (K)	Mexico	KF522178	KX379410	KX373059	KX372892	-	-
*Coulteria platyloba* (S. Watson) N. Zamora	Steinmann 3199 (INIREB, K)	Mexico	KF522184	-	-	-	-	-
*Coulteria platyloba* (S. Watson) N. Zamora	Espinoza, BioBot01995 BOLD rec.: MHPAF1647-11	Costa Rica	-	-	-	-	-	JQ587527
*Coulteria platyloba* (S. Watson) N. Zamora	Espinoza, BioBot01996 BOLD rec.: MHPAF1648-11	Costa Rica	-	-	-	-	-	JQ587528
***Caesalpinia colimensis* F. J. Herm.**	Sousa 6163 (K)	Mexico	**KF522176**	**KX379409**	**KX373058**	**KX372893**	-	-
***Caesalpinia colimensis* F.J. Herm.**	Sousa 7659 (TEX)	Mexico	-	-	-	-	**AF430713**	-
***Caesalpinia pringlei* (Britton & Rose) Standl.**	Cruz Duran 926 (MEXU)	Mexico	**KF522180**	-	-	-	-	-
***Caesalpinia pringlei* (Britton & Rose) Standl.**	Panero 4037 (TEX)	Mexico	-	-	-	-	**AF430732**	-
*Caesalpinia pumila* (Britton & Rose) F.J.Herm.	Gagnon & Marazzi EG 2010.014 (MT)	USA, cultivated	KF522182	KX379405	KX373054	KX372889	-	-
*Caesalpinia pumila* (Britton & Rose) F.J.Herm.	Lewis et al. 2067 (K)	Mexico	KF522177	KX379406	KX373055	KF379234	KF379385	KX176832
*Caesalpinia pumila* (Britton & Rose) F.J.Herm.	Nabhan et al. 1988 (MEXU)	Mexico	KF522185	-	-	-	-	-
*Caesalpinia pumila* (Britton & Rose) F.J.Herm.	Cavan 5535 (TEX)	Mexico	-	-	-	-	AF430720	-
*Caesalpinia velutina* (Britton & Rose) Standl.	Hughes et al. 2087 (FHO)	Mexico	KF522189	KX379404	KX373053	KX372888	-	-
*Caesalpinia velutina* (Britton & Rose) Standl.	Lewis 1797 (NY)	Mexico	KF522179	KX379408	KX373056	KX372891	-	-
*Caesalpinia velutina* (Britton & Rose) Standl.	Tenorio 296 (MEXU)	Mexico	KF522191	-	-	-	-	-
*Caesalpinia velutina* (Britton & Rose) Standl.	Torres 1590 (MEXU)	Mexico	KF522186	-	-	-	-	-
*Caesalpinia velutina* (Britton & Rose) Standl.	Way et al. JIC 22176 (K)	Mexico	KF522190	KX379402	KX373052	KX372890	-	-
*Caesalpinia velutina* (Britton & Rose) Standl.	Hughes 255 (FHO)	Guatemala	AY899752	-	-	-	AY899694	-
*Caesalpinia velutina* (Britton & Rose) Standl.	Torres 10741 (K)	Mexico	-	-	-	-	AF430741	-
*Caesalpinia violacea* (Mill.) Standl.	Lewis et al. 1763 (NY)	Mexico	KF522188	KX379401	KX373050	-	KX373100	JX099334
*Caesalpinia violacea* (Mill.) Standl.	Tenorio 4442 (MEXU)	Mexico	KF522181	-	-	-	-	-
***Erythrostemon*** (Hook.) Klotzsch (9/9 species)								
**Erythrostemon gilliesii* Klotzsch	Marazzi et al. BM131 (CTES, Z)	Argentina	AM086914	-	-	-	-	AM086829
**Erythrostemon gilliesii* Klotzsch	Spellenberg 12701 (MT)	USA, cultivated	KF522296	KP003786	KP003729	KP003681	JX073265	JX099328
**Erythrostemon gilliesii* Klotzsch	Jodrell 688-86 (K)	Chile	-	-	-	AY549891	AF430721	-
**Erythrostemon gilliesii* Klotzsch	Wojciechowski 882 (ASU)	USA	-	-	-	-	-	AY386845
**Erythrostemon gilliesii* Klotzsch	Hick & Bertone 34 (CORD)	Argentina	-	-	-	-	JX219458	-
*Erythrostemon calycinus* (Benth) L.P. Queiroz	Giulietti 2045 (HUEFS)	Brazil	KF522304	KX379278	KX373075	-	-	-
*Erythrostemon calycinus* (Benth) L.P. Queiroz	Lewis & Andrade 2003 (K)	Brazil	AY899749	-	-	-	KX373110	-
*Erythrostemon calycinus* (Benth) L.P. Queiroz	Lewis & Andrade 1885 (K)	Brazil	KF522303	-	KX373074	KX372895	AF430710	EU361899
*Caesalpinia angulata* (Hook & Arn.) Baill.	Brownless et al. 591 (E)	Chile	KF522288	KX379303	KX373080	KX372902	-	-
*Caesalpinia angulata* (Hook & Arn.) Baill.	Nee 37585 (K)	Chile	KF522287	-	-	-	-	-
*Caesalpinia argentina* Burkart	Hughes et al. 2460 (FHO)	Bolivia	KF522289	KX379309	KX373088	KX372908	-	-
*Caesalpinia argentina* Burkart	Pennington et al. 13323 (K)	Bolivia	KF522290	KX379310	KX373089	KX372909	-	-
***Caesalpinia caudata* (A. Gray) Fisher**	Simpson I-IV-01-3 (TEX)	USA	**KF522298**	**KX379277**	**KX373084**	-	-	-
***Caesalpinia caudata* (A. Gray) Fisher**	Neff 99-3-16-1(TEX)	USA	-	-	-	-	**AF430712**	-
*Caesalpinia coluteifolia* Griseb.	Gagnon et al. EG207 (MT)	Argentina	KF522291	KX379311	KX373090	KX372903	-	-
*Caesalpinia coluteifolia* Griseb.	Gagnon & Atchison EG223 (MT)	Argentina	KF522292	KX379312	KX373091	KX372904	-	-
*Caesalpinia coulterioides* Griseb. emend Burkart	Gagnon & Atchison EG209 (MT)	Argentina	KF522285	KX379308	KX373081	KX372910	-	-
*Caesalpinia exilifolia* Griseb.	Gagnon et al. EG201 (MT)	Argentina	KF522295	KX379307	KX373085	KX372907	-	-
*Caesalpinia exilifolia* Griseb.	Gagnon & Atchison EG219 (MT)	Argentina	KF522293	KX379306	KX373086	KX372906	-	-
***Caesalpinia exilifolia* Griseb.**	Gagnon & Atchison EG222 (MT)	Argentina	**KX373134**	**KX379257**	**KX373087**	**KX372905**	-	-
***Caesalpinia exilifolia* Griseb.**	Galleto 167 (CORD)	Argentina	-	-	-	-	**AF430716**	-
*Caesalpinia fimbriata* Tul.	Hughes et al. 2441 (FHO)	Bolivia	KF522284	KP003785	KP003728	KP003680	-	-
*Caesalpinia cf. fimbriata* Tul.	Hughes et al. 2466 (FHO)	Bolivia	KF522286	KX379304	KX373082	KX372911	-	-
*Caesalpinia fimbriata* Tul.	Wood 10627 (K)	Bolivia	KF522211	-	-	-	-	-
*Caesalpinia fimbriata* Tul.	Solomon & Nee 16062 (NY)	Bolivia	KF522297	KX379305	KX373083	KX372912	KX373111	-
***Guilandina*** L. (6/7-18 species)								
**Guilandina bonduc* L.	Bruneau 1342 (MT)	Madagascar	KF522062	KX379370	KX372967	KX372797	-	KX176816
**Guilandina bonduc* L.	van Balooy s.n., Krukoff coll. (K)	Malaysia	KF522063	-	-	-	AF430708	-
**Guilandina bonduc* L.	Herendeen 9-XII-97-3 (US)	Tanzania	-	-	-	KF379229	KX373103	-
**Guilandina bonduc* L.	Espinoza, BioBot02010 BOLD rec.: MHPAF1662-11	Costa Rica	-	-	-	-	-	JQ587518
**Guilandina bonduc* L.	Espinoza, BioBot02011 BOLD rec.: MHPAF1663-11	Costa Rica	-	-	-	-	-	JQ587519
**Guilandina bonduc* L.	Espinoza, BioBot02012 BOLD rec.: MHPAF1664-11	Costa Rica	-	-	-	-	-	JQ587520
*Guilandina ciliata* Wikstr.	Ekman 5413 (K)	Haiti	KX373125	-	-	-	-	-
*Guilandina ciliata* Wikstr.	Walker 51 (K)	British Virgin Islands	KX373126	KX379372	-	KX372798	-	-
*Guilandina major* L.	Herendeen & Pooma 30-IV-1999-1 (US)	USA, cultivated	KF522253	KX379374	KX372965	-	KX373104	-
*Caesalpinia minax* Hance	Li Shi Jin 802 (CAS, IBSC)	China	KF522131	KX379369	-	KX372926	-	-
*Caesalpinia minax* Hance	Living collection National Botanic Garden of Belgium 19645275(BR)	China, cultivated	KF522132	-	-	-	-	-
*Caesalpinia minax* Hance	PS1368MT01, GenBank	N/A	-	-	-	GU217664	-	HM049550
*Caesalpinia murifructa* Gillis & Proctor	Gillis 13096 (K)	Bahamas	KF522064	KX379373	KX372966	KX372799	-	-
*Caesalpinia volkensii* Harms	Archbold 2861 (K)	Tanzania	KF522065	-	-	-	-	-
*Caesalpinia volkensii* Harms	Friis et al. 3516 (K)	Ethiopia	KF522066	KX379375	KX372968	KX372800	-	-
*Caesalpinia volkensii* Harms	Somers s.n., RBG Liv.Coll. 1978-891 (K)	Kenya	KF522067	KX379371	KX372969	KX372801	-	-
***Haematoxylum*** L. (3/5 species)								
**Haematoxylum campechianum* L.	Bruneau 1313 (MT)	Mexico	KF522200	KX379329	KX373039	-	-	-
**Haematoxylum campechianum* L.	du Puy et al. M356 (K)	Madagascar	KF522208	-	-	-	-	-
**Haematoxylum campechianum* L.	Hughes 1273 (FHO)	Guatemala	AY899754	-	-	-	AY899697	-
**Haematoxylum campechianum* L.	Miller & Morello 8849 (MO)	Dominica	KF522201	KX379328	KX373038	KX372832	-	-
*Haematoxylum brasiletto* H. Karst.	Bernandes et al. 891 (MO)	Colombia	KF522209	KX379325	KX373042	KX372831	-	-
*Haematoxylum brasiletto* H. Karst.	Gagnon & Marazzi EG2010.011 (MT)	USA, cultivated	KF522207	KX379327	KX373041	KX372834	-	-
***Haematoxylum brasiletto* H. Karst.**	Gagnon & Marazzi EG2010.013 (MT)	USA, cultivated	**KF522206**	**KX379326**	**KX373040**	**KX372833**	-	-
***Haematoxylum brasiletto* H. Karst.**	Haston V200307 (RNG), OFI 14/83 (OFI)	Mexico	-	-	-	-	**AY899696**	-
***Haematoxylum brasiletto* H. Karst.**	Wojciechowski 953 (ASU)	USA	-	-	-	-	-	**AY386905**
*Haematoxylum brasiletto* H. Karst.	Lewis et al. 2057 (FHO)	Mexico	AY899753	-	-	-	AY899695	-
*Haematoxylum brasiletto* H. Karst.	Simpson 17-XI-97 (TEX)	USA	-	-	-	-	AF430777	-
***Haematoxylum dinteri* Harms**	Sucheach s.n. (OFI), Haston V200308 (RNG)	Namibia	AY899755	-	-	-	**AY899698**	-
***Haematoxylum dinteri* Harms**	Millennium seed bank project, HK2728 (K)	Namibia	**KX373135**	**KX379324**	**KX373037**	**KX372830**	-	-
***Hoffmannseggia*** Cav. (24/24 species)								
**Hoffmannseggia glauca* (Ortega) Eifert	Gagnon & Marazzi EG2010.05 (MT)	USA	KF522214	KX379318	KX372941	KX372792	-	-
**Hoffmannseggia glauca* (Ortega) Eifert	Gagnon & Marazzi EG2010.19 (MT)	USA	KF522212	KX379319	KX372940	KX372793	-	-
**Hoffmannseggia glauca* (Ortega) Eifert	Wojciechowski 1501 (ASU)	USA	-	-	-	-	-	JQ619977
**Hoffmannseggia glauca* (Ortega) Eifert	Spellenberg 12699 (MT)	USA	KF522213	KP003796	KP003744	KP003690	AF365069	EU361969
**Hoffmannseggia glauca* (Ortega) Eifert	Hick & Bertone 5 (CORD)	Argentina	-	-	-	-	JX219459	JX219465
**Hoffmannseggia glauca* (Ortega) Eifert	Cocucci 15-VI-1991 (CORD)	Argentina	-	-	-	X	AF430747	-
**Hoffmannseggia glauca* (Ortega) Eifert	Simpson 91-VII-22-1 (TEX)	Mexico	-	-	-	X	AY308488	-
*Hoffmannseggia aphylla* (Phil.) G.P. Lewis & Sotuyo	Gardner & Knees 6503 (E)	Chile	KF522146	KX379314	KX372937	KX372923	-	-
*Hoffmannseggia aphylla* (Phil.) G.P. Lewis & Sotuyo	Gardner & Knees 6557 (E)	Chile	KF522144	-	-	-	-	-
*Hoffmannseggia arequipensis* Ulibarri	Simpson 20-II-00-1 (TEX)	Peru	-	-	-	AY308550	AY308483	-
*Hoffmannseggia arequipensis* Ulibarri	Simpson 20-II-00-2 (TEX)	Peru	-	-	-	AY308551	AY308484	-
*Hoffmannseggia doelli* Philippi	Simpson 11-II-00-2 (TEX)	Chile	-	-	-	AY308552	AY308485	-
Hoffmannseggia doelli subsp. argentina Ulibarri	Gagnon & Atchison EG220 (MT, K)	Argentina	KX373136	KX379320	KX372943	KX372791	-	-
*Hoffmannseggia drepanocarpa* A. Gray	Simpson 29-V-89 (TEX)	Mexico	-	-	-	AY308553	AF430745	-
*Hoffmannseggia drummondii* Torr. & A. Gray	Simpson 05-15-92-2 (TEX)	Mexico	-	-	-	AY308554	AF430747	-
*Hoffmannseggia erecta* Philippi	Chumley 7379 (TEX)	Argentina	-	-	-	AY308555	AY308486	-
*Hoffmannseggia eremophila* (Phil.) Ulibarri	Aranoi & Sequeo 10334 (CORD)	Chile	-	-	-	AY308556	AY308487	-
*Hoffmannseggia humilis* (M. Martens & Galeotti) Hemsl.	Mayfield et al. 898 (TEX)	Mexico	-	-	-	AY308559	AF430748	-
*Hoffmannseggia intricata* Brandegee	Irwin 2371 (TEX)	Mexico	-	-	-	AY308560	AY308489	-
*Hoffmannseggia microphylla* Torr.	Holmgren 6505 (NY)	USA	KF522145	KX379315	KX372938	KX372920	-	-
*Hoffmannseggia microphylla* Torr.	Simpson 03-15-03-1 (TEX)	Mexico	-	-	-	AY308561	AF430749	-
*Hoffmannseggia minor* (Phil.) Ulibarri	Simpson 1-II-00-9 (TEX)	Argentina	-	-	-	AY308562	AY308534	-
*Hoffmannseggia miranda* Sandwith	FLSP 945 (NY)	Peru	KF522239	KX379321	KX372946	KX372796	-	-
*Hoffmannseggia miranda* Sandwith	Hughes & Daza 2358 (FHO)	Peru	KF522240	KX379322	KX372945	KX372795	-	-
*Hoffmannseggia miranda* Sandwith	Simpson 22-II-00-2 (TEX)	Peru	-	-	-	AY308565	AY308492	-
*Hoffmannseggia miranda* Sandwith	Simpson 21-II-00-1 (TEX)	Peru	-	-	-	AY308564	AY308491	-
*Hoffmannseggia miranda* Sandwith	Dillon & Dillon 3958 (F)	Peru	-	-	-	AY308563	AF430750	-
Hoffmannseggia oxycarpa Benth. subsp. arida (Rose) B. B. Simpson	Simpson 91-VII-21-2 (TEX)	Mexico	-	-	-	AY308566	AF430751	-
*Hoffmannseggia peninsularis* (Britton) Wiggins	Simpson 03-15-93-5 (TEX)	Mexico	-	-	-	AY308567	AF430752	-
*Hoffmannseggia prostrata* DC.	Hughes & Daza 2359 (FHO)	Peru	KF522241	KX379323	KX372944	KX372794	-	-
*Hoffmannseggia prostrata* DC.	Dillon & Dillon 5926 (F)	Chile	-	-	-	AY308568	AF430753	-
*Hoffmannseggia pumilio* (Griseb.) B.B. Simpson	Gagnon & Atchison EG221 (MT, K)	Argentina	KX373137	KX379268	-	KX372919	-	-
*Hoffmannseggia pumilio* (Griseb.) B.B. Simpson	Simpson 1-II-00-1 (TEX)	Argentina	-	-	-	AY308549	AF430791	-
*Hoffmannseggia repens* (Eastw.) Cockerell	Simpson 27-V-89-7 (TEX)	USA	-	-	-	AY308569	AF430755	-
*Hoffmannseggia tenella* Tharp & L.O. Williams	Neff 4-XI-88 (TEX)	USA	-	-	-	AY308570	AF430755	-
*Hoffmannseggia ternata* DC.	Dillon & Dillon 3746 (F)	Peru	-	-	-	AY308571	AF430756	-
*Hoffmannseggia ternata* DC.	Simpson 22-II-00-1 (TEX)	Peru	-	-	-	AY308574	AY308495	-
*Hoffmannseggia ternata* DC.	Simpson 15-II-00-1 (TEX)	Chile	-	-	-	AY308572	AY308493	-
*Hoffmannseggia ternata* DC.	Simpson 21-II-00-2 (TEX)	Peru	-	-	-	AY308573	AY308494	-
*Hoffmannseggia ternata* DC.	Simpson 22-II-00-3 (TEX)	Peru	KF522139	-	-	AY308575	AY308496	-
*Hoffmannseggia trifoliata* Cav.	Simpson 21-I-00-3A (TEX)	Argentina	-	-	-	AY308576	AY308497	-
*Hoffmannseggia viscosa* Hook.& Arn.	Eastwood et al. RJE35 (FHO)	Peru	KF522138	KX379317	KX372942	KX372924	-	-
*Hoffmannseggia viscosa* Hook.& Arn.	Hughes et al. 2221 (FHO)	Peru	KF522137	KX379316	KX372939	KX372925	-	-
*Hoffmannseggia viscosa* Hook.& Arn	Sagastegui 11465 (MO)	Peru	-	-	-	AY308577	AY308498	-
*Hoffmannseggia viscosa* Hook.& Arn	Richardson 2039	Peru	-	-	-	AY308578	AY308499	-
*Hoffmannseggia watsonii* (Fisher) Rose	Hunter 25354 (TEX)	Mexico	-	-	-	AY308579	AY308500	-
*Hoffmannseggia yaviensis* Ulibarri	Simpson 30-I-00-1 (TEX)	Argentina	-	-	-	AY308580	AY308501	-
***Libidibia*** (DC.) Schltdl. (6/6-8 species)								
**Libidibia coriaria* (Jacq.) Schltdl.	Fougère-Danezan 20 (MT)	Singapore, cultivated	KF522109	KX379423	KX373008	-	-	-
****Libidibia coriaria* (Jacq.) Schltdl.**	**Hughes 1495 (K)**	**Mexico**	AY899750	-	-	-	**AY899692**	-
****Libidibia coriaria* (Jacq.) Schltdl.**	**Hughes et al. 2163 (FHO)**	**Mexico**	**KF522107**	**KP003797**	**KP003745**	**KP003691**	-	
****Libidibia coriaria* (Jacq.) Schltdl.**	**Espinoza, BioBot00788** **BOLD rec.: MHPAD924-09**	**Costa Rica**	-	-	-	-	-	**JQ587521**
**Libidibia coriaria* (Jacq.) Schltdl.	Espinoza, BioBot00789 BOLD rec.: MHPAD925-09	Costa Rica	-	-	-	-	-	JQ587522
**Libidibia coriaria* (Jacq.) Schltdl.	Espinoza, BioBot00790 BOLD rec.: MHPAD926-09	Costa Rica	-	-	-	-	-	JQ587523
*Libidibia ferrea* (Mart. ex Tul.) L.P. Queiroz	Fougère-Danezan 21 (MT)	Singapore, cultivated	KF522105	KX379425	KX373014	-	JX073260	EU361901
*Libidibia ferrea* (Mart. ex Tul.) L.P. Queiroz	Lewis et al. 1623 (K)	Brazil	KF522114	-	-	-	-	-
*Libidibia ferrea* (Mart. ex Tul.) L.P. Queiroz	Kew Living coll. 1973-21715 (K)	Brazil	-	-	-	-	AF430718	-
*Libidibia glabrata* (Kunth) C.Cast. & G.P. Lewis	Delgado 2097 (MEXU)	Peru	KF522103	-	-	-	-	-
*Libidibia glabrata* (Kunth) C.Cast. & G.P. Lewis	Eastwood et al. RJE84 (FHO)	Peru	KF522102	KX379427	KX373010	KX372913	-	-
*Libidibia glabrata* (Kunth) C.Cast. & G.P. Lewis	Lewis & Lozano 3043 (K)	Ecuador	KF522101	KX379428	KX373012	-	KX373101	-
*Libidibia glabrata* (Kunth) C.Cast. & G.P. Lewis	Särkinen et al. 2151 (FHO)	Peru	KF522104	KX379429	KX373011	-	-	-
*Libidibia glabrata* (Kunth) C.Cast. & G.P. Lewis	Lewis & Klitgaard 3337 (K)	Ecuador	-	-	-	-	AF430722	-
*Libidibia paraguariensis* (Parodi) G.P. Lewis	Hughes et al. 2307 (FHO)	Bolivia	KF522110	KX379420	KX373006	-	-	-
*Libidibia paraguariensis* (Parodi) G.P. Lewis	Hughes et al. 2475 (FHO)	Bolivia	KF522111	KX379421	KX373007	-	-	-
*Libidibia paraguariensis* (Parodi) G.P. Lewis	Lewis & Klitgaard 2170 (K)	Argentina	KF522112	KX379419	KX373005	KF379233	KX373119	EU361905
*Libidibia paraguariensis* (Parodi) G.P. Lewis	Zardini & Velazquez 19907 (K)	Paraguay	KF522113	-	-	-	-	-
*Libidibia punctata* (Willd.) Britton	Cardenas 4071 (K)	Venezuela	KF522106	KX379424	KX373015	-	-	-
***Libidibia sclerocarpa* (Standl.) Britton & Rose**	Lewis & Hughes 1778 (K)	Mexico	**KF522108**	**KX379426**	**KX373013**	-	-	-
***Libidibia sclerocarpa* (Standl.) Britton & Rose**	Kew seed collection s.n.	Mexico	-	-	-	-	**AF430736**	-
***Lophocarpinia*** Burkart (1/1 species)								
**Lophocarpinia aculeatifolia* (Burkart) Burkart	Fortunato 8639 (BAB)	Argentina	-	-	-	-	JX219460	JX219466
***Mezoneuron*** Desf. (11/26 species)								
*Mezoneuron andamanicum* Prain	Herendeen 29-IV-1999-1 (US)	Thailand	KF522305	-	KX372957	KX372815	-	AY386931
*Mezoneuron angolense* Welw. ex Oliv.	Herendeen 12-XII-97-1 (US)	Tanzania	-	-	-	-	AF365068	EU361897
*Mezoneuron benthamianum* Baill.	Ern 2602 (K)	Togo	KF522196	KX379388	KX372960	KX372818	-	-
*Mezoneuron benthamianum* Baill.	Morton & Jarr SL3295 (K)	Sierra Leone	KF522195	KX379387	KX372959	KX372817	-	-
*Mezoneuron benthamianum* Baill.	Vigne 3487 (FHO)	Ghana	KF522197	-	-	-	-	-
*Mezoneuron cucullatum* (Roxb.) Wight & Arn.	Grierson & Long 3623 (K)	Bhutan	KF522194	KX379266	-	KX372819	-	-
*Mezoneuron deverdiana* Guillaumin	McPherson 6211 (K)	New Caledonia	KF522078	-	-	-	-	-
*Mezoneuron hildebrandtii* Vatke	Lewis et al. 2137 (K)	Madagascar	KF522198	KX379386	KX372958	KX372816	KX373107	KX176807
*Mezoneuron hildebrandtii* Vatke	Simpson 17-XI-97 (TEX)	Madagascar	-	-	-	-	AF430780	-
*Mezoneuron hymenocarpum* Prain	Larsen & Larsen 34232 (K)	Thailand	-	KX379390	KX372956	KX372820	-	-
*Mezoneuron kauaiensis* (H. Mann) Hillbr.	Lorence & Wagner 8904 (NTBG)	Hawaii, USA	KF522192	KX379391	KX372961	KX372823	EU361770	EU361903
*Mezoneuron kauaiensis* (H. Mann) Hillbr.	Melville 71/1033 (K)	Hawaii, USA	-	-	-	KX372822	-	-
*Mezoneuron scortechinii* F. Muell.	Wieringa et al. 4195 (WAG)	Australia	KF522077	KX379394	KX372964	KF379231	KX373106	KX176821
*Mezoneuron sumatranum* (Roxb.) Wight & Arn.	Beaman 9642 (NY, MO)	Malaysia	KF522199	-	-	KX372821	-	-
*Mezoneuron montrouzieri* Guillaumin	Pullen 7619 (K)	Papua New Guinea	KF522193	-	-	-	-	-
*Caesalpinia erythrocarpa* Pedley	Schodde 2246 (K)	Papua New Guinea	KF522257	KX379393	KX372963	KX372813	-	-
*Caesalpinia nitens* (F. Muell ex Benth.) Pedley	Bean 18033 (MO)	Australia	KF522076	KX379392	KX372962	KX372814	-	-
***Moullava*** Adans. (1/1 species)								
**Moullava spicata* (Dalzell) Nicolson	Critchett 11/79 (K)	Zambia, cultivated	KF522252	KX379378	-	KX372805	JX073267	KX176818
**Moullava spicata* (Dalzell) Nicolson	Hutchison 2784 (TEX)	Sri Lanka	-	-	-	-	AF430782	-
***Poincianella*** Britton & Rose (32/~35 species)								
**Poincianella mexicana* (A. Gray) Britton & Rose	Hughes et al. 1606 (NY, FHO)	Mexico	KF522218	KX379296	KX373061	-	EU361772	EU361904
**Poincianella mexicana* (A. Gray) Britton & Rose	Delgado 01-2114 (MEXU)	Mexico	KF522219	-	-	-	EF177387	-
**Poincianella mexicana* (A. Gray) Britton & Rose	Lewis s.n., Kew Living Coll. 1973-21714 (K)	Mexico	KF522215	KP003788	KP003730	KP003683	-	-
**Poincianella mexicana* (A. Gray) Britton & Rose	Gagnon & Marazzi EG2010.015 (MT)	USA, cultivated	KF522217	KX379295	KX373062	-	-	-
**Poincianella mexicana* (A. Gray) Britton & Rose	Mason s.n. (K)	USA	-	-	-	AY549892	AF430727	-
*Poincianella aff. mexicana*	Contreras s.n. (MEXU)	Mexico	KF522227	-	-	-	-	-
*Poincianella acapulcensis* (Standl.) Britton & Rose	Lott 3205 (K)	Mexico	KF522233	-	-	-	-	-
*Poincianella acapulcensis* (Standl.) Britton & Rose	MacQueen et al. 406 (K)	Mexico	KF522235	KX379280	KX373065	-	-	-
*Poincianella bracteosa* (Tul.) L.P. Queiroz	Carvalho-Sobrinho 218 (HUEFS)	Brazil	KF522035	KX379449	KX372983	-	-	-
*Poincianella bracteosa* (Tul.) L.P. Queiroz	de Queiroz 10085 (HUEFS)	Brazil	KF522079	KX458251	-	-	-	-
*Poincianella bracteosa* (Tul.) L.P. Queiroz	de Queiroz7845 (HUEFS)	Brazil	KF522036	KX379448	-	-	-	-
*Poincianella bracteosa* (Tul.) L.P. Queiroz	(retrieved from GenBank)	Brazil	-	-	-	DQ787395	-	-
*Poincianella caladenia* (Standl.) Britton & Rose	Contreras 2868 (MEXU)	Mexico	KF522234	-	-	-	-	-
***Poincianella caladenia* (Standl.) Britton & Rose**	Contreras 2818 (MEXU)	Mexico	-	-	-	-	**EF177383**	-
***Poincianella caladenia* (Standl.) Britton & Rose**	Lewis et al. 2072 (K)	Mexico	**KF522228**	**KX379285**	**KX373066**	-	-	-
*Poincianella eriostachys* (Benth.) Britton & Rose	Hughes 1832 (K)	Mexico	AY899751	-	-	-	AY899693	-
*Poincianella eriostachys* (Benth.) Britton & Rose	Lewis et al. 1799 (K)	Mexico	KF522029	KX379444	KX372993	KX372836	AF430715	-
*Poincianella eriostachys* (Benth.) Britton & Rose	MacQueen 449 (MEXU)	Mexico	-	-	-	-	EF177389	-
*Poincianella exostemma* (DC.) Britton & Rose	Contreras s.n. Febrero 2000 (MEXU)	Mexico	KF522237	-	-	-	-	-
Poincianella exostemma (DC.) Britton & Rose subsp. exostemma	Bruneau 1317 (MT)	Mexico	KF522221	KX379292	KX373072	-	-	-
Poincianella exostemma (DC.) Britton & Rose subsp. exostemma	Lewis & Hughes 1712, RBG Liv.Coll. 1989-3073 (K)	Guatemala	KF522224	KX379290	-	-	AF430717	-
Poincianella exostemma (DC.) Britton & Rose subsp. exostemma	Lewis & Hughes 1753 (K)	Guatemala	KF522222	KX379291	KX373071	-	-	-
*Poincianella exostemma* (DC.) Britton & Rose	Espinoza, BioBot00766 BOLD rec.: MHPAD902-09	Costa Rica	-	-	-	-	-	JQ587524
*Poincianella exostemma* (DC.) Britton & Rose	Espinoza, BioBot00767 BOLD rec.: MHPAD903-09	Costa Rica	-	-	-	-	-	JQ587525
*Poincianella gaumeri* (Greenm.) Britton & Rose	Calzada 19333 (K, MEXU)	Mexico	KF522030	-	-	-	-	-
*Poincianella gaumeri* (Greenm.) Britton & Rose	Hughes 492 (K)	Mexico	KF522034	KX379445	-	-	-	-
*Poincianella gaumeri* (Greenm.) Britton & Rose	Lewis & Hughes 1762 (K)	Mexico	KF522044	KP003799	KP003739	KP003692	-	-
*Poincianella glandulosa* (DC.) Britton & Rose	Ekman 9838 (K)	Haiti	KX373138	KX379279	-	-	-	-
*Poincianella laxa* (Benth.) Britton & Rose	Delgado 2337 (MEXU)	Mexico	KF522274	-	-	-	-	-
*Poincianella laxiflora* (Tul.) L.P. Queiroz	de Queiroz 7063 (HUEFS)	Brazil	KF522051	KX379440	-	KX372849	-	-
*Poincianella laxiflora* (Tul.) L.P. Queiroz	Lewis & Andrade 2012 (MO)	Brazil	-	-	-	KX372929	-	-
*Poincianella melanadenia* (Rose) Britton & Rose	Hughes et al. 2091 (FHO)	Mexico	KF522275	KX379301	KX373078	-	-	-
*Poincianella melanadenia* (Rose) Britton & Rose	Contreras 7369 (MEXU)	Mexico	KF522277	-	-	-	-	-
***Poincianella melanadenia* (Rose) Britton & Rose**	Hughes et al. 2074 (FHO)	Mexico	**KF522276**	**KX379302**	-	**KX372896**	-	-
***Poincianella melanadenia* (Rose) Britton & Rose**	Sotuyo and Gonzalez s.n. (MEXU)	Mexico	-	-	-	-	**DQ208904**	-
*Poincianella microphylla* (Mart. ex. G. Don) L.P. Queiroz	Coradin et al. 5941 (K)	Brazil	KF522040	-	-	-	-	-
*Poincianella microphylla* (Mart. ex. G. Don) L.P. Queiroz	de Queiroz 9060 (HUEFS)	Brazil	KF522039	KX379450	KX372984	KX372847	-	-
*Poincianella nelsonii* Britton & Rose	Contreras & Sotuyo s.n. (MEXU)	Mexico	KF522300	KX379281	KX373070	-	-	-
*Poincianella nelsonii* Britton & Rose	Lewis et al. 1794 (K)	Mexico	-	-	-	-	AF430728	-
*Poincianella nelsonii* Britton & Rose	Sotuyo s.n., RBG Liv.Coll. 2002-3577 (K)	Mexico	KF522301	KP003789	KP003731	KP003684	EF177385	-
*Poincianella palmeri* (S. Watson) Britton & Rose	Gagnon et al. EG2010.010 (MT)	USA, cultivated	KF522230	KX379286	KX373067	-	-	-
*Poincianella palmeri* (S. Watson) Britton & Rose	Gagnon et al. EG2010.023 (MT)	USA, cultivated	KF522229	KX379284	KX373068	-	-	-
*Poincianella palmeri* (S. Watson) Britton & Rose	Lewis 2064 (K)	Mexico	KF522232	-	-	-	-	-
*Poincianella palmeri* (S. Watson) Britton & Rose	Lewis et al. 2065 (K)	Mexico	KF522231	KP003790	KP003732	KP003685	KX373113	KF379243
*Poincianella pannosa* (Standl.) Britton & Rose	Gentry 4365 (MEXU)	Mexico	KF522283	-	-	-	-	-
*Poincianella pannosa* (Standl.) Britton & Rose	Lewis 2051 (K)	Mexico	KF522282	KP003791	KP003734	KP003686	KX373112	-
*Poincianella pannosa* (Standl.) Britton & Rose	Turner s.n. (TEX)	Mexico	-	-	-	AY549890	AY535804-AY535817	-
*Poincianella pellucida* (Vogel) Britton & Rose	Ekman 4999 (K)	Haiti	-	KX379452	-	-	-	-
*Poincianella phyllanthoides* (Standl.) Britton & Rose	Nee 32666 (K)	Mexico	KF522220	KX379294	KX373060	-	-	-
*Poincianella phyllanthoides* (Standl.) Britton & Rose	Steinmann 3718 (INIREB, MEXU)	México	KF522216	-	-	-	-	-
*Poincianella placida* (Brandegee) Britton & Rose	Lewis et al. 2032 (K)	Mexico	KF522273	KP003792	KP003735	KP003687	-	-
*Poincianella placida* (Brandegee) Britton & Rose	Lewis 2046 (K)	Mexico	KF522272	KP003792	X	KP003687	KX373122	
*Poincianella pluviosa* (DC.) L.P. Queiroz	de Queiroz 12795 (HUEFS)	Brazil	KF522049	KP003800	KP003735	KP003687	-	-
*Poincianella pluviosa* (DC.) L.P. Queiroz	Wood et al. 26552 (K)	Bolivia	KF522047	X	KX372987	KX372840	-	-
Poincianella pluviosa (DC.) L.P. Queiroz var. pluviosa	Wood 8838 (K)	Bolivia	KF522052	KX379442	-	KX372842	-	-
Poincianella pluviosa (DC.) L.P. Queiroz var. pluviosa	Nee 40000 (K)	Bolivia	KF522054	-	-	KX372841	-	-
*Poincianella pluviosa* (DC.) L.P. Queiroz	Nee 38223 (TEX)	Bolivia	-	-	-	-	AF430731	
Poincianella pluviosa var. peltophoroides (DC.) L.P. Queiroz	Lewis et al. 1632 (K, NY)	Brazil	-	-	-	KX372848	-	-
Poincianella pluviosa var. sanfranciscana (G.P. Lewis) L.P. Queiroz	Lewis & Andrade 1896 (K)	Brazil	KF522050	KX379443	KX372990	KX372850	-	-
*Poincianella pyramidalis* (Tul.) L.P. Queiroz	Dorea 117 (HUEFS)	Brazil	KF522041	KX379441	KX372985	KX372851	-	-
*Poincianella pyramidalis* (Tul.) L.P. Queiroz	de Queiroz 9020 (HUEFS)	Brazil	KF522042	KX379451	KX372986	KX372852	-	-
*Poincianella pyramidalis* (Tul.) L.P. Queiroz	Taylor et al. 1361 (MO, NY)	Brazil	-	-	-	KX372930	-	-
*Poincianella pyramidalis* (Tul.) L.P. Queiroz	Mori & Boom 14207 (K)	Brazil	KF522038	-	-	-	-	-
*Poincianella pyramidalis* (Tul.) L.P. Queiroz	Sampaio s.n. (retrieved from GenBank)	Brazil	-	-	-	-	-	JX850053
*Poincianella standleyii* Britton & Rose	Contreras 2745 (K)	Mexico	KF522236	KX379282	KX373069	-	-	-
Poincianella yucatanensis (Greenm.) Britton & Rose subsp. yucatanensis	Lewis 1765 (K)	Mexico	KF522280	KX379288	KX373063	-	AF430743	
Poincianella yucatanensis (Greenm.) Britton & Rose subsp. yucatanensis	Lewis & Hughes 1766 (K, NY)	Mexico	KF522281	KX379289	-	-	-	-
*Caesalpinia coccinea* G.P. Lewis & J.L. Contr.	Lewis 1802 (K)	Mexico	KF522225	KX379283	-	KX372901	-	-
*Caesalpinia coccinea* G.P. Lewis & J.L. Contr.	Lewis 1803 (K)	Mexico	KF522226	-	-	-	EF177386	-
*Caesalpinia echinata* Lam.	Filgueiras 3391 (NY)	Brazil, cultivated	KF522099	KP003802	KP003746	KP003695	KX373105	-
*Caesalpinia echinata* Lam.	Lewis et al. 1624 (K)	Brazil	KF522072	KX379412	KX373029	KX372866	-	KX176825
*Caesalpinia echinata* Lam.	Miranda 76 (HUEFS)	Brazil	KF522100	KX379411	KX373044	KX372867	KX373121	KX176822
***Caesalpinia epifanioi* J.L. Contr.**	Contreras 2039 (K)	Mexico	**KF522278**	**KP003787**	**KP003733**	**KP003682**		-
***Caesalpinia epifanioi* J.L. Contr.**	Sotuyo et al. 63 (MEXU)	Mexico	-	-	-	-	**DQ208901**	-
*Caesalpinia epifanioi* J.L. Contr.	Sotuyo & Sotuyo 20 (MEXU)	Mexico	KF522279	-	-	-	-	-
*Caesalpinia hintonii* Sandwith.	Sotuyo 46 (MEXU)	Mexico	KF522270	KX379299	KX373076	KX372897	DQ208882	-
*Caesalpinia hughesii* G.P. Lewis	Lewis et al. 1795 (K)	Mexico	KF522223	KX379293	KX373073	-	AF430725	-
*Caesalpinia macvaughii* J.L. Contr. & G.P. Lewis	Sotuyo et al. 8 (MEXU)	Mexico	KF522299	KX379297	KX373077	KX372898	KX373108	-
*Caesalpinia macvaughii* J.L. Contr. & G.P. Lewis	Sotuyo et al. 54 (MEXU)	Mexico	KF522269	-	-	-	-	-
*Caesalpinia macvaughii* J.L. Contr. & G.P. Lewis	Steinmann 3175 (INIREB, K, MEXU)	Mexico	KF522268	KX379298	-	-	DQ208916	-
*Caesalpinia marginata* Tul.	Dubs 1746 (K)	Brazil	KF522045	-	-	KX372839	-	-
*Caesalpinia marginata* Tul.	Wood et al. 26514 (K)	Bolivia	KF522048	KX379438	KX372989	KX372838	-	-
*Caesalpinia marginata* Tul.	Wood et al. 26561 (K)	Bolivia	KF522046	KX379439	KX372988	KX372837	-	KX176808
*Caesalpinia nicaraguensis* G.P. Lewis	Hawkins & Hughes 4 (K)	Nicaragua	KF522302	-	-	KX372899	-	-
*Caesalpinia oyamae* Sotuyo & G.P. Lewis	Hawkins & Hughes 23 (FHO, MEXU, TEX)	Mexico	KF522210	KX379300	KX373079	-	AF430724	-
Caesalpinia pluviosa var. maraniona G.P. Lewis & C.E. Hughes	Hughes et al. 2215 (FHO)	Peru	KF522033	KX379436	KX372996	KX372844	KX373120	-
Caesalpinia pluviosa var. maraniona G.P. Lewis & C.E. Hughes	Hughes et al. 3105 (MT)	Peru	KF522032	KX379437	KX372997	KX372843	-	KX176836
Caesalpinia pluviosa var. maraniona G.P. Lewis & C.E. Hughes	Pennington et al. 793 (E, K)	Peru	KF522031	KX379434	KX372995	KX372845	-	-
Caesalpinia pluviosa var. maraniona G.P. Lewis & C.E. Hughes	Särkinen et al. 2191 (FHO)	Peru	KF522043	KX379435	KX372994	KX372846	-	-
Caesalpinia yucatanensis subsp. chiapensis G.P. Lewis	Hughes 1353 (FHO)	Mexico	KF522271	KX379287	KX373064	KX372900	-	-
***Pomaria*** Cav. (15/16 species)								
**Pomaria glandulosa* Cav.	Ventura & López 9294 (TEX)	Mexico	KF522088	-	-	AY549901	AY535823-AY535810	-
*Pomaria austrotexana* B.B. Simpson	Simpson 1-IV-01-2 (TEX)	USA	-	-	-	AY549895	AF430757	-
*Pomaria brachycarpa* (A. Gray) B.B. Simpson	Simpson 92-06-22-3 (TEX)	USA	-	-	-	AY549896	AF430758	-
*Pomaria burchellii* (DC.) B.B. Simpson & G.P. Lewis	Mott 766 (MO)	South Africa	-	-	-	AY549897	AY535819-AY535806	-
*Pomaria burchellii* (DC.) B.B. Simpson & G.P. Lewis	Klepper 252/A/42 (PRU)	South Africa	-	-	-	AY549898	AF430744	-
*Pomaria canescens* (Fisher) B.B. Simpson	Turner et al. 93-128 (TEX)	Mexico	-	-	-	AY549899	AY535820-AY535807	-
*Pomaria fruticosa* (S. Watson) B.B. Simpson	Villareal 4439 (TEX)	Mexico	-	-	-	AY549901	AY535822-AY535809	-
*Pomaria jamesii* (Torr. & A. Gray) Walp.	Gagnon & Marazzi EG2010.020 (MT)	USA	KF522089	KX379313	KX372947	-	-	KX176830
*Pomaria jamesii* (Torr. & A. Gray) Walp.	Higgins 17628 (NY)	USA	KF522090	KP003793	KP003736	KP003677	EU361830	EU362029
*Pomaria lactea* (Schinz) B.B. Simpson & G.P. Lewis	Pearson 9742 (MO)	South Africa	-	-	-	AY549904	AY535824-AY535811	-
*Pomaria melanosticta* S. Schauer	Simpson 92-06-23-1 (TEX)	USA	-	-	-	AY549905	AF430760	-
*Pomaria multijuga* (S. Watson) B.B. Simpson	Engard 649 (TEX)	Mexico	-	-	-	AY549906	AY535825- AY535812	-
*Pomaria pilosa* (Vogel) B.B. Simpson & G.P. Lewis	Wasum et al. 4571 (NY)	Brazil	-	-	-	AY549900	AY535821-AY535808	-
*Pomaria pilosa* (Vogel) B.B. Simpson & G.P. Lewis	Wasum & Bastos 8008 (NY)	Brazil	-	-	-	AY549907	AY535824-AY535813	-
*Pomaria rubicunda* (Vogel) B.B. Simpson & G.P. Lewis	Biganzoli et al. s.n. (NY)	Argentina	KF522085	KP003795	KP003738	KP003679	EU361775	-
*Pomaria rubicunda* (Vogel) B.B. Simpson & G.P. Lewis	Lima 463 (HUEFS)	Brazil	KP003642	KP003794	KP003737	KP003678	-	-
Pomaria rubicunda (Vogel) B.B. Simpson & G.P. Lewis var. rubicunda	Vanni & Marunak 3755 (NY)	Argentina	-	-	-	AY549909	AY535827-AY535814	-
Pomaria rubicunda var. hauthalii (Harms) B.B. Simpson & G.P. Lewis	Ibarrola 1750 (US)	Argentina	KF522087	-	-	AY549908	AF430723	-
*Pomaria sandersonii* (Harv.) B.B. Simpson & G.P. Lewis	Hilliard & Burtt 9225 (MO)	South Africa	-	-	-	AY549910	AY535828-AY535815	-
*Pomaria stipularis* (Vogel) B.B. Simpson & G.P. Lewis	Jönsson 1002a (A)	Brazil	KF522086	-	-	AY549911	AF430739	-
*Pomaria wootonii* (Britton) B.B. Simpson	Johnston 4341 (TEX)	Mexico	-	-	-	AY549912	AY535829-AY535816	-
***Pterolobium*** R. Br. ex Wight & Arn. (4/10 species)								
**Pterolobium stellatum* (Forssk.) Brenan	Herendeen 17-XII-97-9 (US)	Tanzania	KF522238	KX379457	-	KX372812	KX373115	EU362032
**Pterolobium stellatum* (Forssk.) Brenan	Briden & al. RNB219 (JRAU) BOLD rec. KNPA1387-09	South Africa	-	-	-	-	-	JF270908
**Pterolobium stellatum* (Forssk.) Brenan	Albers 63080 (TEX)	Ethiopia	-	-	-	-	AF430783	-
*Pterolobium hexapetalum* (Roth) Santapau & Wagh	Grierson & Long 2075 (P)	Bhutan	KX373139	-	KX372973	KX372806	-	-
*Pterolobium integrum* Craib	van Beusekom 4021 (P)	Thailand	-	KX379456	-	-	-	-
*Pterolobium macropterum* Kurz	Grierson & Long 1624 (P)	Bhutan	KX373141	KX379454	KX372974	-	-	-
*Pterolobium macropterum* Kurz	Geesink & al. 5934 (P)	Thailand	KX373140	KX379455	-	-	-	-
***Stahlia*** Bello (1/1 species)								
**Stahlia monosperma* (Tul.) Urb.	Gardner 7029 (E)	Dominican Republic	KX373142	KX379422	KX373009	-	EU361838	EU362050
**Stahlia monosperma* (Tul.) Urb.	Proctor 48543 (MO)	Puerto Rico	-	-	-	-	AF430787	-
***Stenodrepanum*** Harms (1/1 species)								
**Stenodrepanum bergii* Harms	Hick & Bertone 8 (CORD)	Argentina	-	-	-	-	JX219461	JX219467
**Stenodrepanum bergii* Harms	Hick & Bertone 16 (CORD)	Argentina	-	-	-	-	JX219462	-
***Stuhlmannia*** Taub. (1/1 species)								
**Stuhlmannia moavi* Taub.	Luke 3710 (MO, K)	Tanzania	KF522061	KX379431	KX373001	KX372829	-	-
**Stuhlmannia moavi* Taub.	Keraudren-Aymonin & Aymonin 25628 (MO)	Madagascar	KF522060	KX379433	KX373000	KX372828	-	-
**Stuhlmannia moavi* Taub.	Tanner 2404 (NY)	Tanzania	-	-	-	-	AF430789	-
**Stuhlmannia moavi* Taub.	Luke & Robertson 2336 (K)	Kenya	KF522058	-	-	-	-	-
**Stuhlmannia moavi* Taub.	Robertson 7509 (K)	Kenya	KF522059	KX379432	KX372999	KX372827	EU361839	KX176819
**Stuhlmannia moavi* Taub.	Tanner 3167 (K)	Tanzania	AY899765	-	-	-	AY899707	-
***Tara*** Molina (3/3 species)								
**Tara spinosa* (Molina) Britton & Rose	Eastwood et al. RJE36 (FHO)	Peru	KF522128	KX379398	KX373046	-	-	KF379250
**Tara spinosa* (Molina) Britton & Rose	Hughes 2360 (FHO)	Peru	KF522129	KX379399	KX373045	KX372881	-	-
**Tara spinosa* (Molina) Britton & Rose	Nee 45494 (MO)	Australia, cultivated	KF522130	KX379400	KX373047	KX372880	-	-
**Tara spinosa* (Molina) Britton & Rose	Lewis 2200 (K)	Ecuador	-	-	-	KF379235	KX373099	-
**Tara spinosa* (Molina) Britton & Rose	Aronson 7756 (TEX)	Chile	-	-	-	-	AF430738	-
*Tara cacalaco* (Humb. & Bonpl.) Molinari & Sánchez Och.	Gagnon & Marazzi EG2010.022 (MT)	USA, cultivated	KF522202	KX379396	-	KX372885	-	-
*Tara cacalaco* (Humb. & Bonpl.) Molinari & Sánchez Och.	Soto Nuñez 13682 (MEXU)	Mexico	KF522312	-	-	-	-	-
*Tara cacalaco* (Humb. & Bonpl.) Molinari & Sánchez Och.	Walker s.n., RBG Liv.Coll. 1986-6481 (K)	Mexico	KF522203	KX379397	KX373048	KX372886	-	-
***Tara cacalaco* (Humb. & Bonpl.) Molinari & Sánchez Och.**	Lewis 1789 (K)	Mexico	-	-	-	-	**AF430709**	**EU361898**
***Tara cacalaco* (Humb. & Bonpl.) Molinari & Sánchez Och.**	Lewis 1788 (K)	Mexico	-	-		**KX372884**	-	-
*Tara vesicaria* (L.) Molinari, Sánchez Och. & Mayta	Hawkins & Hughes 11 (FHO)	Nicaragua	KF522204	KX379395	KX373049	KX372882	-	-
*Tara vesicaria* (L.) Molinari, Sánchez Och. & Mayta	Lewis & Hughes 1768 (K)	Mexico	KF522205	-	-	KX372883	AF430742	-
***Zuccagnia*** Cav. (1/1 species)								
**Zuccagnia punctata* Cav.	Fortunato 5545 (MO)	Argentina	KF522142	KX379417	KX373002	KX372917	-	-
**Zuccagnia punctata* Cav.	Galleto et al. 171 (CORD)	Argentina	KF522141	KP003798	KP003742	KP003688	AF430791	KX176813
**Zuccagnia punctata* Cav.	Guglianone et al. 1668 (K, SI)	Argentina	KF522143	KX379418	KX373003	KX372916	-	-
**Zuccagnia punctata* Cav.	Lutz 136 (NY)	Argentina	KF522140	KX379416	KX373004	KX372918	EU361842	-
**Zuccagnia punctata* Cav.	Tapia & al. s.n. (CORD)	Argentina	-	-	-	-	JX219463	JX219468
**Unassigned Old World taxa (13/~20 species)**								
*Caesalpinia crista* L.	Herendeen 1-V-99-3 (US)	Thailand	KF522073	KX379384	KX372971	KX372807	KX373094	EU361900
*Caesalpinia crista* L.	Wieringa et al. 4199 (WAG)	Australia, cultivated	KF522074	KX379385	KX372972	KX372808	-	-
*Caesalpinia crista* L.	PS1367MT01 (retrieved from GenBank)	N/A	-	-	-	-	-	HM049549
*Caesalpinia decapetala* (Roth) Alston	Marazzi BM137 (Z)	Switzerland, cultivated	AM086910	-	-	-	-	AM086828
*Caesalpinia decapetala* (Roth) Alston	Hughes et al. 2227 (FHO)	Peru, cultivated	KF522081	KX379353	KX372978	KX372922	-	-
*Caesalpinia decapetala* (Roth) Alston	Hooper & Gandhi 2429 (US)	India, cultivated	KF522080	-	-	-	-	-
*Caesalpinia decapetala* (Roth) Alston	Herendeen & Mbago 19-XII-97-1 (US)	Tanzania	KF522082	KX379354	KX372979	KX372921	KX373098	KX176817
*Caesalpinia decapetala* (Roth) Alston	PS1589MT01 (retrieved from GenBank)	N/A	-	-	-	GU217669	-	HM049555
*Caesalpinia decapetala* (Roth) Alston	Corby 2173; Krukoff coll. (K)	N/A	-	-	-	-	AF430714	-
*Caesalpinia decapetala* (Roth) Alston	(retrieved from GenBank)	N/A	-	-	-	JF708207	-	-
*Caesalpinia digyna* Rottler	van Beusekom & Phengklai 3036 (P)	Thailand	KX373144	KX379381	-	-	-	-
*Caesalpinia digyna* Rottler	Maxwell 91-827 (P)	Thailand	KX373146	KX379383	-	KX372803	-	-
*Caesalpinia digyna* Rottler	Parnell et al. 95-617 (K)	Thailand	KX373145	KX379265	-		-	-
*Caesalpinia digyna* Rottler	Cheng et al. CL 643 (P)	Cambodia	KX373143	KX379382	-	KX372802	-	-
*Caesalpinia godefroyana* Kuntze	Cheng et al. CL 642 (P)	Cambodia	KX373147	KX379351	KX372976	KX372809	-	-
*Caesalpinia mimosoides* Lam.	Larsen et al. 44653 (MO)	Thailand	KF522251	KX379357	KX372955	-	-	-
*Caesalpinia mimosoides* Lam.	Clark RPC237 (K)	Thailand	KX373148	KX379262	KX372954	-	KX373093	-
*Caesalpinia oppositifolia* Hattink	Lugas 607 (K)	Malaysia	KF522056	KX379355	-	-	-	-
*Caesalpinia oppositifolia* Hattink	Lugas 921 (K)	Malaysia	KF522055	KX379356	KX372970	KX372810	-	-
*Caesalpinia parviflora* Prain	van Beusekom et al. 3977 (K)	Thailand	KF522057	KX379389	KX372977	KX372811	-	-
*Caesalpinia pearsonii* Bolus	Strey 2475 (K)	Namibia	KX373150	KX379377	KX373017	KX372825	-	KX176826
*Caesalpinia pearsonii* Bolus	Kolberg & Loots, NAM2943-HK1399 (K)	Namibia	KX373149	KX379376	KX373016	KX372824	KX373123	KX176827
*Caesalpinia milettii* Hook. & Arn.	Ying 1639 (K)	China	-	KX379260	-	-	-	-
^a^***Caesalpinia sappan* L.**	Jinawn 76 (K)	Sabah	-	**KX379261**	-	-	-	-
^a^***Caesalpinia sappan* L.**	PS1370MT04, (retrieved from GenBank)	N/A	-	-	-	**GQ434751**	-	-
^a^***Caesalpinia sappan* L.**	PS1370MT05, (retrieved from GenBank)	N/A	-	-	-	-	-	**HM049952**
^b^***Caesalpinia sappan* L.**	Gillis 9548 (P)	India	-	**KX379352**	-	-	-	-
^b^***Caesalpinia sappan* L.**	(retrieved from GenBank)	N/A	-	-	-	**EU243573**	-	-
^b^***Caesalpinia sappan* L.**	PS1370MT01, (retrieved from GenBank)	N/A	-	-	-	-	-	**HM049551**
*Caesalpinia tortuosa* Roxb.	Lace 6332 (K)	Burma	-	KX379264	-	KX372804	-	-
*Caesalpinia vernalis* Benth.	Li Shi Jin 787 (CAS, IBSC)	China	KF522075	-	-		-	-
*Caesalpinia welwitschiana* (Oliv.) Brenan	Bidgood et al. 2913 (K)	Tanzania	KF522133	KX379379	-	-	-	-
*Caesalpinia welwitschiana* (Oliv.) Brenan	Malaisse 13658 (K)	Zaire	KF522134	KX379380	KX372975	-	-	KX176831

a Accessions with the same prefix were combined together.

b Accessions with the same prefix were combined together.

## Supplementary materials

Supplementary material 1 Table 3. Summary of the branch support results from phylogenetic analyses of the Caesalpinia group. Data type: results from phylogenetic analyses Explanation note: Bootstrap support from the ML analyses of the six individual loci and the combined datasets, as well as Bootstrap support and Posterior probabilities from the parsimony and Bayesian analyses of the combined datasets, for various proposed genera from Gagnon et al. (2013). This dataset is made available under the Open Database License (http://opendatacommons.org/licenses/odbl/1.0/). The Open Database
License (ODbL) is a license agreement intended to allow users to freely share, modify,
and use this Dataset while maintaining this same freedom for others, provided that the
original source and author(s) are credited.Edeline Gagnon, Anne Bruneau, Colin E. Hughes, Luciano Paganucci De Queiroz, Gwilym P. Lewis
